# Towards a General Understanding of Carbonyl‐Stabilised Ammonium Ylide‐Mediated Epoxidation Reactions

**DOI:** 10.1002/chem.201602052

**Published:** 2016-07-06

**Authors:** Johanna Novacek, Lukas Roiser, Katharina Zielke, Raphaël Robiette, Mario Waser

**Affiliations:** ^1^ Institute of Organic ChemistryJohannes Kepler University LinzAltenbergerstrasse 694040LinzAustria; ^2^ Institute of Condensed Matter and NanosciencesUniversité catholique de LouvainPlace Louis Pasteur 1 box L4.01.021348Louvain-la-NeuveBelgium

**Keywords:** chiral auxiliary, density functional calculations, diastereoselectivity, enantioselectivity, ylides

## Abstract

The key factors for carbonyl‐stabilised ammonium ylide‐mediated epoxidation reactions were systematically investigated by experimental and computational means and the hereby obtained energy profiles provide explanations for the observed experimental results. In addition, we were able to identify the first tertiary amine‐based chiral auxiliary that allows for high enantioselectivities and high yields for such epoxidation reactions.

## Introduction

Onium ylides have found widespread applications for (dia)stereoselective epoxide, aziridine and cyclopropane syntheses.^1^, ^2^, ^3^, ^4^ Among the different classes of onium ylides that can be used for such three‐ring‐forming reactions, sulfur ylides have been the most frequently used ones, and a variety of applications by using achiral or chiral sulfur ylides (either preformed or generated in situ with catalytic quantities of a chiral sulfide) have been reported since.^3^ In contrast to the privileged use of sulfonium ylides, easily available ammonium ylides have been less routinely employed in the past.^5^, ^6^, ^7^, ^8^, ^9^, ^10^ Especially, the use of chiral amines to render such reactions enantioselective has so far mainly been limited to cyclopropanation reactions, a methodology that was impressively developed by Gaunt et al.^5^ However, their use in asymmetric epoxidation and aziridination reactions was found to be rather difficult,^6^, ^7^, ^9^ which can mainly be explained by the weaker leaving‐group ability of the amine group as compared to the use of sulfonium ylides.^11^

**Scheme 1:**
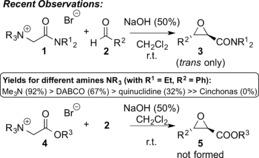
Recent observations made in ammonium ylide‐mediated syntheses of epoxides (DABCO=1,4‐diazabicyclo[2.2.2]octane).

Our group has recently introduced a highly *trans*‐selective protocol for the synthesis of glycidic amides^7^ and analogous aziridines9b starting from ammonium acetamides **1** (which are in situ deprotonated to give the corresponding amide‐stabilised ammonium ylides) (Scheme 1). Hereby, several important observations were made: First, the reaction is strongly dependent on the nature of the used amine leaving group, with trimethylamine being clearly superior to other achiral amines,7b a trend that was also observed when using benzylic ammonium ylides.6d Unfortunately, attempts to carry out this reaction in an enantioselective fashion by using chiral Cinchona alkaloids as the amine leaving group (which is the method of choice for cyclopropanations^5^) failed completely.7b, 12 In addition, under the developed conditions, this methodology does not give any epoxides **5** when using the more stabilised ester‐based ammonium salts **4**.^13^

Based on these results we became interested in investigating this reaction in more detail, especially by computational means. Our goals were to rationalise the different reactivities of esters and amide derivatives and to understand the high *trans* selectivity in this reaction. We also would like to compare the hereby obtained energy profiles with those of analogous sulfur ylide‐mediated reactions to understand the different reactivities of sulfur and ammonium ylides. In addition, as Cinchona alkaloids failed as chiral auxiliaries for such reactions, we decided to investigate the use of alternative (synthetic) chiral amines for their potential to facilitate this reaction in an enantioselective manner.

## Results and Discussion

### Computational studies

**Figure 1:**
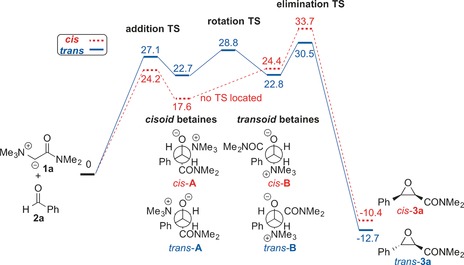
Computed free energy profiles [kcal mol^−1^] for the formation of the *cis* and *trans* glycidic amides **3 a** from the ammonium ylide **1 a**.

In order to address the above‐mentioned questions, we have investigated the free energy profile of the parent reaction between the amide‐stabilised ylide **1 a** and benzaldehyde (**2 a**) (Figure 1). Calculations were carried out at the B3LYP‐D3/6‐311+G**//B3LYP/6‐31G* level of theory,^14^ including a continuum description of dichloromethane as solvent.

The mechanistic sequence is similar to that of previously studied ylide‐mediated epoxidation reactions: addition of the ylide to the aldehyde generates a *cisoid* betaine intermediate through a [2+2]‐type approach, torsional rotation converts the later into the *transoid* conformer and finally ring closure, with concomitant expulsion of the amine, gives the corresponding epoxide.

As a result of the stabilisation of the ylide by an amide group, the initial addition step is endergonic and the *cisoid* betaines *cis*‐**A** and *trans*‐**A** lying at 17.6 and 22.7 kcal mol^−1^ above the reactants.^15^ Noteworthy, the *cisoid cis*‐**A** betaine is more stable than the *cisoid trans*‐**A** betaine. However, a significant difference for the required bond rotation towards the *transoid* betaines **B** and the following ring closure can be observed for the two diastereomeric pathways leading to *cis*‐**3 a** or *trans‐* **3 a**. First, the required bond rotation to the *transoid cis*‐**B** betaine is an endergonic step (6.8 kcal mol^−1^) for which no transition state (TS) could be located and therefore, the *cis*‐**B** conformer does not represent a thermodynamic local minimum.^16^ In contrast, the corresponding *trans*‐**A** and *trans*‐**B** betaines are of similar stability and the bond rotation between these conformations occurs with a free energy barrier of 6.1 kcal mol^−1^. Finally, the ring‐closure step is found to be the rate‐limiting step in both cases, even if the elimination TS is only slightly higher than the rotation TS for the *trans*‐**B** betaine. Accordingly, the selectivity is determined at the elimination step and the higher stability of the *trans* elimination TS (by 3.2 kcal mol^−1^) as compared to the *cis* elimination TS accounts for the observed high *trans* selectivity.

**Scheme 2:**
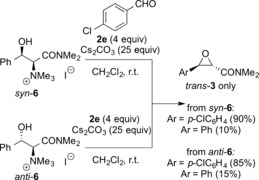
Experimental proof of the reversibility of the betaine formation by crossover experiments.

To prove that betaine formation is indeed a reversible process, we carried out crossover experiments in analogy to previous studies on sulfur ylides by the group of Aggarwal.3f When reacting the racemic *syn* and *anti* β‐hydroxy ammonium salts **6** with an excess of a more reactive aldehyde, such as *p*‐chlorobenzaldehyde, we found that only the *trans* epoxides **3** are formed (Scheme 2). In addition, the more reactive aldehyde is incorporated predominantly, giving 90 % of the corresponding epoxide starting from *syn‐* **6** and 85 % starting from *anti*‐**6** as precursor. Accordingly, these results clearly show that betaine formation is indeed highly reversible for both diastereomeric pathways, although slightly more for the *cis* one.

**Figure 2:**
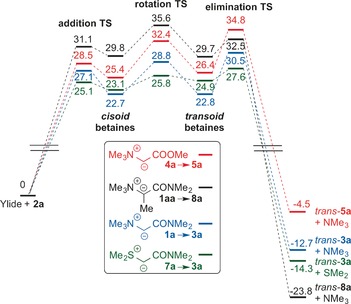
Computed free energy profiles [kcal mol^−1^] for the epoxidation by using the ammonium ylides **1 a**, **1 aa**, and **4 a**, as well as the sulfonium ylide **7 a**.

Aggarwal et al. have carefully investigated the energy profile for epoxidation reactions of amide‐stabilised sulfur ylides in the past and it was found that hereby also the elimination step is the selectivity‐determining step.3f To elucidate the reactivity difference between ammonium and sulfonium ylide‐mediated epoxidation reactions we thus re‐addressed this transformation (considering inclusion of entropic and thermal contributions and a dispersion correction, which were not included previously3f). Our calculations clearly confirm the earlier conclusions by Aggarwal et al.3f that indeed the ring closure is the selectivity‐determining step when using amide‐stabilised sulfonium ylides. As it can be seen in Figure 2, the overall energy profile for the epoxidation mediated by the sulfur ylide **7 a** is energetically lower than for the one mediated by the ammonium ylide **1 a** (green vs. blue pathway in Figure 2). This confirms the higher overall barrier for ammonium ylides as compared to sulfonium ones. This lower reactivity of compound **1 a** can be accounted for by a significantly higher barrier to elimination due to the poorer leaving‐group ability of ammonium (7.7 kcal mol^−1^), as compared to sulfonium (2.7 kcal mol^−1^).^11^

During our earlier investigations in this project, we also tried to use the propionamide‐based ammonium ylide **1 aa** for the epoxidation reaction. However, under neither conditions we were able to obtain any product. By analysing the corresponding free energy profile for this transformation (Figure 2, black pathway) it becomes obvious that the whole reaction is significantly higher in energy as compared to the use of the acetamide‐based ylides **1**. Noteworthy, is the fact that the increased steric demand in the α‐position leads to a significantly higher rotation barrier, which becomes the rate‐limiting step hereby. This observation can also serve as a useful explanation to understand the lower yield when using the sterically more demanding Cinchona alkaloid leaving groups as outlined in Scheme 1.

We also used the opportunity to compare the drastic influence of an ester group on the reactivity. As mentioned in the beginning, the so far developed reaction conditions did not allow us to obtain any epoxides **5** when using the ester‐based ammonium salts **4** (Scheme 1).^7^ Analysing this observation by DFT calculations reveals that the free energy profile for the reaction of the ylide **4 a** involves indeed a high overall barrier (34.8 kcal mol^−1^) (Figure 2). This lower reactivity is mainly due to a higher stabilisation of the ylide **4 a** as compared to compound **1 a**, which leads to a more endothermic betaine formation. The free energy barrier for the elimination is also slightly increased with the more electron‐withdrawing ester group.^17^

**Scheme 3:**
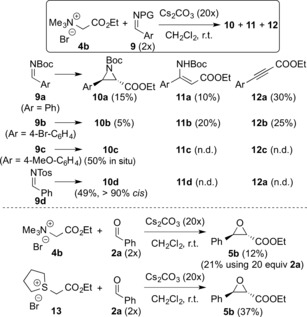
Reactions of ester‐stabilised ammonium and sulfonium ylides.

With these illustrative data in hand, we became interested in testing whether it may be possible to use the ester‐stabilised ammonium ylides **4** for aziridination reactions instead. It was shown in the past by Aggarwal and co‐workers that aziridinations by using ester‐stabilised sulfonium ylides are possible, whereas the corresponding epoxidations are very difficult.4b By using the ammonium salt **4 b** for aziridinations with the imines **9**, we found that indeed some aziridine **10 a** can be formed under carefully optimised reaction conditions by using an excess of solid Cs_2_CO_3_ as the base (Scheme 3). However, this reaction is accompanied by the formation of significant amounts of the α,β‐unsaturated α‐amino ester **11 a** and the alkyne **12 a**. Although the formation of the alkenes **11** is a side reaction that is often observed in ammonium ylide‐mediated aziridination reactions, especially with electron‐poor imines,7c, 9b the formation of the alkyne **12 a** came unexpected. By changing the electronic properties of the imine **9**, we found that formation of compound **11** increases when using the bromine‐substituted imine **9 b**, whereas the presence of an electron‐donating group, such as a methoxy group (i.e., compound **9 c**), only gave the aziridine **10 c**, but none of the unsaturated side products **11 c** and **12 c** (the trend for the formation of the alkene **11** is in analogy to our recent observations7c, 9b). Control experiments showed that compound **12** is not formed from compound **11** but most probably elimination seems to occur from the intermediate betaines. By changing the N‐protecting group to a tosyl group (i.e., compound **9 d**) we only observed the formation of the *cis*‐aziridine **10 d**,^18^ but no formation of the products **11** and **12**.

To elucidate if the formation of compound **12** may be rationalised by an in situ hydrolysis of the *tert*‐butyloxycarbonyl (Boc)‐protected imines **9** to aldehydes **2** and reaction of compound **2** with the ylide, we carried out the direct reaction of compound **4 b** with benzaldehyde (**2 a**) (Scheme 3, lower part). Interestingly, we did not observe any formation of compound **12**, but instead small quantities of the epoxide **5 b** was isolated. This came as a big surprise as so far we have never been able to obtain even trace quantities of compound **5**. Obviously, the crucial role in this reaction seems to be the solid carbonate base. It was reported in the past that solid inorganic bases, that is, carbonates, can have a very special effect on sulfonium ylide‐mediated reactions.^19^ We thus wondered, whether the liquid–solid combination of CH_2_Cl_2_/Cs_2_CO_3_ may also allow us to increase the yield for the analogous sulfonium ylide‐mediated epoxidation by using ester **13**. We were indeed able to isolate the epoxide **5 b** in 37 % yield, which proves the positive effect of Cs_2_CO_3_ as compared to other previously used bases (i.e., *t*BuOK, KOH or K_2_CO_3_), but it must be admitted that this methodology could not further be improved by using alternative sulfur leaving groups or conditions.

Finally, we also calculated the energy profile for the reaction of the DABCO‐ and quinuclidine‐based amide‐stabilised ammonium ylides **1** to rationalise the significant yield differences when using them for epoxidation reactions7b (compare with Scheme 1).

**Figure 3:**
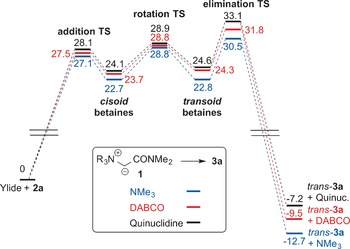
Computed free energy profiles [kcal mol^−1^] for the epoxidation by using trimethylamine, DABCO and quinuclidine‐based ammonium ylides **1**.^20^

First, these calculations show that the barrier to ring closure is higher for quinuclidine (8.5 kcal mol^−1^) as compared to trimethylamine (7.7 kcal mol^−1^) and DABCO (7.5 kcal mol^−1^), thus providing a reasonable explanation for the lower yields obtained with this leaving groups. However, this step alone does not explain why trimethylamine allows for significantly higher epoxidation yields than DABCO. As it can be seen in Figure 3, the whole energy profile for trimethylamine is energetically lower than for DABCO, which seems to be mainly due to a higher stabilisation of the DABCO ylide as compared to the trimethylamine derivative,^20^ thus resulting in an overall higher reactivity towards epoxidation of the trimethyl ammonium salts.

### Enantioselective epoxidation

Controlling the absolute configuration in ammonium ylide‐mediated epoxidation reactions has so far been a very challenging task. Although the use of Cinchona alkaloids is the method of choice for ammonium ylide‐mediated cyclopropanations,^5^ their use in epoxidation reactions does not allow for any product formation (Scheme 1).^6^, ^7^ Kimachi et al. showed that brucine can be used as a chiral leaving group for benzylic ammonium ylide‐based epoxidations.6b We have recently reported an alternative strategy by using chiral trimethylammonium‐based acetamides with a phenylglycinol‐based amide auxiliary,7c which allowed for high selectivities in epoxidation and aziridination reactions. However, based on the fact that this protocol requires the cleavage of the auxiliary in a subsequent step, a strategy by using a chiral amine leaving group in ammonium ylide‐mediated epoxidations would be much more appealing. Based on the low reactivity associated with the use of simple Cinchona alkaloids we therefore, decided to systematically screen a variety of other chiral tertiary amines. Table 1 gives an overview of the most significant results obtained in a detailed screening of different chiral tertiary amines under different reaction conditions.

**Table 1:** Identification of the best‐suited chiral amine leaving group for the enantioselective synthesis of the glycidic amide **3 b**. 
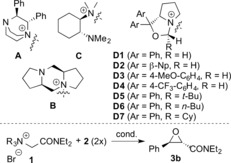

Entry	Amine	Conditions^[a]^	Yield [%]^[b]^	e.r.^[c]^
1	**A**	I	7	6:94
2	**A**	II	n.r.	n.d.
3	**B**	I	23	28:72
4	**B**	II	12	28:72
5	**C**	I	31	24:76
6	**C**	II	4	27:73
7	**D1**	I	62	76:24
8	**D1**	II	80	75:25
9	**D1**	III	85	78:22
10	**D2**	III	32	78:22
11	**D3**	III	30	67:33
12	**D4**	III	96	64:36
13	**D5**	III	69	92:8
14	**D6**	III	55	88:12
15	**D7**	III	88	93:7
16	**D7**	IV	66	95:5
17	**D7**	I	80	93:7

[a] I) NaOH (50 % aq., 20 equiv), CH_2_Cl_2_, RT, 24 h; II) Cs_2_CO_3_ (s, 20 equiv), CH_2_Cl_2_, RT, 24 h; III) Cs_2_CO_3_ (s, 20 equiv), *i*PrOH, RT, 24 h; IV) Cs_2_CO_3_ (s, 20 equiv), toluene, RT, 24 h; [b] Yield of the isolated compound. [c] Determined by HPLC by using a chiral stationary phase and given as (2*S*,3*R*)/(2*R*,3*S*) ratio. The absolute configuration was determined by comparison of the retention order with reported data.3e

Because DABCO itself was a reasonably good leaving group in our racemic approach (Scheme 1),7a we first focused on the known chiral DABCO derivative **A**.^**[**21]^ Under liquid/liquid biphasic conditions we obtained some product **3 b** in high enantiopurity (Table 1, entry 1). Unfortunately, the yield was rather low and no further improvement with alternative solvents and bases was possible (e.g., Table 1, entry 2). We next attempted the use of the proline dimer **B**,^**[**21]^ which unfortunately gave only a slightly higher yield, but with significantly lower enantioselectivity (Table 1, entries 3 and 4). Similar observations were made by using the *trans*‐cyclohexane diamine **C** (Table 1, entries 5 and 6) or derivatives thereof as the auxiliary. Finally, we reasoned that it may be possible to increase the leaving‐group ability of the amine by using an (hemi)aminal‐type structure with a less basic nitrogen.^23^ We thus, synthesised a small collection of the proline‐derived amines **D**.^24^, ^25^ Gratifyingly, already the use of the most simple derivative **D1** proved our hypothesis right, giving the target epoxide **3 b** in more than 60 % yield with a promising initial level of enantioselectivity (enantiomeric ratio (e.r.) =76:24) under biphasic liquid/liquid conditions (Table 1, entry 7). Testing alternative reaction conditions showed us that liquid/solid conditions by using Cs_2_CO_3_ as the base gave compound **3 b** in more than 80 % and comparable selectivity in solvents like *i*PrOH or dichloromethane (Table 1, entries 8 and 9). It should be noted that changing the reaction temperature did not have any beneficial effect and we thus, kept these room‐temperature conditions to further optimise the auxiliary next (Table 1, entries 9–15). Changing the aryl moiety did not allow us to improve the outcome (see Table 1, entries 9–12 for representative results) and as we were not able to introduce aliphatic groups instead^25^ we thus kept the phenyl residues and started varying the substituent R (see Table 1, entries 13–15 for representative details). We immediately realised that the introduction of sterically demanding aliphatic groups provides reasonably high selectivities (e.r.> 90:10) and after some fine‐tuning the cyclohexane‐based auxiliary **D7** 26 was found to be the most promising one to obtain compound **3 b** in high yield and high enantiopurity (Table 1, entries 15–17). Unfortunately, attempts to use this auxiliary in a catalytic fashion by starting from the α‐bromo‐acetamide (in analogy to Gaunt's cyclopropanation^5^) did not allow us to obtain reasonable quantities of the product (<5 % when using 10 mol % of amine catalyst but with a similar enantioselectivity as in the use of preformed ammonium salts). Furthermore, in‐situ reaction of an equimolar mixture of amine and α‐bromo‐acetamide with benzaldehyde also only resulted in a rather low yield of 10–15 % of epoxide (again with the same selectivity). This limited reactivity seems to be mainly because formation of the ammonium salt proceeds relatively slowly under the reaction conditions and in addition, the auxiliary itself undergoes instead a partial aminal hydrolysis under these conditions (this hydrolysis is slow when using the formaldehyde‐based auxiliary **D1** but faster with compounds **D5** and **D7**).

**Scheme 4:**
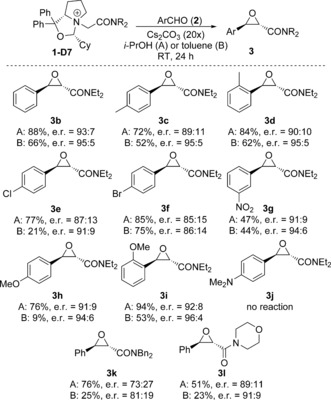
Application scope of the enantioselective epoxidation by using the chiral ammonium ylide‐precursor **1‐D7**.^27^

Having identified the first chiral *tert*‐amine auxiliary that warrants both, high yield and high enantioselectivity in ammonium ylide‐mediated epoxidation reactions to access the glycidic amides **3**, we next investigated the application scope of this methodology. As it can be seen in Scheme 4, a variety of aromatic aldehydes were usually tolerated.

In most cases, we found that higher yields can be obtained when using *i*PrOH as solvent as compared to toluene, which however, in a few cases, gave a slightly higher enantioselectivity. This solvent effect was most striking when using the *p*‐methoxybenzaldehyde **2 h** and it is still not clear why in this specific case the yield in toluene was so much lower. Interestingly, also nitrobenzaldehyde (**2 g**), which was found to be a problematic substrate in the past (mainly because of competing Cannizzaro disproportionation under the basic conditions),^7^ could be successfully employed under these reaction conditions giving the corresponding epoxide **3 g** with high selectivity and in a moderate yield. Only in the presence of the rather strongly electron‐donating dimethylamino group no product **3 j** could be obtained. Attempts to use enolisable aldehydes like cyclohexanecarbaldehyde or dodecanal showed low conversion rates (<10–20 % after 2–3 days) and a pronounced tendency towards byproduct formation (e.g., aldol reactions) what is in accordance with our earlier racemic approach.^7^ Changing the amide substituents on the other hand gave the products **3 k** and **3 l** in reasonable yields too. However, hereby it was found that especially the dibenzylamide group gave a significantly lower enantioselectivity than the usually used diethylamide group.

**Scheme 5:**
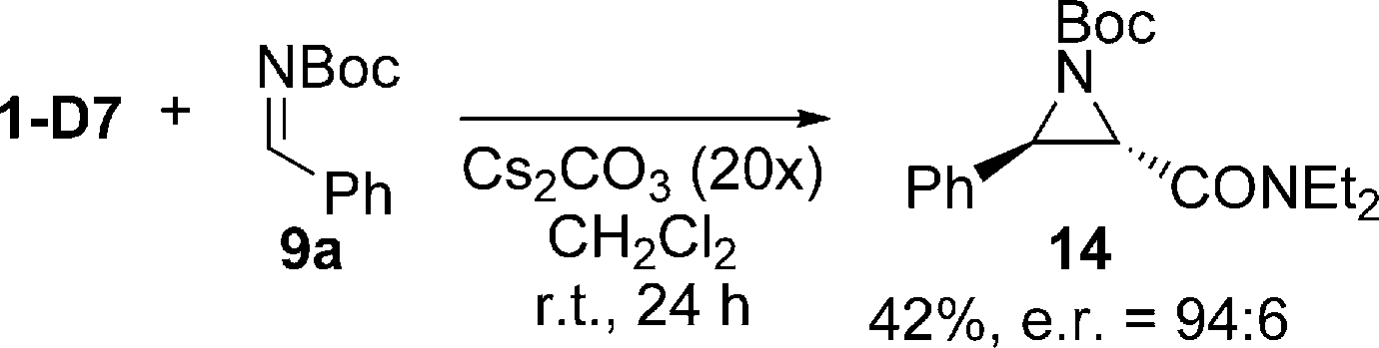
Proof of concept of the enantioselective aziridination by using the chiral ammonium salt **1‐D7**.

Encouraged by the successful use of the ammonium salt **1‐D7** for the highly enantioselective synthesis of the glycidic amides **3** we also wanted to investigate if this methodology allows the synthesis of the analogous aziridine **14** in an enantioenriched form. Gratifyingly, dichloromethane was found to be the solvent of choice to obtain compound **14** in a moderate yield of 42 % and a high enantiomeric ratio of 94:6 (Scheme 5, other solvents gave almost no product formation). This methodology in general thus also holds promise for the synthesis of chiral aziridines by using ammonium ylides, albeit in lower yields.

## Conclusion

Systematic experimental and computational studies allowed us to reveal the key factors for ylide‐mediated epoxidation reactions when using carbonyl‐stabilised ammonium ylides. The pronounced reactivity differences between esters and amides as well as the high diastereoselectivity and the different leaving‐group abilities of different amines were investigated and the hereby obtained energy profiles provide detailed explanations for the observed experimental results. In addition, we were able to identify the first tertiary amine‐based auxiliary that allows for high enantioselectivities and high yields for such epoxidation reactions. This represents a significant improvement compared to previous reports which mainly focused on the use of Cinchona alkaloid‐based structures and were not satisfactory for epoxidation reactions. The herein presented proline‐based auxiliary approach constitutes an alternative strategy allowing to overcome these limitations.

## Experimental Section

General details can be found in the Supporting Information. This document also contains detailed synthesis procedures for the auxiliaries and the epoxidation reactions and analytical data of the novel compounds and reaction products as well as copies of the NMR spectra and HPLC traces. The Supporting Information also includes the details of the computational investigations.

**General asymmetric epoxidation procedure**: The ammonium salt **1** was dissolved in the given solvent (A: 0.1 m in *i*PrOH, B: 0.1 m in toluene) and Cs_2_CO_3_ (20 equiv) were added followed by the addition of the corresponding carbaldehyde (2 equiv). The mixture was stirred at room temperature for 24 h (under an argon atmosphere). The reaction was quenched by addition of H_2_O and extracted with dichloromethane. The combined organic phases were dried over Na_2_SO_4_ and evaporated to dryness. Purification by column chromatography (gradient of heptane and EtOAc) gave the corresponding epoxides in the reported yields and enantiopurities.

**Epoxide 3 b**: Obtained as a white solid on a 0.5 mmol scale. Method A: 88 % yield and e.r.=93:7 and method B: 66 % yield and e.r.=95:5. [*α*]20D =106.7 (*c*=0.675, dichloromethane, e.r.=95:5); ^1^H NMR (300 MHz, CDCl_3_, 298 K): *δ*=1.17 (t, *J*=7.3 Hz, 3 H), 1.21 (t, *J*=7.3 Hz, 3 H), 3.39–3.52 (m, 4 H), 3.59 (d, *J*=1.8 Hz, 1 H), 4.10 (d, *J*=1.8 Hz, 1 H), 7.32–7.39 ppm (m, 5 H); ^13^C NMR (125 MHz, CDCl_3_, 298 K): *δ*=13.1, 15.1, 41.0, 41.6, 57.4, 57.7, 125.8, 128.6, 128.7,135.9, 165.9 ppm. The enantioselectivity was determined by HPLC (Chiralcel OD‐H, eluent: hexane/*i*PrOH=80:20, 0.5 mL min^−1^, 10 °C, retention times: *t*_major_ (2*S*,3*R*)=15.1 min, *t*_minor_ (2*R*,3*S*)=17.3 min).

## Supporting information

As a service to our authors and readers, this journal provides supporting information supplied by the authors. Such materials are peer reviewed and may be re‐organized for online delivery, but are not copy‐edited or typeset. Technical support issues arising from supporting information (other than missing files) should be addressed to the authors.

Supplementary
