# Supplementary material for: Towards a General Understanding of Carbonyl‐Stabilised Ammonium Ylide‐Mediated Epoxidation Reactions
Source: Chemistry. 2016 Jul 6;22(32):11422–8. doi: 10.1002/chem.201602052 (PMC5066843; doi:10.1002/chem.201602052)
Supplement: Supplementary file 1 — Supplementary [file CHEM-22-11422-s001.pdf]

CHEMISTRY
A European Journal

Supporting Information

Towards a General Understanding of Carbonyl-Stabilised Ammonium Ylide-Mediated Epoxidation Reactions

Johanna Novacek+,[a] Lukas Roiser+,[a] Katharina Zielke+,[a] Raphaël Robiette,*[b] and Mario Waser*[a]

chem_201602052_sm_miscellaneous_information.pdf

SUPPORTING INFORMATION

Towards a General Understanding of Carbonyl-Stabilised Ammonium Ylide-Mediated Epoxidation Reactions

Johanna Novacek,[a] Lukas Roiser,[a] Katharina Zielke,[a] Raphaël Robiette,[b,*] and Mario Waser[a,*]

a) Institute of Organic Chemistry, Johannes Kepler University Linz, Altenbergerstraße 69, 4040 Linz, Austria. Fax: +43 732 2468 8747; Tel: +43 732 2468 8748; E-mail: Mario.waser@jku.at

b) Institute of Condensed Matter and Nanosciences, Université catholique de Louvain, Place Louis Pasteur 1 box L4.01.02, 1348 Louvain-la-Neuve, Belgium. E-mail: raphael.robiette@uclouvain.be

1. General Information: 3
1.2. General Methods 3
1.2. Computational Methods 4
2. Syntheses 5
2.1 Reactions of Ester-Stabilised Ylides 5
2.2 Reactions of Amide-Stabilised Ylides 9
3. Computational results – Energies and Geometries 24
3.1 Reaction of amide-stabilized trimethylammonium ylide with benzaldehyde 24
3.2 Reaction of ester-stabilized trimethylammonium ylide with benzaldehyde 28
3.3 Reaction of amide-stabilized DABCO-ammonium ylide with benzaldehyde 31
3.4 Reaction of amide-stabilized quinculidine-ammonium ylide with benzaldehyde 33
3.5 Reaction of α-methyl amide-stabilized trimethylammonium ylide with benzaldehyde 36
3.6 Reaction of sulfonium ylide 6a and benzaldehyde 39
4. pKa Values of Ammonium Salts 43
5. Copies of HPLC Chromatograms 45
6. Copies of NMR-Spectra 72

1. General Information:

1.2. General Methods

^{1}H- and ^{13}C-NMR spectra were recorded on a Bruker Avance III 300 MHz spectrometer with a broad band observe probe and a sample changer for 16 samples and on a Bruker Avance III 700 MHz spectrometer with with an Ascend magnet and TCI cryoprobe, which are both property to the Austro-Czech NMR-Research Center “RERI-uasb”. All NMR spectra were referenced on the solvent peak.

High resolution mass spectra were obtained using an Agilent 6520 Q-TOF mass spectrometer with an ESI source and an Agilent G1607A coaxial sprayer or a Thermo Fisher Scientific LTQ Orbitrap XL with an Ion Max API Source. Analyses were made in the positive ionization mode if not otherwise stated. Purine (exact mass for $[M+H]^+$ = 121.050873) and 1,2,3,4,5,6-hexakis(2,2,3,3-tetrafluoropropoxy)-1,3,5,2,4,6-triazatriphosphinane (exact mass for $[M+H]^+$ = 922.009798) were used for internal mass calibration.

IR spectra were recorded on a Shimadzu IR Affinity-1 fourier transform infrared spectrometer or a Bruker Tensor 27 FT-IR spectrometer with ATR unit.

Preparative column chromatography was carried out using Davisil LC 60A 70-200 MICRON silica gel. TLC probes were detected at 254 nm or stained with with an appropriate staining solution (compare section 3.1.3).

HPLC was performed using a Dionex Summit HPLC system consisting of a Dionex P-680 pump, an ASI-100 HPLC-autosampler, a STH-585 column oven and a PDA-100 detector or a Thermo Scientific Dionex Ultimate 3000 system with diode array detector with a Chiralcel OD-H (250 x 4.6 mm, 5 µm), a Chiralcel OD-R (250 x 4.6 mm, 10 µm), a Chiralcel OJ-H (250 x 4.6 mm, 5 µm) or a Chiralpak AD-H (250 x 4.6 mm, 5 µm) chiral stationary phase.

Optical rotations were recorded on a Perkin Elmer Polarimeter Model 241 MC (1 dm cuvette) and on a Schmidt + Haensch Polarimeter Model UniPol L 1000 (1 dm cuvette).

Melting points were measured on a Kofler melting point microscope (Reichert, Vienna).

Single-crystal structure analyses were carried out on a Bruker Smart X2S diffractometer operating with Mo-K_α radiation (λ= 0.71073 Å).

All chemicals were purchased from commercial suppliers and used without further purification unless otherwise stated.

1.2. Computational Methods

Geometry optimization has been performed using the Jaguar 8.0 pseudospectral program package using the well-established B3LYP hybrid density functional with the D3 dispersion correction and the standard split valence polarized 6-31G* basis as implemented in Jaguar.

All the optimization calculations include an implicit description of dichloromethane solvent using the Poisson–Boltzmann polarizable continuum method as incorporated in Jaguar, and parameters for dichloromethane.

Electronic energies were obtained by single point calculations at the B3LYP-D3/6-311+G**(dichloromethane) level of theory.

The correct nature of each stationary point (minima or transition state) has been checked by performing frequency calculations at the B3LYP/6-31G*(dichloromethane) level of theory (no/one imaginary frequency for minima/transition state).

Thermal and entropic contributions to free energy (at 298.15 K) and zero-point energy have been obtained from these frequency calculations. In Jaguar, the translational partition function is computed for ideal gas standard conditions, corresponding to a pressure of 1 atmosphere at 298.15 K. For solution reactions, the standard condition is instead 1 mol/L. Accordingly, the free energy value computed in Jaguar was corrected by a concentration term, equal to RT ln (V_mol_gas_1atm / V_mol_1M), i.e. 1.89 kcal/mol at 298.15 K.

For the large reaction systems there are usually several local minima or saddle points corresponding to each intermediate or transition state. This is due to the possibility of multiple conformations of substituents. We have made a systematic attempt to locate all possible local minima and saddle points, with the data presented referring to the lowest energy form unless mentioned otherwise. All species have been fully geometry optimized, and the Cartesian coordinates are supplied in section 3.

2. Syntheses

2.1 Reactions of Ester-Stabilised Ylides

2.1.1 Synthesis of Starting Onium Salts

<u>Ammonium salt **4b**:</u> In analogy to literature,[1] to a solution of ethyl bromoacetate (1 equiv.) in THF (50 ml/g) were added 1.5 equiv. of trimethylamine (33 wt% in EtOH). The solution was stirred at RT for 24 hours, during which the product precipitated as a white solid. The resulting suspension was cooled to 0 °C, filtrated, washed with ice-cold Et_2O and dried *in vacuo* to yield ammonium salt **4b** in 98% (10 mmol scale) as a white solid. ^{1}H NMR (300 MHz, δ, $CDCl_3$, 298 K): 1.31 (t, *J* = 7.1 Hz, 3H), 3.68 (s, 9H), 4.26 (q, *J* = 7.1 Hz, 2H), 5.08 (s, 2H) ppm; ^{13}C NMR (75 MHz, δ, $CDCl_3$, 298 K): 13.9, 54.3, 62.9, 63.0, 164.7 ppm.

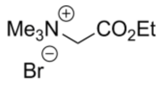

4b

<u>Sulfonium salt **13**:</u> According to literature,[2] to a solution of ethyl bromoacetate (0.9 equiv.) in THF (2.8 M) was added 1 equiv. of tetrahydrothiophene. The solution was stirred at RT for 24 hours, during which the product precipitated as colourless needles. The resulting suspension was cooled to 0 °C, filtrated, washed with ice-cold Et_2O and dried *in vacuo* to yield sulfonium salt **13** in 96% (5.5 mmol scale) as colourless crystals. Analytical data match those reported previously. ^{1}H NMR (300 MHz, δ, $CDCl_3$, 298 K): 1.32 (t, *J* = 7.1 Hz, 3H), 2.45-2.53 (m, 4H), 3.80-3.91 (m, 2H), 3.99-4.10 (m, 2H), 4.28 (q, *J* = 7.1 Hz, 2H), 5.05 (s, 2H) ppm.

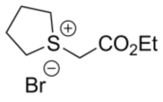

13

2.1.2 Synthesis of Starting Imines

<u>Imine **9a**:</u> According to literature,[3] benzaldehyde (2 equiv.), *tert*-butyl carbamate (1 equiv.), sodium benzenesulfinate (2.5 equiv.) and formic acid (2 equiv), were suspended in a 1:2 mixture of MeOH and water (0.3 M). The reaction mixture was stirred at RT for 24 h and the resulting precipitate was filtered and washed with Et_2O to yield sulfonyl carbamate **SI-1** in 76% (10 mmol scale) as a white solid in sufficient purity for further reactions. Analytical data match those reported previously. ^{1}H NMR (300 MHz, δ, $DMSOd_6$, 298 K): 1.18 (s, 9H), 6.02 (d, *J* = 10.8 Hz, 1H), 7.38-7.45 (m, 3H), 7.59-7.77 (m, 5H), 7.87 (d, *J* = 7.1 Hz, 2H), 8.73 (d, *J* = 10.8 Hz, 1H) ppm.

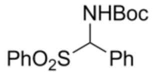

SI-1

1) Shi, L.; Han, Y.; Yang, Z.; Liu, W.; Liang, Y.; *Synthesis*, **2005**, *17*, 2851.
2) Grange, E.; Aggarwal, V. K.; *Chem. Eur. J.*, **2006**, *12*, 568.
3) Flegeau, E. F.; Bruneau, C.; Pierre, H., *J. Am. Chem. Soc.*, **2011**, *133*, 10161.

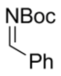

9a

According to literature,[3] **SI-1** (1 equiv.) was then dissolved in DCM (0.06 M) and 22 equiv. of K_2CO_3 (1.4 M aqueous solution) were added. The biphasic mixture was stirred vigorously at RT for 4 h. The organic layer was separated and the aqueous layer was extracted twice with DCM. The combined organics were evaporated to dryness at RT to yield **9a** in 45% (0.5 mmol scale). Analytical data match those reported previously. ^{1}H NMR (300 MHz, δ, $CDCl_3$, 298 K): 1.60 (s, 9H), 7.39-7.63 (m, 3H), 7.84-7.97 (m, 2H), 8.87 (s, 1H) ppm.

<u>Imine **9b**</u>: According to literature,[4] 4-bromobenzaldehyde (1.2 equiv.), *tert*-butyl carbamate (1 equiv.), sodium benzenesulfinate (2.5 equiv.) and formic acid (2 equiv), were suspended in a 1:2 mixture of MeOH and water (0.3 M). The reaction mixture was stirred at RT for 48 h and the resulting precipitate was filtered and washed with Et_2O to yield sulfonyl carbamate **SI-2** in 45% (5 mmol scale) as white solid in sufficient purity for further reactions. Analytical data match those reported previously. ^{1}H NMR (300 MHz, δ, $CDCl_3$, 298 K): 1.22 (s, 9H), 5.66 (d, *J* = 10.8 Hz, 1H), 5.86 (d, *J* = 10.8 Hz, 1H), 7.31 (d, *J* = 7.6 Hz, 2H), 7.50-7.70 (m, 5H), 7.89 (d, *J* = 7.6 Hz, 2H) ppm.

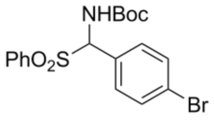

SI-2

According to literature,[4] anhydrous K_2CO_3 (6 equiv.) and Na_2CO_3 (1 g/mmol **SI-2**) were flame-dried in a 3-neck flask. After cooling, **SI-2** (1 equiv.) was added and dissolved in anhydrous THF (0.1 M). The mixture was refluxed for 15 h and afterwards the solids were removed by suction filtration. The filtrate was evaporated to dryness at RT to yield **9b** in 80% (1.5 mmol scale). Analytical data match those reported previously. ^{1}H NMR (300 MHz, δ, $CDCl_3$, 298 K): 1.59 (s, 9H), 7.59 (d, *J* = 8.3 Hz, 2H), 7.75 (d, *J* = 8.3 Hz, 2H), 8.79 (s, 1H) ppm.

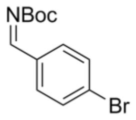

9b

<u>Imine **9c**</u>: According to literature,[4] 4-methoxybenzaldehyde (1.2 equiv.), *tert*-butyl carbamate (1 equiv.), sodium benzenesulfinate (1.3 equiv.) and formic acid (2 equiv.), were suspended in a 1:9 mixture of MeOH and water (1 M). The reaction mixture was stirred at RT for 9 h and the resulting precipitate was filtered and washed with Et_2O to yield sulfonyl carbamate **SI-3** in 60% (5 mmol scale) as white solid in sufficient purity for further reactions. Analytical data match those reported previously. ^{1}H NMR (300 MHz, δ, $CDCl_3$, 298 K): 1.22 (s, 9H), 2.38 (s, 3H), 3.78 (s, 3H), 5.77 (d, *J* = 11.0 Hz, 1H), 5.82 (d, *J* = 11.0 Hz, 1H), 6.90 (d, *J* = 8.8 Hz, 1H), 7.29 (d, *J* = 7.9 Hz, 2H), 7.34 (d, *J* = 8.8 Hz, 2H), 7.76 (d, *J* = 7.9 Hz, 2H) ppm.

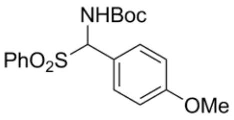

SI-3

According to literature,[4] anhydrous K_2CO_3 (6 equiv.) and Na_2CO_3 (1 g/mmol **SI-3**) were flame-dried in a 3-neck flask. After cooling, **SI-3** (1 equiv.) was added and dissolved in anhydrous THF (0.1 M). The mixture was refluxed for 24 h and afterwards the solids were removed by suction filtration. The filtrate was evaporated to dryness at RT to yield **9b** in 88% (1.3 mmol scale). Analytical data match those reported previously. ^{1}H NMR (300 MHz, δ, $CDCl_3$, 298 K): 1.59 (s, 9H), 3.86 (s, 3H), 6.94 (d, *J* = 8.8 Hz, 2H), 7.86 (d, *J* = 8.8 Hz, 2H), 8.87 (s, 1H) ppm.

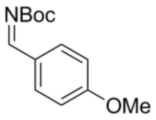

9c

---

4) Wenzel, A. G.; Jacobsen, E. N., *J. Am. Chem. Soc.*, **2002**, *124*, 12964.

Imine **9d**: According to literature,[5] benzaldehyde (1 equiv.), and *p*-toluenesulfonamide (1 equiv.) were dissolved in toluene (0.37 M). The reaction flask was fitted with a Dean-Stark condenser and the mixture was refluxed for 24 h. The solution was then evaporated to dryness to afford a white solid, which was recrystallized from Et_2O and dried *in vacuo* to yield **9d** in 80% (15 mmol scale) as a white solid. Analytical data match those reported previously. ^{1}H NMR (300 MHz, δ, $CDCl_3$, 298 K): 2.44 (s, 3H), 7.35 (d, *J* = 7.9 Hz, 2H), 7.49 (d, *J* = 7.2 Hz, 2H), 7.60 (d, *J* = 7.3 Hz, 1H), 7.87-7.96 (m, 4H), 9.03 (s, 1H) ppm.

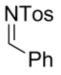

9d

2.1.3 Reactions of Ester-Stabilised Onium Salts with Electrophiles

General procedure: 2 Equiv. of electrophile were dissolved in anhydrous DCM (0.08 M) and the onium salt (1 equiv.) and Cs_2CO_3 (20 equiv.) were added. The mixture was stirred for 24 h at the stated temperature and then brine was added, the phases separated, and the aqueous phase was extracted three times with DCM. The combined organic phases were dried over Na_2SO_4 and evaporated to dryness. The resulting crude product was subjected to column chromatography as stated below.

Reaction of **4b** with imine **9a**: The mixture was stirred at RT for 24 h (0.2 mmol scale) and the crude product separated by column chromatography (silica gel, heptanes:EtOAc = 50:1) to yield a mixture of **10a** (15%) **11a (**10%) and **12a** (30%). Analytical data of **10a** and **12a** match those reported previously.[6,7] ^{1}H NMR (300 MHz, δ, $CDCl_3$, 298 K): ***10a:*** 1.25 (t, *J* = 7.1 Hz, 3H), 1.39 (s, 9H), 3.02 (d, *J* = 2.6 Hz, 1H), 3.74 (d, *J* = 2.6 Hz, 1H), 4.09-4.31 (m, 2H), 7.20-7.31 (m, 5H); ***11a:*** 1.24 (t, *J* = 7.2 Hz, 3H), 1.29 (s, 9H), 4.14 (q, *J* = 7.2 Hz, 2H), 5.08 (s, 1H), 7.25-7.35 (m, 5H); ***12a:*** 1.29 (t, *J* = 7.1 Hz, 3H), 4.23 (q, *J* = 7.1 Hz, 2H), 7.24-7.42 (m, 3H), 7.49-7.55 (m, 2H) ppm; ^{13}C NMR (75 MHz, δ, $CDCl_3$, 298 K): ***11a:*** 14.3, 27.9 (3C), 60.0, 81.2, 99.0, 127.2 (2C), 127.9 (2C), 129.5, 136.3, 151.6, 155.3, 168.6 ppm.

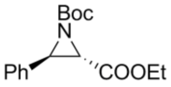

10a

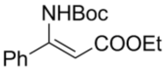

11a

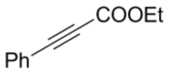

12a

Reaction of **4b** with imine **9b**: The mixture was stirred at RT for 24 h (0.2 mmol scale), yielding a mixture of **10b** (5%) **11b** (20%) and **12b** (25%) (determined by ^{1}H NMR of the crude product, rapid decomposition during silica gel column chromatography).

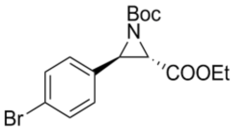

10b

5) Durán-Galván, M.; Connell, B. T., *Tetrahedron*, **2011**, *67*, 7901.
6) Zhao, W.; Lu, Z.; Wul, W. D., *J. Org. Chem.*, **2014**, *79*, 10068.
7) Castagnolo, D.; Botta, M., *Eur. J. Org. Chem.*, **2010**, *17*, 3224.

<u>Reaction of **4b** with imine **9c**</u>: The mixture was stirred at RT for 24 h (0.2 mmol scale) yielding **10c** in 50% crude yield (determined by ^{1}H NMR of the crude product, rapid decomposition during silica gel column chromatography).

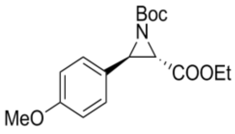

10c

<u>Reaction of **4b** with imine **9d**</u>: The mixture was refluxed for 24 h (0.2 mmol scale) and the crude product purified by column chromatography (silica gel, heptanes:EtOAc = 5:1) to yield **10d** in 49% (> 90% cis). Analytical data of **10d** match those reported previously.[8] ^{1}H NMR (300 MHz, δ, $CDCl_3$, 298 K): *cis isomer:* 0.99 (t, *J* = 7.0 Hz, 3H), 2.46 (s, 3H), 3.71 (d, *J* = 7.6 Hz, 1H), 3.87-4.05 (m, 2H), 4.14 (d, *J* = 7.6 Hz, 1H), 7.25-7.35 (m, 5H) 7.38 (d, *J* = 8.1 Hz, 2H), 7.95 (d, *J* = 8.1 Hz, 2H) *trans isomer:* 1.39 (t, *J* = 7.2 Hz, 3H), 2.46 (s, 3H), 4.48 (d, *J* = 4.2 Hz, 1H), 4.28-4.33 (m, 2H), 4.54 (d, *J* = 4.2 Hz, 1H), 7.25-7.39 (m, 7H) 7.81 (d, *J* = 8.1 Hz, 2H) ppm.

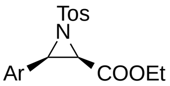

10d

<u>Reaction of **4b** with benzaldehyde:</u> The reaction mixture was stirred at RT for 24 h (0.2 mmol scale) and the crude product purified by column chromatography (silica gel, heptanes:EtOAc = 7:3) to yield **5b** in 12%. Analytical data match those reported previously.[9] ^{1}H NMR (300 MHz, δ, $CDCl_3$, 298 K): 1.33 (t, *J* = 7.2 Hz, 3H), 3.51 (t, *J* = 1.7 Hz, 1H), 4.10 (d, *J* = 1.7 Hz, 1H), 4.24-4.26 (m, 2H), 7.87-7.96 (m, 4H), 7.27-7.41 (m, 5H) ppm.

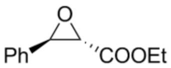

5b

<u>Reaction of **13** with benzaldehyde:</u> The mixture was stirred at RT for 24 h (0.2 mmol scale) and the crude product purified by column chromatography (silica gel, heptanes:EtOAc = 7:3) to yield **5b** in 37%. Analytical data match those reported previously.[9] ^{1}H NMR (300 MHz, δ, $CDCl_3$, 298 K): 1.33 (t, *J* = 7.2 Hz, 3H), 3.51 (t, *J* = 1.7 Hz, 1H), 4.10 (d, *J* = 1.7 Hz, 1H), 4.24-4.26 (m, 2H), 7.87-7.96 (m, 4H), 7.27-7.41 (m, 5H) ppm.

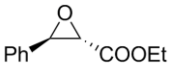

5b

8) Vyas, R.; Gao, G.-Y.; Harden, J. D.; Zhang, X. P., *Org. Lett.*, **2004**, *6*, 1907.
9) Kang, B.; Kim, M.; Lee, J.; Do, Y.; Chang, S., *J. Org. Chem.*, **2006**, *71*, 6721.

2.2 Reactions of Amide-Stabilised Ylides

2.2.1 Synthesis of Chiral Amines A-C

Amine A: According to literature,[10] (1*S*,2*S*)-1,2-Diphenylethylenediamine (1 equiv.) was dissolved in DCM (0.25 M) and 4-DMAP (4-Dimethylaminopyrridine, 4 mol%) and triethylamine (4.9 equiv.) were added. The mixture was cooled to 5 °C and chloroacetyl chloride (3 equiv.) was added dropwise. Afterwards the solution was allowed to warm to RT and stirred for 3 hours. The mixture was then cooled to 0 °C and filtered by suction filtration. The precipitate was washed with ice-cold water an dried *in vacuo* to yield **SI-4** in 89% (2.5 mmol scale) as a brown solid. Analytical data match those reported previously. ^{1}H NMR (300 MHz, δ, $CDCl_3$, 298 K): 4.04 (d, *J* = 15.1 Hz, 2H), 4.11 (d, *J* = 15.1 Hz, 2H), 5.27-5.38 (m, 2H), 7.10-7.17 (m, 4H), 7.21-7.27 (m, 6H) ppm.

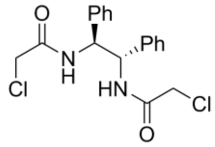
SI-4

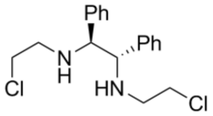
SI-5

According to literature,[10] **SI-4** (1 equiv.) was dissolved in THF (0.11 M) and a 1 M solution of BH_3 in THF (4.2 equiv.) was added slowly. The reaction mixture was refluxed for 2 hours, cooled to 0 °C and MeOH was added to quench excess BH_3. The mixture was evaporated to dryness and the residue was redissolved in 5 wt% aqueous HCl (10 ml/mmol) and extracted with 5 ml/mmol DCM. The organic phase was discarded and the aqueous phase was basified by addition of 15 wt% of aqueous NaOH and extracted with DCM. The combined organic phases were dried over Na_2SO_4 and evaporated to dryness to yield **SI-5** in 52% (2.2 mmol scale) as yellow foam and used in the next step without further purification.

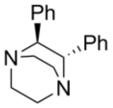
A

According to literature,[10] **SI-5** (1 equiv.) was dissolved in DMF (0.3 M) and refluxed for 4 hours. The reaction mixture was concentrated under reduced pressure and the residue was dissolved in water, basified with 15 wt% aqueous NaOH and extracted with DCM. The combined organic phases were dried over Na_2SO_4 and evaporated to dryness to yield **A** in 77% (0.28 mmol scale) as a brown oil. Analytical data match those reported previously. ^{1}H NMR (300 MHz, δ, $CDCl_3$, 298 K): 2.56-2.68 (m, 2H), 2.73-2.87 (m, 2H), 2.97-3.05 (m, 4H), 4.18 (s, 2H), 7.31-7.52 (m, 10H) ppm.

Amine B: According to literature,[11] L-proline (1 equiv.) was suspended in MeOH (1 M) and 2 equiv. thionylchloride were added at -10 °C over 1 hour. The resulting solution was stirred at RT for 2 hours, refluxed for 1.5 hours and evaporated to dryness. The resulting residue was re-dissolved in the same amount of MeOH and concentrated under reduced pressure. This procedure was repeated three times to remove excess thionylchloride and yield a yellow wax. This residue was dissolved in DCM (1 M) and 6 equiv. $NaHCO_3$ were added and the mixture

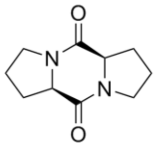
SI-6

10) Oi, R.; Sharpless, B. K., *Tetrahedron Lett.*, **1991**, *32*, 4853.
11) Nakamura, D.; Kakiuchi, K.; Koga, K.; Shirai, R., *Org. Lett,* **2006,** *8*, 6139.

stirred vigorously for 30 minutes. The remaining residue was removed by filtration, washed thoroughly with DCM and the filtrate was evaporated to dryness. The residue was stirred at RT for 4 days and then dissolved in DCM, washed with brine and dried *in vacuo*, yielding **SI-6** in 43% (7.6 mmol scale) as white crystals in sufficient purity for further reactions. Analytical data match those reported previously. ^{1}H NMR (300 MHz, δ, $CDCl_3$, 298 K): 1.85-2.39 (m, 8H), 3.50-3.58 (m, 4H), 4.17 (t, *J* = 8.1 Hz, 2H) ppm.

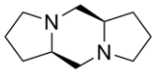

B

According to literature,[11] $LiAlH_4$ (1 equiv.) was suspended in THF (4 M) and a solution of **SI-6** (0.4 M) in THF was added dropwise over 15 minutes. The mixture was refluxed for 5 hours, cooled to 0 °C and then the reaction was quenched by addition of H_2O, extracted with Et_2O, and evaporated to dryness. The residue was purified by distillation in a bulb-to-bulb apparatus (150 °C/14 mbar) to yield **B** in 69% (4.2 mmol scale) as white crystals. ^{1}H NMR data were consistent with literature. ^{1}H NMR (300 MHz, δ, $CDCl_3$, 298 K): 1.35-1.57 (m, 6H), 1.59-1.76 (m, 2H), 2.23-2.37 (m, 4H), 2.39-2.49 (m, 4H), 2.68 (ddd, *J* = 10.4, 8.8, 1.6 Hz, 2H) ppm.

<ins>Amine **C**:</ins> According to literature,[12] (1*R*,2*R*)-(+)-1,2-diaminocyclohexane L-tartrate (1 equiv.) was dissolved in 0.4 ml/mmol of 85 wt% formic acid and a solution of 37% aqueous formaldehyde (6.5 equiv.) was added over 15 minutes at RT. The reaction mixture was refluxed for 2 hours and then cooled to 0 °C. Solid NaOH was added until the reaction mixture reached a pH of 13. The basic mixture was extracted with Et_2O and the combined organic phases were dried over Na_2SO_4 and evaporated to dryness, yielding **B** in 80% (7.6 mmol scale) as an orange liquid in sufficient purity for further reactions. Analytical data match those reported previously.[13] ^{1}H NMR (300 MHz, δ, $CDCl_3$, 298 K): 0.93-1.20 (m, 4H), 1.55-1.71 (m, 2H), 1.71-1.83 (m, 2H), 2.21 (s, 12H).

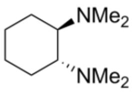

C

2.2.2 Synthesis of Bicyclic Chiral Amines D Based on L-Proline

Protection of L-Proline:

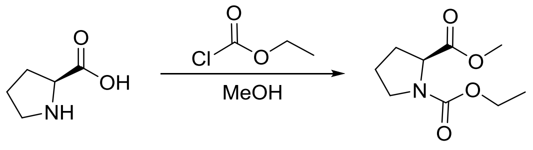

SI-7 **SI-8**

According to literature,[14] L-proline **SI-7** (5.00 g, 43.4 mmol) was dissolved in 100 mL MeOH followed by the addition of K_2CO_3 (6.01 g, 43.5 mmol). The mixture was cooled to 0 °C and ethyl

---

12) Cabello, N.; Kizirian, J.-C.; Gille, S.; Alexakis, A.; Bernardinelli, G.; Pinchard, L.; Caille, J.-C., *Eur. J. Org. Chem.*, **2005**, 52, 4835.
13) Remenar, J. F.; Lucht, B. L., Collum, D. B., *J. Am. Chem. Soc.*, **1997**, *119*, 5567.

chloroformate (9.11 mL, 10.34 g, 95.3 mmol) was added dropwise over 30 minutes. The mixture was stirred for 4 hours at 0 °C and 12 hours at RT. Methanol was removed under reduced pressure and 40 mL water were added to the residual oil. This solution was extracted with DCM and the combined organic phases were washed with brine. The organic phases were dried with Na_2SO_4 and evaporated to dryness to yield the product **SI-8** as a pale yellow oil (8.53 g, 42.4 mmol, 97%). Analytical data were consistent with literature.[15] ^{1}H NMR (300 MHz, δ, $CDCl_3$, 298 K, mixture of rotamers): 1.16 (t, 3H, *J* = 7.1 Hz), 1.23 (t, 3H, J = 7.1 Hz), 1.79-2.04 (m, 5H), 2.04-2.33 (m, 3H), 3.35-3.61 (m, 4H), 3.69 (s, 3H), 3.70 (s, 3H), 4.00-4.19 (m, 4H), 4.25-4.32 (m, 1H), 4.32-4.40 (m, 1H) ppm; ^{13}C NMR (75 MHz, δ, CDCl3, 298 K, mixture of rotamers): 14.6, 14.7, 13.5, 24.3, 29.9, 30.9, 46.3, 46.7, 52.1, 52.2, 58.8, 59.0, 61.3, 61.4, 154.7, 155.2, 173.3, 173.4 ppm.

General Procedure Step A (Grignard Reaction):

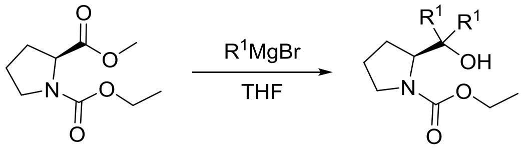

SI-8

According to literature,[15] Mg-turnings (4 eq.) were suspended in THF (3 mL/10 mmol Mg) and 4 eq. of the corresponding arylbromide (dissolved in 7 mL THF per 10 mmol arylbromide) were added dropwise over 30 minutes. The reaction mixture was stirred for 30 minutes and refluxed for 1 hour to obtain the Grignard reagent. The reaction mixture was cooled to 0 °C and 1 eq. of **SI-8** was added as a solution in THF (15 mL/10 mmol) over 30 minutes. The reaction mixture was stirred for 1 hour at 0 °C and 4 hours at room temperature. Subsequently the reaction mixture was cooled to 0 °C and quenched by adding water. The mixture was filtrated by suction filtration and washed with diethylether. The filtrate was extracted with diethylether and the combined organic phases were dried over Na_2SO_4 and evaporated to dryness. Purification by column chromatography (gradient of heptanes and EtOAc) gave the corresponding Grignard-Products.

General Procedure Step B (Deprotection):

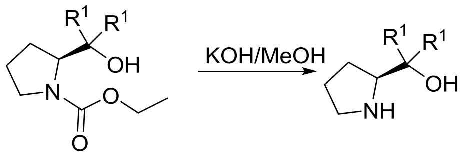

According to literature,[18] 10 eq.of KOH were dissolved in methanol (0.5 mL/mmol KOH), 1 eq. of the protected prolinol-amine was added and the mixture was refluxed overnight. Subsequently, the reaction mixture was concentrated *in vacuo*, water was added and the mixture was extracted with DCM. The combined organic products were dried over Na_2SO_4 and concentrated *in vacuo* to yield the corresponding compounds.

---

14) Bhaskar, J.V.; Periasamy, M.; *Tetrahedron*, **1993**, *49*, 5127–5132
15) Gautier, F.-M., Jones, S., and Martin, S. J.; *Org. Biomol. Chem.*, **2009**, *7*, 229–231.

General Procedure Step C (Ring Closure):

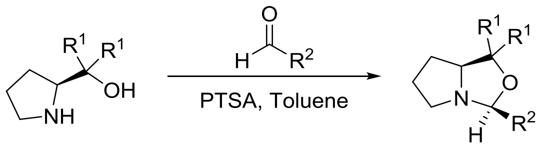

In accordance to literature,[16] 1 eq. of unprotected amine was dissolved in toluene (20 mL/mmol) followed by the addition of 0.125 eq. PTSA and 1.3 eq. aldehyde. The reaction mixture was refluxed overnight using a Dean-Stark apparatus. Subsequently the reaction mixture was cooled to RT and the reaction mixture was washed with saturated aqueous sodium bicarbonate solution, saturated sodium bisulfite solution, water and brine. The organic phase was dried over Na_2SO_4 and evaporated to dryness to yield the corresponding products.

Please note: *This step was the limiting step with respect to the use of aliphatic groups R1 and aromatic groups R2. In neither of the two cases the cyclization proceeded satisfactory and thus this procedure is limited to aromatic groups R1 and aliphatic groups R2!*

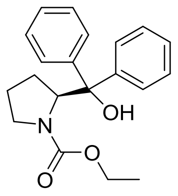

SI-9

Amine **D1**: Grignard-Product **SI-9** was obtained in 76% yield (11.29 mmol scale) as a white solid according to general procedure A. Analytical data were in accordance to literature,[15] $[\alpha]_D^{20}$ = -100.2 (c = 0.998, DCM); IR (film): $\tilde{\nu}$ = 3401, 3057, 2980, 2360, 1665, 1446, 1421, 1380, 1336, 1196, 1126, 1034, 875, 764, 701 cm^{-1}; ^{1}H NMR (300 MHz, δ, $CDCl_3$, 298 K): 0.82 (bs, 1H), 1.25 (t, *J* = 7.0 Hz, 3H), 1.46-1.56 (m, 1H), 1.93-2.00 (m, 1H), 2.08-2.16 (m, 1H), 2.98 (bs, 1H), 3.43 (m, 1H), 4.03-4.22 (m, 2H), 4.95 (dd, 1H, *J* = 3.6, 8.8 Hz), 7.27-7.35 (m, 10H) ppm; ^{13}C NMR (75 MHz, δ, $CDCl_3$, 298 K): 14.7, 22.9, 29.7, 47.7, 61.9, 65.9, 81.6, 127.1, 127.2, 127.4, 127.6, 127.9, 128.2, 143.7, 146.4 ppm; HRMS (ESI): *m/z* calcd. for $C_{20}H_{23}NO_3$: 348.1570 $[M+Na]^+$; found: 348.1570.

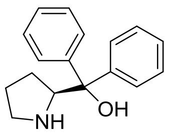

SI-10

Unprotected amine **SI-10** was obtained in 87% yield (1.85 mmol scale) as a yellow oil according to general procedure B. Analytical data were in accordance to literature,[15] $[\alpha]_D^{20}$ = -84.9 (c = 0.989, DCM); IR (film): $\tilde{\nu}$ = 3348, 3056, 2967, 2870, 1597, 1492, 1448, 1397, 1283, 1172, 1103, 1068, 1032, 991, 951, 906, 747, 697, 636 cm^{-1}; ^{1}H NMR (300 MHz, δ, $CDCl_3$, 298 K): 1.52-1.84 (m, 4H), 2.89-3.12 (m, 2H), 4.29 (t, 1H, *J* = 7.5 Hz), 7.20-7.29 (m, 2H), 7.32-7.43 (m, 4H), 7.59-7.65 (m, 2H), 7.66-7.71 (m, 2H) ppm; ^{13}C NMR (75 MHz, δ, $CDCl_3$, 298 K): 25.5, 26.3, 46.8, 64.5, 77.2, 125.5, 125.9, 126.4, 126.5, 128.0, 128.2, 145.4, 148.1 ppm; HRMS (ESI): *m/z* calcd. for $C_{17}H_{19}NO$: 254.1539 $[M+H]^+$; found: 254.1543.

---

16) Zuo, G.; Zhang, Q.; Xu, J., *Heteroatom Chem.*, **2003**, *14*, 42–45

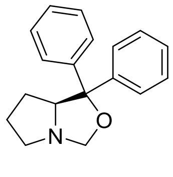

D1

Amine **D1** was obtained in 60% yield (0.34 mmol scale) as a brown oil according to general procedure C. Analytical data were in accordance to literature,[16] $[\alpha]_D^{20}$ = -338.1 (c = 1.080, DCM); IR (film): $\tilde{\nu}$ = 3058, 3026, 2960, 2940, 2868, 1491, 1447, 1152, 1009, 967, 751, 699, 662 cm^{-1}; ^1H NMR (300 MHz, δ, $CDCl_3$, 298 K): 1.03-1.21 (m, 1H), 1.53-1.68 (m, 3H), 2.66-2.81 (m, 1H), 3.15-3.26 (m, 1H), 4.21 (d, J = 6.2 Hz, 1H), 4.35 (t, *J* = 7.0 Hz, 1H), 4.44 (d, *J* = 6.2 Hz, 1H), 7.06-7.33 (m, 8H), 7.40-7.47 (m, 2H) ppm; ^{13}C NMR (75 MHz, δ, $CDCl_3$, 298 K): 26.5, 30.6, 56.7, 70.8, 85.7, 88.1, 125.9, 126.5, 127.1, 127.3, 127.9, 128.2, 143.9, 144.5 ppm; HRMS (ESI): *m/z* calcd. for $C_{18}H_{19}NO$: 266.1539 $[M+H]^+$; found: 266.1543.

Amine **D2**: Grignard-product **SI-11** was obtained in 27% unoptimized yield (4.97 mmol scale) as a white solid according to general procedure A. Analytical data were in accordance to literature,[15] ^1H NMR (300 MHz, δ, $CDCl_3$, 298 K): 0.83 (bs, 1H), 1.19-1.31 (m, 3H), 1.45-1.59 (m, 1H), 2.10-2.32 (m, 2H), 3.02 (bs, 1H), 3.41-3.53 (m, 1H), 4.07-4.27 (m, 2H), 5.20 (dd, 1H, *J* = 3.9, 8.7 Hz), 7.46-7.63 (m, 6H), 7.77-7.96 (m, 8H) ppm; ^{13}C NMR (75 MHz, δ, $CDCl_3$, 298 K): 14.6, 23.1, 29.8, 47.8, 62.1, 66.2, 81.9, 125.2, 125.9, 126.0, 126.0, 126.1, 126.4, 127.0, 127.1, 127.3, 127.5, 127.5, 127.9, 128.4, 128.4, 132.6, 132.6, 132.8, 141.1, 143.6 ppm.

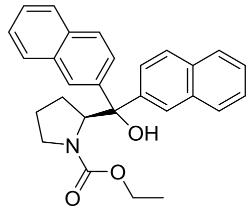

SI-11

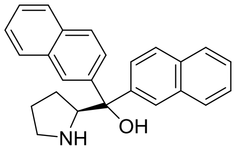

SI-12

Unprotected amine **SI-12** was obtained in 81% yield (1.35 mmol scale) as a white solid according to general procedure B. Analytical data were in accordance to literature.[17] $[\alpha]_D^{20}$ = -92.1 (c = 0.496, MeOH); IR (film): $\tilde{\nu}$ = 3349, 3055, 2967, 2870, 1630, 1599, 1505, 1399, 1270, 1240, 1122, 1101, 859, 820, 788, 747 cm^{-1}; ^1H NMR (300 MHz, δ, $CDCl_3$, 298 K): 1.57-1.89 (m, 5H), 2.98-3.17 (m, 2H), 4.56 (t, 1H, *J* = 7.4 Hz), 7.42-7.67 (m, 5H), 7.69-7.84 (m, 5H), 7.86-7.95 (m, 2H), 8.11-8.17 (m, 2H) ppm; ^{13}C NMR (75 MHz, δ, $CDCl_3$, 298 K): 25.6, 26.5, 46.8, 64.0, 77.3, 123.8, 124.0, 124.4, 125.3, 125.6, 125.8, 125.9, 126.1, 127.5, 127.7, 128.1, 128.2, 128.3, 132.2, 132.3, 133.1, 133.2, 142.6, 145.3 ppm; HRMS (ESI): *m/z* calcd. for $C_{25}H_{23}NO$: 354.1852 $[M+H]^+$; found: 354.1852.

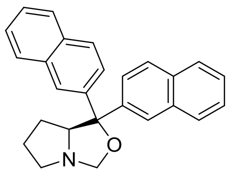

D2

Amine **D2** was obtained in 55% yield (0.51 mmol scale) as a beige solid according to general procedure C. $[\alpha]_D^{20}$ = -320.4 (c = 0.594, DCM); IR (film): $\tilde{\nu}$ = 3054, 2940, 2869, 1505, 1599, 1354, 1272, 1190, 1153, 1123, 1024, 966, 896, 815, 746 cm^{-1}; ^1H NMR (300 MHz, δ, $CDCl_3$, 298 K): 1.12-1.33 (m, 1H), 1.53-1.68 (m, 3H), 1.66-1.82 (m, 1H), 2.81-2.96 (m, 1H), 3.36-3.47 (m, 1H) 4.37 (d, *J* = 6.5 Hz, 1H), 4.61 (d, *J* = 6.5 Hz, 1H), 4.81 (t, *J* = 7.0 Hz, 1H), 7.23-7.35 (m, 2H), 7.41-7.57 (m, 4H), 7.68-7.83 (m, 4H), 7.83-7.95 (m, 2H), 8.08-8.17 (m, 2H) ppm; ^{13}C NMR (75 MHz, δ, $CDCl_3$, 298 K): 26.7, 30.8, 56.9, 69.7, 85.8, 88.6, 124.0, 124.8, 125.7, 125.8, 126.2,

17) Mathre, D.J.; Jones, T.K.; Xavier, L.C.; Blacklock, T.J.; Reamer, R.A.; Mohan, J.J.; Jones, E.T.T.; Hoogsteen, K.; Baum, M.W.; Grabowski, E.J.J., *J. Org. Chem.*, **1991**, *56*, 751–762.

127.0, 127.5, 127.6, 127.7, 128.2, 128.4, 128.6, 128.7, 132.2, 132.7, 132.8, 133.0, 140.9, 141.1 ppm; HRMS (ESI): *m/z* calcd. for $C_{26}H_{23}NO$: 366.1852 $[M+H]^+$; found: 366.1853.

<u>Amine **D3**:</u> Grignard-product **SI-13** was obtained in 90% yield (9.94 mmol scale) as a pale-yellow solid according to general procedure A. ^{1}H NMR (300 MHz, δ, $CDCl_3$, 298 K): 0.86 (bs, 1H), 1.26 (t, 3H, *J* = 7.3 Hz), 1.42-1.58 (m, 1H), 1.88-1.98 (m, 1H), 2.02-2.17 (m, 1H), 2.98 (bs, 1H), 3.33-3.51 (m, 1H), 3.81 (s, 6H), 4.02-4.24 (m, 2H), 4.90 (dd, 1H, J = 3.4, 8.8 Hz), 6.81-7.01 (m, 4H), 7.26-7.38 (m, 4H) ppm.

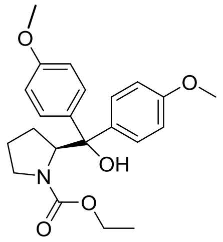

SI-13

Unprotected amine **SI-14** was obtained in 85% yield (1.57 mmol scale) as a yellow solid according to general procedure B. Analytical data were in accordance to literature.[17] $[\alpha]_D^{20}$ = -83.0 (c = 1.106, DCM); IR (film): $\tilde{\nu}$ = 3349; 3035; 2952, 2907; 2870; 2834; 1606; 1582; 1506; 1461; 1298; 1243; 1172; 1095; 1033; 991; 826; 635; 602; 582 cm^{-1}; ^{1}H NMR (300 MHz, δ, $CDCl_3$, 298 K): 1.49-1.85 (m, 4H), 2.92-3.10 (m, 2H), 3.80 (s, 6H), 4.21 (t, 1H, *J* = 7.4 Hz), 6.80-6.89 (m, 4H), 7.37-7.44 (m, 2H), 7.45-7.52 (m, 2H) ppm; ^{13}C NMR (75 MHz, δ, $CDCl_3$, 298 K): 25.5, 26.3, 46.8, 55.2, 55.2, 64.7, 77.3, 113.3, 113.5, 126.6, 126.9, 138.1, 140.6, 157.9, 158.1 ppm; HRMS (ESI): *m/z* calcd. for $C_{19}H_{23}NO_3$: 314.1751 $[M+H]^+$; found: 314.1754.

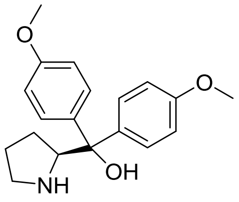

SI-14

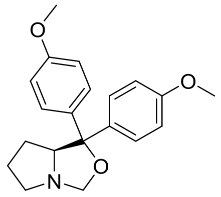

D3

Amine **D3** was obtained in 90 % yield (0.57 mmol scale) as a yellow solid according to general procedure C. $[\alpha]_D^{20}$ = -265.1 (c = 4.47, DCM); IR (film): $\tilde{\nu}$ = 2955, 2937, 2908, 2868, 2835, 2358, 1607, 1581, 1509, 1462, 1442, 1300, 1245, 1174, 1034, 1019, 1003, 968, 953, 824, 586, 564 cm^{-1}; ^{1}H NMR (300 MHz, δ, $CDCl_3$, 298 K): 1.11-1.30 (m, 1H), 1.56-1.79 (m, 3H), 2.74-2.90 (m, 1H), 3.23-3.35 (m, 1H), 3.78 (s, 6H), 4.29 (d, *J* = 6.2 Hz, 1H), 4.37 (t, *J* = 7.1 Hz, 1H), 4.49 (d, *J* = 6.2 Hz, 1H), 6.77-6.90 (m, 4H), 7.23-7.33 (m, 2H), 7.37-7.48 (m, 2H) ppm; ^{13}C NMR (75 MHz, δ, $CDCl_3$, 298 K): 26.5, 30.6, 55.1, 56.7, 70.7, 85.5, 87.7, 113.2, 113.5, 127.0, 128.4, 136.5, 136.8, 158.0, 158.7 ppm; HRMS (ESI): *m/z* calcd. for $C_{20}H_{23}NO_3$: 326.1751 $[M+H]^+$; found: 326.1754.

<u>Amine **D4**:</u> Protected amine **SI-15** was obtained in unoptimized 15% yield (2.48 mmol scale) as a yellow solid according to general procedure A. Analytical data were were in accordance to literature.[18] $[\alpha]_D^{20}$ = -81.9 (c = 0.647, DCM); IR (film): $\tilde{\nu}$ = 3416, 2982, 1666, 1414, 1325, 1165, 1122, 1070, 1017, 837 cm^{-1}; ^{1}H NMR (300 MHz, δ, $CDCl_3$, 298 K): 0.99 (bs, 1H), 1.24 (t, 3H, *J* = 7.2 Hz), 1.52-1.68 (m, 1H), 1.88-2.04 (m, 1H), 2.08-2.23 (m, 1H), 3.04 (bs, 1H), 3.45-3.58 (m, 1H), 4.03-4.26 (m, 2H), 4.94 (dd, 1H, *J* = 4.2, 8.8 Hz), 7.49-7.70 (m, 8H) ppm; ^{13}C NMR (75 MHz, δ, $CDCl_3$, 298 K): 14.5, 23.1, 29.7, 47.9, 62.3, 66.1, 81.2, 124.4 (q, *J* = 270.9 Hz), 124.6 (q, *J* = 3.6 Hz), 125.0 (q, *J* = 3.6 Hz), 127.9, 128.3, 129.5 (q, *J* = 32.2 Hz), 129.9 (q, *J* = 32.2), 147.2, 149.4, 158.6 ppm; HRMS (ESI): *m/z* calcd. for $C_{22}H_{21}F_6NO_3$: 484.1318 $[M+Na]^+$; found: 484.1309.

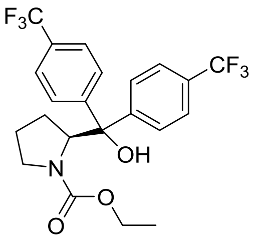

SI-15

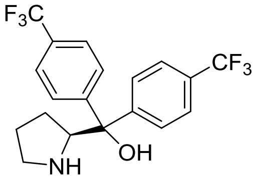

SI-16

Unprotected amine **SI-16** was obtained in 75% yield (0.24 mmol scale) as a pale-yellow solid according to general procedure B. Analytical data were were in accordance to literature.[18] $[\alpha]_D^{20}$ = -56.8 (c = 1.362, DCM); IR (film): $\tilde{\nu}$ = 3352, 2974, 2877, 1676, 1617, 1415, 1325, 1164, 1124, 1099, 1069, 1017, 831 cm^{-1}; ^{1}H NMR (300 MHz, δ, $CDCl_3$, 298 K): 1.52-1.65 (m, 2H), 1.68-1.82 (m, 2H), 2.92-3.15 (m, 2H), 4.30 (t, 1H, *J* = 7.7 Hz), 7.52-7.60 (m, 4H), 7.60-7.66 (m, 2H), 7.68-7.74 (m, 2H) ppm; ^{13}C NMR (75 MHz, δ, $CDCl_3$, 298 K): 25.5, 26.4, 46.8, 64.2, 77.2, 125.2 (q, *J* = 3.8 Hz), 125.4 (q, *J* = 3.8 Hz), 126.1 (d, *J* = 30 Hz), 149.8 (d, *J* = 213 Hz) ppm; HRMS (ESI): *m/z* calcd. for $C_{19}H_{17}F_6NO$: 390.1287 $[M+H]^+$; found: 390.1289.

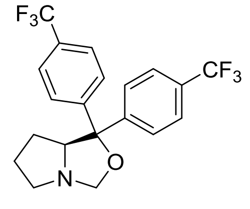

D4

Amine **D4** was obtained in 56% yield (0.62 mmol scale) as a yellow solid according to general procedure C. $[\alpha]_D^{20}$ = -80.5 (c = 0.480, DCM); ^{1}H NMR (300 MHz, δ, $CDCl_3$, 298 K): 0.99-1.24 (m, 1H), 1.60-1.76 (m, 3H), 2.70-2.84 (m, 1H), 3.25-3.41 (m, 1H), 4.24 (d, *J* = 6.4 Hz, 1H), 4.51 (t, *J* = 7.0 Hz, 1H), 4.54 (d, *J* = 6.4 Hz, 1H), 7.37-7.63 (m, 4H), 7.68-7.75 (m, 2H), 7.80-7.87 (m, 2H) ppm; ^{13}C NMR (75 MHz, δ, $CDCl_3$, 298 K): 26.4, 30.0, 53.3, 56.2, 70.5, 85.5, 125.3 (q, *J* = 3.8 Hz), 125.6 (q, *J* = 3.8 Hz), 126.1 (d, J = 30 Hz), 149.8 (d, *J* = 215 Hz) ppm; HRMS (ESI): *m/z* calcd. for $C_{20}H_{17}F_6NO$: 402.1287 $[M+H]^+$; found: 402.1289.

<u>Amine **D5**:</u> Amine **D5** was obtained in 64% yield (0.88 mmol scale) as a yellow oil according to general procedure C. $[\alpha]_D^{20}$ = -149.2 (c = 1.000, DCM); IR (film): $\tilde{\nu}$ = 3058, 3026, 2953, 2906, 2866, 1661, 1481, 1447, 1392, 1357, 1318, 1277, 1208, 1153, 1073, 1025, 751, 700 cm^{-1}; ^{1}H NMR (300 MHz, δ, $CDCl_3$, 298 K): 0.82 (s, 9H), 0.96-1.10 (m, 1H), 1.46-1.64 (m, 1H), 1.74-1.91 (m, 1H), 1.91-2.08 (m, 1H), 2.53-2.65 (m, 1H), 2.87-3.00 (m, 1H), 4.10 (dd, *J* = 4.0, 7.4 Hz, 1H), 4.55 (s, 1H), 7.02-7.24 (m, 6H), 7.33-7.45 (m, 3H), 7.68-7.76 (m, 1H) ppm; ^{13}C NMR (75 MHz, δ, $CDCl_3$, 298 K): 25.1, 25.3, 27.8, 36.9, 55.6, 74.3, 88.1, 107.3, 126.3, 126.3, 126.6, 126.9,

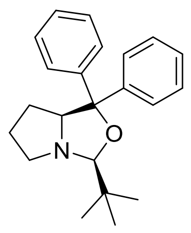

D5

18) Dalicsek, Z.; Pollreisz, F.; Soos, T., *Chem. Commun.*, **2009**, 4587–4589.

127.7, 127.8, 128.3, 130.1, 132.4, 137.6, 144.6, 146.9 ppm; HRMS (ESI): *m/z* calcd. for $C_{22}H_{27}NO$: 322.2165 $[M+H]^+$; found: 322.2173.

Amine **D6**: Amine **D6** was obtained in 33% yield (0.56 mmol scale) as a yellow oil according to general procedure C. Analytical data were in accordance to literature.[16] $[\alpha]_D^{20}$ = -191.8 (c = 0.588, DCM); IR (film): $\tilde{\nu}$ = 3085, 3058, 3025, 2954, 2934, 2871, 1655, 1599, 1491, 1447, 1388, 1324, 1089, 1013, 750, 700 cm^{-1}; ^1H NMR (300 MHz, δ, $CDCl_3$, 298 K): 0.89 (t, *J* = 7.2 Hz, 3H), 1.24-1.92 (m, 10H), 2.75-2.89 (m, 1H), 3.07-3.2 (m, 1H), 4.41 (t, *J* = 6.6 Hz, 1H), 4.71 (d, *J* = 6.0 Hz, 1H), 7.13-7.36 (m, 6H), 7.37-7.47 (m, 2H), 7.51-7.61 (m, 2H) ppm; ^{13}C NMR (75 MHz, δ, $CDCl_3$, 298 K): 14.1, 22.7, 24.8, 27.7, 28.3, 37.0, 53.2, 72.9, 88.1, 98.8, 126.2, 126.2, 126.3, 126.7, 127.8, 128.0, 144.7, 147.8 ppm; HRMS (ESI): *m/z* calcd. for $C_{22}H_{27}NO$: 322.2165 $[M+H]^+$; found: 322.2167.

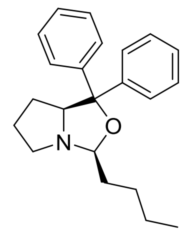

D6

Amine **D7**: Amine **D7** was obtained in around 80% crude yield in >80% purity (which can be used in the next step) and 49% isolated yield after column chromatography (partial decomposition) (32.7 mmol scale) as a brown oil according to general procedure C. $[\alpha]_D^{20}$ = -125.05 (c = 0.986, DCM); IR (film): $\tilde{\nu}$ = 3058, 3024, 2923, 2851, 1491, 1448, 1154, 1020, 751, 700 cm^{-1}; ^1H NMR (300 MHz, δ, $CDCl_3$, 298 K): 0.83-1.20 (m, 6H), 1.28-1.42 (m, 1H), 1.50-1.68 (m, 6H), 1.83-1.93 (m, 1H), 1.93-2.02 (m, 1H), 2.57-2.69 (m, 1H), 2.94-3.05 (m, 1H), 4.22 (t, *J* = 6.5 Hz, 1H), 4.34 (d, *J* = 7.0 Hz, 1H), 7.00-7.29 (m, 8H), 7.35-7.44 (m, 2H) ppm; ^{13}C NMR (75 MHz, δ, $CDCl_3$, 298 K): 25.1, 25.8, 25.9, 26.6, 28.3, 28.7, 28.8, 44.5, 54.7, 73.0, 87.8, 103.5, 126.3, 126.4, 126.7, 127.8, 128.0, 128.3, 130.0, 132.4, 137.6, 144.6, 147.2 ppm; HRMS (ESI): *m/z* calcd. for $C_{24}H_{29}NO$: 348.2322 $[M+H]^+$; found: 348.2326.

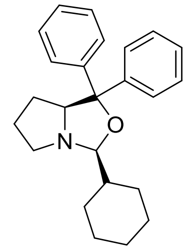

D7

2.2.3 Synthesis of Chiral Ammonium Salts

General procedure D for the synthesis of ammonium acetamides: In analogy to literature,[19] to a solution of 1 equiv. a-bromo amide in THF (10 ml/g amide) was added 1 equiv. amine and the resulting solution was either stirred at RT for 24 hours or refluxed for 24 or 48 hours. The reaction mixture was evaporated to dryness and the crude product was then purified as stated below.

Ammonium salt **1-A**: Obtained in 89% (0.45 mmol scale) according to general procedure D (stirred for 24 h at RT) after column chromatography (silica gel, DCM:MeOH = 3:1). $[\alpha]_D^{20}$= +88.1° (c = 0.5, MeOH); IR(film): $\tilde{\nu}$ = 2923, 2853, 1643, 1456, 1266, 1215, 1102, 895, 832, 752, 726, 698, 578 cm^{-1}; ^1H NMR (700 MHz, δ, $CDCl_3$, 298 K): 0.72 (t, *J* = 7.2 Hz, 3H), 1.11 (t, *J* = 7.1 Hz, 3H), 2.93-3.21 (m, 4H), 3.24-3.33 (m, 2H), 3.36-3.49 (m, 1H), 3.52-3.74 (m, 3H), 4.12-4.27 (m, 1H), 4.63 (d, *J* = 9.3 Hz,

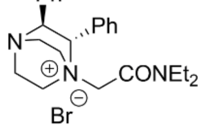

1-A

19) Herchl, R.; Stiftinger, M.; Waser, M., *Org. Biomol. Chem.*, **2011,** *9*, 7023.

1H), 4.66-4.74 (m, 1H), 4.75-4.87 (m, 1H), 5.16-5.36 (m, 1H), 5.92 (d, *J* = 9.2 Hz, 1H), 7.20-7.33 (m, 5H), 7.43- 7.52 (m, 3H), 7.63-7.75 (m, 2H) ppm; ^{13}C NMR (176 MHz, , δ, $CDCl_3$, 298 K): 13.2, 13.9, 40.2, 41.6, 42.4, 47.2, 50.5, 53.7, 58.7, 63.5, 69.2, 127.3, 128.7, 129.2, 130.5, 130.9, 131.9, 136.8, 163.1 ppm; HRMS (ESI): *m/z* calcd. for $C_{24}H_{32}N_3O^+$: 378.2540 $[M]^+$, found: 378.2540.

<u>Ammonium salt **1-B**:</u> Obtained in 88% (2.9 mmol scale) according to general procedure D (stirred for 24 h at RT) after column chromatography (silica gel, DCM:MeOH = 5:1). $[\alpha]_D^{20}$= -14.4° (c = 1.0, MeOH); IR(film): $\bar{\nu}$ = 2967, 2943, 2802, 2752, 1633, 1439, 1383, 1280, 1101, 1033, 909, 792, 594 cm-1; ^{1}H NMR (700 MHz, δ, $CDCl_3$, 298 K): 1.10 (t, *J* = 7.1 Hz, 3H), 1.25 (t, *J* = 7.2 Hz, 3H), 1.35-1.44 (m, 1H), 1.72-1.79 (m, 1H), 1.79-185 (m, 1H), 1.85-1.91 (m, 1H), 2.06-2.14 (m, 1H), 2.24-2.30 (m, 1H), 2.30-2.36 (m, 2H), 2.36-2.42 (m, 1H), 2.42-2.48 (m, 1H), 2.82 (t, *J* = 11.7 Hz, 1H), 2.92 (d, *J* = 12.7 Hz, 1H), 3.03 (t, *J* = 8.8 Hz, 1H), 3.10 (d, *J* = 13.5 Hz, 1H), 3.29 (m, 1H), 3.37 (m, 1H), 3.41 (m, 1H), 3.59 (m, 1H), 4.05 (m, 1H), 4.19-4.25 (m, 1H), 4.56 (m, 1H), 4.62 (d, *J* = 16.5 Hz), 4.93 (m, 1H), 5.32 (d, *J* = 16.5 Hz) ppm; ^{13}C NMR (176 MHz, , δ, $CDCl_3$, 298 K): 12.9, 14.5, 19.3, 21.0, 24.6, 27.9, 40.9, 42.2, 47.9, 52.7, 56.7, 56.8, 57.2, 64.5, 71.9, 162.6 ppm; HRMS (ESI): *m/z* calcd. for $C_{16}H_{30}N_3O$+: 280.2383 $[M]^+$, found: 280.2383.

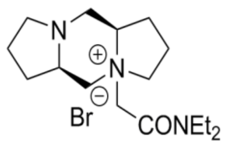

1-B

<u>Ammonium salt **1-C**:</u> Obtained in 74% (2.5 mmol scale) according to general procedure D (stirred for 24 h at RT) after column chromatography (silica gel, DCM:MeOH = 5:1). $[\alpha]_D^{20}$= -22.0° (c = 1.0, MeOH); IR(film): $\bar{\nu}$ = 2972, 2934, 2865, 1642, 1456, 1434, 1273, 1216, 1036, 865, 613 cm $^{-1}$; ^{1}H NMR (700 MHz, δ, $CDCl_3$, 298 K): 1.11 (t, *J* = 7.2 Hz), 1.18-1.36 (m, 3H), 1.24 (t, 3H, *J* = 7.2 Hz), 1.47-1.63 (m, 1H), 1.72-1.81 (m, 1H), 1.86-2.00 (m, 2H), 2.07-2.16 (m, 1H), 2.22 (s, 6H), 2.67-2.78 (m, 1H), 3.23-3.60 (m, 6H), 3.48 (s, 3H), 3.63 (s, 3H), 4.54-4.74 (m, 1H), 5.21-5.56 (m, 2H) ppm; ^{13}C NMR (176 MHz, , δ, $CDCl_3$, 298 K): 12.8, 14.3, 22.8, 24.3, 25.4, 26.9, 39.9, 40.9, 42.2, 49.9, 64.3, 64.6, 69.2, 164.0 ppm; HRMS (ESI): *m/z* calcd. for $C_{16}H_{34}N_3O^+$: 284.2696 $[M]^+$, found: 284.2698.

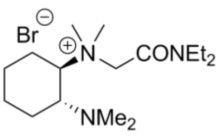

1-C

<u>Ammonium salt **1-D1**:</u> Obtained in 90% yield (1.44 mmol scale) according to general procedure D (stirred for 24 h at RT) after column chromatography (silica gel, DCM:MeOH = 9:1).as a beige solid. $[\alpha]_D^{20}$ = -175.1 (c = 0.989, DCM); IR (film): $\bar{\nu}$ = 3419, 3058, 3029, 2974, 2936, 2914, 2877, 1650, 1489, 1451, 1115, 753, 710; ^{1}H NMR (300 MHz, δ, $CDCl_3$, 298 K): 0.97 (t, J = 7.2 Hz, 3H), 1.07 (t, *J* = 7.2 Hz, 3H), 1.48-1.65 (m, 1H), 1.98-2.15 (m, 1H), 2.43-2.67 (m, 2H), 3.00-3.30 (m, 2H), 3.37-3.62 (m, 2H), 3.74-3.86 (m, 1H), 3.89 (d, *J* = 16.9 Hz, 1H), 4.07-4.20 (m, 1H), 4.57 (d, *J* = 7.3 Hz, 1H), 5.64 (d, *J* = 7.3 Hz, 1H), 6.31 (d, J = 16.9 Hz, 1H), 6.76 (t, *J* = 7.9 Hz, 1H), 7.20-7.42 (m, 6H), 7.43-7.52 (m, 2H), 7.79-7.87 (m, 2H) ppm; ^{13}C NMR (75 MHz, δ, $CDCl_3$, 298 K): 12.7, 13.9, 25.4, 30.1, 40.6, 42.1, 64.7, 67.8, 80.5, 90.6, 94.1, 124.9, 126.0, 128.1, 128.8, 129.2, 129.9, 138.7, 139.5, 163.0 ppm; HRMS (ESI): m/z calcd. for $C_{24}H_{31}N_2O_2^+$: 379.2380 $[M]^+$; found: 379.2379.

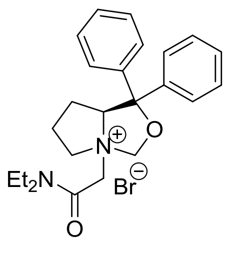

1-D1

Ammonium salt **1-D2**: Obtained in 70% yield (0.41 mmol scale) according to general procedure D (stirred for 24 h at RT) after column chromatography (silica gel, DCM:MeOH = 9:1) as a beige solid. $[\alpha]_D^{20}$ = -133.1 (c = 0.801, DCM); ^{1}H NMR (300 MHz, δ, $CDCl_3$, 298 K): 0.85 (t, *J* = 7.2 Hz, 3H), 1.06 (t, *J* = 7.2 Hz, 3H), 1.51-1.74 (m, 1H), 2.01-2.20 (m, 1H), 2.46-2.67 (m, 2H), 3.02-3.28 (m, 2H), 3.32-3.63 (m, 2H), 3.76-3.91 (m, 1H), 4.13 (d, *J* = 16.8 Hz, 1H), 4.09-4.24 (m, 1H), 4.59 (d, *J* = 7.2 Hz, 1H), 5.76 (d, *J* = 7.2 Hz, 1H), 6.31 (d, *J* = 16.8 Hz, 1H), 7.14 (t, *J* = 7.9 Hz, 1H), 7.39-7.62 (m, 6H), 7.67-7.92 (m, 6H), 8.01-8.11 (m, 1H), 8.23-8.29 (m, 1H) ppm; ^{13}C NMR (75 MHz, δ, $CDCl_3$, 298 K): 12.7, 13.7, 25.4, 30.2, 40.6, 42.2, 65.0, 67.7, 80.1, 91.1, 94.0, 123.0, 126.5, 126.8, 127.2, 127.3, 127.5, 127.7, 128.3. 129.0, 129.1, 130.2, 163.0 ppm; HRMS (ESI): *m/z* calcd. for $C_{32}H_{35}N_2O_2$: 479.2693 $[M]^+$; found: 479.2682.

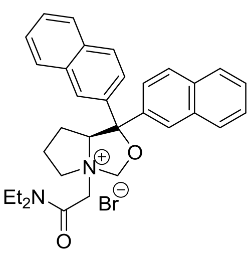

1-D2

Ammonium salt **1-D3**: Obtained in 93% yield (0.43 mmol scale) according to general procedure D (stirred for 24 h at RT) after column chromatography (silica gel, DCM:MeOH = 9:1) as a yellow solid. $[\alpha]_D^{20}$ = -173.0 (c = 0.733, DCM); IR (film): $\tilde{\nu}$ = 3418, 2972, 2837, 1650, 1607, 1583, 1510, 1462, 1304, 1251, 1179, 1114, 1028, 898, 833, 730, 586 cm^{-1}; ^{1}H NMR (300 MHz, δ, $CDCl_3$, 298 K): 1.03 (t, *J* = 7.1 Hz, 3H), 1.08 (t, *J* = 7.1 Hz, 3H), 1.49-1.63 (m, 1H), 1.97-2.18 (m, 1H), 2.32-2.56 (m, 2H), 3.05-3.32 (m, 2H), 3.37-3.58 (m, 2H), 3.77 (s, 3H), 3.78 (s, 3H), 3.67-3.81 (m, 1H), 3.92 (d, *J* = 16.8 Hz, 1H), 4.03-4.14 (m, 1H), 4.53 (d, *J* = 7.2 Hz, 1H), 5.61 (d, *J* = 7.2 Hz, 1H), 6.11 (d, *J* = 16.8 Hz, 1H), 6.54 (t, *J* = 7.8 Hz), 6.77-6.86 (m, 2H), 6.92-7.02 (m, 2H), 7.18-7.30 (m, 2H), 7.40-7.47 (m, 2H) ppm; ^{13}C NMR (75 MHz, δ, CDCl3, 298 K): 12.7, 14.0, 25.3, 30.3, 40.7, 42.1, 55.4, 56.8, 64.5, 67.7, 80.7, 90.3, 93.9, 113.5, 113.9, 114.0, 115.1, 126.2, 126.8, 127.5, 128.4, 163.1 ppm; HRMS (ESI): *m/z* calcd. for $C_{26}H_{35}N_2O_4^+$: 439.2591 $[M]^+$; found: 439.2583.

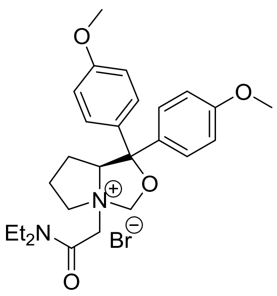

1-D3

Ammonium salt **1-D4**: Obtained in 30% yield (0.40 mmol scale) according to general procedure D (stirred for 24 h at RT) after column chromatography (silica gel, DCM:MeOH = 9:1) as a yellow solid; $[\alpha]_D^{20}$ = -157.9 (c = 0.425, DCM). IR (film): $\tilde{\nu}$ = 3426, 2976, 2941, 1652, 1619, 1417, 1323, 1272, 1168, 1122, 1069, 988, 852, 835, 612 cm^{-1}; ^{1}H NMR (300 MHz, δ, $CDCl_3$, 298 K): 1.00 (t, *J* = 7.1 Hz, 3H), 1.08 (t, *J* = 7.1 Hz, 3H), 1.44-1.62 (m, 1H), 2.02-2.19 (m, 1H), 2.45-2.65 (m, 2H), 3.06-3.34 (m, 2H), 3.37-3.55 (m, 2H), 3.73-3.89 (m, 1H), 3.83 (d, *J* = 16.7 Hz, 1H), 4.08-4.21 (m, 1H), 4.54 (d, *J* = 7.6 Hz, 1H), 5.75 (d, *J* = 7.2 Hz, 1H), 6.17 (d, *J* = 16.7 Hz, 1H), 7.20 (t, *J* = 8.1 Hz), 7.52-7.64 (m, 4H), 7.74-7.81 (m, 2H), 8.08-8.16 (m, 2H) ppm; ^{13}C NMR (75 MHz, δ, $CDCl_3$, 298 K): 12.7, 13.9, 25.2, 30.5, 40.7, 42.1, 65.1, 68.0, 80.7, 90.1, 93.9, 125.4, 126.1 (d, *J* = 3.7 Hz), 126.8, 127.1 (d, *J* = 3.7 Hz), 131.8, 132.2, 142.1, 142.4, 162.6 ppm; HRMS (ESI): *m/z* calcd. for $C_{26}H_{29}F_6N_2O_2^+$: 515.2128 $[M]^+$; found 515.2117.

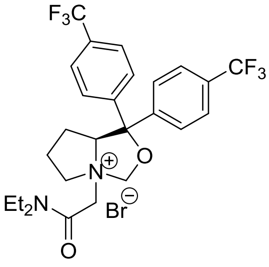

1-D4

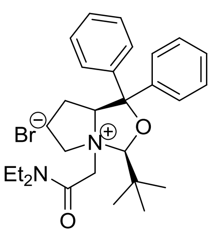

1-D5

Ammonium salt **1-D5**: Obtained in 43% yield (2.64 mmol scale) according to

general procedure D (refluxed for 24 h) after column chromatography (silica gel, DCM:MeOH = 9:1) as a yellow solid. $[\alpha]_D^{20}$ = -173.0 (c = 0.733, DCM); ^{1}H NMR (300 MHz, δ, $CDCl_3$, 298 K): 0.74 (t, *J* = 7.3 Hz, 3H), 0.98 (t, *J* = 7.3 Hz, 3H), 0.84-1.22 (m, 9H), 1.45-1.56 (m, 1H), 1.67-1.84 (m, 1H), 1.86-2.07 (m, 1H), 2.29-2.43 (m, 1H), 2.58-2.77 (m, 1H), 2.88-3.16 (m, 4H), 3.31-3.53 (m, 1H), 3.87 (d, *J* = 16.7 Hz, 1H), 4.52 (dd, *J* = 6.3, 9.0 Hz, 1H), 6.02 (d, 1H, *J* = 16.7 Hz), 6.78 (t, *J* = 8.1 Hz, 1H), 7.02-7.50 (m, 6H), 7.55-7.61 (m, 2H), 7.77-7.87 (m, 2H) ppm; ^{13}C NMR (75 MHz, δ, $CDCl_3$, 298 K): 12.9, 13.9, 22.4, 25.1, 28.5, 29.5, 31.4, 40.8, 42.0, 54.5, 60.5, 65.0, 82.6, 104.3, 124.8, 125.4, 125.5, 125.7, 128.0, 128.8, 129.0, 130.0, 163.2 ppm; HRMS (ESI): *m/z* calcd. for $C_{28}H_{39}N_2O_4^+$: 435.3006 $[M]^+$; found: 435.3002.

<u>Ammonium salt **1-D6**: </u>Obtained in 76% yield (0.17 mmol scale) according to general procedure D (refluxed for 24 h) after column chromatography (silica gel, DCM:MeOH = 20:1) as a white solid. $[\alpha]_D^{20}$ = -133.2 (c = 1.152, DCM); IR (film): $\tilde{\nu}$ = 3433, 2964, 2933, 2872, 1656, 1451, 1383, 1271, 1215, 1143, 1100, 1019, 752, 709 cm^{-1}; ^{1}H NMR (300 MHz, δ, $CDCl_3$, 298 K): 0.81 (t, *J* = 7.2 Hz, 3H), 1.02 (t, *J* = 7.1 Hz, 3H), 1.05 (t, *J* = 7.1 Hz, 3H), 1.39-1.66 (m, 3H), 1.69-2.08 (m, 4H), 2.34-2.49 (m, 1H), 2.49-2.62 (m, 1H), 2.85-3.16 (m, 3H), 3.36-3.60 (m, 2H), 3.82-4.05 (m, 2H), 3.94 (d, *J* = 16.8 Hz 1H), 4.59 (d, *J* = 8.8 Hz, 1H), 6.06 (d, *J* = 16.8 Hz, 1H), 6.85 (t, *J* = 8.1 Hz, 1H), 7.03-7.53 (m, 6H), 7.53-7.69 (m, 2H), 7.82-7.93 (m, 2H) ppm; ^{13}C NMR (75 MHz, δ, $CDCl_3$, 298 K): 12.7, 13.8, 13.9, 22.4, 25.1, 28.5, 29.5, 31.3, 40.8, 42.0, 46.3, 60.3, 65.0, 82.6, 88.2, 104.3, 124.8, 125.7, 128.0, 128.4, 128.6, 128.8, 129.0, 130.0, 139.7, 139.9, 162.7 ppm; HRMS (ESI): *m/z* calcd. for $C_{28}H_{39}N_2O_2^+$: 435.3006 $[M]^+$; found: 435.3000.

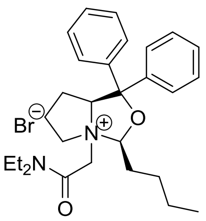

1-D6

<u>Ammonium salt **1-D7**: </u>Obtained in 43 % yield (2.64 mmol scale) according to general procedure D (refluxed for 24 h) after column chromatography (silica gel, DCM:MeOH = 20:1) as a beige solid. $[\alpha]_D^{20}$ = -160.4 (c = 1.006, DCM); IR (film): $\tilde{\nu}$ = 3412, 2931, 2855, 1655, 1450, 1383, 1270, 1217, 1140, 750, 709 cm^{-1}; ^{1}H NMR (300 MHz, δ, $CDCl_3$, 298 K): 0.73 (t, *J* = 7.2 Hz, 3H), 1.04 (t, *J* = 7.2 Hz, 3H), 1.09-1.58 (m, 5H), 1.63-1.94 (m, 6H), 1.96-2.09 (m, 1H), 2.10-2.21 (m, 1H), 2.71-3.08 (m, 4H), 3.35-3.55 (m, 2H), 3.76-4.00 (m, 2H), 3.89 (d, *J* = 16.8 Hz), 4.37 (s, 1H), 6.09 (d, *J* = 16.8 Hz, 1H), 6.81 (dd, *J* = 6.3, 11.1 Hz), 7.39-7.52 (m, 4H), 7.18-7.36 (m, 4H), 7.83-7.97 (m, 2H) ppm; ^{13}C NMR (75 MHz, δ, $CDCl_3$, 298 K): 12.8, 13.7, 24.8, 25.5, 26.0, 26.4, 27.1, 30.0, 32.5, 38.9, 40.7, 41.9, 61.2, 66.2, 81.9, 87.2, 108.2, 124.8, 125.7, 128.0, 128.7, 129.0, 130.0, 139.3, 139.9, 162.9 ppm; HRMS (ESI): *m/z* calcd. for $C_{30}H_{41}N_2O_2^+$: 461.3163 $[M]^+$; found: 461.3148.

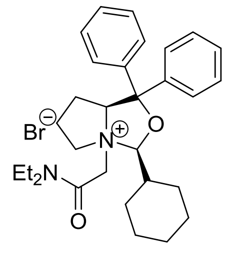

1-D7

<u>Ammonium salt **1-D8**</u>: Obtained in 35% yield (0.21 mmol scale) as a beige solid (refluxed for 48 h). ^{1}H NMR (300 MHz, δ, $CDCl_3$, 298 K): 1.21-1.58 (m, 5H), 1.69-1.95 (m, 5H), 1.98-2.09 (m, 1H), 2.09-2.24 (m, 1H), 2.67-2.95 (m, 2H), 3.29-3.57 (m, 3H), 3.12-3.35 (m, 2H), 3.53-3.76 (m, 4H), 3.78-3.88 (m, 2H), 3.95 (d, *J* = 16.9 Hz, 1H), 4.47 (s, 1H), 6.20 (d, *J* = 16.9 Hz, 1H), 6.74 (dd, *J* = 6.7, 10.5 Hz, 1H), 7.21-7.52 (m, 8H), 7.77-7.88 (m, 2H) ppm. ^{13}C NMR (75 MHz, δ, $CDCl_3$, 298 K): 24.3, 24.9, 25.6, 25.9, 26.4, 27.3, 30.0, 32.3, 38.9, 42.2, 45.8, 61.0, 65.7, 66.1, 66.3, 66.4, 66.8, 81.8, 108.2, 124.8, 125.4, 125.7, 128.0, 128.1,

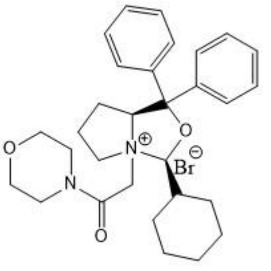

128.8, 129.2, 129.9, 162.6 ppm. HRMS (ESI): *m/z* calcd. for $C_{30}H_{39}N_2O_3^+$: 475.2955 $[M]^+$; found: 475.2947.

<u>Ammonium salt **1-D9:**</u> Obtained in 35% yield (0.21 mmol scale) as a beige solid (refluxed for 48 h).

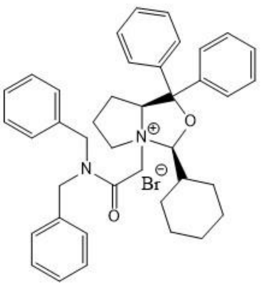

$[\alpha]_D^{20}$ (c = 0.500, DCM) = -74.2. ^{1}H NMR (300 MHz, δ, $CDCl_3$, 298 K): 1.32-1.61 (m, 5H), 1.66-1.97 (m, 6H), 1.98-2.09 (m, 1H), 2.11-2.24 (m, 1H), 2.73-2.90 (m, 1H), 2.94-3.08 (m, 1H), 3.87 (d, *J* = 14.8 Hz, 1H), 3.91-3.99 (m, 2H), 4.05 (d, *J* = 17.1 Hz, 1H), 4.22 (d, *J* = 17.6 Hz, 1H), 4.47 (s, 1H), 4.98 (d, *J* = 17.6 Hz, 1H), 5.06 (d, *J* = 14.8 Hz, 1H), 6.43 (d, *J* = 17.1 Hz, 1H), 6.67 (dd, *J* = 6.5, 10.7 Hz, 1H), 6.78-6.87 (m, 2H), 7.08-7.41 (m, 16H), 7.66-7.75 (m, 2H) ppm. ^{13}C NMR (75 MHz, δ, $CDCl_3$, 298 K): 24.5, 24.8, 25.6, 26.0, 26.4, 27.2, 29.8, 30.0, 30.9, 32.5, 38.9, 47.9, 48.9, 49.8, 61.3, 66.4, 81.7, 108.4, 124.8, 125.2, 125.5, 126.4, 126.5, 127.6, 127.9, 128.0, 128.0, 128.1, 128.2, 128.3, 128.6, 128.7, 128.9, 129.0, 129.1, 129.6, 138.7, 139.9, 164.7 ppm. HRMS (ESI): *m/z* calcd. for $C_{40}H_{45}N_2O_2^+$: 585.3467 $[M]^+$; found: 585.3472.

2.2.4 Reactions of Chiral Ammonium Salts 1-D7 with Electrophiles

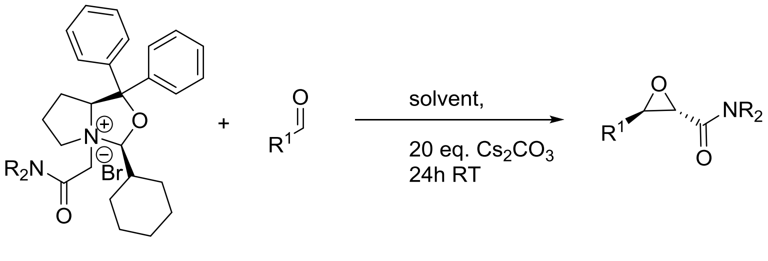

1-D7

General Procedure: The ammonium salt (1 eq.) was dissolved in the given solvent (1 mL/0.1 mmol; A: *i*-PrOH, B: toluene). 20 eq. Cs_2CO_3 were added and after 5 minutes of stirring 2 eq. of the aldehyde were added and the reaction was stirred at RT for 24 hours. The reaction was quenched by addition of H_2O and extracted with DCM. The combined organic phases were dried over Na_2SO_4 and evaporated to dryness. Purification by column chromatography (gradient of heptanes and EtOAc) gave the corresponding epoxides in the reported yields.

Epoxide 3b. Obtained as a white solid (0.5 mmol scale). Analytical data match those reported previously.[19] A: 88% yield, (*e.r.* = 93:7) and B: 66% yield, $[\alpha]_D^{20}$= 106.7 (c = 0.675, DCM, *e.r.* = 95:5); ^{1}H NMR (300 MHz, δ, $CDCl_3$, 298 K): 1.17 (t, *J* = 7.3 Hz, 3H), 1.21 (t, *J* = 7.3 Hz, 3H), 3.39-3.52 (m, 4H), 3.59 (d, *J* = 1.8 Hz, 1H), 4.10 (d, *J* = 1.8 Hz, 1H), 7.32-7.39 (m, 5H) ppm; ^{13}C NMR (125 MHz, δ, $CDCl_3$, 298 K): 13.1, 15.1, 41.0, 41.6, 57.4, 57.7, 125.8, 128.6, 128.7,135.9, 165.9 ppm. The enantioselectivity was determined by HPLC (Chiralcel OD-H, eluent: hexane:*i*-PrOH = 80:20, 0.5 mL/min, 10 °C, retention times: t_{major} (2S,3R) = 15.1 min, t_{minor} (2R,3S) =17.3 min).

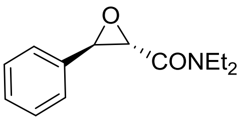

3b

Epoxide 3c. Obtained as a yellow solid. Analytical data match those reported previously,[19] A: 72% yield (*e.r.* = 89:11) and B: 52% yield; $[\alpha]_D^{20}$ = 57.5 (c = 0.725, DCM, *e.r.* = 95:5); ^{1}H NMR (300 MHz, δ, $CDCl_3$, 298 K): 1.10 (t, *J* = 7.2 Hz, 3H), 1.14 (t, *J* = 7.1 Hz, 3H), 2.29 (s, 3H), 3.30-3.42 (m, 4H), 3.51 (d, *J* = 1.9 Hz, 1H), 3.98 (d, *J* = 1.9 Hz, 1H), 7.10 (d, *J* = 8.2 Hz, 2H), 7.16 (d, *J* = 8.2 Hz, 2H) ppm; ^{13}C NMR (75 MHz, δ, $CDCl_3$, 298 K): 13.0, 15.1, 21.3, 40.8, 41.5, 57.2, 57.8, 125.7, 129.4, 132.7, 138.7, 165.9 ppm. The enantioselectivity was determined by HPLC (Chiralcel OD-H, eluent: hexane:*i*-PrOH = 90:10, 0.5 mL/min, 10 °C, retention times: t_{major} (2S,3R) = 14.1 min, t_{minor} (2R,3S) =16.3 min).

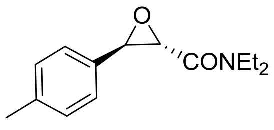

3c

Epoxide 3d. Obtained as a white solid (0.1 mmol scale). Analytical data match those reported previously.[19] A: 84% yield (*e.r.* = 90:10) and B: 62% yield, $[\alpha]_D^{20}$ = -68.2 (c = 1.012, DCM, *e.r.* = 95:5); ^{1}H NMR (300 MHz, δ, $CDCl_3$, 298 K): 1.17 (t, *J* = 7.3 Hz, 3H), 1.21 (t, *J* = 7.3 Hz, 3H), 3.39-3.52 (m, 4H), 3.59 (d, *J* = 1.8 Hz, 1H), 4.10 (d, *J* = 1.8 Hz, 1H), 7.32-7.39 (m, 5H) ppm; ^{13}C NMR (125 MHz, δ, $CDCl_3$, 298 K): 13.1, 15.1, 41.0, 41.6, 57.4, 57.7, 125.8, 128.6, 128.7,135.9, 165.9 ppm. The enantioselectivity was determined by HPLC (Chiralpak AD-H, eluent: hexane:*i*-PrOH = 95:5, 0.5 mL/min, 10 °C, retention times: t_{minor} (2R,3S) =28.5 min , t_{major} (2S,3R) = 30.9 min).

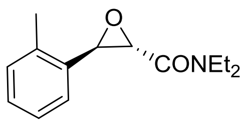

3d

Epoxide 3e. Obtained in as a white solid (0.1 mmol scale). Analytical data match those reported previously.[19] A: 77% yield, (*e.r.* = 87:13) and B: 21% yield, $[\alpha]_D^{20}$ = 63.7 (c = 0.575, MeOH, *e.r.* = 91:9); ^{1}H NMR (300 MHz, δ, $CDCl_3$, 298 K): 1.10 (t, *J* = 7.2 Hz, 3H), 1.15 (t, *J* = 7.2 Hz, 3H), 3.29-3.44 (m, 4H), 3.47 (d, *J* = 1.9 Hz, 1H), 4.01 (d, *J* = 1.9 Hz, 1H), 7.20 (d, *J* = 8.3 Hz, 2H), 7.28 (d, *J* = 8.3 Hz, 2H) ppm; ^{13}C NMR (125 MHz, δ, $CDCl_3$, 298 K): 12.4, 14.5, 40.5, 41.2, 56.5, 56.7, 126.6, 128.4, 133.9, 134.2, 164.9 ppm. The enantioselectivity was determined by HPLC (Chiralcel OD-H, eluent: hexane:*i*-PrOH = 80:20, 0.5 mL/min, 10 °C, retention times: t_{major} (2S,3R) = 18.3 min, t_{minor} (2R,3S) =21.3 min).

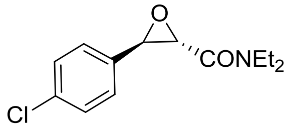

3e

Epoxide 3f. Obtained as a colourless oil (0.1 mmol scale). Analytical data match those reported previously.[19] A: 85% yield (*e.r.* = 85:15) and B: 75% yield, $[\alpha]_D^{20}$ = 76.2 (c = 0.955, DCM, *e.r.* = 86:14); ^{1}H NMR (300 MHz, δ, $CDCl_3$, 298 K): 1.19 (t, *J* = 7.2 Hz, 3H), 1.24 (t, *J* = 7.2 Hz, 3H), 3.41-3.53 (m, 4H), 3.56 (d, *J* = 1.8 Hz, 1H), 4.10 (d, *J* = 1.8 Hz, 1H), 7.23 (d, *J* = 8.5 Hz, 2H), 7.53 (d, *J* = 8.5 Hz, 2H) ppm; ^{13}C NMR (125 MHz, δ, $CDCl_3$, 298 K): 13.1, 15.1, 41.0, 41.6, 57.1, 57.3, 122.8, 127.5, 131.9, 135.1, 165.4 ppm. The enantioselectivity was determined by HPLC (Chiralcel OD-H, eluent: hexane:*i*-PrOH = 80:20, 0.5 mL/min, 10 °C, retention times: t_{major} (2S,3R) = 18.5 min, t_{minor} (2R,3S) =22.4 min).

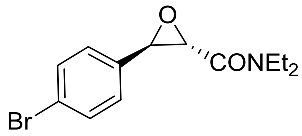

3f

Epoxide 3g. Obtained as a yellow oil (0.1 mmol scale). Analytical data match those reported previously.[19] A: 47% yield (*e.r.* = 91:9) and B: 44% yield, $[\alpha]_D^{20}$ = 50.2 (c = 0.520, MeOH, *e.r.* = 94:6); ^{1}H NMR (300 MHz, δ, $CDCl_3$, 298 K): 1.23 (t, *J* = 7.2 Hz, 3H), 1.29 (t, *J* = 7.2 Hz, 3H), 3.46-3.59 (m, 4H), 3.64 (d, *J* = 1.8 Hz, 1H), 4.29 (d, *J* = 1.8 Hz, 1H), 7.61 (t, *J* = 8.0 Hz, 1H), 7.74 (d, *J* = 7.5 Hz, 1H), 8.25 (m, 2H) ppm; ^{13}C NMR (125 MHz, δ, $CDCl_3$, 298 K): 13.1, 15.1, 41.2, 41.8, 56.5, 57.4, 120.8, 123.7, 129.9, 132.0, 138.4, 148.7, 164.9 ppm. The enantioselectivity was determined by HPLC (Chiralcel OD-H, eluent: hexane:*i*-PrOH = 80:20, 0.5 mL/min, 10 °C, retention times: t_{major} (2S,3R) = 35.3 min, t_{minor} (2R,3S) =45.1 min).

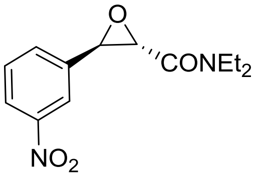

3g

Epoxide 3h. Obtained as a colourless oil (0.1 mmol scale). Analytical data match those reported previously.[19] A: 76% yield (*e.r.* = 91.9) and B: 9% yield, $[\alpha]_D^{20}$ = 56.1 (c = 0.880, DCM, *e.r.* = 94:6); ^{1}H NMR (300 MHz, δ, $CDCl_3$, 298 K): 1.19 (t, *J* = 7.1 Hz, 3H), 1.23 (t, *J* = 7.1 Hz, 3H), 3.38-3.53 (m, 4H), 3.59 (d, *J* = 1.9 Hz, 1H), 3.83 (s, 3H), 4.05 (d, *J* = 1.9 Hz, 1H), 6.92 (d, *J* = 8.8 Hz, 2H), 7.27 (d, *J* = 8.8 Hz, 2H) ppm; ^{13}C NMR (125 MHz, δ, $CDCl_3$, 298 K): 12.6, 14.7, 40.8, 41.4, 55.3, 57.0, 57.6, 114.1, 127.0, 127.7, 160.1, 165.8 ppm; The enantioselectivity was determined by HPLC (Chiralcel OD-H, eluent: hexane:*i*-PrOH = 80:20, 0.5 mL/min, 10 °C, retention times: t_{major} (2S,3R) = 19.1 min, t_{minor} (2R,3S) = 22.5 min).

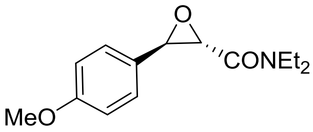

3h

Epoxide 3i. Obtained as a yellow solid (0.1 mmol scale). Analytical data match those reported previously.[19] A: 94% yield (*e.r.* = 92:8) and B: 53% yield, $[\alpha]_D^{20}$ = -17.1 (c = 1.545, DCM, *e.r.* = 96:4); ^{1}H NMR (300 MHz, δ, $CDCl_3$, 298 K): 1.18 (t, *J* = 7.1 Hz, 3H), 1.21 (t, *J* = 7.1 Hz, 3H), 3.29-3.55 (m, 4H), 3.49 (d, *J* = 1.9 Hz, 1H), 3.85 (s, 3H), 4.35 (d, *J* = 1.9 Hz, 1H), 6.89 (d, *J* = 8.1 Hz, 2H), 6.96 (t, *J* = 7.4 Hz, 2H) 7.24 (d, *J* = 7.7 Hz), 7.29 (t, *J* = 8.0 Hz) ppm; ^{13}C NMR (125 MHz, δ, $CDCl_3$, 298 K): 13.0, 14.7, 40.8, 41.4, 53.9, 55.5, 56.7, 110.3, 120.9, 124.5, 125.5, 129.6, 158.1, 166.1 ppm. The enantioselectivity was determined by HPLC (Chiralcel OD-H, eluent: hexane:*i*-PrOH = 80:20, 0.5 mL/min, 10 °C, retention times: t_{major} (2S,3R) = 19.2 min, t_{minor} (2R,3S) =26.4 min.)

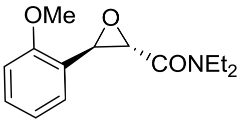

3i

Epoxide 3k. Obtained as a yellow solid. Analytical data match those reported previously.[19] A: 76% yield (*e.r.* = 73:27) and B: 25% yield, $[\alpha]_D^{20}$ = 11.0 (c = 0.250, MeOH, *e.r.* = 81:19); ^{1}H NMR (300 MHz, δ, $CDCl_3$, 298 K): 3.70 (d, *J* = 1.9 Hz, 1H), 4.14 (d, *J* = 1.9 Hz, 1H), 4.56 (s, 2H), 4.67 (d, *J* = 5.1 Hz, 2H), 7.14-7.40 (m, 15H) ppm; ^{13}C NMR (125 MHz, δ, $CDCl_3$, 298 K): 48.8, 49.5, 57.5, 58.2, 125.8, 126.8, 127.8, 128.0, 128.4, 128.7, 128.7, 128.8, 129.2, 135.5, 135.9, 136.5, 167.3 ppm. The enantioselectivity was determined by HPLC (Chiralcel OD-H, eluent: hexane:*i*-PrOH = 90:10, 0.5 mL/min, 10 °C, retention times: t_{major} (2S,3R) = 49.3 min, t_{minor} (2R,3S) =57.1 min.

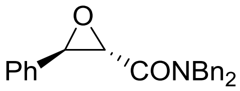

3k

Epoxide 3l. Obtained as a colourless oil. Analytical data match those reported previously.[19] A: 51% yield (*e.r.* = 89:11) and B: 23% yield, $[\alpha]_D^{20}$ = 41.5 (c = 0.320, DCM, *e.r.* = 91:9); ^{1}H NMR (300 MHz, δ, $CDCl_3$, 298 K): 3.66 (d, *J* = 2.0 Hz, 1H), 3.62-3.78 (m, 7H), 4.14 (d, *J* = 2.0 Hz, 1H), 7.31-7.46 (m, 5H) ppm; ^{13}C NMR (125 MHz, δ, $CDCl_3$, 298 K): 42.5, 45.6, 57.4, 57.8, 66.8, 66.8, 125.8, 128.8, 129.0, 135.4, 165.3 ppm. The enantioselectivity was determined by HPLC (Chiralpak AD-H, eluent: hexane:*i*-PrOH = 88:12, 0.5 mL/min, 10 °C, retention times: t_{major} (2S,3R) = 38.7 min, t_{minor} (2R,3S) =46.7 min.

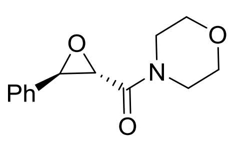

3l

3. Computational results – Energies and Geometries

3.1 Reaction of amide-stabilized trimethylammonium ylide with benzaldehyde

3.1.1 PhCHO

E(B3LYP/6-31G*(dichloromethane)) = -884.058884
E(B3LYP-D3/6-311+G**(dichloromethane)) = -884.304324
G_{tot}(B3LYP/6-31G*(dichloromethane)) = -883.721283

Number of imaginary frequencies: 0

C1	-1.08600	0.82060	0.00000	C6	-0.39980	2.03540	0.00000	H11	-0.94920	2.97370	0.00000
C2	-0.37450	-0.38940	0.00000	H7	-2.17500	0.80330	0.00000	C12	-1.11950	-1.66460	0.00000
C3	1.03120	-0.37670	0.00000	H8	1.56820	-1.32170	0.00000	O13	-0.60710	-2.77660	0.00000
C4	1.71320	0.83620	0.00000	H9	2.80070	0.85040	0.00000	H14	-2.22430	-1.55790	0.00000
C5	0.99810	2.04160	0.00000	H10	1.53440	2.98820	0.00000				

3.1.2 Ylide

E(B3LYP/6-31G*(dichloromethane)) = -884.058884
E(B3LYP-D3/6-311+G**(dichloromethane)) = -884.304324
G_{tot}(B3LYP/6-31G*(dichloromethane)) = -883.721283

Number of imaginary frequencies: 0

C1	0.93890	-0.78370	-0.17800	H10	4.18830	-0.03000	-0.95550	C19	-1.83800	1.43150	0.67360
H2	0.46150	-1.65580	-0.58960	H11	2.78280	-0.17130	-2.06600	H20	-2.71240	1.82100	0.13600
N3	2.42290	-0.90890	-0.11450	C12	2.80140	-2.30460	-0.52150	H21	-2.20310	0.92600	1.58530
C4	2.92560	-0.66740	1.28970	H13	3.88680	-2.41440	-0.45960	H22	-1.20020	2.26270	0.96820
H5	2.48290	-1.42570	1.93700	H14	2.31040	-3.00970	0.15230	C23	-1.81560	-0.63130	-0.62000
H6	4.01780	-0.73650	1.30490	H15	2.46240	-2.47230	-1.54590	H24	-2.83480	-0.33140	-0.88460
H7	2.58560	0.32610	1.57900	C16	0.32530	0.44110	0.08210	H25	-1.35710	-1.07280	-1.50920
C8	3.10230	0.06480	-1.05020	O17	0.91460	1.48190	0.53000	H26	-1.88550	-1.41670	0.15310
H9	2.76630	1.06160	-0.76750	N18	-1.07340	0.54140	-0.19040				

3.1.3 *cis*-TSadd

E(B3LYP/6-31G*(dichloromethane)) = -884.058884
E(B3LYP-D3/6-311+G**(dichloromethane)) = -884.304324
G_{tot}(B3LYP/6-31G*(dichloromethane)) = -883.721283

Number of imaginary frequencies: 0

C1	0.71140	0.91100	0.42900	C15	1.93960	0.74320	2.61410	H29	-3.95090	-0.64230	-2.09070
C2	-0.59120	2.29930	-0.69600	H16	1.97280	1.14210	3.63250	C30	1.95600	0.72700	-0.32160
H3	0.30740	2.80380	-1.09850	H17	1.67030	-0.31530	2.63630	N31	2.01660	-0.32780	-1.21460
O4	-1.33890	2.91830	0.11610	H18	2.89970	0.88230	2.11820	O32	2.91200	1.53970	-0.24960
H5	0.08340	0.03450	0.53940	C19	-2.34920	-0.44730	-3.52610	C33	3.22620	-0.49280	-2.01280
N6	0.88600	1.48670	1.82660	C20	-1.12980	0.17090	-3.81550	H34	2.96120	-0.87150	-3.00640
C7	1.24510	2.95780	1.80300	C21	-0.55190	1.04670	-2.89270	H35	3.72510	0.47040	-2.11040
H8	2.15780	3.06080	1.22060	C22	-1.18330	1.31390	-1.67020	H36	3.92840	-1.20380	-1.54930
H9	0.41280	3.48690	1.33920	C23	-2.41860	0.70720	-1.39890	C37	1.14370	-1.49800	-1.13990
H10	1.39230	3.28870	2.83440	C24	-2.99420	-0.17360	-2.31440	H38	1.28880	-2.09690	-2.04360
C11	-0.43440	1.33530	2.54150	H25	-2.80020	-1.13050	-4.24330	H39	1.37560	-2.13370	-0.27130
H12	-1.20120	1.83410	1.94510	H26	-0.63180	-0.02580	-4.76280	H40	0.09090	-1.21500	-1.10680
H13	-0.65270	0.26960	2.64910	H27	0.39410	1.53250	-3.12350				
H14	-0.34710	1.79570	3.52910	H28	-2.92230	0.94780	-0.46640				

3.1.4 *trans*-TSadd

E(B3LYP/6-31G*(dichloromethane)) = -884.058884
E(B3LYP-D3/6-311+G**(dichloromethane)) = -884.304324
G_{tot}(B3LYP/6-31G*(dichloromethane)) = -883.721283

Number of imaginary frequencies: 0

C1	0.22250	0.88710	0.30250	H15	-3.07280	4.45290	-2.85100	H29	2.71410	2.05510	0.94990
C2	-1.30260	0.82680	-0.42680	H16	-2.85250	2.98830	-0.83020	C30	1.35960	0.45280	-0.61990
C3	-1.37230	1.72000	-1.69580	N17	0.58650	2.18960	1.06230	N31	1.46300	-0.88130	-0.90290
C4	-0.65530	1.42790	-2.86580	C18	0.59350	3.42780	0.19530	O32	2.11670	1.27860	-1.15900
C5	-0.77940	2.22440	-4.00730	H19	1.23520	3.24100	-0.66160	C33	0.79990	-1.98340	-0.20520
C6	-1.63850	3.32730	-4.00430	H20	-0.42400	3.64010	-0.12100	H34	1.52550	-2.53070	0.41450
C7	-2.38350	3.60980	-2.85640	H21	0.97820	4.25190	0.80090	H35	-0.01620	-1.64400	0.42930
C8	-2.25720	2.80410	-1.72070	C22	-0.43610	2.39360	2.16370	H36	0.38340	-2.68050	-0.94190
O9	-2.29220	0.97820	0.44060	H23	-1.42410	2.26850	1.71370	C37	2.47820	-1.31590	-1.86250
H10	0.09510	0.17700	1.12050	H24	-0.27720	1.62800	2.92750	H38	2.04140	-2.05460	-2.54320
H11	-1.19760	-0.21280	-0.83680	H25	-0.27160	3.38580	2.59120	H39	2.83130	-0.45730	-2.42980
H12	-0.00300	0.55880	-2.89880	C26	1.94380	2.06320	1.71840	H40	3.33060	-1.77860	-1.34780
H13	-0.21120	1.97810	-4.90280	H27	2.07810	2.91920	2.38530				
H14	-1.73710	3.94880	-4.89280	H28	1.96660	1.13670	2.29760				

3.1.5 *cis-syn*-Betaine

E(B3LYP/6-31G*(dichloromethane)) = -884.058884
E(B3LYP-D3/6-311+G**(dichloromethane)) = -884.304324
G_{tot}(B3LYP/6-31G*(dichloromethane)) = -883.721283

Number of imaginary frequencies: 0

C1	0.27820	1.26930	0.18220	C15	1.87290	1.10140	2.13970	H29	-3.88840	-0.65430	-2.51410
C2	-0.76110	2.28480	-0.59560	H16	2.00940	1.41690	3.17740	C30	1.53380	1.01760	-0.65410
H3	-0.06130	3.03360	-1.03860	H17	1.69570	0.02370	2.10620	N31	1.75770	-0.22560	-1.15800
O4	-1.71420	2.80880	0.18410	H18	2.75270	1.36330	1.55260	O32	2.31990	1.95230	-0.89420
H5	-0.25590	0.34780	0.41100	C19	-2.29460	-0.14960	-3.88120	C33	2.93470	-0.37550	-2.02330
N6	0.66920	1.81590	1.58260	C20	-1.12540	0.59430	-4.06070	H34	2.99810	-1.41020	-2.36410
C7	0.94570	3.30170	1.54750	C21	-0.62850	1.38260	-3.01760	H35	2.86370	0.28480	-2.89460
H8	1.73710	3.47990	0.82120	C22	-1.28690	1.43850	-1.78150	H36	3.85140	-0.12040	-1.48050
H9	0.01240	3.78290	1.25290	C23	-2.47090	0.70670	-1.62210	C37	0.89250	-1.38790	-0.91710
H10	1.25730	3.60920	2.54860	C24	-2.96950	-0.08760	-2.65680	H38	1.29020	-2.23770	-1.47370
C11	-0.50340	1.56180	2.50630	H25	-2.68270	-0.76430	-4.69140	H39	0.87110	-1.66740	0.14330
H12	-1.37770	1.97160	1.99350	H26	-0.60270	0.56480	-5.01520	H40	-0.12930	-1.20740	-1.26220
H13	-0.59250	0.48360	2.66110	H27	0.27990	1.96440	-3.16810				
H14	-0.30330	2.05990	3.45810	H28	-2.99820	0.79270	-0.67520				

3.1.6 *trans-syn*-Betaine

E(B3LYP/6-31G*(dichloromethane)) = -884.058884
E(B3LYP-D3/6-311+G**(dichloromethane)) = -884.304324
G_{tot}(B3LYP/6-31G*(dichloromethane)) = -883.721283

Number of imaginary frequencies: 0

C1	0.22250	0.88710	0.30250	H11	-1.19760	-0.21280	-0.83680	H21	0.97820	4.25190	0.80090
C2	-1.30260	0.82680	-0.42680	H12	-0.00300	0.55880	-2.89880	C22	-0.43610	2.39360	2.16370
C3	-1.37230	1.72000	-1.69580	H13	-0.21120	1.97810	-4.90280	H23	-1.42410	2.26850	1.71370
C4	-0.65530	1.42790	-2.86580	H14	-1.73710	3.94880	-4.89280	H24	-0.27720	1.62800	2.92750
C5	-0.77940	2.22440	-4.00730	H15	-3.07280	4.45290	-2.85100	H25	-0.27160	3.38580	2.59120
C6	-1.63850	3.32730	-4.00430	H16	-2.85250	2.98830	-0.83020	C26	1.94380	2.06320	1.71840
C7	-2.38350	3.60980	-2.85640	N17	0.58650	2.18960	1.06230	H27	2.07810	2.91920	2.38530
C8	-2.25720	2.80410	-1.72070	C18	0.59350	3.42780	0.19530	H28	1.96660	1.13670	2.29760
O9	-2.29220	0.97820	0.44060	H19	1.23520	3.24100	-0.66160	H29	2.71410	2.05510	0.94990
H10	0.09510	0.17700	1.12050	H20	-0.42400	3.64010	-0.12100	C30	1.35960	0.45280	-0.61990

N31	1.46300	-0.88130	-0.90290	H35	-0.01620	-1.64400	0.42930	H39	2.83130	-0.45730	-2.42980
O32	2.11670	1.27860	-1.15900	H36	0.38340	-2.68050	-0.94190	H40	3.33060	-1.77860	-1.34780
C33	0.79990	-1.98340	-0.20520	C37	2.47820	-1.31590	-1.86250				
H34	1.52550	-2.53070	0.41450	H38	2.04140	-2.05460	-2.54320				

3.1.7 *cis*-TSrot
Not detected.

3.1.8 *trans*-TSrot
E(B3LYP/6-31G*(dichloromethane)) = -884.058884
E(B3LYP-D3/6-311+G**(dichloromethane)) = -884.304324
G_{tot}(B3LYP/6-31G*(dichloromethane)) = -883.721283

Number of imaginary frequencies: 0

C1	0.23840	0.98760	0.31630	H15	-4.72370	3.67590	0.88760	H29	2.83670	1.74030	1.11040
C2	-1.19140	0.98700	-0.67780	H16	-3.32550	1.60110	0.87610	C30	1.37590	0.49770	-0.58030
C3	-1.99620	2.30220	-0.64420	N17	0.75300	2.18670	1.19400	N31	1.52450	-0.84110	-0.75330
C4	-1.74720	3.33040	-1.56460	C18	0.96860	3.48510	0.45230	O32	2.10940	1.31660	-1.16730
C5	-2.53800	4.48270	-1.59160	H19	1.64990	3.29300	-0.37180	C33	0.66670	-1.87320	-0.15840
C6	-3.60360	4.61900	-0.69780	H20	0.01010	3.84180	0.08450	H34	1.08710	-2.24140	0.78830
C7	-3.88080	3.58670	0.20460	H21	1.39230	4.20580	1.15680	H35	-0.34790	-1.48760	-0.02560
C8	-3.09200	2.43400	0.21780	C22	-0.25420	2.42810	2.29040	H36	0.61600	-2.71580	-0.85640
O9	-1.88980	-0.09020	-0.36260	H23	-1.21310	2.67620	1.84070	C37	2.60070	-1.33270	-1.61220
H10	-0.04180	0.26410	1.07890	H24	-0.34420	1.52480	2.89780	H38	2.18890	-1.80230	-2.51460
H11	-0.70790	0.97830	-1.68740	H25	0.09860	3.25850	2.90720	H39	3.24150	-0.50120	-1.90030
H12	-0.92750	3.22280	-2.27490	C26	2.05740	1.81230	1.86670	H40	3.19100	-2.08070	-1.06920
H13	-2.32860	5.26770	-2.31600	H27	2.31050	2.59160	2.59080				
H14	-4.22320	5.51340	-0.71660	H28	1.92700	0.85800	2.38180				

3.1.9 *cis-anti*-Betaine
E(B3LYP/6-31G*(dichloromethane)) = -884.058884
E(B3LYP-D3/6-311+G**(dichloromethane)) = -884.304324
G_{tot}(B3LYP/6-31G*(dichloromethane)) = -883.721283

Number of imaginary frequencies: 0

C1	0.38490	1.28810	0.29860	C15	1.88040	0.98050	2.32950	H29	1.39980	4.00490	-4.22160
C2	-0.80100	2.08010	-0.53160	H16	2.02070	1.28970	3.36840	C30	1.67060	1.05600	-0.52040
H3	-1.43170	2.51660	0.28720	H17	1.55990	-0.06340	2.29470	N31	1.72620	-0.03020	-1.33450
O4	-1.42980	1.20340	-1.32070	H18	2.80100	1.12130	1.76830	O32	2.63040	1.84060	-0.41860
H5	-0.09250	0.34260	0.55650	C19	0.67710	5.49380	-2.83470	C33	2.96530	-0.31010	-2.05930
N6	0.79370	1.84180	1.71900	C20	-0.01450	5.70830	-1.64200	H34	3.31150	-1.32370	-1.81950
C7	1.26050	3.27800	1.72940	C21	-0.47480	4.61810	-0.89400	H35	2.79710	-0.24950	-3.14180
H8	2.08270	3.37230	1.02580	C22	-0.24950	3.30070	-1.31060	H36	3.72670	0.41270	-1.77150
H9	0.43230	3.92040	1.44080	C23	0.40620	3.10510	-2.53520	C37	0.64660	-0.99830	-1.54320
H10	1.57900	3.51780	2.74730	C24	0.87850	4.18310	-3.28250	H38	0.80220	-1.89670	-0.92730
C11	-0.40510	1.73080	2.63390	H25	1.04200	6.33640	-3.41910	H39	-0.31800	-0.52170	-1.34880
H12	-1.21400	2.35240	2.25350	H26	-0.20300	6.72310	-1.29560	H40	0.65960	-1.30280	-2.59650
H13	-0.72310	0.68690	2.68120	H27	-1.03540	4.80340	0.02250				
H14	-0.10840	2.07600	3.62720	H28	0.52680	2.09040	-2.90300				

3.1.10 *trans-anti*-Betaine
E(B3LYP/6-31G*(dichloromethane)) = -884.058884
E(B3LYP-D3/6-311+G**(dichloromethane)) = -884.304324
G_{tot}(B3LYP/6-31G*(dichloromethane)) = -883.721283

Number of imaginary frequencies: 0

C1	0.34750	0.94670	0.13650	H15	-0.48010	-2.70940	-3.53510	H29	-0.39200	3.52850	1.06290
C2	1.58110	0.00910	-0.29740	H16	-0.15740	-1.86250	-1.22050	C30	0.56810	1.26550	1.62800
C3	1.42500	-0.50660	-1.74310	N17	0.02670	2.30130	-0.63170	N31	-0.26380	0.67860	2.52640
C4	2.25800	-0.07550	-2.78390	C18	1.27470	3.09380	-0.93230	O32	1.42250	2.10560	1.95880
C5	2.10440	-0.56740	-4.08650	H19	1.81300	3.24320	0.00180	C33	-0.15770	1.04050	3.93760
C6	1.10980	-1.50590	-4.36660	H20	1.88390	2.53570	-1.64180	H34	-1.16220	1.11520	4.36870
C7	0.28830	-1.96610	-3.32910	H21	0.97420	4.04840	-1.37290	H35	0.41010	0.28680	4.49750
C8	0.45680	-1.48070	-2.03290	C22	-0.67760	1.99410	-1.93080	H36	0.34720	2.00230	4.02500
O9	1.62340	-0.97420	0.62080	H23	-0.04830	1.34780	-2.53730	C37	-1.08360	-0.49830	2.20840
H10	-0.56700	0.37600	-0.01900	H24	-1.62690	1.49930	-1.71340	H38	-1.29890	-1.03170	3.13830
H11	2.47100	0.68890	-0.30320	H25	-0.86470	2.93660	-2.45230	H39	-2.04350	-0.21850	1.75140
H12	3.04630	0.64760	-2.57400	C26	-0.91090	3.17140	0.17540	H40	-0.51680	-1.15870	1.54890
H13	2.76480	-0.21940	-4.87890	H27	-1.21480	4.01870	-0.44540				
H14	0.98210	-1.88630	-5.37790	H28	-1.78960	2.58470	0.45170				

3.1.11 *cis*-TSelim

E(B3LYP/6-31G*(dichloromethane)) = -884.058884
E(B3LYP-D3/6-311+G**(dichloromethane)) = -884.304324
G_{tot}(B3LYP/6-31G*(dichloromethane)) = -883.721283

Number of imaginary frequencies: 0

C1	0.26920	0.92110	0.07480	H15	-1.09550	3.40470	-4.60440	H29	2.05090	-0.97970	1.37900
C2	-1.08400	1.44350	-0.33820	H16	-0.39710	3.28770	-2.21580	C30	1.50780	1.49660	-0.59350
H3	0.31970	0.77290	1.14650	N17	0.62200	-1.06330	-0.17790	N31	2.42010	2.11820	0.20860
C4	-1.49200	1.49300	-1.80710	C18	-0.40610	-1.73200	0.64860	O32	1.71520	1.31410	-1.80110
C5	-2.37920	0.54530	-2.33410	H19	-0.34100	-1.37570	1.68100	C33	2.15010	2.61050	1.55940
C6	-2.78140	0.60110	-3.67170	H20	-1.39930	-1.50840	0.25420	H34	2.55450	3.62570	1.65010
C7	-2.31230	1.62560	-4.49700	H21	-0.25560	-2.82050	0.63920	H35	1.07600	2.66260	1.72870
C8	-1.45130	2.59420	-3.97130	C22	0.52480	-1.50500	-1.58650	H36	2.63240	1.98240	2.32070
C9	-1.05000	2.52980	-2.63620	H23	-0.47920	-1.30330	-1.96100	C37	3.69490	2.53550	-0.36980
O10	-0.73350	2.64390	0.24270	H24	1.24320	-0.94500	-2.18400	H38	4.49500	2.36400	0.35940
H11	-1.87620	0.88480	0.20170	H25	0.72910	-2.58320	-1.65580	H39	3.89130	1.95600	-1.27150
H12	-2.77520	-0.23880	-1.68870	C26	1.96770	-1.36110	0.35700	H40	3.68260	3.60320	-0.62990
H13	-3.46930	-0.14540	-4.06430	H27	2.14270	-2.44640	0.36860				
H14	-2.62600	1.67750	-5.53780	H28	2.72350	-0.88900	-0.27360				

3.1.12 *trans*-TSelim

E(B3LYP/6-31G*(dichloromethane)) = -884.058884
E(B3LYP-D3/6-311+G**(dichloromethane)) = -884.304324
G_{tot}(B3LYP/6-31G*(dichloromethane)) = -883.721283

Number of imaginary frequencies: 0

C1	0.42870	0.70550	0.19660	H15	-0.32450	-2.58140	-3.83170	H29	-0.31960	3.56620	1.10450
C2	1.59460	-0.05450	-0.37740	H16	-0.07690	-1.89990	-1.44690	C30	0.66940	1.18410	1.61500
C3	1.48800	-0.48340	-1.83610	N17	-0.08930	2.44230	-0.67020	N31	-0.27040	0.87050	2.55070
C4	2.33140	0.05370	-2.81740	C18	1.14060	3.17680	-1.04510	O32	1.64580	1.90850	1.86720
C5	2.21090	-0.33360	-4.15610	H19	1.74810	3.32280	-0.15090	C33	-0.15100	1.43000	3.89470
C6	1.24740	-1.27440	-4.52750	H20	1.70050	2.59130	-1.77580	H34	-1.14670	1.70160	4.26410
C7	0.41730	-1.83560	-3.55090	H21	0.88160	4.14830	-1.48890	H35	0.29310	0.70310	4.58890
C8	0.54370	-1.44750	-2.21640	C22	-0.87850	2.09070	-1.87280	H36	0.48060	2.31720	3.86110
O9	1.34320	-1.04100	0.55650	H23	-0.28700	1.44470	-2.52230	C37	-1.26420	-0.19400	2.38440
H10	-0.52960	0.26130	-0.04020	H24	-1.79000	1.56470	-1.57320	H38	-1.41050	-0.68880	3.35000
H11	2.54310	0.49450	-0.23310	H25	-1.15930	3.00040	-2.42160	H39	-2.23560	0.19870	2.05350
H12	3.10020	0.77110	-2.53140	C26	-0.91640	3.26590	0.24190	H40	-0.89810	-0.93950	1.67870
H13	2.87480	0.09260	-4.90550	H27	-1.26850	4.16690	-0.28030				
H14	1.15150	-1.57840	-5.56800	H28	-1.78410	2.68960	0.57490				

3.1.13 *cis*-Epoxide

E(B3LYP/6-31G*(dichloromethane)) = -884.058884
E(B3LYP-D3/6-311+G**(dichloromethane)) = -884.304324
G_{tot}(B3LYP/6-31G*(dichloromethane)) = -883.721283

Number of imaginary frequencies: 0

C1	0.67330	-1.69510	1.07970	C10	1.95170	-3.80070	0.47410	O19	1.26550	-1.91980	-2.23730
C2	0.59550	-0.89630	-0.17500	C11	2.93830	-4.74370	0.76810	C20	3.06500	0.57560	-0.30390
O3	-0.32280	-1.95930	0.07450	H12	4.46010	-5.37320	2.16570	H21	3.19900	1.60410	-0.66080
H4	0.27830	-1.20650	1.97370	H13	4.02910	-3.49420	3.74170	H22	2.33620	0.58280	0.50610
H5	0.15380	0.10140	-0.10590	H14	2.26830	-1.82600	3.23280	H23	4.02410	0.22290	0.09780
C6	3.69320	-4.63540	1.93930	H15	1.36400	-3.88430	-0.43420	C24	3.55920	-0.39360	-2.52010
C7	3.45200	-3.58020	2.82360	H16	3.11510	-5.56820	0.08050	H25	3.85470	0.60970	-2.84950
C8	2.46230	-2.63910	2.53460	C17	1.53660	-1.09810	-1.35580	H26	4.46330	-0.94480	-2.22570
C9	1.71150	-2.73520	1.35340	N18	2.62870	-0.28540	-1.39970	H27	3.07240	-0.92090	-3.34040

3.1.14 *trans*-Epoxide

E(B3LYP/6-31G*(dichloromethane)) = -884.058884
E(B3LYP-D3/6-311+G**(dichloromethane)) = -884.304324
G_{tot}(B3LYP/6-31G*(dichloromethane)) = -883.721283

Number of imaginary frequencies: 0

C1	-0.57700	-0.36130	-0.52910	C10	1.53460	0.73680	-2.65470	O19	-1.77830	-0.22670	1.53430
C2	0.71670	0.33820	-0.29620	C11	2.56160	0.73260	-3.59970	C20	-2.24160	-2.96440	1.70420
O3	-0.49850	1.06600	-0.53800	H12	4.66030	0.37190	-3.96470	H21	-1.59730	-3.58960	2.33600
H4	-0.75080	-0.79390	-1.51420	H13	5.14080	-0.24290	-1.60110	H22	-2.71560	-2.19950	2.31810
H5	1.05400	0.39050	0.74120	H14	3.31890	-0.22690	0.08210	H23	-3.01150	-3.60300	1.25290
C6	3.86110	0.37480	-3.22650	H15	0.52740	1.02840	-2.94260	C24	-0.83890	-3.22150	-0.31280
C7	4.13060	0.02820	-1.90000	H16	2.34890	1.01240	-4.62930	H25	-1.58260	-3.62730	-1.01200
C8	3.10470	0.03610	-0.95260	C17	-1.32910	-0.96160	0.64770	H26	-0.05410	-2.72140	-0.87980
C9	1.79810	0.38200	-1.32430	N18	-1.45670	-2.31690	0.65640	H27	-0.37620	-4.05920	0.22250

3.1.15 Trimethylamine

E(B3LYP/6-31G*(dichloromethane)) = -884.058884
E(B3LYP-D3/6-311+G**(dichloromethane)) = -884.304324
G_{tot}(B3LYP/6-31G*(dichloromethane)) = -883.721283

Number of imaginary frequencies: 0

N1	-0.51110	1.07590	1.44770	C6	-1.40010	0.56990	0.40410	H11	-0.88640	-0.52680	2.75840
C2	-0.47280	2.53670	1.43930	H7	-1.06110	0.92090	-0.57790	H12	-0.19850	0.91970	3.52300
H3	0.23220	2.89530	2.19870	H8	-1.38160	-0.52640	0.39870	H13	-1.92130	0.89220	3.06410
H4	-0.13100	2.89510	0.46100	H9	-2.45280	0.89230	0.53640				
H5	-1.45990	2.99790	1.64540	C10	-0.90480	0.56950	2.76090				

3.2 Reaction of ester-stabilized trimethylammonium ylide with benzaldehyde

3.2.1 Ylide

E(B3LYP/6-31G*(dichloromethane)) = -441.61587203602
E(B3LYP-D3/6-311+G**(dichloromethane)) = -441.75501336460
G_{tot}(B3LYP/6-31G*(dichloromethane)) = -441.457690

Number of imaginary frequencies: 0

C1	0.92710	-0.71880	-0.31370	H9	2.79750	1.13200	-0.66950	O17	0.84270	1.50960	0.55790
H2	0.38710	-1.64170	-0.46740	H10	4.20160	0.03090	-0.91070	O18	-1.07660	0.32550	0.10000
N3	2.40220	-0.88530	-0.20040	H11	2.82670	0.01690	-2.06870	C19	-1.80650	1.48930	0.48720
C4	2.87560	-0.77820	1.23350	C12	2.76740	-2.24570	-0.72490	H20	-2.86260	1.23140	0.36700
H5	2.39400	-1.57250	1.80630	H13	3.85240	-2.36580	-0.67740	H21	-1.61370	1.76210	1.53090
H6	3.96400	-0.88390	1.27240	H14	2.27730	-3.00060	-0.10580	H22	-1.56730	2.35100	-0.14590
H7	2.56640	0.19880	1.60380	H15	2.41680	-2.32430	-1.75620				
C8	3.12110	0.15550	-1.02740	C16	0.31980	0.44110	0.15270				

3.2.2 *trans*-TSadd

E(B3LYP/6-31G*(dichloromethane)) = -787.18738212688
E(B3LYP-D3/6-311+G**(dichloromethane)) = -787.41656724555
G_{tot}(B3LYP/6-31G*(dichloromethane)) = -786.924355

Number of imaginary frequencies: 1 (-114.13)

C1	0.35920	1.30880	0.22840	H13	2.42190	-3.13200	2.48510	H25	-0.99580	2.75770	-2.53730
C2	1.59550	0.13610	-0.78400	H14	4.65050	-2.17600	3.04800	C26	-1.15220	3.28600	0.03100
C3	2.43690	-0.49710	0.31150	H15	5.46870	-0.15860	1.84090	H27	-1.47030	4.08690	-0.64320
C4	1.99020	-1.63810	0.98940	H16	4.05430	0.88930	0.07100	H28	-1.99380	2.62530	0.25300
C5	2.77960	-2.24200	1.97050	N17	-0.06870	2.48060	-0.64510	H29	-0.75090	3.71130	0.95120
C6	4.03160	-1.70710	2.28540	C18	1.09120	3.39740	-0.98310	C30	0.84380	1.70700	1.56590
C7	4.48980	-0.57270	1.60670	H19	1.42780	3.87200	-0.06510	O31	1.50190	2.69920	1.86900
C8	3.69940	0.02250	0.62200	H20	1.87280	2.77000	-1.41110	O32	0.50750	0.76130	2.47890
O9	2.19190	0.74430	-1.74660	H21	0.73210	4.13900	-1.70100	C33	1.08110	0.91110	3.79200
H10	-0.51240	0.66290	0.32440	C22	-0.62480	1.92420	-1.93620	H34	0.70950	0.06460	4.37160
H11	0.76510	-0.55140	-1.05530	H23	0.19030	1.40970	-2.44560	H35	2.17220	0.88470	3.73900
H12	1.01600	-2.05950	0.74280	H24	-1.44440	1.24050	-1.70120	H36	0.76040	1.85150	4.24900

3.2.3 *trans-syn*-Betaine

E(B3LYP/6-31G*(dichloromethane)) = -787.18934956987
E(B3LYP-D3/6-311+G**(dichloromethane)) = -787.42174904828
G_{tot}(B3LYP/6-31G*(dichloromethane)) = -786.926025

Number of imaginary frequencies: 0

C1	0.38450	1.04500	0.21900	H13	0.27660	-3.88430	1.86390	H25	-0.16300	2.68740	-2.73120
C2	1.65180	0.08300	-0.32560	H14	2.11090	-3.73580	3.54590	C26	-1.12930	2.94980	-0.32430
C3	1.77630	-1.01560	0.75910	H15	3.74530	-1.86160	3.42340	H27	-1.27040	3.81370	-0.98110
C4	0.87170	-2.08440	0.82870	H16	3.52560	-0.13460	1.61330	H28	-1.94620	2.23570	-0.46140
C5	0.98630	-3.05910	1.82510	N17	0.17990	2.28330	-0.67590	H29	-1.09440	3.28460	0.71450
C6	2.01750	-2.97750	2.76870	C18	1.31050	3.29230	-0.55630	C30	0.50160	1.41590	1.68360
C7	2.93350	-1.92290	2.69860	H19	1.24090	3.78510	0.41060	O31	1.27870	2.21660	2.17850
C8	2.81450	-0.95270	1.69630	H20	2.23330	2.71290	-0.63860	O32	-0.35730	0.67440	2.40960
O9	2.76070	0.71500	-0.64170	H21	1.19160	4.01440	-1.36770	C33	-0.24220	0.78480	3.84370
H10	-0.53500	0.46760	0.09690	C22	0.11730	1.82860	-2.11680	H34	-1.00250	0.11390	4.24540
H11	1.14030	-0.40880	-1.18000	H23	1.10850	1.46640	-2.39180	H35	0.75300	0.47540	4.16880
H12	0.07010	-2.15870	0.09130	H24	-0.63430	1.04050	-2.21190	H36	-0.42900	1.81210	4.16530

3.2.4 *trans*-TSrot

E(B3LYP/6-31G*(dichloromethane)) = -787.18043438855
E(B3LYP-D3/6-311+G**(dichloromethane)) = -787.41128184918
G_{tot}(B3LYP/6-31G*(dichloromethane)) = -786.916461

Number of imaginary frequencies: 1 (-121.78)

C1	0.07540	0.88110	0.34100	H13	0.48670	-2.21900	-3.80700	H25	1.14380	3.62510	-1.41380
C2	1.26250	-0.13200	0.36900	H14	0.24990	-4.47280	-2.77010	C26	-0.78630	3.21460	0.34460
C3	0.95950	-1.34950	-0.53340	H15	0.47020	-4.71890	-0.30260	H27	-0.52920	4.25230	0.11900
C4	0.83290	-1.22350	-1.92530	H16	0.92520	-2.69810	1.11390	H28	-1.56200	2.86640	-0.34180
C5	0.57970	-2.33730	-2.72860	N17	0.44770	2.36020	0.14430	H29	-1.11420	3.11100	1.37720
C6	0.44770	-3.60250	-2.14730	C18	1.53230	2.82320	1.09320	C30	-0.44790	0.55070	1.76870
C7	0.57330	-3.73740	-0.76220	H19	1.17890	2.66270	2.10880	O31	-0.49610	1.30410	2.74160
C8	0.82820	-2.61770	0.03550	H20	2.43600	2.24170	0.90990	O32	-1.29180	-0.51330	1.62110
O9	1.37810	-0.38750	1.71580	H21	1.72030	3.88060	0.89340	C33	-1.75130	-1.11230	2.83850
H10	-0.69750	0.69780	-0.41240	C22	0.91910	2.56490	-1.27500	H34	-2.39720	-1.94150	2.54040
H11	2.17410	0.33180	-0.07460	H23	1.81730	1.97110	-1.44830	H35	-0.90030	-1.48980	3.41380
H12	0.94120	-0.24780	-2.39780	H24	0.12390	2.26150	-1.96050	H36	-2.31520	-0.39480	3.44240

3.2.5 *trans-anti*-Betaine

E(B3LYP/6-31G*(dichloromethane)) = -787.18334201679
E(B3LYP-D3/6-311+G**(dichloromethane)) = -787.41943688039
G_{tot}(B3LYP/6-31G*(dichloromethane)) = -786.920663

Number of imaginary frequencies: 0

C1	0.31800	0.96850	0.09500	H13	2.87850	-0.21020	-4.81570	H25	-0.75140	3.03100	-2.48950
C2	1.50440	-0.03210	-0.29150	H14	1.11290	-1.86440	-5.40860	C26	-0.84890	3.23150	0.15590
C3	1.41110	-0.54110	-1.74140	H15	-0.42440	-2.70940	-3.64020	H27	-1.12200	4.09760	-0.45400
C4	2.28860	-0.09720	-2.74070	H16	-0.19710	-1.88970	-1.29650	H28	-1.74470	2.66590	0.42120
C5	2.18560	-0.56720	-4.05530	N17	0.06830	2.34400	-0.65420	H29	-0.32560	3.56710	1.05210
C6	1.19980	-1.49860	-4.38760	C18	1.35530	3.08780	-0.91020	C30	0.57330	1.22910	1.58020
C7	0.33530	-1.97080	-3.39160	H19	1.88310	3.20670	0.03550	O31	1.52150	1.86720	2.01180
C8	0.44960	-1.50370	-2.08190	H20	1.95980	2.51540	-1.61240	O32	-0.41850	0.75840	2.35170
O9	1.39930	-0.98730	0.65390	H21	1.10980	4.06230	-1.34160	C33	-0.24080	0.91070	3.77700
H10	-0.62520	0.43360	-0.02300	C22	-0.61660	2.07730	-1.97210	H34	-1.13770	0.48380	4.22820
H11	2.43450	0.58860	-0.24110	H23	0.00350	1.41060	-2.56740	H35	0.64910	0.36710	4.10510
H12	3.07360	0.61540	-2.48740	H24	-1.58880	1.61790	-1.77980	H36	-0.14260	1.96750	4.03990

3.2.6 *trans*-TSelim

E(B3LYP/6-31G*(dichloromethane)) = -787.17388501770
E(B3LYP-D3/6-311+G**(dichloromethane)) = -787.40334669018
G_{tot}(B3LYP/6-31G*(dichloromethane)) = -786.913945

Number of imaginary frequencies: 1 (-300.10)

C1	0.39540	0.73640	0.17580	H13	2.98950	0.01460	-4.84670	H25	-1.02750	3.13630	-2.41940
C2	1.53330	-0.07710	-0.37940	H14	1.24950	-1.61090	-5.57420	C26	-0.83050	3.34340	0.25810
C3	1.45830	-0.50970	-1.83680	H15	-0.32770	-2.56100	-3.89960	H27	-1.14540	4.26230	-0.25580
C4	2.35760	-0.00080	-2.78360	H16	-0.16720	-1.87460	-1.50910	H28	-1.71670	2.78840	0.57680
C5	2.28230	-0.38890	-4.12460	N17	-0.02020	2.50440	-0.65540	H29	-0.23380	3.61980	1.13070
C6	1.30900	-1.30380	-4.53200	C18	1.25090	3.18620	-0.98840	C30	0.65960	1.15760	1.59600
C7	0.42190	-1.83550	-3.58970	H19	1.84900	3.29140	-0.08110	O31	1.68960	1.68840	1.98000
C8	0.50090	-1.44520	-2.25170	H20	1.80030	2.59040	-1.71900	H32	-1.21970	1.09950	4.23940
O9	1.20370	-1.03000	0.56640	H21	1.05010	4.17840	-1.41580	H33	-0.05000	2.40430	3.84970
H10	-0.59100	0.33540	-0.02520	C22	-0.79710	2.20670	-1.88090	O34	-0.41030	0.95110	2.38380
H11	2.50230	0.42720	-0.21030	H23	-0.21770	1.54770	-2.52880	C35	-0.26360	1.33480	3.76970
H12	3.13580	0.69310	-2.46710	H24	-1.73460	1.71290	-1.60800	H36	0.54450	0.76600	4.23730

3.2.7 *trans*-Epoxide

E(B3LYP/6-31G*(dichloromethane)) = -612.73501306184
E(B3LYP-D3/6-311+G**(dichloromethane)) = -612.90870908580
G_{tot}(B3LYP/6-31G*(dichloromethane)) = -612.591561

Number of imaginary frequencies: 0

C1	-0.65980	0.28280	-0.26690	C9	1.71450	0.01580	-1.32860	C17	-1.61500	-0.36010	0.70230
C2	0.79550	-0.02030	-0.15260	C10	1.80020	1.14190	-2.15870	O18	-1.38780	-0.52690	1.88580
O3	0.25110	1.22080	0.31820	C11	2.66720	1.14260	-3.25260	H19	-4.56510	-1.61350	0.20500
H4	-1.06730	0.55970	-1.23850	H12	4.13160	0.02440	-4.38030	O20	-2.73660	-0.74000	0.07390
H5	1.07830	-0.70440	0.64960	H13	3.99060	-1.97600	-2.90650	C21	-3.73700	-1.40340	0.88350
C6	3.45460	0.02070	-3.52860	H14	2.46120	-1.97600	-0.95350	H22	-3.33200	-2.33070	1.29700
C7	3.37540	-1.10260	-2.70100	H15	1.20140	2.02120	-1.93500	H23	-4.06060	-0.74610	1.69500
C8	2.51280	-1.10350	-1.60300	H16	2.73290	2.02330	-3.88800				

3.3 Reaction of amide-stabilized DABCO-ammonium ylide with benzaldehyde

3.3.1 Ylide

E(B3LYP/6-31G*(dichloromethane)) = -631.91416345312
E(B3LYP-D3/6-311+G**(dichloromethane)) = -632.09396177088
G_{tot}(B3LYP/6-31G*(dichloromethane)) = -631.657217

Number of imaginary frequencies: 0

C1	0.92970	-0.77120	-0.17770	C12	-1.85670	1.43480	0.66720	C23	4.36080	-2.42220	-0.42170
H2	0.45850	-1.64380	-0.59570	H13	-2.74490	1.79730	0.13460	C24	4.45980	-0.79770	1.34070
N3	2.40490	-0.89070	-0.10710	H14	-2.19900	0.94120	1.59670	N25	4.98790	-1.16350	0.01330
C4	2.91230	-0.66010	1.31180	H15	-1.22930	2.28400	0.93620	H26	2.40610	-1.40640	1.92790
C5	3.10150	0.08580	-1.04830	C16	-1.82480	-0.64240	-0.61120	H27	2.55040	0.33030	1.58620
H6	2.75410	1.07250	-0.74340	H17	-2.85050	-0.35480	-0.86560	H28	2.43890	-2.43720	-1.53450
H7	2.71170	-0.13780	-2.04360	H18	-1.37150	-1.08150	-1.50650	H29	2.28520	-2.97530	0.15000
C8	2.81280	-2.28940	-0.51790	H19	-1.87620	-1.42610	0.16670	H30	4.76040	-1.56680	2.06050
C9	0.30870	0.44990	0.08320	C20	4.64140	-0.09800	-0.94540	H31	4.92470	0.14430	1.64980
O10	0.88880	1.49330	0.53730	H21	5.06070	-0.35910	-1.92310	H32	4.77600	-2.70160	-1.39600
N11	-1.08950	0.54160	-0.19420	H22	5.11930	0.83150	-0.61830	H33	4.62970	-3.21060	0.28930

3.3.2 *trans*-TSadd

E(B3LYP/6-31G*(dichloromethane)) = -977.48664199669
E(B3LYP-D3/6-311+G**(dichloromethane)) = -977.75671111853
G_{tot}(B3LYP/6-31G*(dichloromethane)) = -977.125265

Number of imaginary frequencies: 1 (-141.98)

C1	0.23660	1.15140	0.08820	N17	-1.08760	0.93790	0.80140	H33	0.12210	3.01600	-3.42300
C2	1.29870	-0.57900	-0.02340	C18	-1.89850	-0.19800	0.18640	C34	2.31990	2.98750	-0.86190
C3	1.31970	-0.92930	-1.50260	H19	-1.97230	0.02370	-0.87600	H35	3.19210	3.00470	-1.52550
C4	2.26970	-0.35540	-2.35790	H20	-1.29100	-1.08900	0.34330	H36	2.22170	3.98470	-0.40840
C5	2.32760	-0.70540	-3.70870	C21	-0.78770	0.57070	2.25160	H37	2.52790	2.26230	-0.07650
C6	1.43490	-1.64740	-4.22480	H22	-0.05300	-0.23480	2.21190	C38	-3.16670	1.95660	1.76930
C7	0.49950	-2.24620	-3.37520	H23	-0.34250	1.46220	2.70330	C39	-3.27620	-0.26890	0.89590
C8	0.45090	-1.89590	-2.02540	C24	-1.94960	2.19700	0.83320	C40	-2.11270	0.15830	2.94610
O9	0.86280	-1.45310	0.80950	H25	-1.30580	3.00660	1.18560	H41	-2.18380	0.64140	3.92670
H10	0.83960	1.70510	0.80340	H26	-2.24000	2.39620	-0.19650	H42	-2.14160	-0.92450	3.10860
H11	2.23180	-0.03550	0.23940	C27	0.09090	1.84690	-1.22000	N43	-3.28530	0.53420	2.13410
H12	2.98460	0.36120	-1.95870	N28	1.13680	2.63690	-1.64100	H44	-4.08660	2.27610	1.26780
H13	3.07590	-0.25120	-4.35550	O29	-0.91410	1.69190	-1.94590	H45	-3.07060	2.54300	2.68930
H14	1.47640	-1.92300	-5.27670	C30	1.05970	3.28980	-2.94270	H46	-3.51070	-1.31020	1.14240
H15	-0.18750	-2.99470	-3.76580	H31	1.10580	4.38180	-2.82600	H47	-4.07290	0.10320	0.24210
H16	-0.25320	-2.38380	-1.35730	H32	1.89980	2.97790	-3.57740				

3.3.3 *trans-syn*-Betaine

E(B3LYP/6-31G*(dichloromethane)) = -977.48929617481
E(B3LYP-D3/6-311+G**(dichloromethane)) = -977.76339583252
G_{tot}(B3LYP/6-31G*(dichloromethane)) = -977.126618

Number of imaginary frequencies: 0

C1	0.25110	1.20290	0.09680	N17	-1.26790	0.94310	0.05030	H33	1.62910	4.19470	-2.19300
C2	1.26970	-0.13100	-0.09150	C18	-1.77410	0.39340	-1.28170	C34	2.53450	2.95830	0.81600
C3	1.17790	-0.73760	-1.51930	H19	-1.36450	1.02730	-2.06340	H35	3.57570	2.72930	0.55710
C4	1.56460	-0.03880	-2.67360	H20	-1.35760	-0.60720	-1.37480	H36	2.52000	3.89920	1.38280
C5	1.49700	-0.63560	-3.93480	C21	-1.62480	-0.07050	1.14070	H37	2.16400	2.16290	1.45910
C6	1.05260	-1.95500	-4.06670	H22	-0.89420	-0.88030	1.06150	C38	-3.54080	1.84910	0.60900
C7	0.69770	-2.67260	-2.92160	H23	-1.45230	0.44310	2.09120	C39	-3.32460	0.37800	-1.25780
C8	0.77080	-2.06890	-1.66260	C24	-2.05050	2.21810	0.36960	C40	-3.10900	-0.47900	0.96460
O9	1.15180	-0.99020	0.91000	H25	-1.58700	2.66200	1.25410	H41	-3.59070	-0.53870	1.94720
H10	0.41050	1.42560	1.15190	H26	-1.91810	2.88680	-0.47830	H42	-3.19240	-1.46740	0.49820
H11	2.23480	0.44140	-0.10320	C27	0.62890	2.40780	-0.76520	N43	-3.83570	0.49040	0.12010
H12	1.93100	0.98160	-2.59640	N28	1.74180	3.11290	-0.40370	H44	-4.18140	2.56760	0.08590
H13	1.79570	-0.07050	-4.81620	O29	-0.01130	2.70780	-1.78930	H45	-3.78930	1.89670	1.67430
H14	0.99890	-2.42070	-5.04880	C30	2.19820	4.20390	-1.26550	H46	-3.68680	-0.55370	-1.70670
H15	0.37140	-3.70770	-3.00920	H31	2.05880	5.17110	-0.76450	H47	-3.73400	1.20620	-1.84690
H16	0.52320	-2.61740	-0.75800	H32	3.26510	4.07770	-1.48560				

3.3.4 *trans*-TSrot

E(B3LYP/6-31G*(dichloromethane)) = -977.48065233927

E(B3LYP-D3/6-311+G**(dichloromethane)) = -977.75527613225

G_{tot}(B3LYP/6-31G*(dichloromethane)) = -977.118471

Number of imaginary frequencies: 0 (17.57 smallest frequency)

C1	0.26350	1.10010	0.01820	N17	-0.87440	1.54490	-0.96870	H33	1.91650	4.35450	1.70290
C2	1.51700	-0.00780	-0.31580	C18	-0.40810	2.11640	-2.30350	C34	0.98060	1.30610	2.87130
C3	1.40680	-0.72940	-1.67780	H19	0.33140	2.87930	-2.07450	H35	1.78520	1.31150	3.61460
C4	1.99070	-0.19850	-2.83750	H20	0.07200	1.29630	-2.83330	H36	0.02770	1.46590	3.39610
C5	1.95530	-0.89510	-4.04900	C21	-1.76150	0.34110	-1.25800	H37	1.01080	0.34080	2.35930
C6	1.34020	-2.14830	-4.11490	H22	-1.11730	-0.46530	-1.60240	C38	-3.04620	2.81130	-1.15060
C7	0.78380	-2.70330	-2.95760	H23	-2.21010	0.04910	-0.30440	C39	-1.64010	2.66560	-3.07650
C8	0.83180	-2.00410	-1.74920	C24	-1.76760	2.58950	-0.29390	C40	-2.83200	0.73590	-2.31400
O9	1.59830	-0.82610	0.72600	H25	-2.00040	2.21220	0.70490	H41	-3.81110	0.35730	-2.00200
H10	-0.30870	0.55240	0.76480	H26	-1.17030	3.49580	-0.20700	H42	-2.60120	0.29010	-3.28730
H11	2.37820	0.69670	-0.44760	C27	0.93750	2.35460	0.58980	N43	-2.90410	2.19710	-2.47990
H12	2.49090	0.76890	-2.78800	N28	1.24260	2.38370	1.91180	H44	-3.22670	3.88520	-1.26720
H13	2.41580	-0.46490	-4.93700	O29	1.23030	3.30060	-0.16770	H45	-3.92340	2.37750	-0.65950
H14	1.31120	-2.69460	-5.05560	C30	1.87030	3.58160	2.46810	H46	-1.59300	2.34020	-4.12150
H15	0.32420	-3.68920	-2.99610	H31	1.28160	3.94280	3.32060	H47	-1.64470	3.76080	-3.07380
H16	0.44520	-2.43940	-0.83120	H32	2.88520	3.35450	2.81780				

3.3.5 *trans-anti*-Betaine

E(B3LYP/6-31G*(dichloromethane)) = -977.48740415456

E(B3LYP-D3/6-311+G**(dichloromethane)) = -977.76298955123

G_{tot}(B3LYP/6-31G*(dichloromethane)) = -977.124725

Number of imaginary frequencies: 0

C1	0.35730	0.93340	0.13850	H13	2.73580	-0.17480	-4.88930	H25	-1.74210	2.55510	0.52450
C2	1.59480	-0.00010	-0.29460	H14	0.97360	-1.86420	-5.38220	H26	-0.33400	3.49100	1.08030
C3	1.44080	-0.51480	-1.74010	H15	-0.45040	-2.73080	-3.53100	C27	0.58170	1.24510	1.63080
C4	2.25460	-0.06070	-2.78650	H16	-0.11240	-1.90090	-1.21040	N28	-0.26440	0.67440	2.52740
C5	2.09210	-0.54160	-4.09170	N17	0.02810	2.28310	-0.61850	O29	1.44910	2.07100	1.96280
C6	1.10900	-1.49290	-4.36820	C18	1.27960	3.08180	-0.95160	C30	-0.15580	1.03880	3.93830
C7	0.30860	-1.97760	-3.32610	H19	1.84530	3.17310	-0.02560	H31	-1.16010	1.11200	4.36990
C8	0.48530	-1.50250	-2.02700	H20	1.84900	2.47960	-1.65910	H32	0.41530	0.28870	4.50090
O9	1.63200	-0.98250	0.62560	C21	-0.71100	1.97900	-1.91650	H33	0.34700	2.00150	4.02210
H10	-0.55300	0.35630	-0.01800	H22	-0.13720	1.22810	-2.45470	C34	-1.09200	-0.49790	2.21480
H11	2.48490	0.67820	-0.29650	H23	-1.67420	1.54710	-1.63080	H35	-1.29410	-1.03610	3.14470
H12	3.03500	0.67140	-2.57880	C24	-0.90050	3.17480	0.20570	H36	-2.05760	-0.21390	1.77380

H37	-0.53710	-1.15860	1.54530	H41	-1.90550	3.36910	-3.10060	H45	-2.41500	4.29910	-0.92340
C38	-1.35550	4.38570	-0.66060	H42	-0.21230	3.29990	-3.59900	H46	1.44990	4.65010	-2.46040
C39	0.86110	4.45160	-1.55840	N43	-0.57180	4.47270	-1.90270	H47	1.05750	5.26360	-0.85060
C40	-0.87790	3.29440	-2.72950	H44	-1.22910	5.31160	-0.08960				

3.3.6 *trans*-TSelim

E(B3LYP/6-31G*(dichloromethane)) = -977.47691193718
E(B3LYP-D3/6-311+G**(dichloromethane)) = -977.74785743233
G_{tot}(B3LYP/6-31G*(dichloromethane)) = -977.117415

Number of imaginary frequencies: 1 (-279.93)

C1	0.42030	0.70280	0.19710	N17	-0.08550	2.42630	-0.65290	H33	0.41280	2.33890	3.85370
C2	1.58170	-0.07530	-0.37030	C18	1.13380	3.19550	-1.04350	C34	-1.26000	-0.22610	2.39940
C3	1.47960	-0.50150	-1.83050	H19	1.73100	3.33510	-0.14090	H35	-1.34490	-0.76450	3.34950
C4	2.31230	0.05030	-2.81260	H20	1.70040	2.57270	-1.73820	H36	-2.25350	0.16330	2.13730
C5	2.19540	-0.33660	-4.15140	C21	-0.88090	2.10710	-1.87710	H37	-0.92170	-0.93730	1.64700
C6	1.24650	-1.29260	-4.52200	H22	-0.29490	1.41940	-2.48850	C38	-1.21110	3.43280	-2.63620
C7	0.42760	-1.86880	-3.54460	H23	-1.78990	1.58840	-1.55570	C39	-1.38890	4.54920	-0.52680
C8	0.55030	-1.48040	-2.20980	C24	-0.92480	3.27380	0.24890	H40	-2.47740	4.55370	-0.65180
O9	1.32810	-1.06370	0.55820	H25	-1.77450	2.67180	0.58420	H41	-1.11460	5.45240	0.02920
H10	-0.53910	0.26210	-0.04230	H26	-0.31500	3.53230	1.11660	C42	0.69990	4.54450	-1.70120
H11	2.53360	0.46910	-0.22420	C27	0.66090	1.16770	1.62040	N43	-0.76630	4.60580	-1.86170
H12	3.07050	0.77880	-2.52680	N28	-0.27610	0.84820	2.55600	H44	1.16670	4.65930	-2.68610
H13	2.85070	0.10140	-4.90170	O29	1.63680	1.89080	1.87750	H45	1.01450	5.39510	-1.08670
H14	1.15380	-1.59690	-5.56260	C30	-0.14700	1.40490	3.90020	H46	-2.28940	3.51490	-2.81240
H15	-0.30220	-2.62620	-3.82500	H31	-1.14620	1.59510	4.30720	H47	-0.71420	3.45480	-3.61240
H16	-0.06110	-1.94330	-1.43920	H32	0.37830	0.71090	4.57090				

3.3.7 DABCO

E(B3LYP/6-31G*(dichloromethane)) = -345.33813022504
E(B3LYP-D3/6-311+G**(dichloromethane)) = -345.43101016758
G_{tot}(B3LYP/6-31G*(dichloromethane)) = -345.183863

Number of imaginary frequencies: 0

N1	-0.44810	1.04510	1.45340	C8	-0.89550	0.55780	2.77630	C15	-1.91210	3.04500	1.76290
C2	-0.46800	2.52470	1.45990	H9	-0.86170	-0.53870	2.76890	N16	-2.83590	1.90580	1.96130
H3	0.24910	2.87570	2.21230	H10	-0.17670	0.90000	3.53100	H17	-2.29780	3.65860	0.93980
H4	-0.11360	2.87830	0.48390	C11	-2.83880	1.08060	0.73180	H18	-1.93240	3.66420	2.66810
C5	-1.39510	0.56210	0.42440	C12	-2.33760	1.08140	3.08450	H19	-3.53920	0.24970	0.88000
H6	-1.04600	0.91110	-0.55530	H13	-2.35320	1.69420	3.99430	H20	-3.22830	1.69330	-0.09020
H7	-1.35880	-0.53430	0.40990	H14	-3.03830	0.25200	3.23970				

3.4 Reaction of amide-stabilized quinculidine-ammonium ylide with benzaldehyde

3.4.1 Ylide

E(B3LYP/6-31G*(dichloromethane)) = -615.89342036117
E(B3LYP-D3/6-311+G**(dichloromethane)) = -616.06796267162
G_{tot}(B3LYP/6-31G*(dichloromethane)) = -615.626106

Number of imaginary frequencies: 0

C1	0.93890	-0.75790	-0.19010	N3	2.41320	-0.88510	-0.10990	C5	3.11380	0.09260	-1.05200
H2	0.46470	-1.63470	-0.59580	C4	2.90580	-0.65480	1.31780	H6	2.75710	1.07600	-0.74690

H7	2.71380	-0.13230	-2.04270	H17	-2.84710	-0.37200	-0.86140	H27	2.42240	-2.42990	-1.53480
C8	2.80420	-2.29190	-0.51990	H18	-1.36460	-1.08990	-1.50360	H28	2.25520	-2.96440	0.14420
C9	0.31270	0.45890	0.07160	H19	-1.85880	-1.42840	0.17460	H29	4.71230	-1.54880	2.14830
O10	0.88610	1.50820	0.52260	C20	4.65150	-0.06210	-0.97680	H30	4.88960	0.15740	1.73770
N11	-1.08920	0.54410	-0.20320	H21	5.05280	-0.30630	-1.96720	H31	4.73130	-2.78830	-1.40810
C12	-1.85510	1.42050	0.67630	H22	5.11290	0.88220	-0.66530	H32	4.57230	-3.29270	0.27340
H13	-2.76810	1.75230	0.16750	C23	4.33730	-2.48690	-0.43110	C33	5.00200	-1.17510	0.02550
H14	-2.15530	0.92410	1.61800	C24	4.44470	-0.78800	1.40610	H34	6.08790	-1.30600	0.07910
H15	-1.24550	2.28840	0.92360	H25	2.38080	-1.39820	1.92080				
C16	-1.81760	-0.64900	-0.60910	H26	2.53330	0.33390	1.58310				

3.4.2 *trans*-TSadd

E(B3LYP/6-31G*(dichloromethane)) = -961.46604949745
E(B3LYP-D3/6-311+G**(dichloromethane)) = -961.73067072835
G_{tot}(B3LYP/6-31G*(dichloromethane)) = -961.093332

Number of imaginary frequencies: 1 (-141.02)

C1	0.23660	1.18620	0.09990	N17	-1.08880	0.95280	0.79820	H33	0.18880	3.01080	-3.42560
C2	1.35080	-0.59030	-0.01700	C18	-1.87070	-0.19830	0.16910	C34	2.32580	3.02630	-0.81380
C3	1.31670	-0.95220	-1.48770	H19	-1.88820	0.00500	-0.89910	H35	3.20620	3.08960	-1.46470
C4	2.21710	-0.36130	-2.38470	H20	-1.26320	-1.08100	0.36690	H36	2.19310	4.00710	-0.33100
C5	2.23170	-0.72510	-3.73260	C21	-0.79200	0.58720	2.25190	H37	2.54460	2.28340	-0.04750
C6	1.34440	-1.69550	-4.20390	H22	-0.01570	-0.17730	2.21320	C38	-3.17310	2.00810	1.76490
C7	0.45800	-2.30900	-3.31290	H23	-0.38980	1.49620	2.70930	C39	-3.27920	-0.28950	0.79620
C8	0.45330	-1.94680	-1.96570	C24	-1.96770	2.20490	0.81740	C40	-2.08090	0.10350	2.95250
O9	0.94280	-1.43770	0.84560	H25	-1.31940	3.02590	1.13330	H41	-2.13540	0.54530	3.95370
H10	0.84080	1.72450	0.82500	H26	-2.26550	2.37470	-0.21540	H42	-2.05960	-0.98540	3.07940
H11	2.26760	-0.00880	0.21260	C27	0.10850	1.87690	-1.20490	H43	-4.08230	2.37120	1.27250
H12	2.92630	0.37900	-2.02000	N28	1.16550	2.66180	-1.61910	H44	-3.04330	2.59670	2.68060
H13	2.94220	-0.25900	-4.41300	O29	-0.88680	1.72690	-1.94800	H45	-3.52180	-1.34190	0.98250
H14	1.35200	-1.98090	-5.25410	C30	1.09340	3.33040	-2.91160	H46	-4.03690	0.09990	0.10510
H15	-0.22360	-3.07930	-3.66940	H31	1.07110	4.42250	-2.78250	C47	-3.30180	0.51370	2.10920
H16	-0.20950	-2.44610	-1.26450	H32	1.97010	3.07690	-3.52200	H48	-4.22980	0.32740	2.65940

3.4.3 *trans-syn*-Betaine

E(B3LYP/6-31G*(dichloromethane)) = -961.46946182732
E(B3LYP-D3/6-311+G**(dichloromethane)) = -961.73833460194
G_{tot}(B3LYP/6-31G*(dichloromethane)) = -961.096049

Number of imaginary frequencies: 0

C1	0.25850	1.21140	0.09310	N17	-1.25970	0.95240	0.04210	H33	1.60780	4.27660	-2.12000
C2	1.28410	-0.11520	-0.09450	C18	-1.76230	0.41170	-1.29990	C34	2.50220	2.97990	0.86880
C3	1.17370	-0.74480	-1.51150	H19	-1.31490	1.03150	-2.07190	H35	3.57170	2.90840	0.63860
C4	1.52940	-0.06080	-2.68490	H20	-1.36460	-0.59760	-1.38010	H36	2.34790	3.85970	1.50870
C5	1.44720	-0.68010	-3.93500	C21	-1.61320	-0.06990	1.13180	H37	2.22290	2.08850	1.42550
C6	1.01880	-2.00680	-4.03760	H22	-0.85860	-0.85800	1.06650	C38	-3.51650	1.90940	0.65510
C7	0.69140	-2.70850	-2.87360	H23	-1.46050	0.45350	2.08140	C39	-3.30590	0.42450	-1.33240
C8	0.77870	-2.08280	-1.62570	C24	-2.03350	2.23540	0.36620	C40	-3.07590	-0.53020	0.96110
O9	1.19330	-0.95960	0.92220	H25	-1.53590	2.69060	1.22640	H41	-3.53310	-0.63340	1.95240
H10	0.41620	1.43330	1.14840	H26	-1.91380	2.88870	-0.49590	H42	-3.11950	-1.51700	0.48190
H11	2.24490	0.46190	-0.13340	C27	0.64360	2.42060	-0.76060	H43	-4.14610	2.66490	0.17170
H12	1.88020	0.96620	-2.63220	N28	1.74840	3.12740	-0.37610	H44	-3.72020	1.95490	1.73150
H13	1.72070	-0.12620	-4.83140	O29	0.02270	2.71990	-1.79640	H45	-3.65670	-0.48820	-1.82920
H14	0.95490	-2.48920	-5.01140	C30	2.20160	4.24140	-1.20900	H46	-3.67390	1.27430	-1.92150
H15	0.37510	-3.74820	-2.93750	H31	2.09290	5.19070	-0.66740	C47	-3.83590	0.50490	0.10970
H16	0.55050	-2.61850	-0.70770	H32	3.25990	4.10690	-1.46400	H48	-4.91400	0.31670	0.13860

3.4.4 *trans*-TSrot

E(B3LYP/6-31G*(dichloromethane)) = -961.46116309696
E(B3LYP-D3/6-311+G**(dichloromethane)) = -961.73086607998
G_{tot}(B3LYP/6-31G*(dichloromethane)) = -961.087137

Number of imaginary frequencies: 0 (18.72 smallest frequency)

C1	0.28210	1.09180	0.01050	N17	-0.85880	1.53640	-0.96800	H33	1.91510	4.34820	1.71380
C2	1.54210	0.00310	-0.33660	C18	-0.39020	2.08950	-2.31370	C34	0.94540	1.30250	2.86800
C3	1.41970	-0.73010	-1.69180	H19	0.39620	2.80590	-2.09150	H35	1.71090	1.33180	3.65120
C4	1.99450	-0.21210	-2.86140	H20	0.03670	1.24420	-2.84900	H36	-0.03640	1.43960	3.34490
C5	1.94300	-0.91820	-4.06690	C21	-1.76020	0.33300	-1.23290	H37	1.02200	0.33730	2.36050
C6	1.32100	-2.16840	-4.11720	H22	-1.11480	-0.49500	-1.51770	C38	-3.05240	2.80510	-1.07070
C7	0.77410	-2.71150	-2.94990	H23	-2.23150	0.09110	-0.27620	C39	-1.58190	2.70980	-3.08010
C8	0.83800	-2.00280	-1.74790	C24	-1.73260	2.60140	-0.29130	C40	-2.80150	0.66790	-2.32530
O9	1.65590	-0.80720	0.71050	H25	-1.90730	2.25890	0.73150	H41	-3.76850	0.24040	-2.03830
H10	-0.28210	0.54000	0.76010	H26	-1.13100	3.50870	-0.26260	H42	-2.51730	0.21140	-3.28070
H11	2.39120	0.71770	-0.48930	C27	0.95460	2.34610	0.58650	H43	-3.26590	3.87790	-1.13290
H12	2.49600	0.75490	-2.82570	N28	1.22940	2.38150	1.91530	H44	-3.89010	2.33630	-0.54150
H13	2.39530	-0.49660	-4.96300	O29	1.27000	3.28520	-0.16980	H45	-1.50590	2.43340	-4.13780
H14	1.27850	-2.72160	-5.05340	C30	1.84940	3.57870	2.48130	H46	-1.54580	3.80470	-3.03010
H15	0.30810	-3.69490	-2.97590	H31	1.24280	3.94580	3.31860	C47	-2.89980	2.19580	-2.47460
H16	0.45900	-2.43010	-0.82300	H32	2.85570	3.34990	2.85460	H48	-3.75040	2.46820	-3.10760

3.4.5 *trans-anti*-Betaine

E(B3LYP/6-31G*(dichloromethane)) = -961.46779859104
E(B3LYP-D3/6-311+G**(dichloromethane)) = -961.73812759053
G_{tot}(B3LYP/6-31G*(dichloromethane)) = -961.093165

Number of imaginary frequencies: 0

C1	0.35420	0.93640	0.13930	N17	0.03020	2.28300	-0.61700	H33	0.32000	2.04080	4.01320
C2	1.58620	-0.00560	-0.29560	C18	1.29090	3.07040	-0.95790	C34	-1.08570	-0.49350	2.23010
C3	1.43290	-0.51470	-1.74440	H19	1.88110	3.10960	-0.04410	H35	-1.25530	-1.04270	3.16060
C4	2.24590	-0.05520	-2.78970	H20	1.82440	2.47370	-1.69730	H36	-2.06650	-0.21620	1.81800
C5	2.08210	-0.52800	-4.09800	C21	-0.72180	1.97250	-1.90990	H37	-0.54120	-1.14210	1.54080
C6	1.09830	-1.47770	-4.38040	H22	-0.18540	1.17180	-2.41270	C38	-1.41650	4.36160	-0.63610
C7	0.30010	-1.96950	-3.33920	H23	-1.70110	1.59320	-1.60400	C39	0.91930	4.46840	-1.50630
C8	0.47860	-1.50250	-2.03680	C24	-0.88860	3.18240	0.21600	C40	-0.84010	3.24190	-2.78410
O9	1.61780	-0.99240	0.62110	H25	-1.69680	2.54910	0.58920	H41	-1.84560	3.28460	-3.21720
H10	-0.55890	0.36140	-0.01090	H26	-0.29210	3.52650	1.05980	H42	-0.13020	3.20070	-3.61860
H11	2.48010	0.66880	-0.29500	C27	0.58510	1.24740	1.63110	H43	-1.35850	5.28190	-0.04440
H12	3.02620	0.67640	-2.57860	N28	-0.26210	0.68370	2.53190	H44	-2.47020	4.20850	-0.89660
H13	2.72530	-0.15530	-4.89350	O29	1.45620	2.06950	1.96200	H45	1.55780	4.69550	-2.36740
H14	0.96130	-1.84210	-5.39670	C30	-0.15800	1.06420	3.93920	H46	1.10960	5.23960	-0.75060
H15	-0.45920	-2.72190	-3.54730	H31	-1.16350	1.11520	4.37230	C47	-0.56520	4.47860	-1.91180
H16	-0.11810	-1.90550	-1.22150	H32	0.43380	0.33510	4.50950	H48	-0.81050	5.39670	-2.45500

3.4.6 *trans*-TSelim

E(B3LYP/6-31G*(dichloromethane)) = -961.45552034304
E(B3LYP-D3/6-311+G**(dichloromethane)) = -961.72076455837
G_{tot}(B3LYP/6-31G*(dichloromethane)) = -961.084685

Number of imaginary frequencies: 1 (-289.81)

C1	0.43620	0.67400	0.20420	C6	1.23600	-1.27730	-4.53340	H11	2.54660	0.43830	-0.22210
C2	1.59160	-0.09750	-0.37370	C7	0.43310	-1.87320	-3.55510	H12	3.05870	0.78780	-2.53170
C3	1.48700	-0.51580	-1.83520	C8	0.56400	-1.49880	-2.21700	H13	2.82150	0.13740	-4.91230
C4	2.30580	0.05460	-2.81840	O9	1.32340	-1.08440	0.55560	H14	1.13540	-1.56890	-5.57690
C5	2.17910	-0.31700	-4.16050	H10	-0.52740	0.24500	-0.03660	H15	-0.29190	-2.63430	-3.83730

H16	-0.03760	-1.97380	-1.44600	C27	0.68290	1.15460	1.61970	C38	-1.19480	3.38290	-2.68970
N17	-0.08280	2.42530	-0.65380	N28	-0.26010	0.85990	2.55900	C39	-1.45480	4.52310	-0.48250
C18	1.14080	3.19320	-1.03410	O29	1.67170	1.86210	1.87070	H40	-2.54340	4.47050	-0.60480
H19	1.74760	3.29320	-0.13310	C30	-0.13320	1.43460	3.89580	H41	-1.23900	5.41950	0.11030
H20	1.69230	2.57580	-1.74600	H31	-1.12770	1.70230	4.27040	C42	0.75520	4.56710	-1.65400
C21	-0.87250	2.09450	-1.87860	H32	0.32210	0.71850	4.59400	H43	1.27320	4.70690	-2.61010
H22	-0.29240	1.38000	-2.46420	H33	0.49150	2.32600	3.84760	H44	1.06740	5.38680	-0.99580
H23	-1.78670	1.59110	-1.54730	C34	-1.26790	-0.19190	2.40970	H45	-2.26620	3.42680	-2.91730
C24	-0.92700	3.25880	0.25660	H35	-1.37490	-0.71090	3.36820	H46	-0.66210	3.37500	-3.64830
H25	-1.74810	2.63180	0.61630	H36	-2.24900	0.21610	2.13130	C47	-0.77130	4.60920	-1.85960
H26	-0.30420	3.52950	1.11140	H37	-0.94070	-0.92410	1.67180	H48	-1.06150	5.53330	-2.37130

3.4.7 Quinuclidine

E(B3LYP/6-31G*(dichloromethane)) = -329.31494318211
E(B3LYP-D3/6-311+G**(dichloromethane)) = -329.40225494037
G_{tot}(B3LYP/6-31G*(dichloromethane)) = -329.149446

Number of imaginary frequencies: 0

N1	-0.45540	1.04790	1.45420	C8	-0.89430	0.55660	2.77810	C15	-1.88890	3.09910	1.75830
C2	-0.46550	2.52650	1.45920	H9	-0.84840	-0.53900	2.76420	H16	-2.24290	3.72700	0.93080
H3	0.25990	2.86640	2.20820	H10	-0.16480	0.89620	3.52320	H17	-1.87900	3.73140	2.65510
H4	-0.10080	2.87050	0.48380	C11	-2.85550	1.05070	0.68390	H18	-3.53990	0.20180	0.80690
C5	-1.39450	0.56080	0.42130	C12	-2.33280	1.05230	3.13640	H19	-3.23080	1.64120	-0.16150
H6	-1.03280	0.90770	-0.55420	H13	-2.32650	1.64550	4.05960	C20	-2.84890	1.91040	1.96390
H7	-1.34380	-0.53460	0.40840	H14	-3.00990	0.20580	3.30750	H21	-3.85990	2.27460	2.17900

3.5 Reaction of α-methyl amide-stabilized trimethylammonium ylide with benzaldehyde

3.5.1 Ylide

E(B3LYP/6-31G*(dichloromethane)) = -500.35323417075
E(B3LYP-D3/6-311+G**(dichloromethane)) = -500.50190691769
G_{tot}(B3LYP/6-31G*(dichloromethane)) = -500.128770

Number of imaginary frequencies: 0

C1	0.93330	-0.90240	-0.16780	C11	-1.86630	1.26750	0.85280	H21	2.59940	-2.26940	-1.79540
N2	2.44830	-0.82070	-0.24590	H12	-1.82660	2.22480	0.30540	H22	2.85220	-2.92080	-0.14910
C3	3.03460	-0.59120	1.12970	H13	-2.92140	0.99800	0.99180	C23	0.34640	-2.28470	-0.05490
C4	2.87980	0.30190	-1.16180	H14	-1.41430	1.42670	1.83420	H24	-0.69200	-2.17470	0.26490
H5	2.43130	1.21930	-0.78830	C15	-1.81520	-0.09240	-1.13690	H25	0.34070	-2.87890	-0.98280
H6	2.51130	0.07280	-2.16280	H16	-1.81630	0.79510	-1.79810	H26	0.83700	-2.91000	0.71060
C7	3.04300	-2.08080	-0.81590	H17	-1.29240	-0.89670	-1.65550	H27	3.97190	0.35880	-1.15880
C8	0.25680	0.27440	0.16970	H18	-2.86000	-0.39840	-0.99260	H28	4.12620	-0.53290	1.06550
O9	0.77640	1.39200	0.49720	H19	2.74030	-1.42780	1.76710	H29	4.12120	-1.93410	-0.91570
N10	-1.19190	0.17620	0.16010	H20	2.60930	0.34130	1.49920				

3.5.2 *trans*-TSadd

E(B3LYP/6-31G*(dichloromethane)) = -845.91764538669
E(B3LYP-D3/6-311+G**(dichloromethane)) = -846.15928244193
G_{tot}(B3LYP/6-31G*(dichloromethane)) = -845.588422

Number of imaginary frequencies: 1 (-153.56)

C1	0.34800	1.32990	0.09710	C2	1.57420	-0.27300	-0.11970	C3	1.27720	-0.94390	-1.45790

C4	1.53120	-0.31070	-2.68340	H18	-1.15670	-1.03260	-0.51620	H32	3.04980	3.90150	-0.51170
C5	1.31820	-0.97420	-3.89320	H19	-0.32280	-0.65860	1.71080	H33	2.99800	2.12550	-0.42620
C6	0.85640	-2.29340	-3.89890	H20	-0.94760	0.77480	2.59190	C34	-2.08990	2.00960	0.61910
C7	0.62620	-2.94470	-2.68360	H21	-1.67930	2.73010	1.32690	H35	-3.02540	1.60580	1.01570
C8	0.84460	-2.27680	-1.47730	H22	-2.24850	2.47380	-0.35000	C36	-1.11490	0.08870	1.76240
O9	1.55260	-0.98830	0.94520	C23	0.30520	2.21080	-1.13040	H37	-2.09910	-0.37280	1.87120
H10	2.44450	0.40140	-0.24650	N24	1.37170	3.02580	-1.47390	C38	-1.69820	-0.09290	-0.57230
H11	1.90660	0.70910	-2.69730	O25	-0.69380	2.24360	-1.88020	H39	-2.74920	-0.25410	-0.31940
H12	1.51870	-0.46290	-4.83290	C26	1.15790	3.92260	-2.61350	C40	0.95690	1.95090	1.35310
H13	0.69120	-2.81190	-4.84130	H27	1.84990	4.76640	-2.52400	H41	1.81060	2.57890	1.10730
H14	0.28650	-3.97890	-2.67690	H28	1.34550	3.41880	-3.57280	H42	0.26700	2.59620	1.91120
H15	0.69720	-2.77990	-0.52540	H29	0.13370	4.29470	-2.61650	H43	1.31440	1.15810	2.01970
N16	-1.11890	0.85590	0.45390	C30	2.77340	2.98470	-1.05100				
H17	-1.60410	0.35520	-1.55520	H31	3.41400	2.91010	-1.93990				

3.5.3 *trans-syn*-Betaine

E(B3LYP/6-31G*(dichloromethane)) = -845.92089371793
E(B3LYP-D3/6-311+G**(dichloromethane)) = -846.16343766771
G_{tot}(B3LYP/6-31G*(dichloromethane)) = -845.589574

Number of imaginary frequencies: 0

C1	-0.04140	1.08400	0.24940	N16	0.17800	2.52610	-0.42420	H31	-0.25950	-1.69330	3.17400
C2	1.01200	-0.09960	-0.43040	H17	1.98670	2.90510	0.63700	H32	-1.88110	-1.17510	2.65930
C3	2.47530	0.01440	0.08160	H18	2.19830	2.37300	-1.06160	H33	-0.57540	-1.41940	1.46890
C4	2.83670	-0.19230	1.42180	H19	0.28270	1.58640	-2.31380	C34	-1.44680	0.62180	-0.16650
C5	4.17360	-0.15510	1.82640	H20	-1.27800	2.43050	-1.99870	H35	-2.21400	1.39530	-0.06970
C6	5.18460	0.07520	0.89020	H21	-1.71060	3.29170	0.25290	H36	-1.40240	0.26740	-1.19860
C7	4.84250	0.24630	-0.45410	H22	-0.29290	3.84220	1.19340	H37	-1.77800	-0.21350	0.44590
C8	3.50350	0.20310	-0.84950	C23	0.12410	1.25480	1.78300	C38	-0.19390	2.47710	-1.89830
O9	0.88240	-0.21430	-1.74330	N24	-0.38970	0.33610	2.66930	H39	0.17060	3.40200	-2.35190
H10	0.60310	-0.97410	0.13640	O25	0.70700	2.24590	2.25340	C40	1.61850	2.98800	-0.37980
H11	2.07350	-0.40240	2.16600	C26	-0.28410	0.69230	4.09050	H41	1.63850	4.02940	-0.71080
H12	4.42580	-0.31570	2.87330	H27	-1.03270	0.11820	4.64450	C42	-0.67140	3.61620	0.20150
H13	6.22650	0.10300	1.20360	H28	0.71040	0.45810	4.49500	H43	-0.60200	4.49230	-0.44810
H14	5.62230	0.40270	-1.19740	H29	-0.46920	1.75760	4.22870				
H15	3.22150	0.30170	-1.89420	C30	-0.80630	-1.05650	2.46680				

3.5.4 *trans*-TSrot

E(B3LYP/6-31G*(dichloromethane)) = -845.90930834617
E(B3LYP-D3/6-311+G**(dichloromethane)) = -846.15463088204
G_{tot}(B3LYP/6-31G*(dichloromethane)) = -845.577497

Number of imaginary frequencies: 1 (-26.42)

C1	-0.05630	1.11300	0.11330	N16	0.13770	2.59240	-0.59370	H31	-0.49920	-1.44560	3.25960
C2	0.99400	0.00880	-0.46040	H17	1.76140	3.35790	0.56310	H32	-1.95740	-0.95960	2.36800
C3	0.29530	-1.00310	-1.44520	H18	2.25680	2.40680	-0.85980	H33	-0.39660	-1.28570	1.51430
C4	-0.02590	-0.73600	-2.78540	H19	0.64660	2.01050	-2.57640	C34	-1.54230	0.75520	-0.08600
C5	-0.64250	-1.69440	-3.59690	H20	-1.11840	2.13850	-2.28790	H35	-2.20730	1.35480	0.54240
C6	-0.94620	-2.95830	-3.08790	H21	-1.83310	3.36230	-0.25360	H36	-1.86590	0.83920	-1.12420
C7	-0.59100	-3.25930	-1.77050	H22	-0.66770	3.63470	1.08580	H37	-1.69660	-0.28890	0.17700
C8	0.03410	-2.29750	-0.97390	C23	0.24470	1.28690	1.65780	C38	-0.14190	2.56720	-2.07580
O9	1.56940	-0.61740	0.57790	N24	-0.44140	0.52310	2.56630	H39	-0.12620	3.60080	-2.43030
H10	1.69660	0.58180	-1.11900	O25	0.92890	2.23310	2.07210	C40	1.55290	3.14390	-0.47670
H11	0.21530	0.22260	-3.23180	C26	-0.39720	0.95930	3.96340	H41	1.60010	4.04640	-1.09040
H12	-0.88110	-1.45070	-4.63110	H27	-1.28570	0.56830	4.47040	C42	-0.80480	3.61350	0.00730
H13	-1.43340	-3.70280	-3.71470	H28	0.49610	0.58410	4.48140	H43	-0.55760	4.59090	-0.41530
H14	-0.79220	-4.24990	-1.36610	H29	-0.40070	2.04780	4.01880				
H15	0.36660	-2.53740	0.03080	C30	-0.86380	-0.86840	2.40130				

3.5.5 *trans-anti*-Betaine

E(B3LYP/6-31G*(dichloromethane)) = -845.91891640476
E(B3LYP-D3/6-311+G**(dichloromethane)) = -846.16402854949
G_{tot}(B3LYP/6-31G*(dichloromethane)) = -845.587174

Number of imaginary frequencies: 0

C1	0.16090	0.86790	0.10150	N16	0.06700	2.36140	-0.63320	H31	-0.61940	-1.17170	3.62210
C2	1.41820	-0.07610	-0.32220	H17	1.95660	2.98950	0.11030	H32	-1.92010	-0.76810	2.48170
C3	1.36330	-0.57670	-1.78420	H18	1.95990	2.47010	-1.60550	H33	-0.30400	-1.33090	1.88870
C4	2.16010	-0.01390	-2.79240	H19	-0.08670	1.47990	-2.57260	C34	-1.18320	0.19410	-0.17160
C5	2.12410	-0.49460	-4.10640	H20	-1.63570	2.10320	-1.91920	H35	-2.02510	0.69010	0.32340
C6	1.28760	-1.56150	-4.43740	H21	-1.74550	2.84840	0.39590	H36	-1.39430	0.12330	-1.23850
C7	0.51820	-2.16180	-3.43430	H22	-0.26110	3.58100	1.08470	H37	-1.13450	-0.82850	0.19140
C8	0.57190	-1.68510	-2.12400	C23	0.45650	1.17130	1.61350	C38	-0.56350	2.27600	-2.00430
O9	1.44580	-1.07210	0.58500	N24	-0.30600	0.63200	2.61650	H39	-0.39820	3.23450	-2.50340
H10	2.29420	0.61280	-0.27170	O25	1.31190	2.02650	1.88920	C40	1.41920	3.01030	-0.83180
H11	2.84350	0.79730	-2.54650	C26	-0.14000	1.22570	3.94650	H41	1.24710	4.03950	-1.16100
H12	2.75690	-0.04010	-4.86660	H27	-1.03930	1.00870	4.53320	C42	-0.78620	3.31610	0.17020
H13	1.25060	-1.93620	-5.45820	H28	0.73400	0.81540	4.47330	H43	-0.95460	4.21240	-0.43360
H14	-0.11710	-3.01220	-3.67450	H29	-0.01650	2.30630	3.86170				
H15	0.01530	-2.18590	-1.33800	C30	-0.83390	-0.74130	2.63710				

3.5.6 *trans*-TSelim

E(B3LYP/6-31G*(dichloromethane)) = -845.91504906478
E(B3LYP-D3/6-311+G**(dichloromethane)) = -846.15697210474
G_{tot}(B3LYP/6-31G*(dichloromethane)) = -845.585918

Number of imaginary frequencies: 1 (-236.90)

C1	0.16260	0.82480	0.07630	N16	-0.03160	2.64940	-0.72680	H31	-0.94420	-0.83910	3.62770
C2	1.38150	0.02220	-0.42500	H17	1.85400	3.23210	0.01680	H32	-2.15440	-0.33260	2.43170
C3	1.33050	-0.48980	-1.87040	H18	1.85250	2.70740	-1.68840	H33	-0.62230	-1.12350	1.91500
C4	2.04550	0.14230	-2.89830	H19	-0.20970	1.85110	-2.68440	C34	-1.20260	0.27410	-0.28390
C5	1.99760	-0.33330	-4.21250	H20	-1.76180	2.31170	-1.92690	H35	-2.01500	0.83650	0.18510
C6	1.23840	-1.46510	-4.51960	H21	-1.79720	3.04380	0.38960	H36	-1.34920	0.26840	-1.36450
C7	0.55070	-2.12660	-3.49720	H22	-0.28730	3.68410	1.10240	H37	-1.28050	-0.76140	0.04080
C8	0.60720	-1.64850	-2.18650	C23	0.46980	1.22740	1.53820	C38	-0.71200	2.58230	-2.04920
O9	1.27750	-0.94850	0.53830	N24	-0.38180	0.86320	2.55010	H39	-0.66180	3.56870	-2.52840
H10	2.28360	0.66170	-0.33690	O25	1.41760	1.99440	1.76540	C40	1.31060	3.26380	-0.92440
H11	2.67100	1.00370	-2.66920	C26	-0.18020	1.51260	3.84720	H41	1.18610	4.30070	-1.26360
H12	2.56260	0.17370	-4.99230	H27	-1.11750	1.45940	4.41060	C42	-0.83570	3.51670	0.17610
H13	1.19820	-1.83880	-5.54070	H28	0.61200	1.02390	4.43230	H43	-1.01870	4.48380	-0.31100
H14	-0.02430	-3.02310	-3.72160	H29	0.09310	2.55810	3.70160				
H15	0.10770	-2.18180	-1.38310	C30	-1.07770	-0.42970	2.62000				

3.5.7 *trans*-Epoxide

E(B3LYP/6-31G*(dichloromethane)) = -671.49586916648
E(B3LYP-D3/6-311+G**(dichloromethane)) = -671.68247624393
G_{tot}(B3LYP/6-31G*(dichloromethane)) = -671.287795

Number of imaginary frequencies: 0

C1	-0.78760	-0.13060	-0.72990	C8	1.75930	0.24540	-1.30220	H15	3.03080	2.04840	-3.90200
C2	0.61060	0.19040	-0.34650	C9	1.86650	1.24600	-2.27670	C16	-1.62380	-0.86530	0.32160
O3	-0.39250	1.22750	-0.42710	C10	2.95570	1.26410	-3.15150	N17	-2.73430	-0.25970	0.81530
H4	0.89170	-0.06220	0.67860	H11	4.79330	0.29750	-3.74490	O18	-1.26860	-2.00780	0.64610
C5	3.94630	0.28350	-3.06220	H12	4.61860	-1.47730	-2.00790	C19	-3.52780	-0.93680	1.83770
C6	3.84820	-0.71320	-2.08630	H13	2.69520	-1.50270	-0.44500	H20	-4.54710	-1.11370	1.47060
C7	2.76450	-0.72850	-1.20750	H14	1.10110	2.01470	-2.33840	H21	-3.05990	-1.88990	2.07990

H22	-3.58590	-0.31460	2.74010	H25	-2.64880	1.47800	-0.39330	H28	-1.22910	-1.52020	-2.30490
C23	-3.24090	1.04990	0.41150	H26	-4.28040	0.95060	0.07280	H29	-2.18590	-0.02880	-2.37850
H24	-3.22080	1.74370	1.26210	C27	-1.19150	-0.43460	-2.15830	H30	-0.47730	-0.01140	-2.86840

3.6 Reaction of sulfonium ylide 6a and benzaldehyde

3.6.1 Sulfonium ylide

E(B3LYP/6-31G*(dichloromethane)) = -764.60517755785
E(B3LYP-D3/6-311+G**(dichloromethane)) = -764.72639870008
G_{tot}(B3LYP/6-31G*(dichloromethane)) = -764.455918

Number if imaginary frequencies: 0

S1	-2.04032	.34792	-.48556	C9	-.64554	1.12291	-1.39147	H17	-.07072	-3.24810	3.31747
C2	-2.69319	1.86454	.32284	H10	-3.01059	2.56980	-.45163	H18	-.65795	-1.57645	3.29923
C3	-1.38333	-.66384	.70847	H11	-1.92387	2.30988	.95963	H19	.57823	-2.10169	2.13278
C4	-1.90501	-1.99766	.77637	H12	-3.55250	1.55781	.92360	H20	-.14054	.32456	-1.94020
O5	-2.80391	-2.43010	.00611	H13	-.63504	-.25152	1.37232	H21	.04498	1.59369	-.68630
N6	-1.37588	-2.83815	1.74550	H14	-1.08194	-4.93719	1.70644	H22	-1.04629	1.86395	-2.09021
C7	-1.87747	-4.19847	1.87887	H15	-2.29293	-4.37345	2.88116				
C8	-.33088	-2.41118	2.66483	H16	-2.66290	-4.34712	1.13937				

3.6.2 Benzaldehyde

See 3.1.1

3.6.3 *trans*-TSadd

E(B3LYP/6-31G*(dichloromethane)) = -1110.18793466084
E(B3LYP-D3/6-311+G**(dichloromethane)) = -1110.38876171061
G_{tot}(B3LYP/6-31G*(dichloromethane)) = -1109.935341

Number if imaginary frequencies: 1 (-130.81)

S1	1.06225	0.94303	-0.56535	C14	5.90812	1.77596	-0.64319	H27	3.41017	-3.41758	-3.36446
C2	0.11068	1.08481	0.98515	C15	5.73231	3.04387	-0.08080	H28	0.22383	0.33458	-2.71915
C3	2.01115	-0.57077	-0.24455	C16	4.74662	3.23412	0.89154	H29	-0.94544	1.35309	-1.81682
C4	2.65802	-0.95560	-1.54096	C17	3.93656	2.16710	1.28999	H30	-0.78659	-0.39590	-1.41722
O5	2.74265	-0.11931	-2.46416	H18	-0.34874	2.07664	1.00302	H31	3.76476	-1.20757	1.00057
N6	3.20411	-2.20385	-1.65076	H19	0.84898	0.95191	1.78494	H32	5.24892	-0.27091	-0.67539
C7	3.06096	-3.29065	-0.68555	H20	-0.65086	0.30137	1.03424	H33	6.68514	1.61281	-1.38799
C8	3.91232	-2.57303	-2.87390	H21	1.34455	-1.31301	0.19712	H34	6.36721	3.87209	-0.38973
C9	-0.26019	0.50080	-1.75575	H22	2.47890	-4.11237	-1.12583	H35	4.61613	4.21430	1.34701
C10	3.19755	-0.26342	1.17055	H23	4.04982	-3.68128	-0.41364	H36	3.19133	2.29655	2.07055
O11	2.56426	-0.15775	2.29358	H24	2.56404	-2.96353	0.22599				
C12	4.08489	0.90029	0.71384	H25	4.94121	-2.87429	-2.63803				
C13	5.09184	0.71613	-0.24527	H26	3.92914	-1.71985	-3.54974				

3.6.4 *cis*-TSadd

E(B3LYP/6-31G*(dichloromethane)) = -1110.18739169442
E(B3LYP-D3/6-311+G**(dichloromethane)) = -1110.38843371726
G_{tot}(B3LYP/6-31G*(dichloromethane)) = -1109.937681

Number if imaginary frequencies: 1 (-214.53)

S1	1.33427	1.02606	-0.78923	H13	-0.32290	2.29717	0.38387	H25	-0.54612	0.17963	-2.09622
C2	0.08472	1.29039	0.51077	H14	0.63558	1.20726	1.44905	C26	3.12423	-3.98921	3.27993
C3	1.85418	-0.66049	-0.41780	H15	-0.70586	0.53789	0.43313	C27	4.00031	-3.82785	2.20348
C4	2.56218	-1.22019	-1.59129	H16	1.01386	-1.21271	-0.00274	C28	4.00438	-2.63318	1.47815
O5	3.17815	-0.44456	-2.35902	H17	0.88339	-3.80847	-1.68051	C29	3.13528	-1.58660	1.81446
N6	2.61201	-2.57979	-1.76387	H18	2.33791	-4.46538	-0.89915	C30	2.27669	-1.74991	2.91014
C7	1.76418	-3.54891	-1.07418	H19	1.43696	-3.18058	-0.10442	C31	2.26344	-2.94373	3.63276
C8	3.35177	-3.12329	-2.89936	H20	3.95473	-3.97835	-2.57122	H32	3.11850	-4.91816	3.84688
C9	0.25141	0.90866	-2.26320	H21	4.00277	-2.35190	-3.30716	H33	4.68477	-4.62977	1.93294
C10	3.15642	-0.27801	1.04333	H22	2.66546	-3.46766	-3.68679	H34	4.69412	-2.50823	0.64539
O11	2.82974	0.80921	1.63845	H23	0.88716	0.60972	-3.09849	H35	1.63036	-0.92312	3.19415
H12	4.02117	-0.24880	0.34719	H24	-0.16568	1.90399	-2.44669	H36	1.58810	-3.05933	4.47868

3.6.5 *trans*-Cisoid betaine

E(B3LYP/6-31G*(dichloromethane)) = -1110.19088730203
E(B3LYP-D3/6-311+G**(dichloromethane)) = -1110.39145542060
G_{tot}(B3LYP/6-31G*(dichloromethane)) = -1109.938748

Number if imaginary frequencies: 0

S1	0.97928	0.85732	-0.33804	C13	5.21383	-1.16209	0.34352	H25	4.53235	-3.14879	-2.81354
C2	-0.40479	0.61792	0.83009	C14	6.50230	-0.84100	-0.09175	H26	3.72031	-1.77602	-3.61309
C3	1.88150	-0.76408	-0.19220	C15	6.88015	0.49743	-0.22460	H27	2.97942	-3.40078	-3.64765
C4	2.55036	-1.07528	-1.52494	C16	5.96544	1.50525	0.09640	H28	0.71706	0.81861	-2.72920
O5	2.84495	-0.14440	-2.29123	C17	4.68129	1.17523	0.53500	H29	-0.71722	1.50012	-1.89431
N6	2.84378	-2.37601	-1.81175	H18	-0.97576	1.55085	0.85579	H30	-0.47826	-0.27674	-1.95532
C7	2.56732	-3.53422	-0.96669	H19	0.08132	0.41586	1.78387	H31	2.92313	-1.54403	1.53545
C8	3.56201	-2.69142	-3.04590	H20	-1.03520	-0.21283	0.49930	H32	4.93427	-2.20857	0.46050
C9	0.02297	0.69402	-1.89965	H21	1.11110	-1.48647	0.08972	H33	7.21303	-1.63376	-0.31934
C10	2.85315	-0.50870	1.11337	H22	1.92939	-4.24845	-1.50444	H34	7.88244	0.75253	-0.56368
O11	2.27568	0.39518	1.91491	H23	3.50542	-4.04391	-0.70983	H35	6.25805	2.55023	0.00896
C12	4.28125	-0.16072	0.64491	H24	2.07148	-3.25324	-0.03866	H36	3.97038	1.94787	0.81379

3.6.6 *cis*-Cisoid betaine

E(B3LYP/6-31G*(dichloromethane)) = -1110.19473504709
E(B3LYP-D3/6-311+G**(dichloromethane)) = -1110.39536052969
G_{tot}(B3LYP/6-31G*(dichloromethane)) = -1109.941487

Number if imaginary frequencies: 0

S1	1.00859	0.82283	-0.38853	O5	3.08770	-0.25822	-2.14357	C9	0.09122	0.67734	-1.97664
C2	-0.40244	0.65727	0.75999	N6	2.54287	-2.46976	-1.97176	C10	2.85571	-0.56531	1.01502
C3	1.82716	-0.83694	-0.22239	C7	1.86281	-3.61964	-1.37796	O11	2.32283	0.34800	1.84062
C4	2.51110	-1.17930	-1.53690	C8	3.33124	-2.80111	-3.16043	H12	3.78958	-0.23611	0.49808

H13	-0.96747	1.59372	0.72563	H21	3.83354	-1.90578	-3.52179	C29	3.15455	-1.94992	1.62263
H14	0.05834	0.50133	1.73531	H22	2.67699	-3.19818	-3.94729	C30	4.22449	-2.73551	1.17329
H15	-1.03529	-0.18492	0.46336	H23	0.82353	0.70720	-2.78438	C31	4.45068	-4.01042	1.70239
H16	1.03974	-1.52078	0.09503	H24	-0.56916	1.54772	-2.04124	H32	3.77995	-5.50719	3.10898
H17	1.20523	-4.08230	-2.12549	H25	-0.49635	-0.24483	-2.01552	H33	1.89778	-4.11200	3.94963
H18	2.59944	-4.36509	-1.05625	C26	3.60783	-4.51492	2.69610	H34	1.52849	-1.82609	3.01020
H19	1.26533	-3.34153	-0.51285	C27	2.54947	-3.72980	3.16575	H35	4.89138	-2.34538	0.40502
H20	4.07674	-3.56671	-2.91121	C28	2.33183	-2.45623	2.63650	H36	5.28778	-4.60689	1.34367

3.6.7 *trans*-TSrot

E(B3LYP/6-31G*(dichloromethane)) = -1110.18630834985
E(B3LYP-D3/6-311+G**(dichloromethane)) = -1110.38698163968
G_{tot}(B3LYP/6-31G*(dichloromethane)) = -1109.934539

Number if imaginary frequencies: 1 (-23.03)

S1	0.38031	0.64421	-0.47041	C13	2.28532	2.24565	1.64803	H25	4.89089	-2.02590	-2.91834
C2	-0.81762	0.28808	0.86366	C14	1.78469	3.21181	2.52530	H26	3.60220	-1.03478	-3.65447
C3	1.73256	-0.59338	-0.13108	C15	1.32513	2.83457	3.79031	H27	3.44905	-2.81384	-3.60501
C4	2.45039	-0.69826	-1.46913	C16	1.38078	1.48962	4.17281	H28	0.00247	0.13624	-2.78296
O5	2.42214	0.29551	-2.22635	C17	1.88313	0.52876	3.29119	H29	-1.51113	0.59725	-1.93897
N6	3.12952	-1.82721	-1.77520	H18	-1.68756	0.92950	0.69228	H30	-0.83989	-1.05770	-1.72552
C7	3.20465	-3.01620	-0.92197	H19	-0.34632	0.54323	1.81272	H31	3.64141	0.36827	0.42607
C8	3.80816	-1.92783	-3.06633	H20	-1.10838	-0.76627	0.84770	H32	2.66388	2.54754	0.67188
C9	-0.59712	-0.00336	-1.88388	H21	1.24806	-1.50391	0.22912	H33	1.76288	4.25819	2.22600
C10	2.90515	-0.17762	1.07052	H22	2.46726	-3.76926	-1.23434	H34	0.93920	3.58512	4.47728
O11	3.33614	-1.28528	1.63120	H23	4.20458	-3.45160	-1.02673	H35	1.03940	1.19334	5.16302
C12	2.32554	0.89233	2.01447	H24	3.07830	-2.73007	0.12428	H36	1.96062	-0.51729	3.57624

3.6.8 *cis*-TSrot

E(B3LYP/6-31G*(dichloromethane)) = -1110.18720807401
E(B3LYP-D3/6-311+G**(dichloromethane)) = -1110.38757901709
G_{tot}(B3LYP/6-31G*(dichloromethane)) = -1109.935500

Number if imaginary frequencies: 1 (-27.98)

S1	1.15820	0.94604	-0.56111	H13	-0.41123	2.05887	0.85595	H25	-0.75709	0.33410	-1.93927
C2	-0.04312	1.02964	0.81475	H14	0.48211	0.78588	1.74009	C26	6.75732	-1.77330	0.18062
C3	1.67827	-0.82144	-0.46931	H15	-0.86429	0.32760	0.64615	C27	5.98124	-2.75122	0.80727
C4	2.40260	-1.10077	-1.77515	H16	0.77009	-1.41335	-0.33991	C28	4.62601	-2.51745	1.06207
O5	2.77197	-0.14603	-2.48928	H17	1.42247	-4.09541	-1.76508	C29	4.02239	-1.31350	0.68981
N6	2.62747	-2.39463	-2.10427	H18	3.10240	-4.21821	-1.19448	C30	4.81714	-0.32884	0.08410
C7	2.24303	-3.54382	-1.28562	H19	1.95282	-3.24060	-0.27753	C31	6.16976	-0.55542	-0.17633
C8	3.31604	-2.71630	-3.35159	H20	4.27386	-3.20867	-3.14105	H32	7.81194	-1.95252	-0.02116
C9	0.01517	1.10728	-1.98398	H21	3.49679	-1.79937	-3.91068	H33	6.43426	-3.69657	1.10172
C10	2.52689	-1.07636	1.02430	H22	2.70033	-3.39762	-3.95258	H34	4.00918	-3.25913	1.56163
O11	1.91485	-1.99138	1.73480	H23	0.62174	1.00996	-2.88445	H35	4.37604	0.62582	-0.19589
H12	2.50651	-0.03225	1.45020	H24	-0.42973	2.10615	-1.92920	H36	6.76722	0.21758	-0.65708

3.6.9 *trans*-Transoid betaine

E(B3LYP/6-31G*(dichloromethane)) = -1110.18855040847
E(B3LYP-D3/6-311+G**(dichloromethane)) = -1110.38882897648
G_{tot}(B3LYP/6-31G*(dichloromethane)) = -1109.936222

Number if imaginary frequencies: 0

S1	0.01629	0.05911	0.11868	C13	0.74483	1.12710	3.26197	H25	-3.90770	3.78460	-1.93320
C2	1.84097	0.09667	-0.00350	C14	1.93069	0.85095	3.95277	H26	-3.74708	2.02605	-2.17937
C3	-0.45025	1.89554	0.10413	C15	3.03704	1.68982	3.80196	H27	-2.99727	3.16543	-3.33402
C4	-1.79116	1.95412	-0.62184	C16	2.94285	2.81552	2.97370	H28	-1.43708	-0.71018	-1.62562
O5	-2.61969	1.05097	-0.39858	C17	1.75514	3.08814	2.29364	H29	0.07945	-1.66336	-1.53456
N6	-2.00786	2.96362	-1.49505	H18	2.18134	-0.94357	-0.01725	H30	0.08844	-0.04687	-2.32304
C7	-1.11506	4.11711	-1.65096	H19	2.22682	0.60253	0.88104	H31	-1.43421	1.88787	2.04361
C8	-3.24024	2.98519	-2.27972	H20	2.15111	0.61441	-0.91562	H32	-0.11884	0.47525	3.38942
C9	-0.35295	-0.65729	-1.53057	H21	0.37040	2.40433	-0.40648	H33	1.98715	-0.01443	4.61076
C10	-0.63133	2.53569	1.60357	H22	-0.38819	3.95567	-2.45973	H34	3.96204	1.47743	4.33437
O11	-0.93652	3.82059	1.45751	H23	-1.72371	4.99003	-1.90776	H35	3.79760	3.48100	2.86567
C12	0.64894	2.23550	2.40975	H24	-0.62000	4.32141	-0.70052	H36	1.65772	3.97236	1.66825

3.6.10 *cis*-Transoid betaine

E(B3LYP/6-31G*(dichloromethane)) = -1110.18716817024
E(B3LYP-D3/6-311+G**(dichloromethane)) = -1110.38767717324
G_{tot}(B3LYP/6-31G*(dichloromethane)) = -1109.935503

Number if imaginary frequencies: 0

S1	0.37501	-0.12399	-0.45699	H13	2.67881	-0.72145	-0.31354	H25	0.99401	0.52122	-2.72353
C2	2.13211	0.19241	-0.06248	H14	2.21260	0.38071	1.00982	C26	-4.17188	1.31761	3.34893
C3	-0.39172	1.54080	-0.17973	H15	2.51185	1.04296	-0.63522	C27	-3.61575	2.59749	3.28721
C4	-1.78073	1.42501	-0.79773	H16	0.25055	2.26852	-0.68099	C28	-2.37162	2.79583	2.68028
O5	-2.22365	0.29419	-1.08510	H17	-1.77377	4.45277	-1.60313	C29	-1.66711	1.72728	2.12031
N6	-2.48917	2.56043	-0.99431	H18	-2.81779	4.45047	-0.16401	C30	-2.22528	0.44327	2.20646
C7	-2.01626	3.90862	-0.67933	H19	-1.15139	3.88262	-0.01082	C31	-3.46781	0.23682	2.80875
C8	-3.81759	2.48738	-1.59773	H20	-4.57090	2.86900	-0.89739	H32	-5.14098	1.15962	3.81889
C9	0.51026	-0.34856	-2.26990	H21	-4.04626	1.45201	-1.84605	H33	-4.15200	3.44273	3.71554
C10	-0.28083	1.98352	1.47323	H22	-3.84896	3.09941	-2.50835	H34	-1.91514	3.78043	2.63412
O11	0.22039	3.19420	1.58261	H23	-0.50401	-0.48041	-2.64600	H35	-1.68768	-0.40989	1.79604
H12	0.36743	1.15537	1.87615	H24	1.10191	-1.25403	-2.43841	H36	-3.88814	-0.76657	2.85730

3.6.11 *trans*-TSelim

E(B3LYP/6-31G*(dichloromethane)) = -1110.18165731504
E(B3LYP-D3/6-311+G**(dichloromethane)) = -1110.38171659165
G_{tot}(B3LYP/6-31G*(dichloromethane)) = -1109.932115

Number if imaginary frequencies: 1 (-194.42)

S1	-0.00130	-0.16220	0.00384	C13	0.87035	1.03184	3.27758	H25	-3.84747	3.56419	-2.11285
C2	1.81167	-0.15531	-0.22950	C14	2.05850	0.79840	3.97736	H26	-3.63549	1.80110	-2.24770
C3	-0.46535	1.96160	0.20096	C15	3.12783	1.69015	3.85496	H27	-2.89382	2.88766	-3.45763
C4	-1.80248	2.00179	-0.52818	C16	2.99699	2.81804	3.03709	H28	-1.63442	-0.72667	-1.64408
O5	-2.69704	1.21740	-0.17290	C17	1.80713	3.04668	2.34245	H29	-0.18910	-1.77040	-1.77790
N6	-1.92715	2.82379	-1.60141	H18	2.14672	-1.18335	-0.39853	H30	-0.14824	-0.10076	-2.43630
C7	-1.02806	3.94283	-1.89631	H19	2.25420	0.22652	0.69222	H31	-1.37031	1.84452	2.15357
C8	-3.15059	2.76510	-2.39949	H20	2.09638	0.47755	-1.07532	H32	0.03430	0.34224	3.38775
C9	-0.54311	-0.74326	-1.64466	H21	0.40314	2.37064	-0.30975	H33	2.14444	-0.07176	4.62542
C10	-0.54921	2.39916	1.65235	H22	-0.35825	3.70933	-2.73486	H34	4.05146	1.51482	4.40289
O11	-0.83225	3.69648	1.32743	H23	-1.63344	4.81254	-2.17511	H35	3.82142	3.52329	2.94967
C12	0.73633	2.15008	2.44381	H24	-0.45883	4.20474	-1.00595	H36	1.68150	3.93041	1.72209

3.6.12 *cis*-TSelim

E(B3LYP/6-31G*(dichloromethane)) = -1110.17458291738
E(B3LYP-D3/6-311+G**(dichloromethane)) = -1110.37488334236
G_{tot}(B3LYP/6-31G*(dichloromethane)) = -1109.923806

Number if imaginary frequencies: 1 (-228.68)

S1	0.01663	-0.31696	-0.14815	H13	2.26238	-1.21473	-0.19260	H25	0.36977	0.03960	-2.54121
C2	1.83577	-0.22772	0.01018	H14	2.06324	0.05646	1.04103	C26	-3.81867	0.57767	3.95325
C3	-0.52587	1.84572	0.11839	H15	2.25551	0.51277	-0.67759	C27	-3.96719	1.80186	3.29466
C4	-1.83253	1.84133	-0.68069	H16	0.35920	2.24194	-0.37271	C28	-2.91657	2.32114	2.53499
O5	-2.60470	0.87866	-0.55546	H17	-0.74415	4.09979	-2.63808	C29	-1.70889	1.62534	2.41638
N6	-2.06494	2.84441	-1.56546	H18	-1.97499	4.93307	-1.66280	C30	-1.56013	0.41013	3.09683
C7	-1.28854	4.07768	-1.68443	H19	-0.60871	4.17026	-0.84038	C31	-2.60905	-0.11664	3.85511
C8	-3.26955	2.78160	-2.39257	H20	-4.04891	3.44855	-2.00014	H32	-4.63493	0.17235	4.54803
C9	-0.14217	-0.70795	-1.92813	H21	-3.64882	1.76050	-2.40444	H33	-4.90153	2.35407	3.37788
C10	-0.56802	2.22197	1.58880	H22	-3.02239	3.09723	-3.41258	H34	-3.00847	3.27827	2.03005
O11	-0.67019	3.55309	1.29333	H23	-1.21025	-0.71782	-2.15353	H35	-0.61414	-0.12824	3.04407
H12	0.38718	1.91575	2.07377	H24	0.28312	-1.69993	-2.11064	H36	-2.48074	-1.06269	4.37750

3.6.13 SMe2

E(B3LYP/6-31G*(dichloromethane)) = -478.02251398273
E(B3LYP-D3/6-311+G**(dichloromethane)) = -478.07128188397
G_{tot}(B3LYP/6-31G*(dichloromethane)) = -477.972867

Number if imaginary frequencies: 0

S1	0.00000	0.00000	-0.57944	H5	0.89626	1.42144	1.20156	H9	-0.89626	-1.42144	1.20156
C2	0.00000	1.42146	0.57352	C6	0.00000	-1.42146	0.57352				
H3	0.00000	2.32771	-0.04094	H7	0.00000	-2.32771	-0.04094				
H4	-0.89626	1.42144	1.20156	H8	0.89626	-1.42144	1.20156				

3.6.14 *trans*-3a and cis-3a

See 3.1.13 and 3.1.14

4. pKa Values of Ammonium Salts

Computed pKa(H_2O) and experimental pKa(DMSO) values for ammonium salts $R_3N^+CH_2EWG$

R_3N	EWG	Comput. pKa(H_2O)[a]	Exp. pKa(DMSO)[b]
Me_3N	$CONMe_2$	16.1	24.9
DABCO	$CONMe_2$	15.9	

Quinuclidine	$CONMe_2$	16.3	
Me_3N	CO_2Me	13.6	20.0

[a] pKa values were computed using the pKa prediction module as implemented in Jaguar 8.5.

[b] pKa data were obtained at D. A. Evans website: http://evans.rc.fas.harvard.edu/pdf/evans_pKa_table.pdf

5. Copies of HPLC Chromatograms

Operator:Admin Timebase:U-3000_DAD Sequence:WAS_20150415_ZIE

Page 1-2
13.5.2016 10:38 AM

3 rac epoxid2 Hexan_20Isoprop

Sample Name:	**rac epoxid2 Hexan_20Isoprop**	*Injection Volume:*	**20,0**
Vial Number:	**RE3**	*Channel:*	**UV_VIS_1**
Sample Type:	**unknown**	*Wavelength:*	**220**
Control Program:	**OD_H_60Min_100A_flow0_5**	*Bandwidth:*	**4**
Quantif. Method:	**OD_H**	*Temperature/Column:*	**10**
Recording Time:	**15.4.2015 15:56**	*Flow ml/min:*	**0,500**
Run Time (min):	**22,28**	*Sample Amount:*	**1,0000**

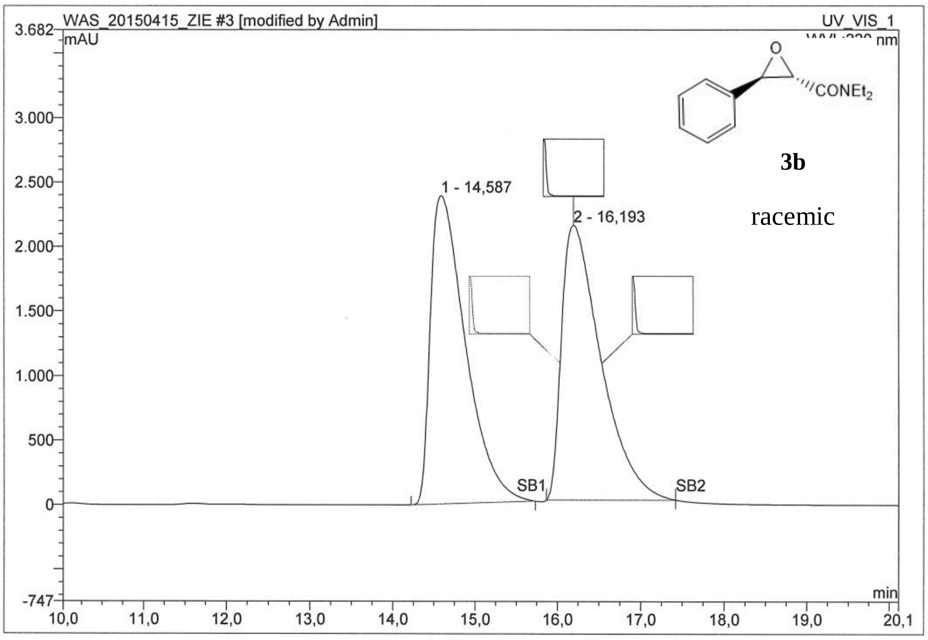

No.	Ret.Time min	Peak Name	Height mAU	Area mAU*min	Rel.Area %	Amount n.a.	Type
1	14,59	n.a.	2387,572	1179,866	50,22	n.a.	BMB*
2	16,19	n.a.	2126,276	1169,484	49,78	n.a.	BMB*
**Total:**			4513,849	2349,350	100,00	0,000	

default/Integration

Chromeleon (c) Dionex 1996-2006
Version 6.80 SR12 Build 3578 (207169)

1 ZIE-192-02 verdünnt2 80Hexan_20Isoprop

Sample Name:	**ZIE-192-02 verdünnt2 80Hexan_20Isoprop**	*Injection Volume:*	**20,0**
Vial Number:	**RE8**	*Channel:*	**UV_VIS_1**
Sample Type:	**unknown**	*Wavelength:*	**220**
Control Program:	**OD_H_60Min_100A_flow0_5**	*Bandwidth:*	**4**
Quantif. Method:	**OD_H**	*Temperature/Column:*	**10**
Recording Time:	**26.5.2015 17:04**	*Flow ml/min:*	**0,500**
Run Time (min):	**21,74**	*Sample Amount:*	**1,0000**

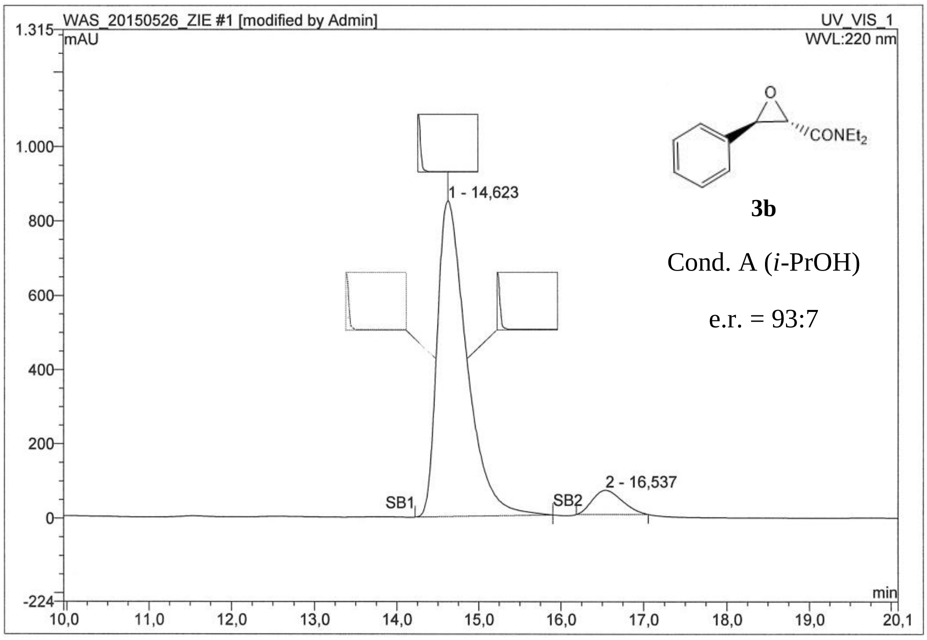

3b

Cond. A (*i*-PrOH)

e.r. = 93:7

No.	Ret.Time min	Peak Name	Height mAU	Area mAU*min	Rel.Area %	Amount n.a.	Type
1	14,62	n.a.	850,041	356,143	92,91	n.a.	BMB*
2	16,54	n.a.	64,857	27,183	7,09	n.a.	BMB*
**Total:**			914,898	383,327	100,00	0,000	

3 ZIE-212-02 80 Hexan_20 Isoprop

Sample Name:	**ZIE-212-02 80 Hexan_20 Isoprop**	*Injection Volume:*	**20,0**
Vial Number:	**RD1**	*Channel:*	**UV_VIS_1**
Sample Type:	**unknown**	*Wavelength:*	**220**
Control Program:	**OD_H_60Min_100A_flow0_5**	*Bandwidth:*	**4**
Quantif. Method:	**OD_H**	*Temperature/Column:*	**10**
Recording Time:	**16.6.2015 11:00**	*Flow ml/min:*	**0,500**
Run Time (min):	**60,00**	*Sample Amount:*	**1,0000**

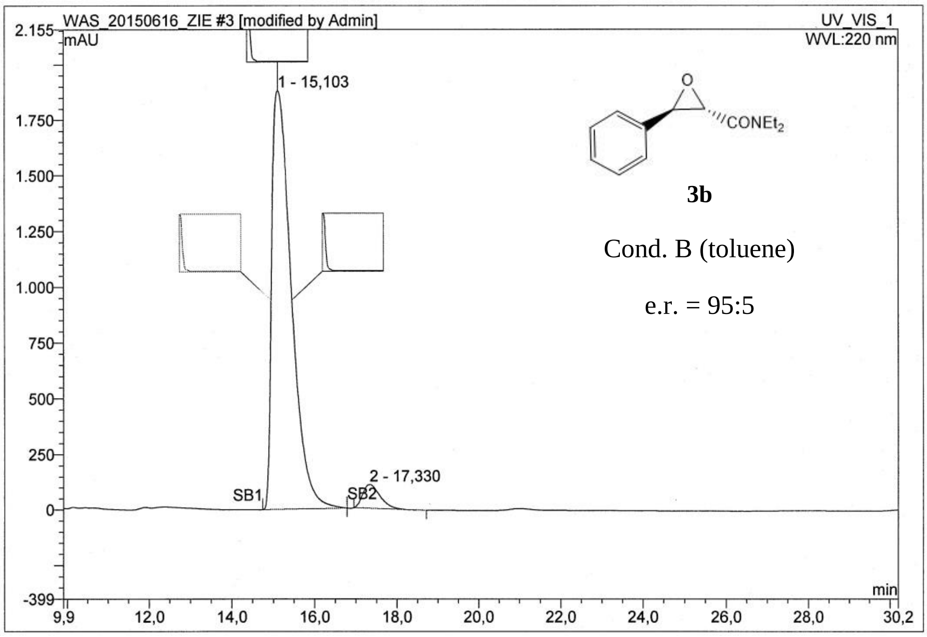

No.	Ret.Time min	Peak Name	Height mAU	Area mAU*min	Rel.Area %	Amount n.a.	Type
1	15,10	n.a.	1881,193	1037,664	95,22	n.a.	BMB*
2	17,33	n.a.	107,751	52,127	4,78	n.a.	BMB*
**Total:**			1988,944	1089,791	100,00	0,000	

3 ZIE-247 80 Hexan_20 Isoprop

Sample Name:	**ZIE-247 80 Hexan_20 Isoprop**	*Injection Volume:*	**20,0**
Vial Number:	**RC8**	*Channel:*	**UV_VIS_1**
Sample Type:	**unknown**	*Wavelength:*	**220**
Control Program:	**OD_H_60Min_100A_flow0_5**	*Bandwidth:*	**4**
Quantif. Method:	**OD_H**	*Temperature/Column:*	**10**
Recording Time:	**21.7.2015 15:20**	*Flow ml/min:*	**0,500**
Run Time (min):	**49,86**	*Sample Amount:*	**1,0000**

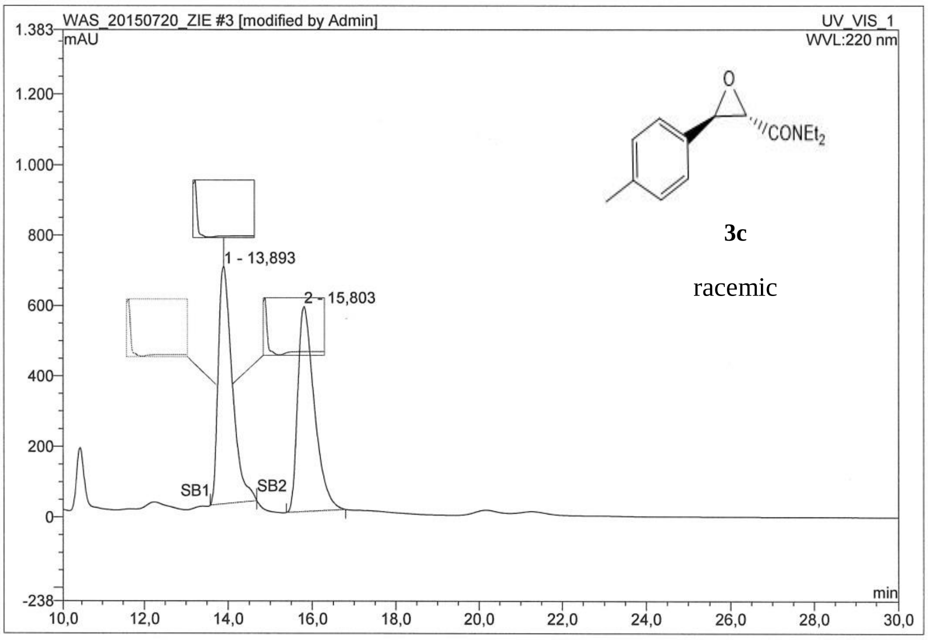

3c

racemic

No.	Ret.Time min	Peak Name	Height mAU	Area mAU*min	Rel.Area %	Amount n.a.	Type
1	13,89	n.a.	673,316	261,435	49,24	n.a.	BMB*
2	15,80	n.a.	581,468	269,479	50,76	n.a.	BMB*
**Total:**			1254,784	530,914	100,00	0,000	

t

15 ZIE-268-01 90 Hexan_10 Isoprop

Sample Name:	**ZIE-268-01 90 Hexan_10 Isoprop**	*Injection Volume:*	**20,0**
Vial Number:	**RD4**	*Channel:*	**UV_VIS_1**
Sample Type:	**unknown**	*Wavelength:*	**220**
Control Program:	**OD_H_60Min_100A_flow0_5**	*Bandwidth:*	**4**
Quantif. Method:	**OD_H**	*Temperature/Column:*	**10**
Recording Time:	**3.8.2015 15:59**	*Flow ml/min:*	**0,500**
Run Time (min):	**24,01**	*Sample Amount:*	**1,0000**

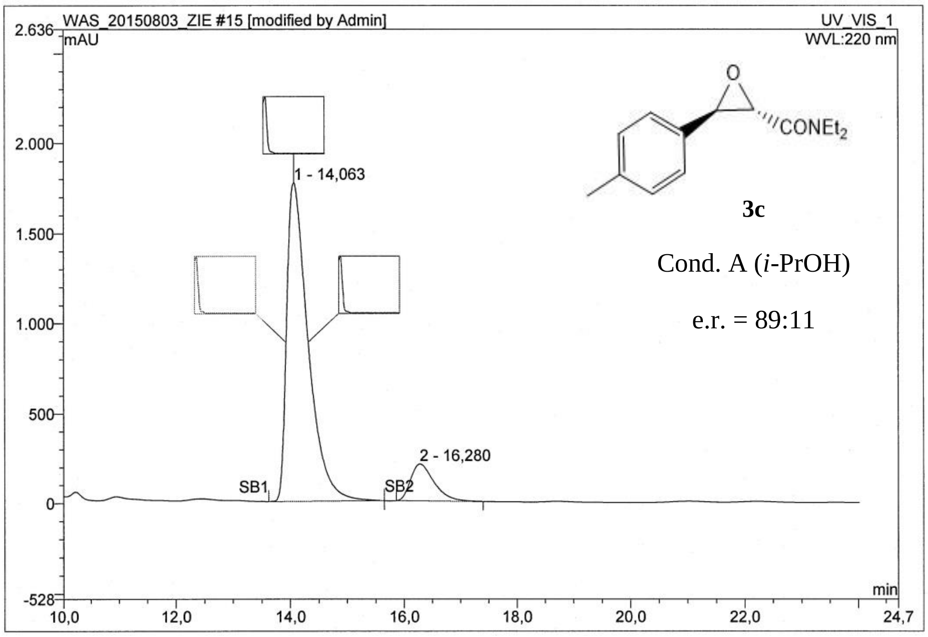

3c

Cond. A (*i*-PrOH)

e.r. = 89:11

No.	Ret.Time min	Peak Name	Height mAU	Area mAU*min	Rel.Area %	Amount	Type
1	14,06	n.a.	1769,180	772,751	88,67	n.a.	BMB*
2	16,28	n.a.	205,552	98,711	11,33	n.a.	BMB*
**Total:**			1974,732	871,462	100,00	0,000	

Chromeleon (c) Dionex 1996-2006
Version 6.80 SR12 Build 3578 (207169)

17 ZIE-269-01 80 Hexan_20 Isoprop

Sample Name:	**ZIE-269-01 80 Hexan_20 Isoprop**	*Injection Volume:*	**20,0**
Vial Number:	**RC3**	*Channel:*	**UV_VIS_1**
Sample Type:	**unknown**	*Wavelength:*	**220**
Control Program:	**OD_H_60Min_100A_flow0_5**	*Bandwidth:*	**4**
Quantif. Method:	**OD_H**	*Temperature/Column:*	**10**
Recording Time:	**3.8.2015 12:00**	*Flow ml/min:*	**0,500**
Run Time (min):	**47,30**	*Sample Amount:*	**1,0000**

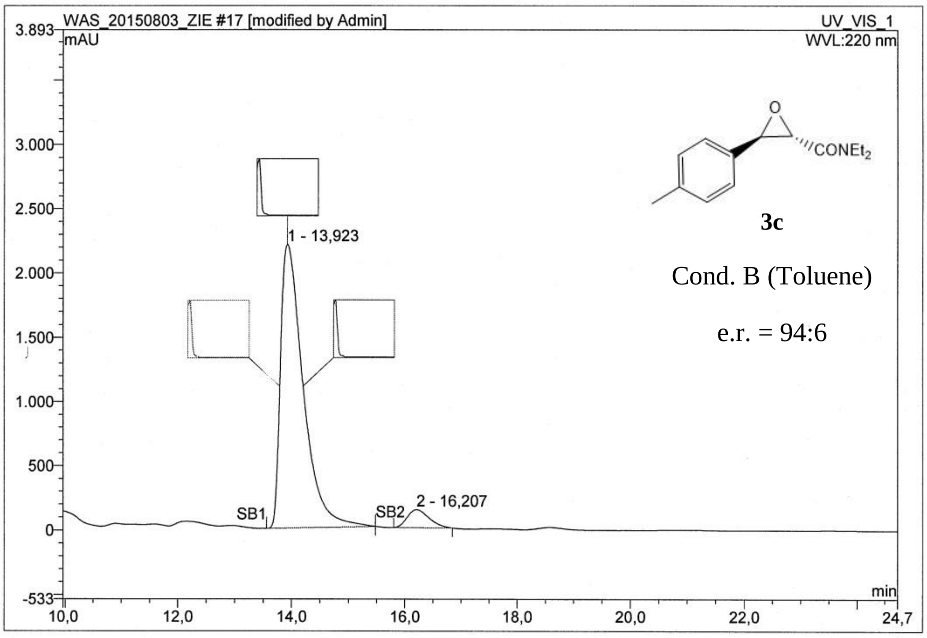

3c

Cond. B (Toluene)

e.r. = 94:6

No.	Ret.Time min	Peak Name	Height mAU	Area mAU*min	Rel.Area %	Amount	Type
1	13,92	n.a.	2205,085	1024,549	94,27	n.a.	BMB*
2	16,21	n.a.	138,813	62,242	5,73	n.a.	BMB*
**Total:**			2343,898	1086,790	100,00	0,000	

8 ZIE-237-02 95_Hexan 5_IPA

Sample Name:	**ZIE-237-02 95_Hexan 5_IPA**	*Injection Volume:*	**20,0**
Vial Number:	**RC3**	*Channel:*	**UV_VIS_1**
Sample Type:	**unknown**	*Wavelength:*	**220**
Control Program:	**AD_H_60Min_100A_flow0_5**	*Bandwidth:*	**4**
Quantif. Method:	**default**	*Temperature/Column:*	**10**
Recording Time:	**25.1.2016 10:08**	*Flow ml/min:*	**0,500**
Run Time (min):	**52,06**	*Sample Amount:*	**1,0000**

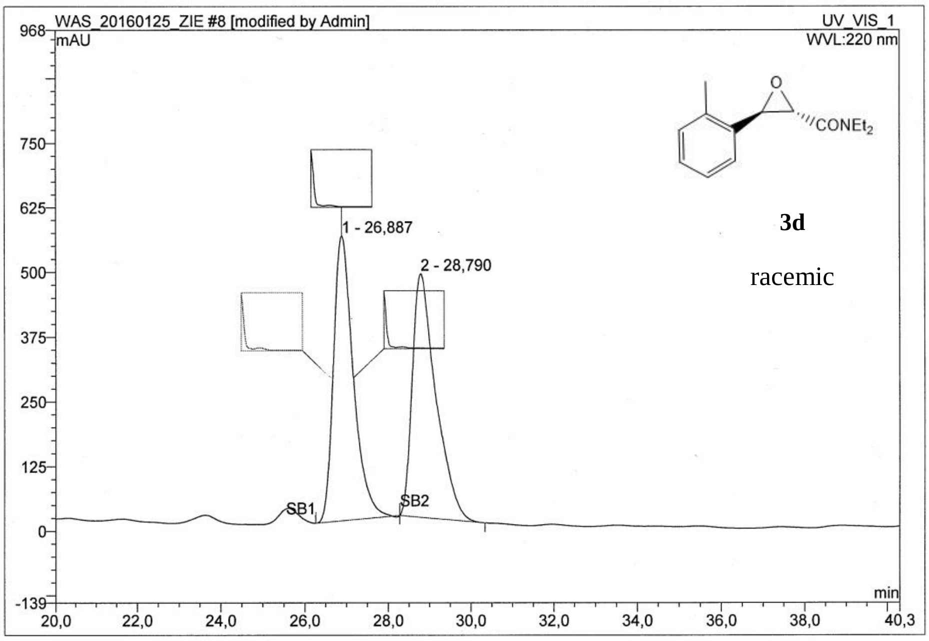

3d

racemic

No.	Ret.Time min	Peak Name	Height mAU	Area mAU*min	Rel.Area %	Amount	Type
1	26,89	n.a.	550,453	303,548	49,37	n.a.	BMb*
2	28,79	n.a.	470,626	311,327	50,63	n.a.	bMB*
**Total:**			1021,079	614,875	100,00	0,000	

default/Integration

Chromeleon (c) Dionex 1996-2006
Version 6.80 SR12 Build 3578 (207169)

11 ZIE-308/2 95_Hexan 5_IPA

Sample Name:	**ZIE-308/2 95_Hexan 5_IPA**	*Injection Volume:*	**20,0**
Vial Number:	**RA3**	*Channel:*	**UV_VIS_1**
Sample Type:	**unknown**	*Wavelength:*	**220**
Control Program:	**AD_H_60Min_100A_flow0_5**	*Bandwidth:*	**4**
Quantif. Method:	**default**	*Temperature/Column:*	**10**
Recording Time:	**2.3.2016 10:27**	*Flow ml/min:*	**0,500**
Run Time (min):	**40,22**	*Sample Amount:*	**1,0000**

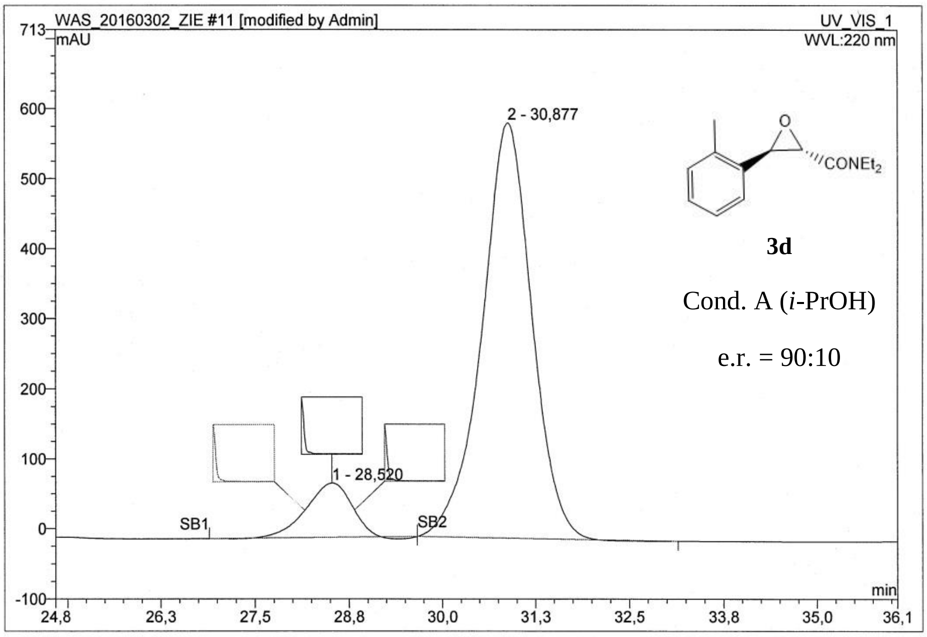

No.	Ret.Time min	Peak Name	Height mAU	Area mAU*min	Rel.Area %	Amount	Type
1	28,52	n.a.	78,037	53,762	10,46	n.a.	BMb*
2	30,88	n.a.	593,549	460,329	89,54	n.a.	bMB*
**Total:**			671,586	514,091	100,00	0,000	

10 ZIE-309/2 95_Hexan 5_IPA

Sample Name:	**ZIE-309/2 95_Hexan 5_IPA**	*Injection Volume:*	**20,0**
Vial Number:	**RA4**	*Channel:*	**UV_VIS_1**
Sample Type:	**unknown**	*Wavelength:*	**220**
Control Program:	**AD_H_60Min_100A_flow0_5**	*Bandwidth:*	**4**
Quantif. Method:	**default**	*Temperature/Column:*	**10**
Recording Time:	**2.3.2016 11:08**	*Flow ml/min:*	**0,500**
Run Time (min):	**42,88**	*Sample Amount:*	**1,0000**

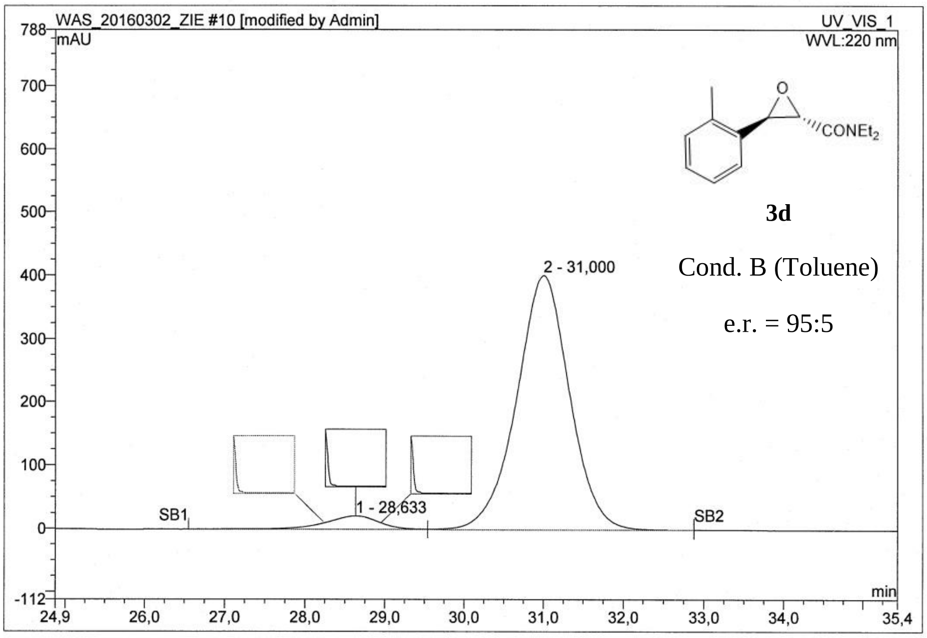

No.	Ret.Time min	Peak Name	Height mAU	Area mAU*min	Rel.Area %	Amount	Type
1	28,63	n.a.	21,551	17,209	5,26	n.a.	BM
2	31,00	n.a.	401,242	310,145	94,74	n.a.	MB
**Total:**			422,793	327,354	100,00	0,000	

4 ZIE-253 80 Hexan_20 Isoprop

Sample Name:	**ZIE-253 80 Hexan_20 Isoprop**	*Injection Volume:*	**20,0**
Vial Number:	**RB7**	*Channel:*	**UV_VIS_1**
Sample Type:	**unknown**	*Wavelength:*	**220**
Control Program:	**AD_H_60Min_100A_flow0_5**	*Bandwidth:*	**4**
Quantif. Method:	**OD_H**	*Temperature/Column:*	**10**
Recording Time:	**21.7.2015 16:11**	*Flow ml/min:*	**0,500**
Run Time (min):	**50,51**	*Sample Amount:*	**1,0000**

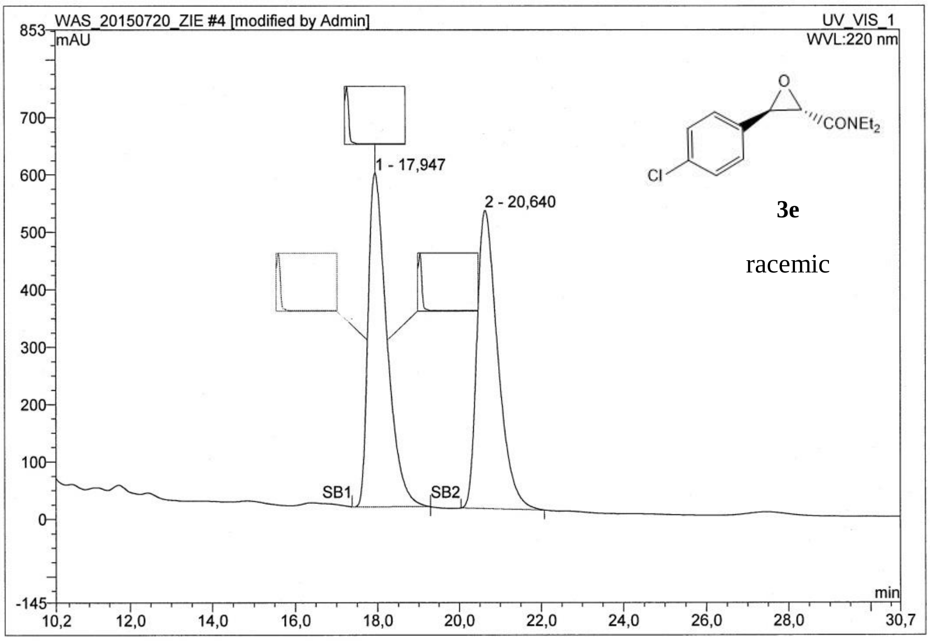

No.	Ret.Time min	Peak Name	Height mAU	Area mAU*min	Rel.Area %	Amount n.a.	Type
1	17,95	n.a.	581,376	309,549	50,13	n.a.	BMB*
2	20,64	n.a.	518,946	307,945	49,87	n.a.	BMB*
Total:			1100,323	617,494	100,00	0,000	

5 ZIE-251-01verd 80 Hexan_20 Isoprop

Sample Name:	**ZIE-251-01verd 80 Hexan_20 Isoprop**	*Injection Volume:*	**20,0**
Vial Number:	**RB2**	*Channel:*	**UV_VIS_1**
Sample Type:	**unknown**	*Wavelength:*	**220**
Control Program:	**OD_H_60Min_100A_flow0_5**	*Bandwidth:*	**4**
Quantif. Method:	**OD_H**	*Temperature/Column:*	**10**
Recording Time:	**23.7.2015 15:33**	*Flow ml/min:*	**0,500**
Run Time (min):	**44,16**	*Sample Amount:*	**1,0000**

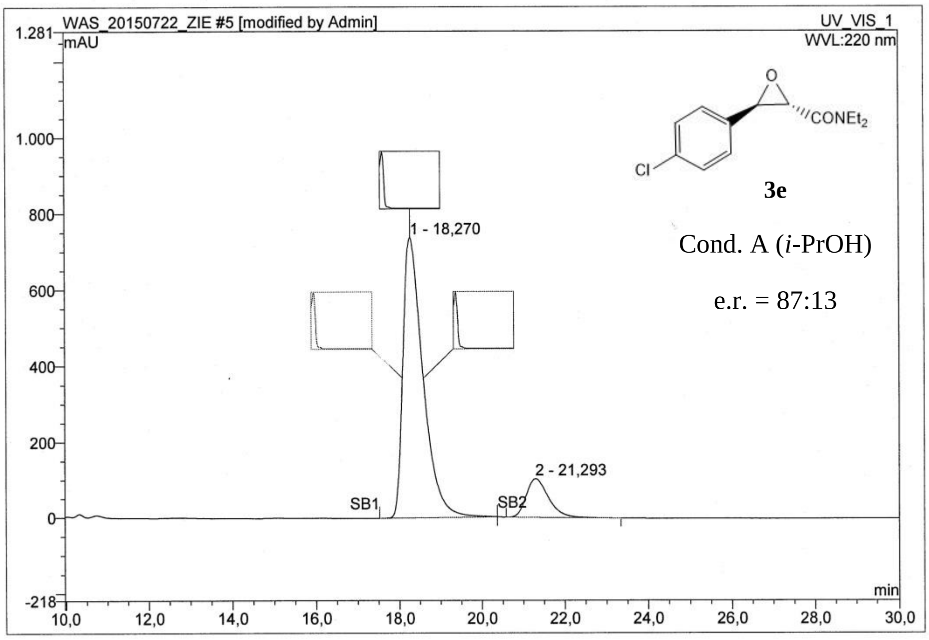

No.	Ret.Time min	Peak Name	Height mAU	Area mAU*min	Rel.Area %	Amount	Type
1	18,27	n.a.	739,069	418,752	86,75	n.a.	BMB*
2	21,29	n.a.	103,144	63,945	13,25	n.a.	BMB*
**Total:**			842,213	482,697	100,00	0,000	

Chromeleon (c) Dionex 1996-2006
Version 6.80 SR12 Build 3578 (207169)

3 ZIE-252-02 80 Hexan_20 Isoprop

Sample Name:	**ZIE-252-02 80 Hexan_20 Isoprop**	*Injection Volume:*	**20,0**
Vial Number:	**RB1**	*Channel:*	**UV_VIS_1**
Sample Type:	**unknown**	*Wavelength:*	**220**
Control Program:	**OD_H_60Min_100A_flow0_5**	*Bandwidth:*	**4**
Quantif. Method:	**OD_H**	*Temperature/Column:*	**10**
Recording Time:	**23.7.2015 14:04**	*Flow ml/min:*	**0,500**
Run Time (min):	**29,13**	*Sample Amount:*	**1,0000**

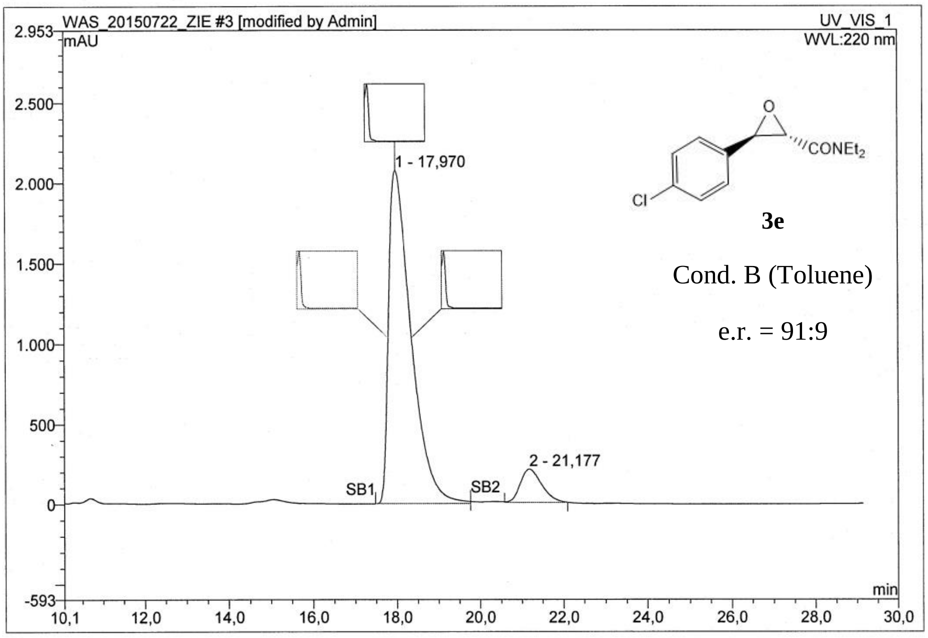

3e

Cond. B (Toluene)

e.r. = 91:9

No.	Ret.Time min	Peak Name	Height mAU	Area mAU*min	Rel.Area %	Amount	Type
1	17,97	n.a.	2080,474	1305,812	91,33	n.a.	BM *
2	21,18	n.a.	208,738	123,964	8,67	n.a.	MB*
**Total:**			2289,212	1429,776	100,00	0,000	

2 ZIE-250 80 Hexan_20 Isoprop

Sample Name:	**ZIE-250 80 Hexan_20 Isoprop**	*Injection Volume:*	**20,0**
Vial Number:	**RD8**	*Channel:*	**UV_VIS_1**
Sample Type:	**unknown**	*Wavelength:*	**220**
Control Program:	**AD_H_60Min_100A_flow0_5**	*Bandwidth:*	**4**
Quantif. Method:	**OD_H**	*Temperature/Column:*	**10**
Recording Time:	**21.7.2015 14:36**	*Flow ml/min:*	**0,500**
Run Time (min):	**43,48**	*Sample Amount:*	**1,0000**

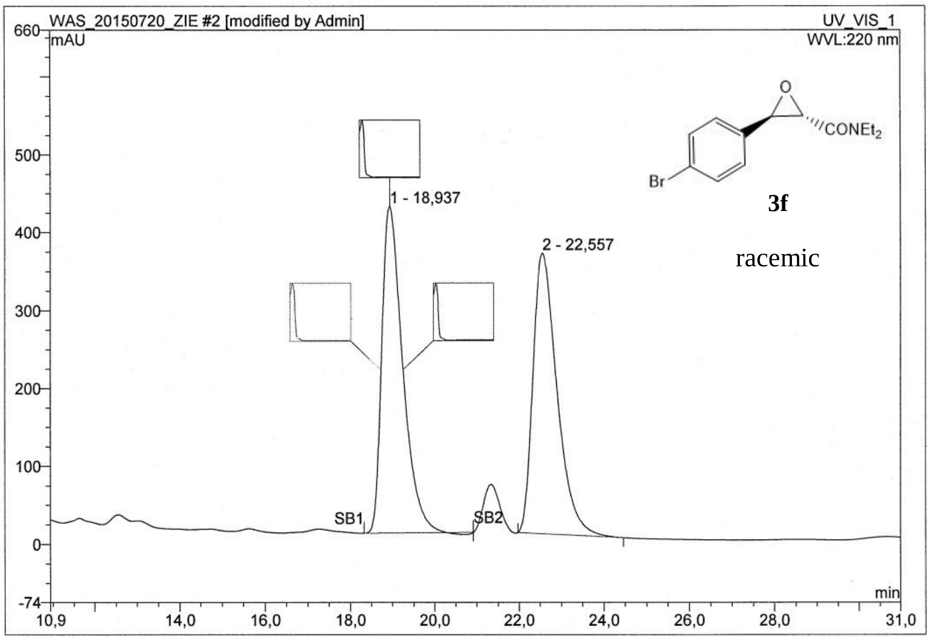

3f

racemic

No.	Ret.Time min	Peak Name	Height mAU	Area mAU*min	Rel.Area %	Amount n.a.	Type
1	18,94	n.a.	419,114	238,754	50,09	n.a.	BMB*
2	22,56	n.a.	359,863	237,850	49,91	n.a.	BMB*
**Total:**			778,976	476,604	100,00	0,000	

Chromeleon (c) Dionex 1996-2006
Version 6.80 SR12 Build 3578 (207169)

1 ZIE-248-01 80 Hexan_20 Isoprop

Sample Name:	**ZIE-248-01 80 Hexan_20 Isoprop**	*Injection Volume:*	**20,0**
Vial Number:	**RD6**	*Channel:*	**UV_VIS_1**
Sample Type:	**unknown**	*Wavelength:*	**220**
Control Program:	**OD_H_60Min_100A_flow0_5**	*Bandwidth:*	**4**
Quantif. Method:	**OD_H**	*Temperature/Column:*	**10**
Recording Time:	**21.7.2015 12:02**	*Flow ml/min:*	**0,500**
Run Time (min):	**58,62**	*Sample Amount:*	**1,0000**

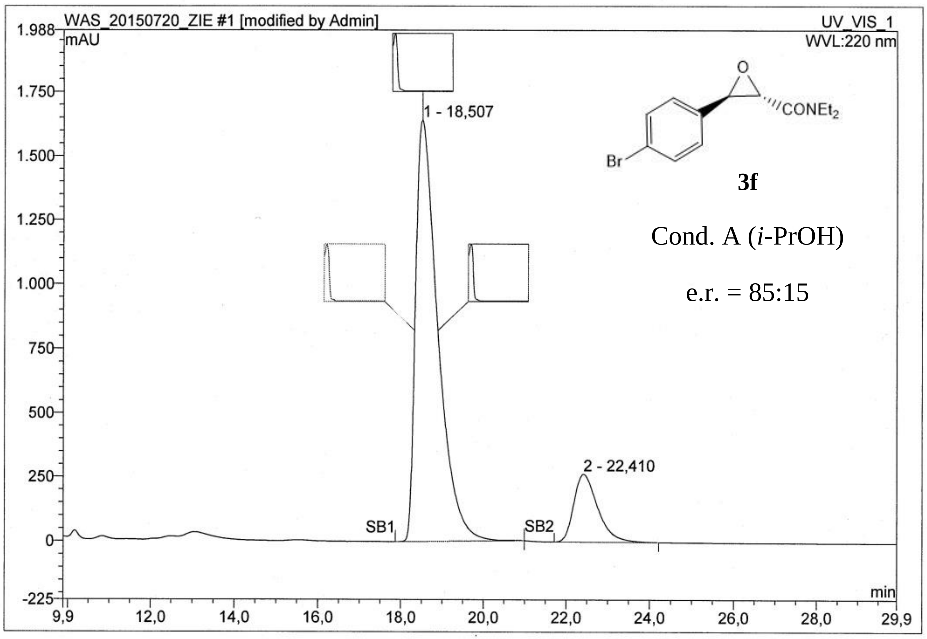

No.	Ret.Time min	Peak Name	Height mAU	Area mAU*min	Rel.Area %	Amount n.a.	Type
1	18,51	n.a.	1639,792	1019,374	85,35	n.a.	BMB*
2	22,41	n.a.	263,145	174,970	14,65	n.a.	BMB*
**Total:**			1902,937	1194,344	100,00	0,000	

6 ZIE-249-03 80 Hexan_20 Isoprop

Sample Name:	**ZIE-249-03 80 Hexan_20 Isoprop**	*Injection Volume:*	**20,0**
Vial Number:	**RA2**	*Channel:*	**UV_VIS_1**
Sample Type:	**unknown**	*Wavelength:*	**220**
Control Program:	**OD_H_60Min_100A_flow0_5**	*Bandwidth:*	**4**
Quantif. Method:	**OD_H**	*Temperature/Column:*	**10**
Recording Time:	**23.7.2015 14:34**	*Flow ml/min:*	**0,500**
Run Time (min):	**28,18**	*Sample Amount:*	**1,0000**

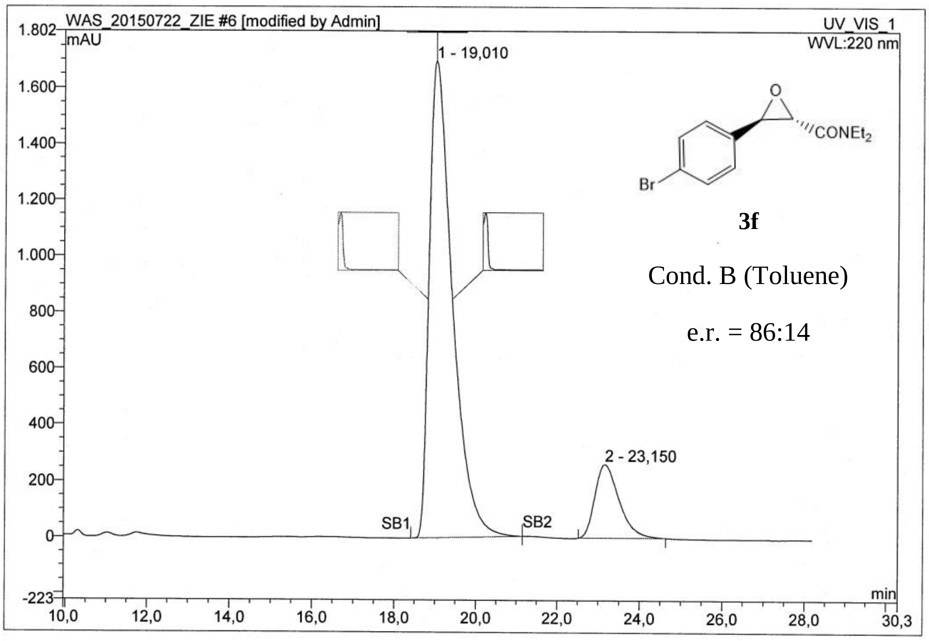

3f

Cond. B (Toluene)

e.r. = 86:14

No.	Ret.Time min	Peak Name	Height mAU	Area mAU*min	Rel.Area %	Amount	Type
1	19,01	n.a.	1694,206	1110,529	86,11	n.a.	BMB*
2	23,15	n.a.	261,519	179,152	13,89	n.a.	BMB*
**Total:**			1955,725	1289,681	100,00	0,000	

7 ZIE-257 conc 80 Hexan_20 Isoprop

Sample Name:	**ZIE-257 conc 80 Hexan_20 Isoprop**	*Injection Volume:*	**20,0**
Vial Number:	**RA2**	*Channel:*	**UV_VIS_1**
Sample Type:	**unknown**	*Wavelength:*	**220**
Control Program:	**OD_H_60Min_100A_flow0_5**	*Bandwidth:*	**4**
Quantif. Method:	**OD_H**	*Temperature/Column:*	**10**
Recording Time:	**30.7.2015 16:15**	*Flow ml/min:*	**0,500**
Run Time (min):	**53,68**	*Sample Amount:*	**1,0000**

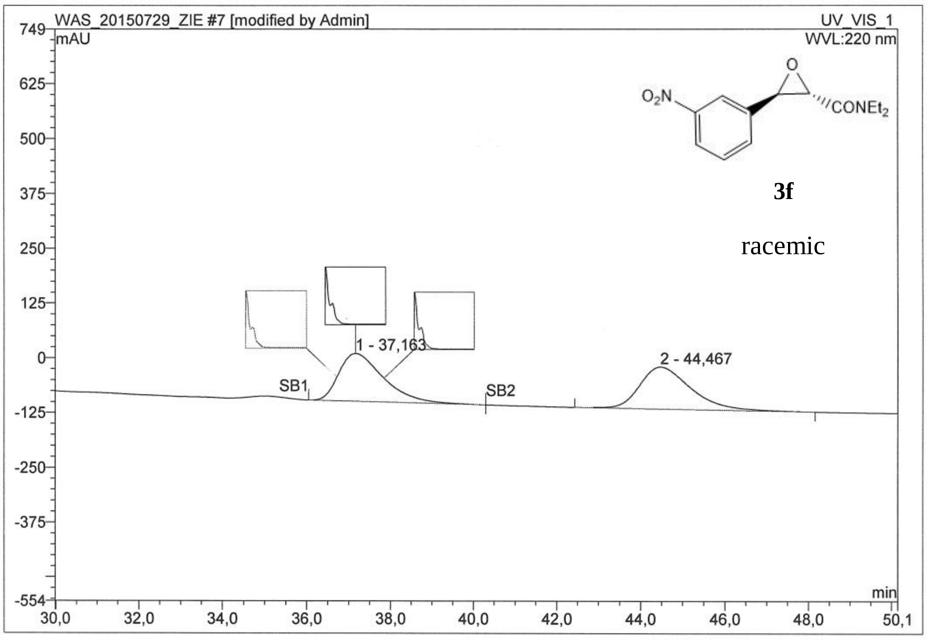

3f

racemic

No.	Ret.Time min	Peak Name	Height mAU	Area mAU*min	Rel.Area %	Amount	Type
1	37,16	n.a.	109,594	136,055	49,81	n.a.	BMB
2	44,47	n.a.	96,247	137,101	50,19	n.a.	BMB
**Total:**			205,841	273,157	100,00	0,000	

default/Integration

Chromeleon (c) Dionex 1996-2006
Version 6.80 SR12 Build 3578 (207169)

8 ZIE-326-01 80 Hexan_20 Isoprop

Sample Name:	**ZIE-326-01 80 Hexan_20 Isoprop**	*Injection Volume:*	**20,0**
Vial Number:	**RB2**	*Channel:*	**UV_VIS_1**
Sample Type:	**unknown**	*Wavelength:*	**220**
Control Program:	**OD_H_60Min_100A_flow0_5**	*Bandwidth:*	**4**
Quantif. Method:	**OD_H**	*Temperature/Column:*	**10**
Recording Time:	**18.1.2016 21:05**	*Flow ml/min:*	**0,500**
Run Time (min):	**50,76**	*Sample Amount:*	**1,0000**

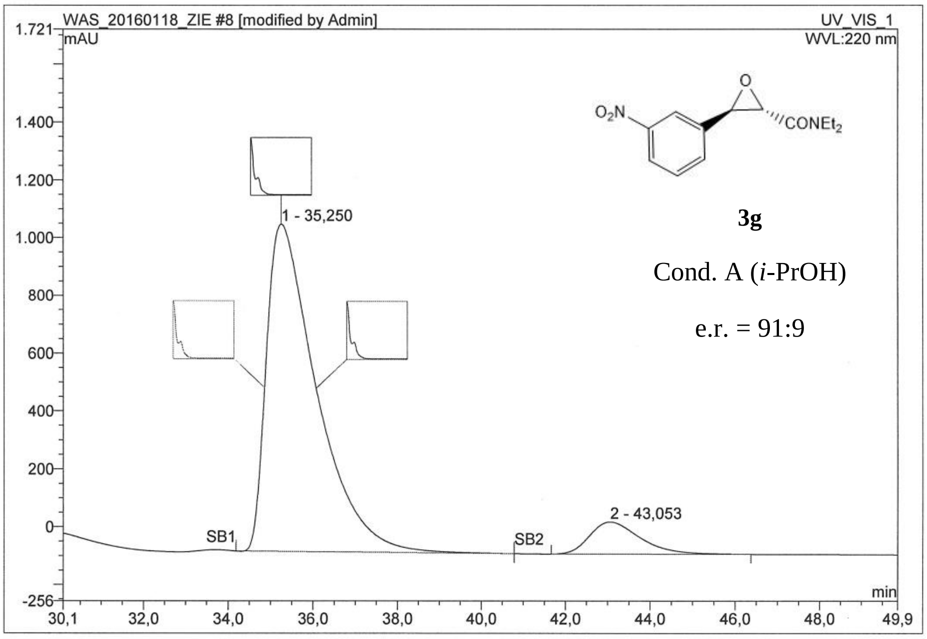

3g

Cond. A (*i*-PrOH)

e.r. = 91:9

No.	Ret.Time min	Peak Name	Height mAU	Area mAU*min	Rel.Area %	Amount n.a.	Type
1	35,25	n.a.	1132,269	1549,210	90,98	n.a.	BMB*
2	43,05	n.a.	110,997	153,577	9,02	n.a.	BMB*
**Total:**			1243,266	1702,787	100,00	0,000	

7 ZIE-327-01 80 Hexan_20 Isoprop

Sample Name:	**ZIE-327-01 80 Hexan_20 Isoprop**	*Injection Volume:*	**20,0**
Vial Number:	**RC2**	*Channel:*	**UV_VIS_1**
Sample Type:	**unknown**	*Wavelength:*	**220**
Control Program:	**OD_H_60Min_100A_flow0_5**	*Bandwidth:*	**4**
Quantif. Method:	**OD_H**	*Temperature/Column:*	**10**
Recording Time:	**18.1.2016 19:43**	*Flow ml/min:*	**0,500**
Run Time (min):	**50,56**	*Sample Amount:*	**1,0000**

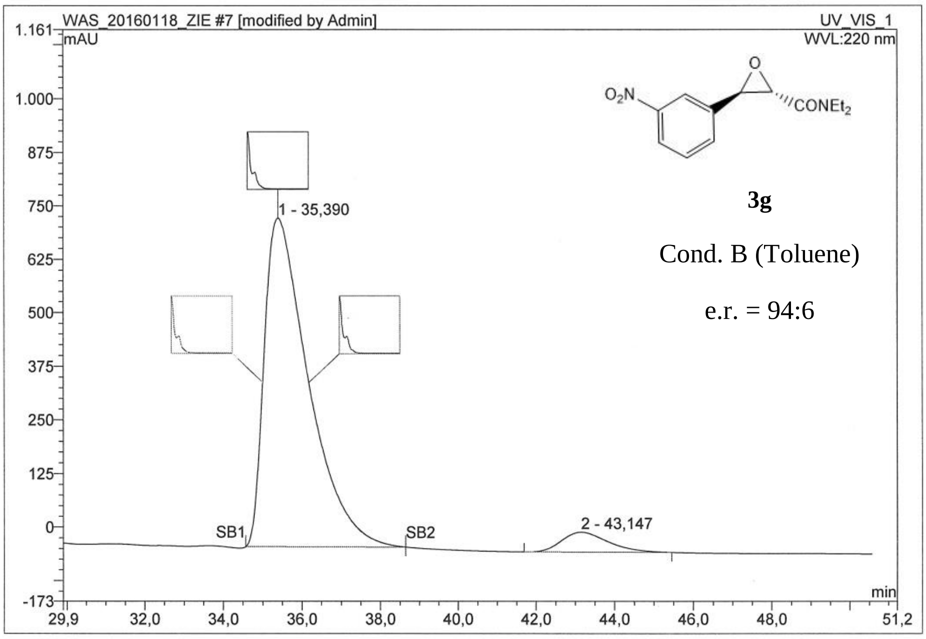

No.	Ret.Time min	Peak Name	Height mAU	Area mAU*min	Rel.Area %	Amount n.a.	Type
1	35,39	n.a.	766,617	996,406	93,97	n.a.	BMB*
2	43,15	n.a.	46,597	63,975	6,03	n.a.	BMB*
**Total:**			813,214	1060,381	100,00	0,000	

1 ZIE-240-01 80 Hexan_20 isopropanol

Sample Name:	**ZIE-240-01 80 Hexan_20 isopropanol**	*Injection Volume:*	**20,0**
Vial Number:	**RD4**	*Channel:*	**UV_VIS_1**
Sample Type:	**unknown**	*Wavelength:*	**220**
Control Program:	**OD_H_60Min_100A_flow0_5**	*Bandwidth:*	**4**
Quantif. Method:	**OD_H**	*Temperature/Column:*	**10**
Recording Time:	**8.7.2015 10:49**	*Flow ml/min:*	**0,500**
Run Time (min):	**60,00**	*Sample Amount:*	**1,0000**

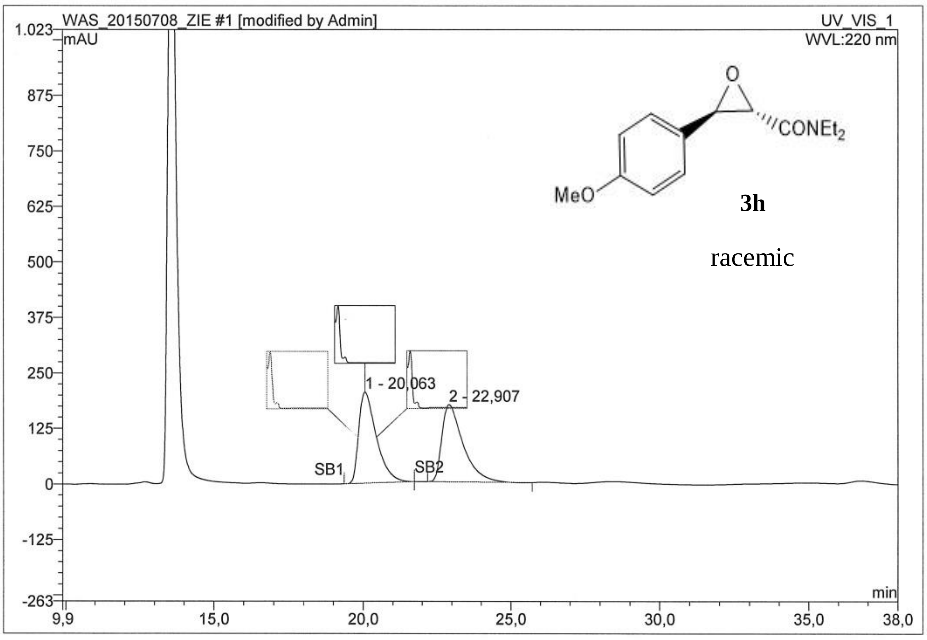

No.	Ret.Time min	Peak Name	Height mAU	Area mAU*min	Rel.Area %	Amount	Type
1	20,06	n.a.	205,040	140,656	49,42	n.a.	BMB*
2	22,91	n.a.	173,630	143,948	50,58	n.a.	BMB*
**Total:**			378,670	284,604	100,00	0,000	

Chromeleon (c) Dionex 1996-2006
Version 6.80 SR12 Build 3578 (207169)

3 ZIE-348-01 80 Hexan_20 Isoprop

Sample Name:	**ZIE-348-01 80 Hexan_20 Isoprop**	*Injection Volume:*	**20,0**
Vial Number:	**RD2**	*Channel:*	**UV_VIS_1**
Sample Type:	**unknown**	*Wavelength:*	**220**
Control Program:	**OD_H_60Min_100A_flow0_5**	*Bandwidth:*	**4**
Quantif. Method:	**OD_H**	*Temperature/Column:*	**10**
Recording Time:	**15.2.2016 14:24**	*Flow ml/min:*	**0,500**
Run Time (min):	**48,76**	*Sample Amount:*	**1,0000**

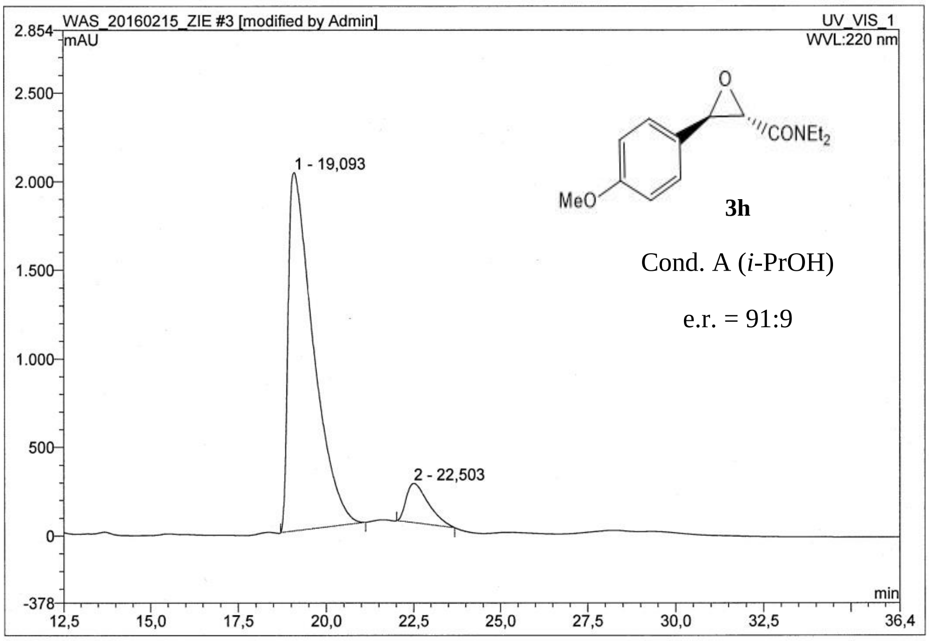

3h

Cond. A (*i*-PrOH)

e.r. = 91:9

No.	Ret.Time min	Peak Name	Height mAU	Area mAU*min	Rel.Area %	Amount n.a.	Type
1	19,09	n.a.	2024,810	1675,337	90,96	n.a.	BMB*
2	22,50	n.a.	222,959	166,516	9,04	n.a.	BMB*
**Total:**			2247,769	1841,854	100,00	0,000	

9 ZIE-260 80 Hexan_20 Isoprop

Sample Name:	**ZIE-260 80 Hexan_20 Isoprop**	*Injection Volume:*	**20,0**
Vial Number:	**RD2**	*Channel:*	**UV_VIS_1**
Sample Type:	**unknown**	*Wavelength:*	**220**
Control Program:	**OD_H_60Min_100A_flow0_5**	*Bandwidth:*	**4**
Quantif. Method:	**OD_H**	*Temperature/Column:*	**10**
Recording Time:	**29.7.2015 16:00**	*Flow ml/min:*	**0,500**
Run Time (min):	**33,34**	*Sample Amount:*	**1,0000**

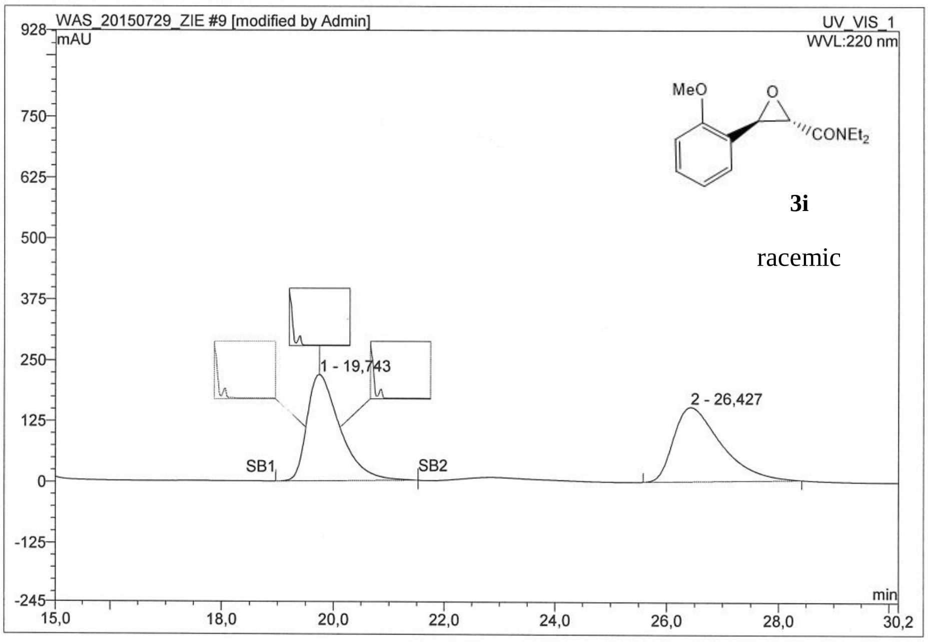

3i

racemic

No.	Ret.Time min	Peak Name	Height mAU	Area mAU*min	Rel.Area %	Amount	Type
1	19,74	n.a.	218,148	149,233	49,94	n.a.	BMB*
2	26,43	n.a.	152,137	149,576	50,06	n.a.	BMB*
**Total:**			370,285	298,810	100,00	0,000	

12 ZIE-258-01 80 Hexan_20 Isoprop

Sample Name:	**ZIE-258-01 80 Hexan_20 Isoprop**	*Injection Volume:*	**20,0**
Vial Number:	**RA3**	*Channel:*	**UV_VIS_1**
Sample Type:	**unknown**	*Wavelength:*	**220**
Control Program:	**OD_H_60Min_100A_flow0_5**	*Bandwidth:*	**4**
Quantif. Method:	**OD_H**	*Temperature/Column:*	**10**
Recording Time:	**29.7.2015 13:32**	*Flow ml/min:*	**0,500**
Run Time (min):	**44,51**	*Sample Amount:*	**1,0000**

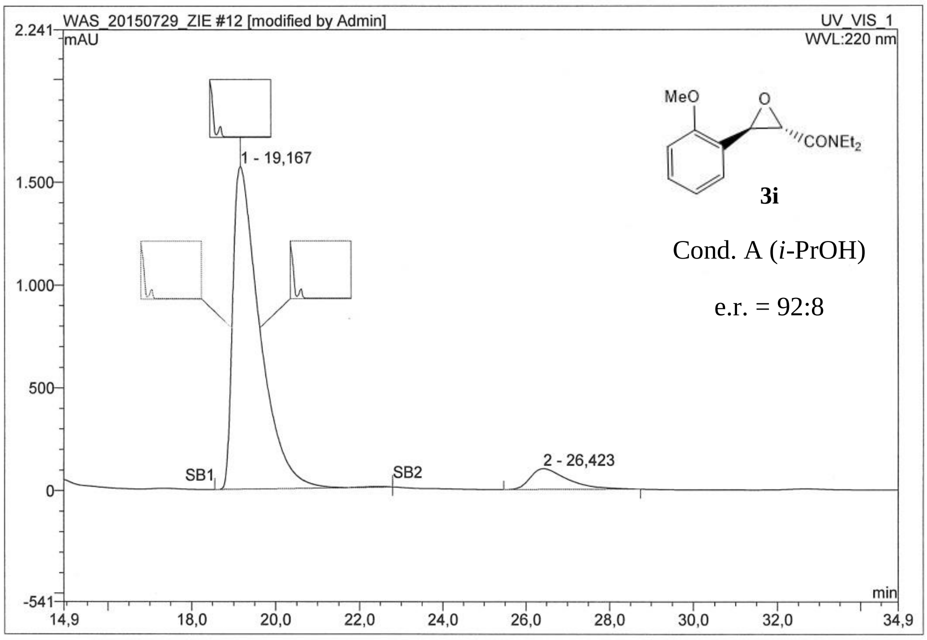

3i

Cond. A (*i*-PrOH)

e.r. = 92:8

No.	Ret.Time min	Peak Name	Height mAU	Area mAU*min	Rel.Area %	Amount	Type
1	19,17	n.a.	1572,028	1168,705	91,60	n.a.	BMB*
2	26,42	n.a.	101,693	107,113	8,40	n.a.	BMB*
**Total:**			1673,721	1275,818	100,00	0,000	

11 ZIE-259-01 80 Hexan_20 Isoprop

Sample Name:	**ZIE-259-01 80 Hexan_20 Isoprop**	*Injection Volume:*	**20,0**
Vial Number:	**RA4**	*Channel:*	**UV_VIS_1**
Sample Type:	**unknown**	*Wavelength:*	**220**
Control Program:	**OD_H_60Min_100A_flow0_5**	*Bandwidth:*	**4**
Quantif. Method:	**OD_H**	*Temperature/Column:*	**10**
Recording Time:	**29.7.2015 12:40**	*Flow ml/min:*	**0,500**
Run Time (min):	**51,04**	*Sample Amount:*	**1,0000**

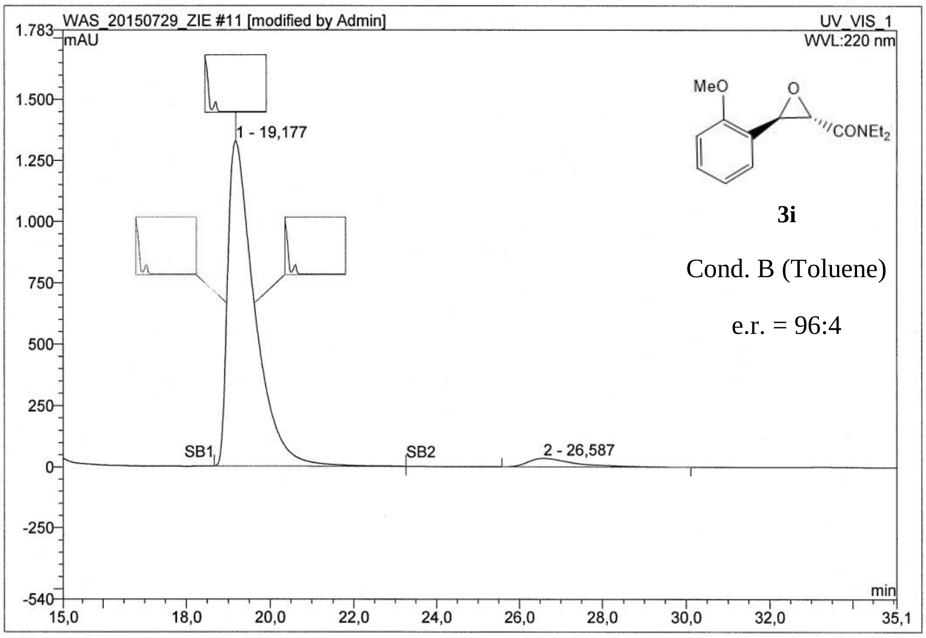

3i

Cond. B (Toluene)

e.r. = 96:4

No.	Ret.Time min	Peak Name	Height mAU	Area mAU*min	Rel.Area %	Amount	Type
1	19,18	n.a.	1326,001	982,486	95,60	n.a.	BMB*
2	26,59	n.a.	34,900	45,237	4,40	n.a.	BMB*
**Total:**			1360,901	1027,723	100,00	0,000	

1 ZIE-403 92 Hexan_8 Isoprop

Sample Name:	**ZIE-403 92 Hexan_8 Isoprop**	*Injection Volume:*	**20,0**
Vial Number:	**RD2**	*Channel:*	**UV_VIS_1**
Sample Type:	**unknown**	*Wavelength:*	**220**
Control Program:	**OD_H_60Min_100A_flow0_5**	*Bandwidth:*	**4**
Quantif. Method:	**OD_H**	*Temperature/Column:*	**10**
Recording Time:	**19.5.2016 22:44**	*Flow ml/min:*	**0,500**
Run Time (min):	**81,88**	*Sample Amount:*	**1,0000**

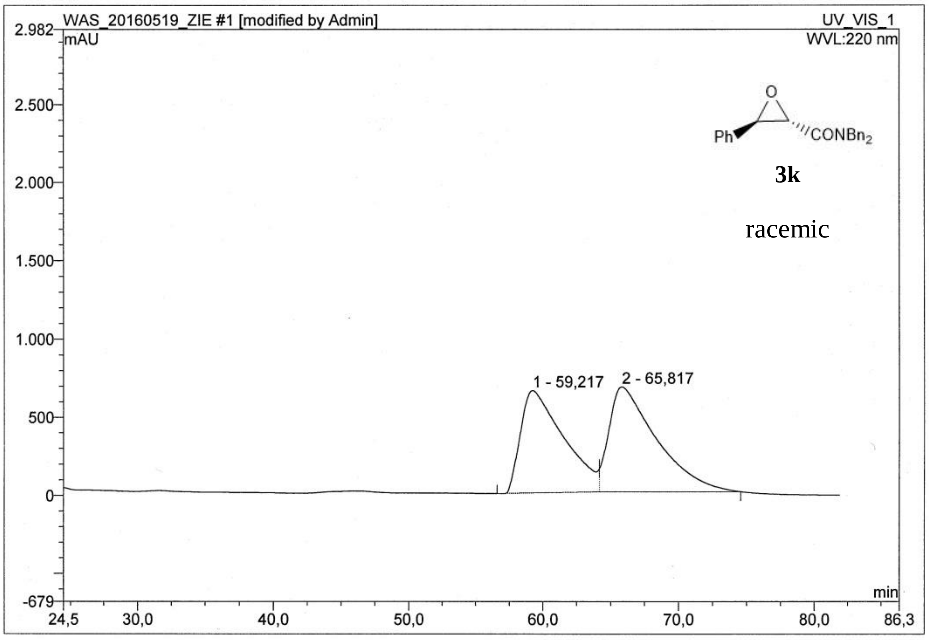

No.	Ret.Time min	Peak Name	Height mAU	Area mAU*min	Rel.Area %	Amount	Type
1	59,22	n.a.	656,809	2436,383	47,20	n.a.	BM *
2	65,82	n.a.	673,157	2725,622	52,80	n.a.	MB*
**Total:**			1329,966	5162,005	100,00	0,000	

Chromeleon (c) Dionex 1996-2006
Version 6.80 SR12 Build 3578 (207169)

3 ZIE-335-02 90 Hexan_10 Isoprop

Sample Name:	**ZIE-335-02 90 Hexan_10 Isoprop**	*Injection Volume:*	**20,0**
Vial Number:	**RD3**	*Channel:*	**UV_VIS_1**
Sample Type:	**unknown**	*Wavelength:*	**220**
Control Program:	**OD_H_90Min_100A_flow0_5**	*Bandwidth:*	**4**
Quantif. Method:	**OD_H**	*Temperature/Column:*	**10**
Recording Time:	**8.2.2016 14:21**	*Flow ml/min:*	**0,500**
Run Time (min):	**75,72**	*Sample Amount:*	**1,0000**

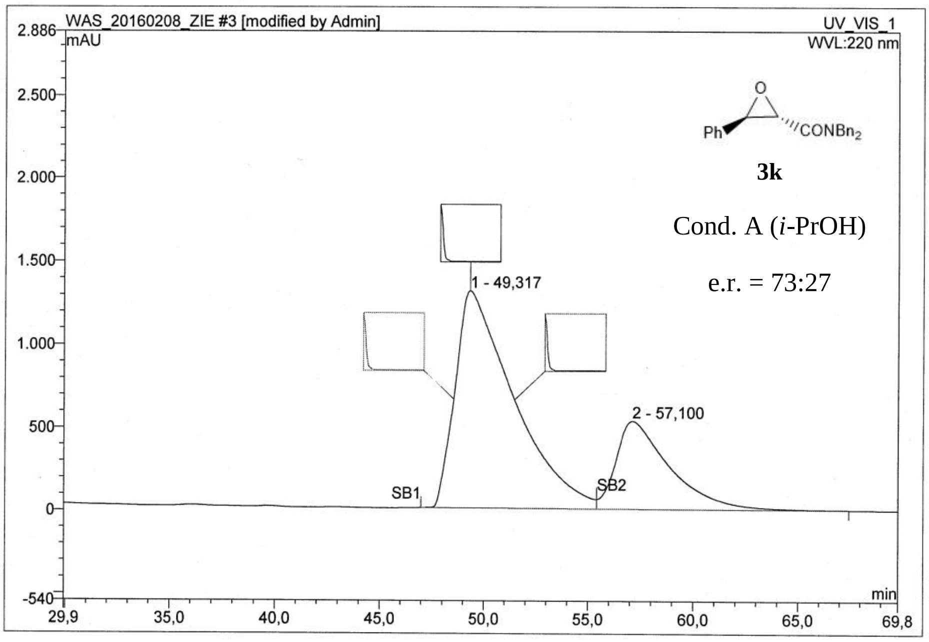

No.	Ret.Time min	Peak Name	Height mAU	Area mAU*min	Rel.Area %	Amount n.a.	Type
1	49,32	n.a.	1307,157	4267,032	72,96	n.a.	BM *
2	57,10	n.a.	529,284	1581,802	27,04	n.a.	MB*
**Total:**			1836,442	5848,834	100,00	0,000	

4 ZIE-404-01 80_Hexan_20_IPA

Sample Name:	**ZIE-404-01 80_Hexan_20_IPA**	*Injection Volume:*	**20,0**
Vial Number:	**RC2**	*Channel:*	**UV_VIS_1**
Sample Type:	**unknown**	*Wavelength:*	**220**
Control Program:	**AD_H_60Min_100A_flow0_5**	*Bandwidth:*	**4**
Quantif. Method:	**default**	*Temperature/Column:*	**10**
Recording Time:	**19.5.2016 16:27**	*Flow ml/min:*	**0,500**
Run Time (min):	**52,47**	*Sample Amount:*	**1,0000**

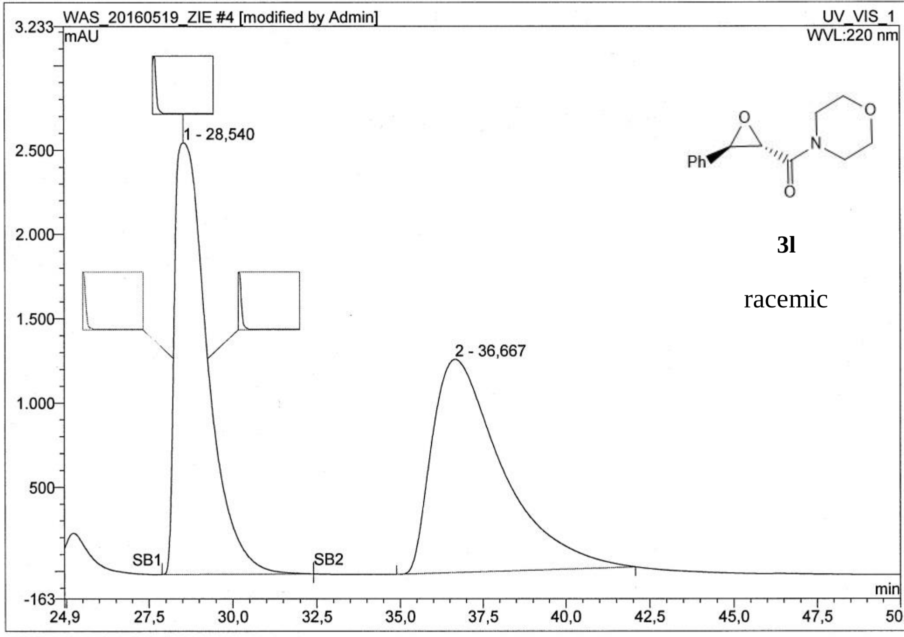

3l

racemic

No.	Ret.Time min	Peak Name	Height mAU	Area mAU*min	Rel.Area %	Amount	Type
1	28,54	n.a.	2562,057	2931,185	48,47	n.a.	BMB*
2	36,67	n.a.	1265,979	3116,128	51,53	n.a.	BMB*
**Total:**			3828,036	6047,313	100,00	0,000	

1 ZIE-317-05hconc 88Hexan_12Isoprop

Sample Name:	**ZIE-317-05hconc 88Hexan_12Isoprop**	*Injection Volume:*	**20,0**
Vial Number:	**RA1**	*Channel:*	**UV_VIS_1**
Sample Type:	**unknown**	*Wavelength:*	**220**
Control Program:	**AD_H_60Min_100A_flow0_5**	*Bandwidth:*	**4**
Quantif. Method:	**default**	*Temperature/Column:*	**10**
Recording Time:	**27.1.2016 16:46**	*Flow ml/min:*	**0,500**
Run Time (min):	**53,65**	*Sample Amount:*	**1,0000**

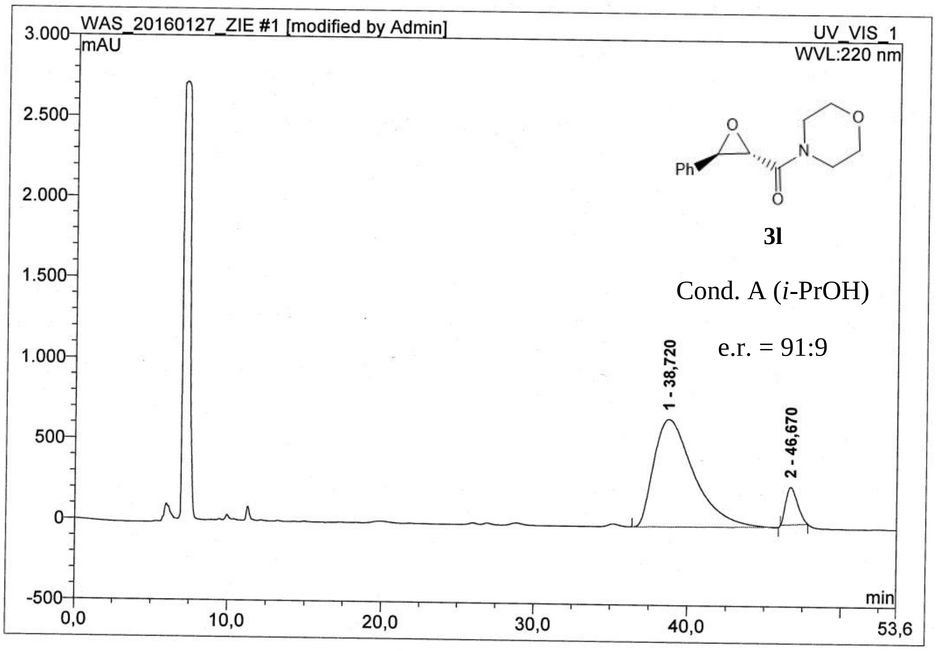

3l

Cond. A (*i*-PrOH)

e.r. = 91:9

No.	Ret.Time min	Peak Name	Height mAU	Area mAU*min	Rel.Area %	Amount	Type
1	38,72	n.a.	667,201	2012,784	90,61	n.a.	BMB*
2	46,67	n.a.	233,611	208,471	9,39	n.a.	BMB*
**Total:**			900,812	2221,255	100,00	0,000	

6. Copies of NMR-Spectra

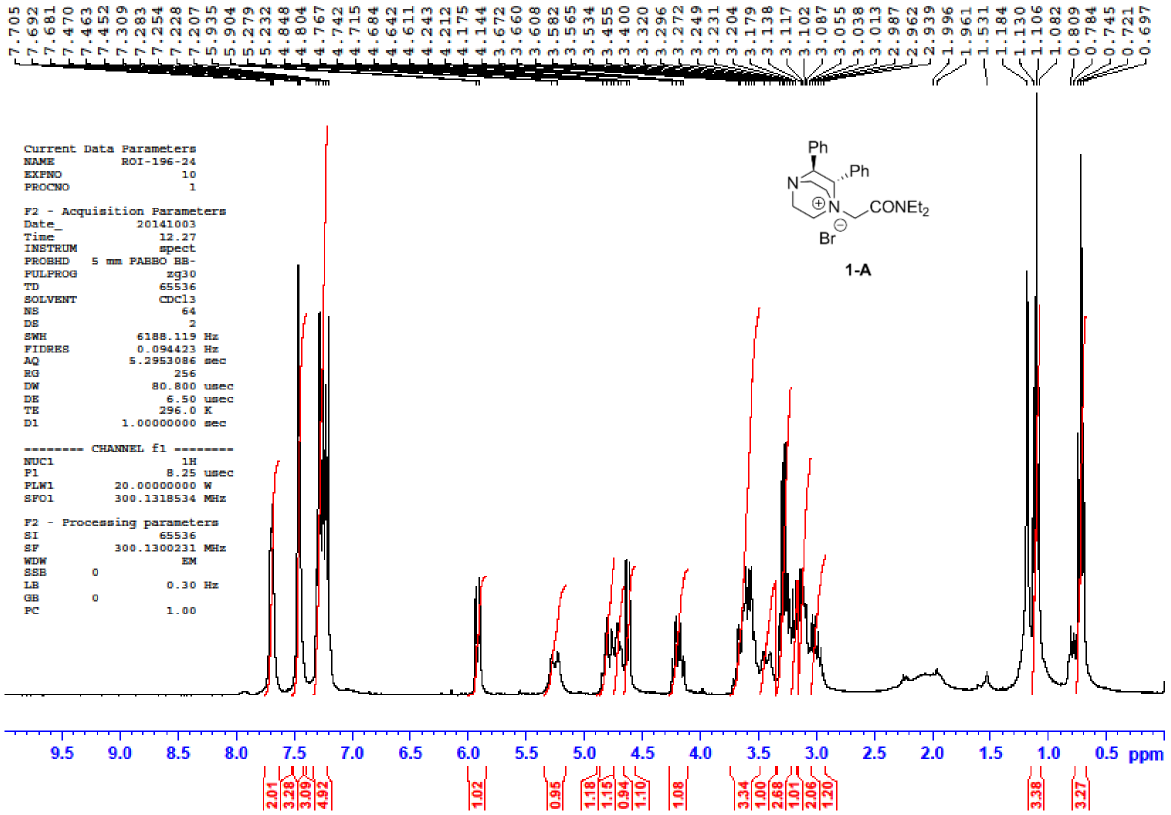

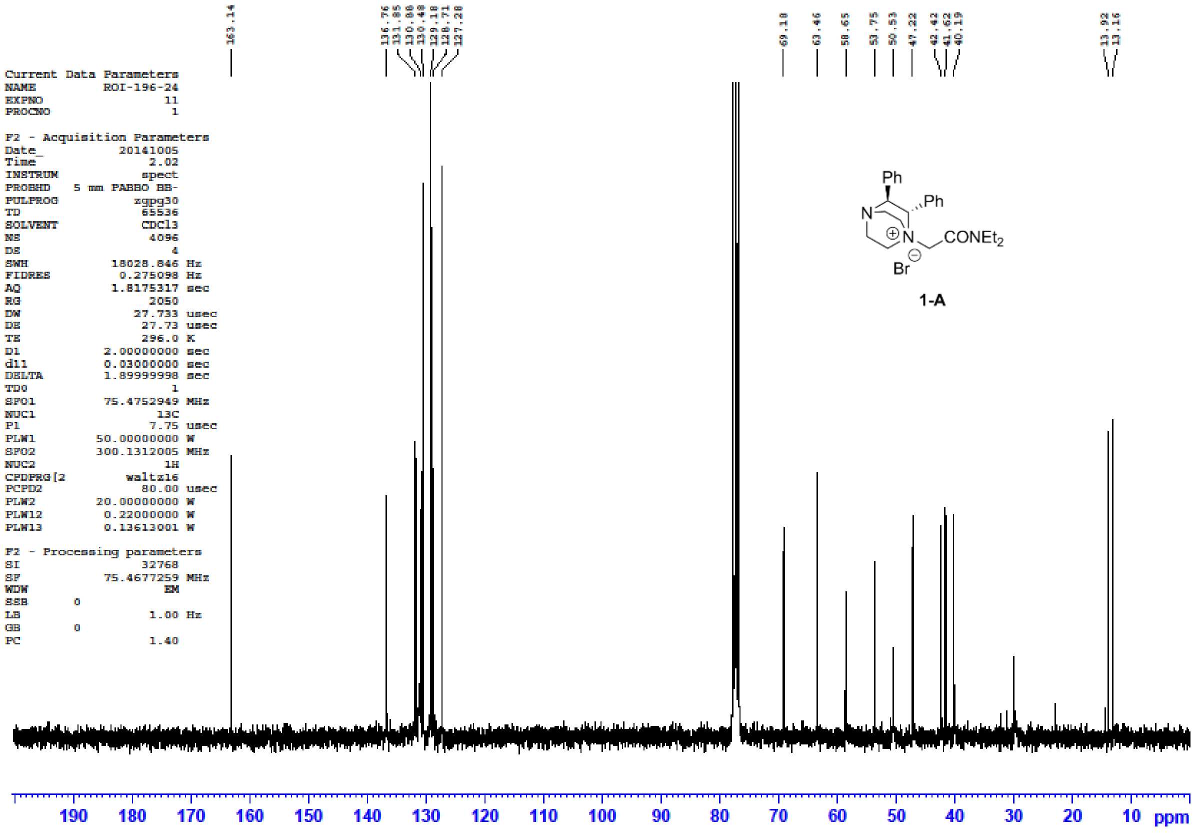

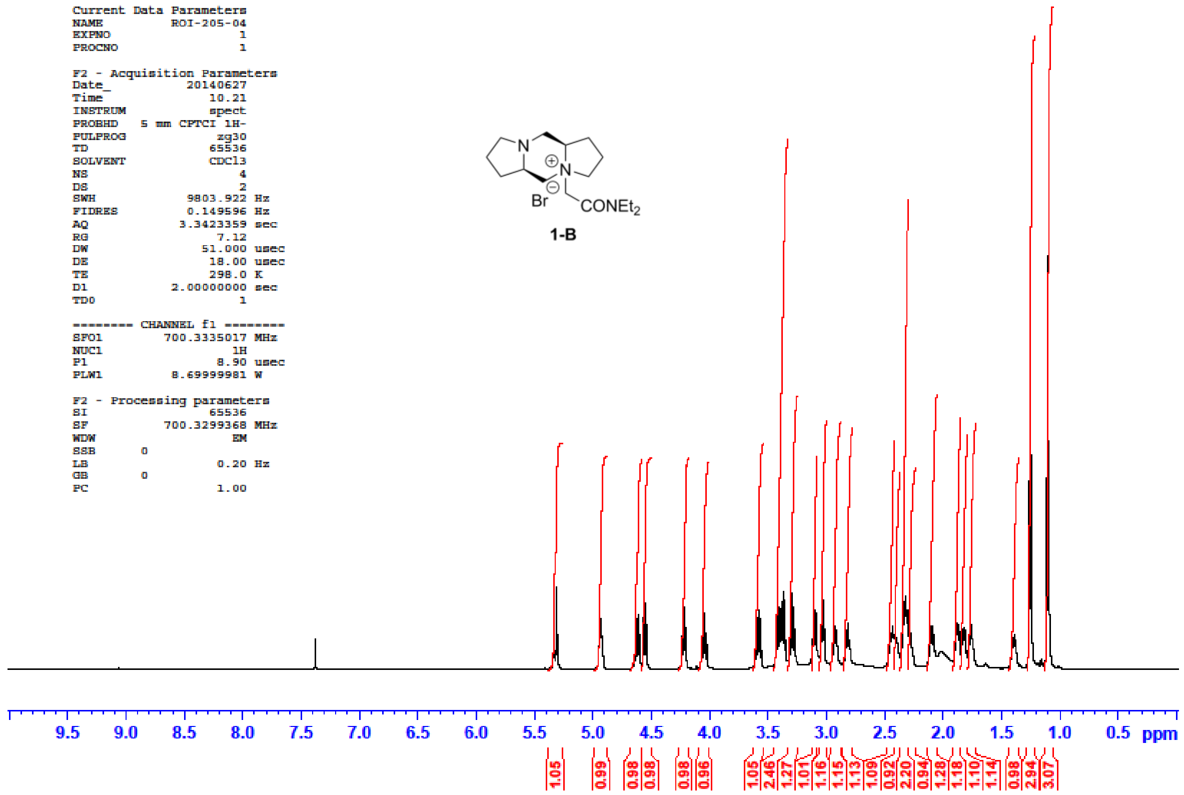
Current Data Parameters
NAME ROI-205-04
EXPNO 1
PROCNO 1
F2 - Acquisition Parameters
Date_ 20140627
Time 10.21
INSTRUM spect
PROBHD 5 mm CPTCI 1H-
PULPROG zg30
TD 65536
SOLVENT CDCl3
NS 4
DS 2
SWH 9803.922 Hz
FIDRES 0.149596 Hz
AQ 3.3423359 sec
RG 7.12
DW 51.000 usec
DE 18.00 usec
TE 298.0 K
D1 2.00000000 sec
TD0 1
======== CHANNEL f1 ========
SFO1 700.3335017 MHz
NUC1 1H
P1 8.90 usec
PLW1 8.69999981 W
F2 - Processing parameters
SI 65536
SF 700.3299368 MHz
WDW EM
SSB 0
LB 0.20 Hz
GB 0
PC 1.00
Br
CONEt2
1-B
9.5 9.0 8.5 8.0 7.5 7.0 6.5 6.0 5.5 5.0 4.5 4.0 3.5 3.0 2.5 2.0 1.5 1.0 0.5 ppm
1.05 0.99 0.98 0.98 0.98 0.96 1.05 2.46 1.27 1.01 1.16 1.15 1.13 1.09 0.92 2.20 0.94 1.28 1.18 1.10 1.14 0.98 2.94 3.07

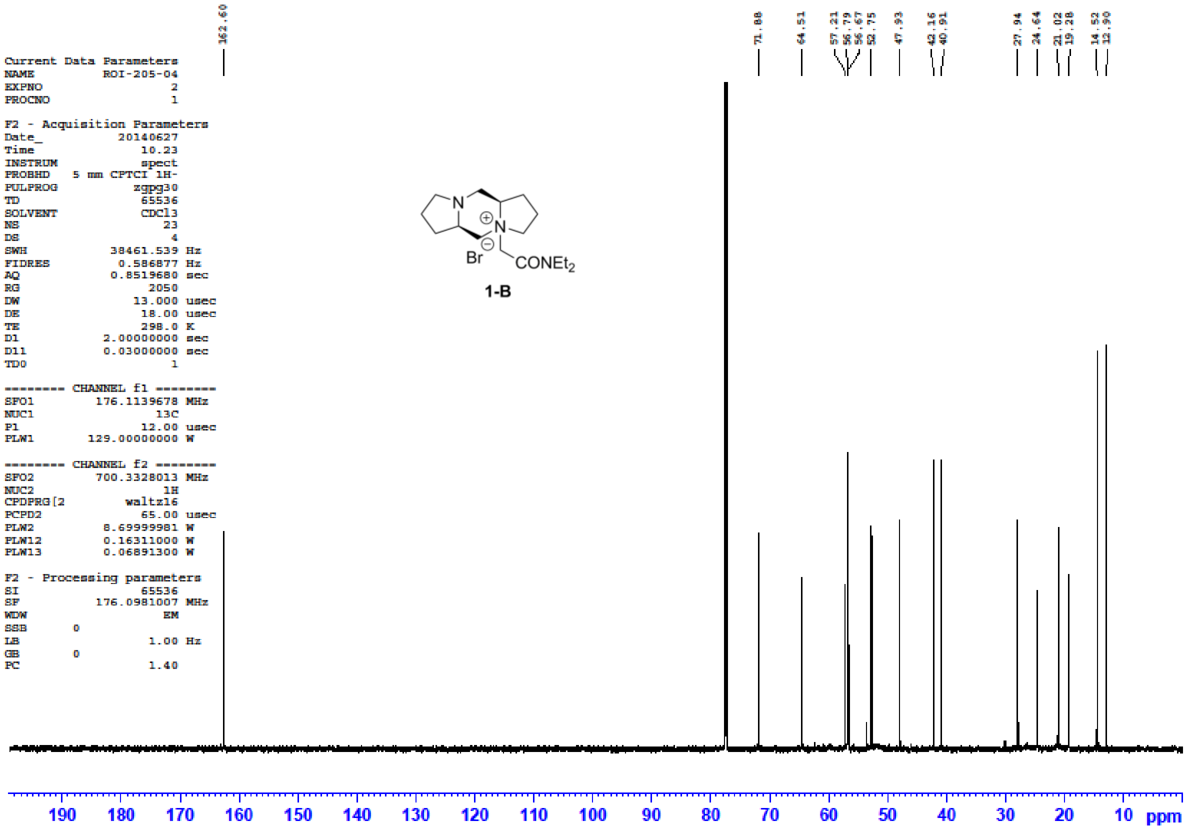
162.60
71.88 64.51 57.21 56.79 56.67 52.75 47.93 42.16 40.91 27.94 24.64 21.02 19.28 14.52 12.90
Current Data Parameters
NAME ROI-205-04
EXPNO 2
PROCNO 1
F2 - Acquisition Parameters
Date_ 20140627
Time 10.23
INSTRUM spect
PROBHD 5 mm CPTCI 1H-
PULPROG zgpg30
TD 65536
SOLVENT CDCl3
NS 23
DS 4
SWH 38461.539 Hz
FIDRES 0.586877 Hz
AQ 0.8519680 sec
RG 2050
DW 13.000 usec
DE 18.00 usec
TE 298.0 K
D1 2.00000000 sec
D11 0.03000000 sec
TD0 1
======== CHANNEL f1 ========
SFO1 176.1139678 MHz
NUC1 13C
P1 12.00 usec
PLW1 129.00000000 W
======== CHANNEL f2 ========
SFO2 700.3328013 MHz
NUC2 1H
CPDPRG[2 waltz16
PCPD2 65.00 usec
PLW2 8.69999981 W
PLW12 0.16311000 W
PLW13 0.06891300 W
F2 - Processing parameters
SI 65536
SF 176.0981007 MHz
WDW EM
SSB 0
LB 1.00 Hz
GB 0
PC 1.40
Br
CONEt2
1-B
190 180 170 160 150 140 130 120 110 100 90 80 70 60 50 40 30 20 10 ppm

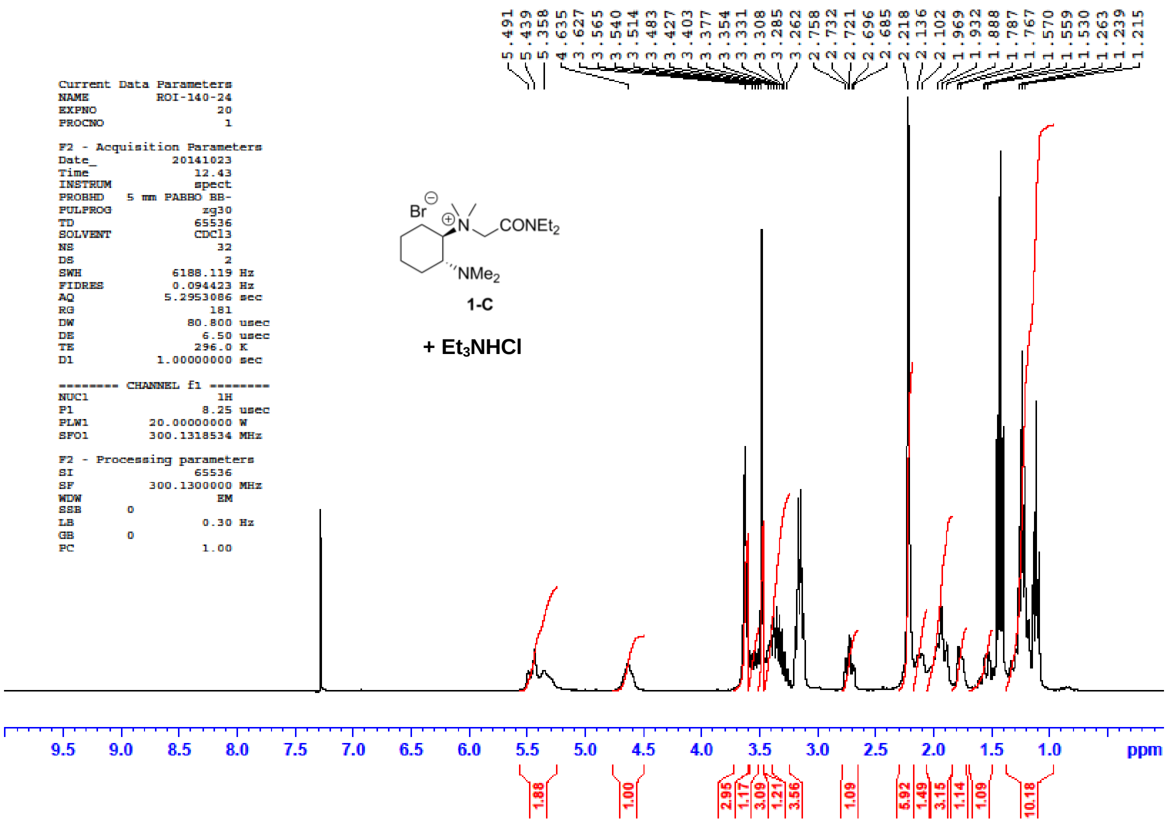
Current Data Parameters
NAME ROI-140-24
EXPNO 20
PROCNO 1
F2 - Acquisition Parameters
Date_ 20141023
Time 12.43
INSTRUM spect
PROBHD 5 mm PABBO BB-
PULPROG zg30
TD 65536
SOLVENT CDCl3
NS 32
DS 2
SWH 6188.119 Hz
FIDRES 0.094423 Hz
AQ 5.2953086 sec
RG 181
DW 80.800 usec
DE 6.50 usec
TE 296.0 K
D1 1.00000000 sec
======== CHANNEL f1 ========
NUC1 1H
P1 8.25 usec
PLW1 20.00000000 W
SFO1 300.1318534 MHz
F2 - Processing parameters
SI 65536
SF 300.1300000 MHz
WDW EM
SSB 0
LB 0.30 Hz
GB 0
PC 1.00
5.491 5.439 5.358 4.635 3.627 3.565 3.540 3.514 3.483 3.427 3.403 3.377 3.354 3.331 3.308 3.285 3.262 2.758 2.732 2.721 2.696 2.685 2.218 2.136 2.102 1.969 1.932 1.888 1.787 1.767 1.570 1.559 1.530 1.263 1.239 1.215
Br
N
CONEt2
NMe2
1-C
+ Et3NHCl
9.5 9.0 8.5 8.0 7.5 7.0 6.5 6.0 5.5 5.0 4.5 4.0 3.5 3.0 2.5 2.0 1.5 1.0 ppm
1.88 1.00 2.95 1.17 3.09 1.21 3.56 1.09 5.92 1.49 3.15 1.14 1.09 10.18

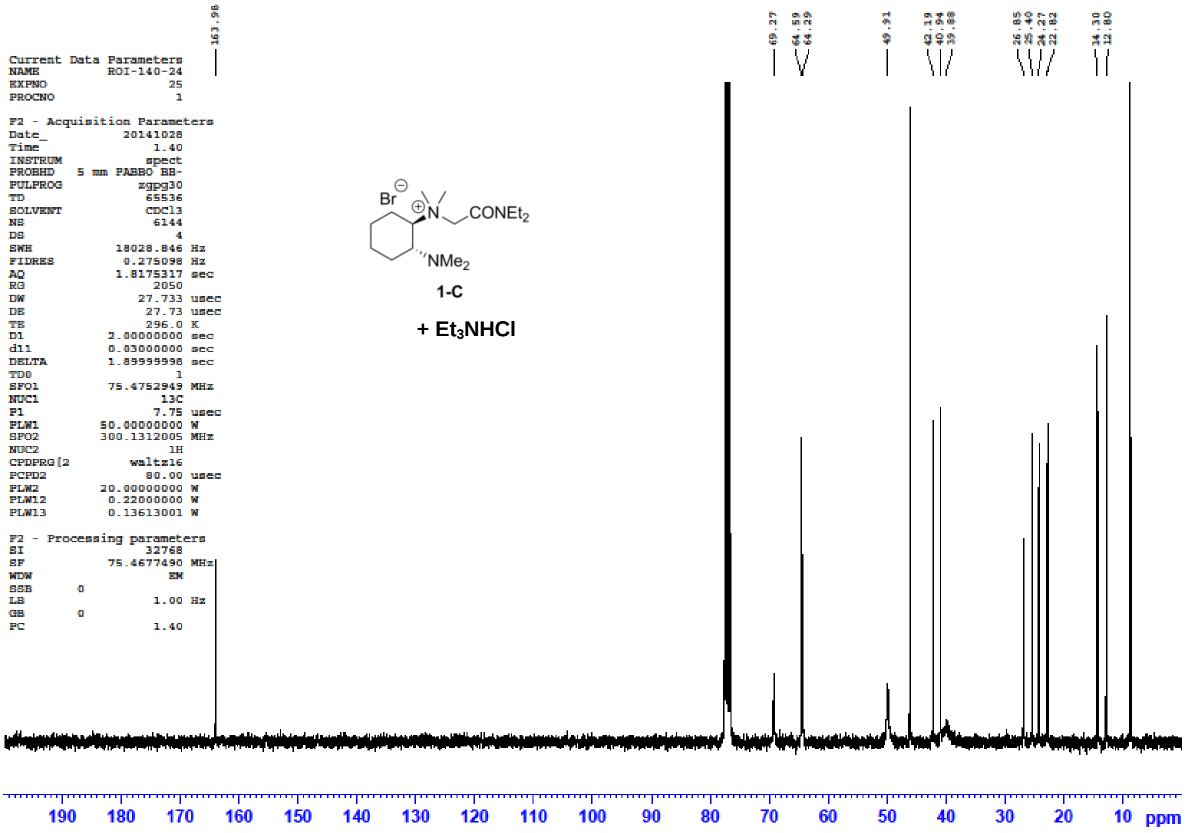
Current Data Parameters
NAME ROI-140-24
EXPNO 25
PROCNO 1
F2 - Acquisition Parameters
Date_ 20141028
Time 1.40
INSTRUM spect
PROBHD 5 mm PABBO BB-
PULPROG zgpg30
TD 65536
SOLVENT CDCl3
NS 6144
DS 4
SWH 18028.846 Hz
FIDRES 0.275098 Hz
AQ 1.8175317 sec
RG 2050
DW 27.733 usec
DE 27.73 usec
TE 296.0 K
D1 2.00000000 sec
d11 0.03000000 sec
DELTA 1.89999998 sec
TD0 1
SFO1 75.4752949 MHz
NUC1 13C
P1 7.75 usec
PLW1 50.00000000 W
SFO2 300.1312005 MHz
NUC2 1H
CPDPRG[2 waltz16
PCPD2 80.00 usec
PLW2 20.00000000 W
PLW12 0.22000000 W
PLW13 0.13613001 W
F2 - Processing parameters
SI 32768
SF 75.4677490 MHz
WDW EM
SSB 0
LB 1.00 Hz
GB 0
PC 1.40
163.98 69.27 64.59 64.29 49.91 42.19 40.94 39.88 26.85 25.40 24.27 22.82 14.30 12.80
Br
N
CONEt2
NMe2
1-C
+ Et3NHCl
190 180 170 160 150 140 130 120 110 100 90 80 70 60 50 40 30 20 10 ppm

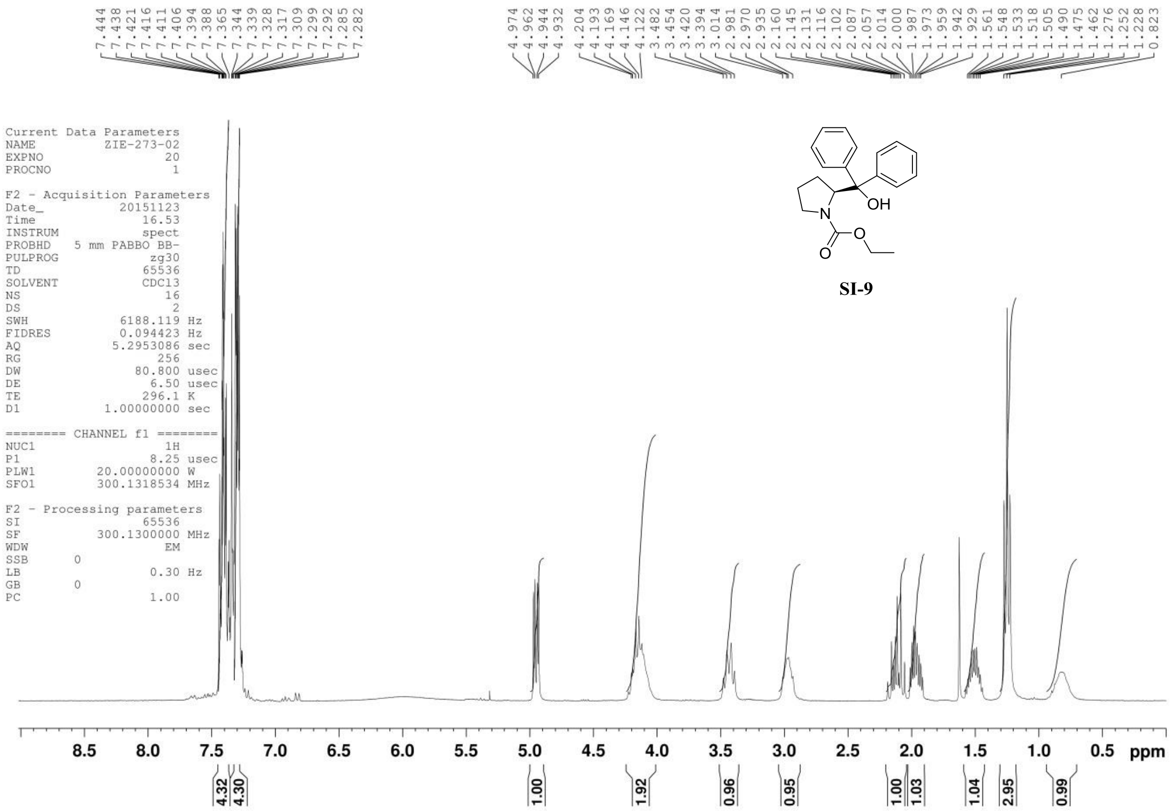

7.444
7.438
7.421
7.416
7.411
7.406
7.394
7.388
7.365
7.344
7.339
7.328
7.317
7.309
7.299
7.292
7.285
7.282
4.974
4.962
4.944
4.932
4.204
4.193
4.169
4.146
4.122
3.482
3.454
3.420
3.394
3.014
2.981
2.970
2.935
2.160
2.145
2.131
2.116
2.102
2.087
2.057
2.014
2.000
1.987
1.973
1.959
1.942
1.929
1.561
1.548
1.533
1.518
1.505
1.490
1.475
1.462
1.276
1.252
1.228
0.823
Current Data Parameters
NAME ZIE-273-02
EXPNO 20
PROCNO 1
F2 - Acquisition Parameters
Date_ 20151123
Time 16.53
INSTRUM spect
PROBHD 5 mm PABBO BB-
PULPROG zg30
TD 65536
SOLVENT CDCl3
NS 16
DS 2
SWH 6188.119 Hz
FIDRES 0.094423 Hz
AQ 5.2953086 sec
RG 256
DW 80.800 usec
DE 6.50 usec
TE 296.1 K
D1 1.00000000 sec
======== CHANNEL f1 ========
NUC1 1H
P1 8.25 usec
PLW1 20.00000000 W
SFO1 300.1318534 MHz
F2 - Processing parameters
SI 65536
SF 300.1300000 MHz
WDW EM
SSB 0
LB 0.30 Hz
GB 0
PC 1.00
8.5
8.0
7.5
7.0
6.5
6.0
5.5
5.0
4.5
4.0
3.5
3.0
2.5
2.0
1.5
1.0
0.5
ppm
4.32
4.30
1.00
1.92
0.96
0.95
1.00
1.03
1.04
2.95
0.99
N
OH
O
O

SI-9

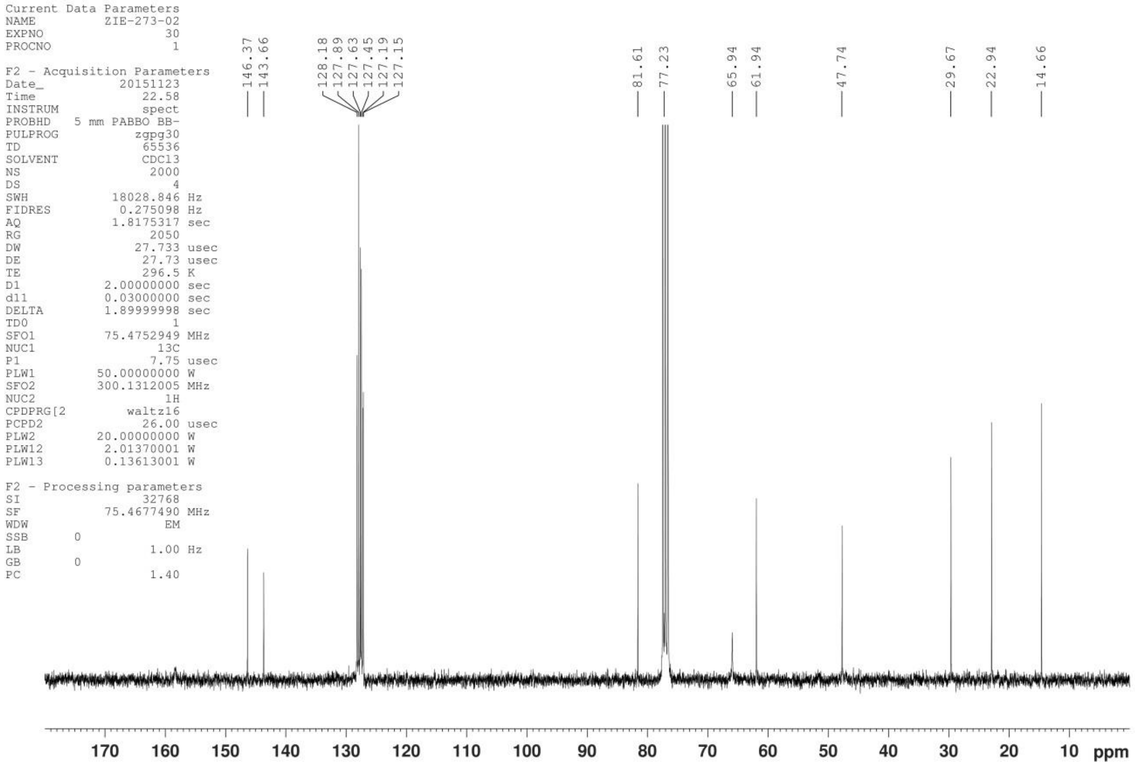

Current Data Parameters
NAME ZIE-273-02
EXPNO 30
PROCNO 1
F2 - Acquisition Parameters
Date_ 20151123
Time 22.58
INSTRUM spect
PROBHD 5 mm PABBO BB-
PULPROG zgpg30
TD 65536
SOLVENT CDCl3
NS 2000
DS 4
SWH 18028.846 Hz
FIDRES 0.275098 Hz
AQ 1.8175317 sec
RG 2050
DW 27.733 usec
DE 27.73 usec
TE 296.5 K
D1 2.00000000 sec
d11 0.03000000 sec
DELTA 1.89999998 sec
TD0 1
SFO1 75.4752949 MHz
NUC1 13C
P1 7.75 usec
PLW1 50.00000000 W
SFO2 300.1312005 MHz
NUC2 1H
CPDPRG[2 waltz16
PCPD2 26.00 usec
PLW2 20.00000000 W
PLW12 2.01370001 W
PLW13 0.13613001 W
F2 - Processing parameters
SI 32768
SF 75.4677490 MHz
WDW EM
SSB 0
LB 1.00 Hz
GB 0
PC 1.40
146.37
143.66
128.18
127.89
127.63
127.45
127.19
127.15
81.61
77.23
65.94
61.94
47.74
29.67
22.94
14.66
170
160
150
140
130
120
110
100
90
80
70
60
50
40
30
20
10
ppm

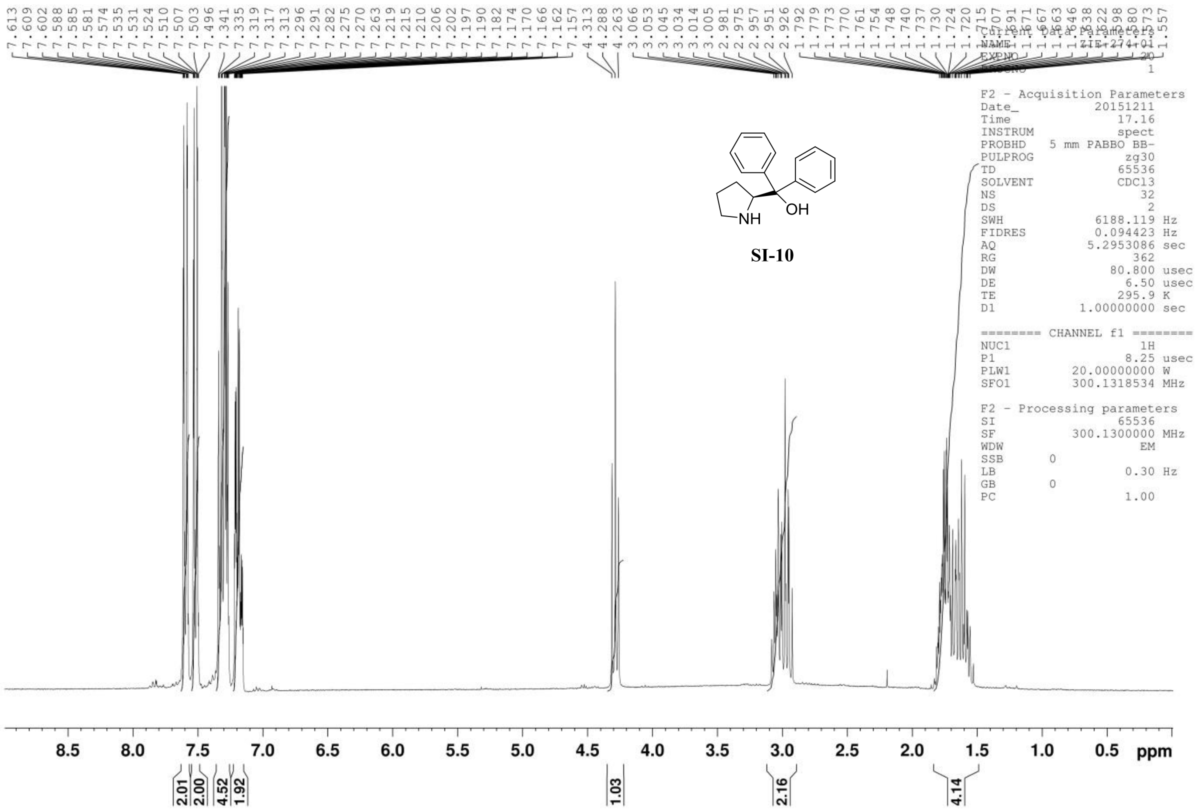
7.613 7.609 7.602 7.588 7.585 7.581 7.574 7.535 7.531 7.524 7.510 7.507 7.503 7.496 7.341 7.335 7.319 7.317 7.313 7.296 7.291 7.282 7.275 7.270 7.263 7.219 7.215 7.210 7.206 7.202 7.197 7.190 7.182 7.174 7.170 7.166 7.162 7.157 4.313 4.288 4.263 3.066 3.053 3.045 3.034 3.014 3.005 2.981 2.975 2.957 2.951 2.926 1.792 1.779 1.773 1.770 1.761 1.754 1.748 1.740 1.737 1.730 1.724 1.720 1.715 1.707
F2 - Acquisition Parameters
Date_ 20151211
Time 17.16
INSTRUM spect
PROBHD 5 mm PABBO BB-
PULPROG zg30
TD 65536
SOLVENT CDCl3
NS 32
DS 2
SWH 6188.119 Hz
FIDRES 0.094423 Hz
AQ 5.2953086 sec
RG 362
DW 80.800 usec
DE 6.50 usec
TE 295.9 K
D1 1.00000000 sec
======== CHANNEL f1 ========
NUC1 1H
P1 8.25 usec
PLW1 20.00000000 W
SFO1 300.1318534 MHz
F2 - Processing parameters
SI 65536
SF 300.1300000 MHz
WDW EM
SSB 0
LB 0.30 Hz
GB 0
PC 1.00
NH
OH
SI-10
8.5 8.0 7.5 7.0 6.5 6.0 5.5 5.0 4.5 4.0 3.5 3.0 2.5 2.0 1.5 1.0 0.5 ppm
2.01 2.00 4.52 1.92 1.03 2.16 4.14

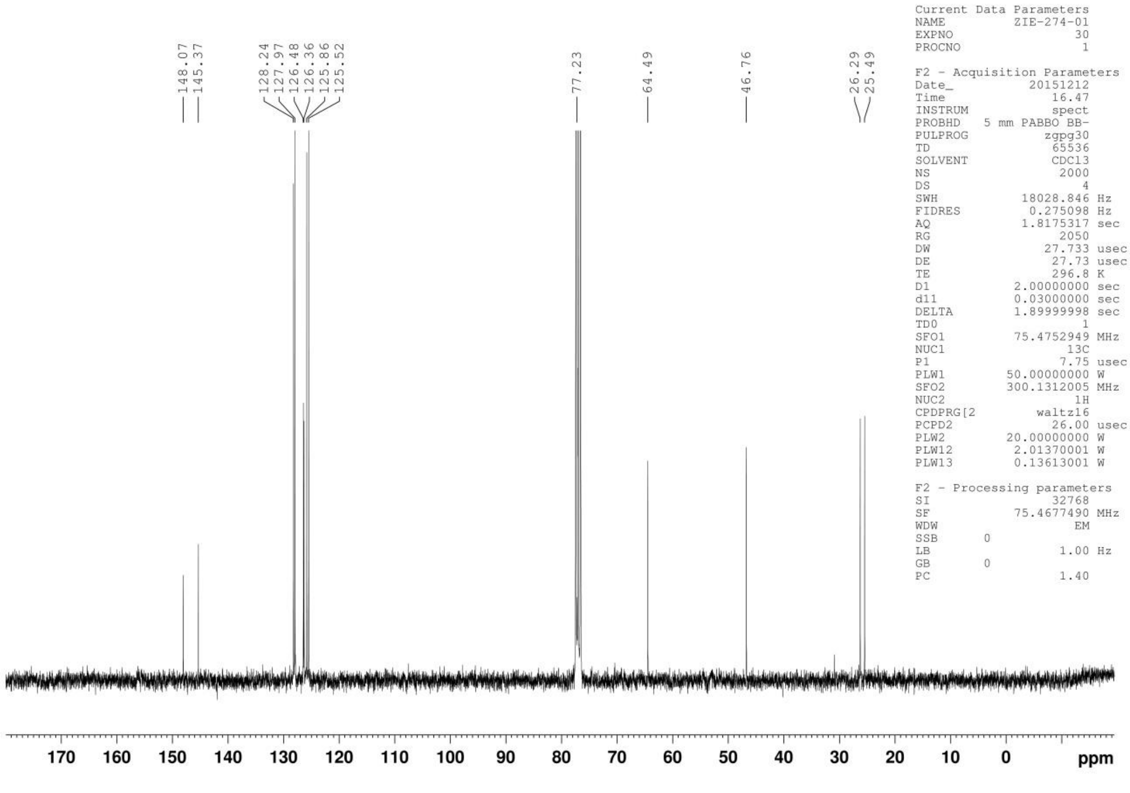
Current Data Parameters
NAME ZIE-274-01
EXPNO 30
PROCNO 1
148.07 145.37 128.24 127.97 126.48 126.36 125.86 125.52 77.23 64.49 46.76 26.29 25.49
F2 - Acquisition Parameters
Date_ 20151212
Time 16.47
INSTRUM spect
PROBHD 5 mm PABBO BB-
PULPROG zgpg30
TD 65536
SOLVENT CDCl3
NS 2000
DS 4
SWH 18028.846 Hz
FIDRES 0.275098 Hz
AQ 1.8175317 sec
RG 2050
DW 27.733 usec
DE 27.73 usec
TE 296.8 K
D1 2.00000000 sec
d11 0.03000000 sec
DELTA 1.89999998 sec
TD0 1
SFO1 75.4752949 MHz
NUC1 13C
P1 7.75 usec
PLW1 50.00000000 W
SFO2 300.1312005 MHz
NUC2 1H
CPDPRG[2 waltz16
PCPD2 26.00 usec
PLW2 20.00000000 W
PLW12 2.01370001 W
PLW13 0.13613001 W
F2 - Processing parameters
SI 32768
SF 75.4677490 MHz
WDW EM
SSB 0
LB 1.00 Hz
GB 0
PC 1.40
170 160 150 140 130 120 110 100 90 80 70 60 50 40 30 20 10 0 ppm

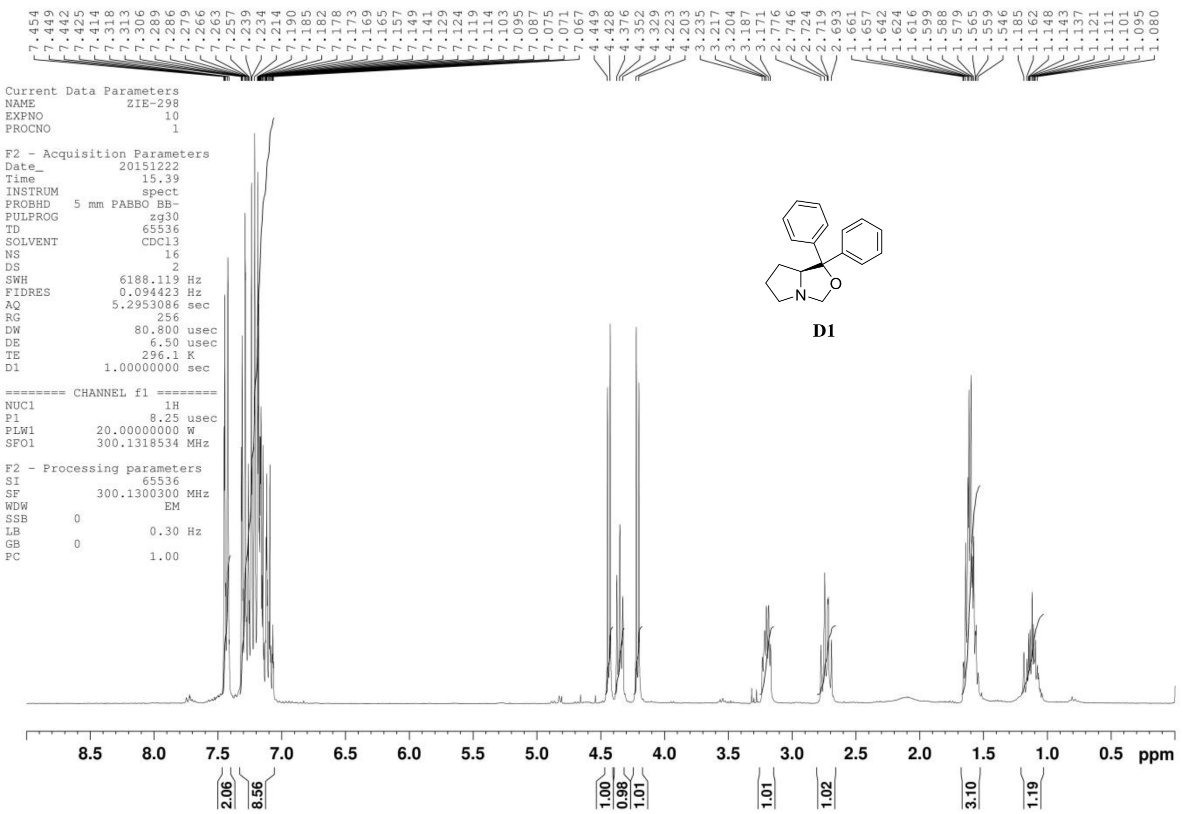
7.454 7.449 7.442 7.425 7.414 7.318 7.313 7.306 7.289 7.286 7.279 7.266 7.263 7.257 7.239 7.234 7.214 7.190 7.185 7.182 7.178 7.173 7.169 7.165 7.157 7.149 7.141 7.129 7.124 7.119 7.114 7.103 7.095 7.087 7.075 7.071 7.067 4.449 4.428 4.376 4.352 4.329 4.223 4.203 3.235 3.217 3.204 3.187 3.171 2.776 2.746 2.724 2.719 2.693 1.661 1.657 1.642 1.624 1.616 1.599 1.588 1.579 1.565 1.559 1.546 1.185 1.162 1.148 1.143 1.137 1.121 1.111 1.101 1.095 1.080
Current Data Parameters
NAME ZIE-298
EXPNO 10
PROCNO 1
F2 - Acquisition Parameters
Date_ 20151222
Time 15.39
INSTRUM spect
PROBHD 5 mm PABBO BB-
PULPROG zg30
TD 65536
SOLVENT CDCl3
NS 16
DS 2
SWH 6188.119 Hz
FIDRES 0.094423 Hz
AQ 5.2953086 sec
RG 256
DW 80.800 usec
DE 6.50 usec
TE 296.1 K
D1 1.00000000 sec
======== CHANNEL f1 ========
NUC1 1H
P1 8.25 usec
PLW1 20.00000000 W
SFO1 300.1318534 MHz
F2 - Processing parameters
SI 65536
SF 300.1300300 MHz
WDW EM
SSB 0
LB 0.30 Hz
GB 0
PC 1.00
8.5 8.0 7.5 7.0 6.5 6.0 5.5 5.0 4.5 4.0 3.5 3.0 2.5 2.0 1.5 1.0 0.5 ppm
2.06 8.56 1.00 0.98 1.01 1.01 1.02 3.10 1.19

D1

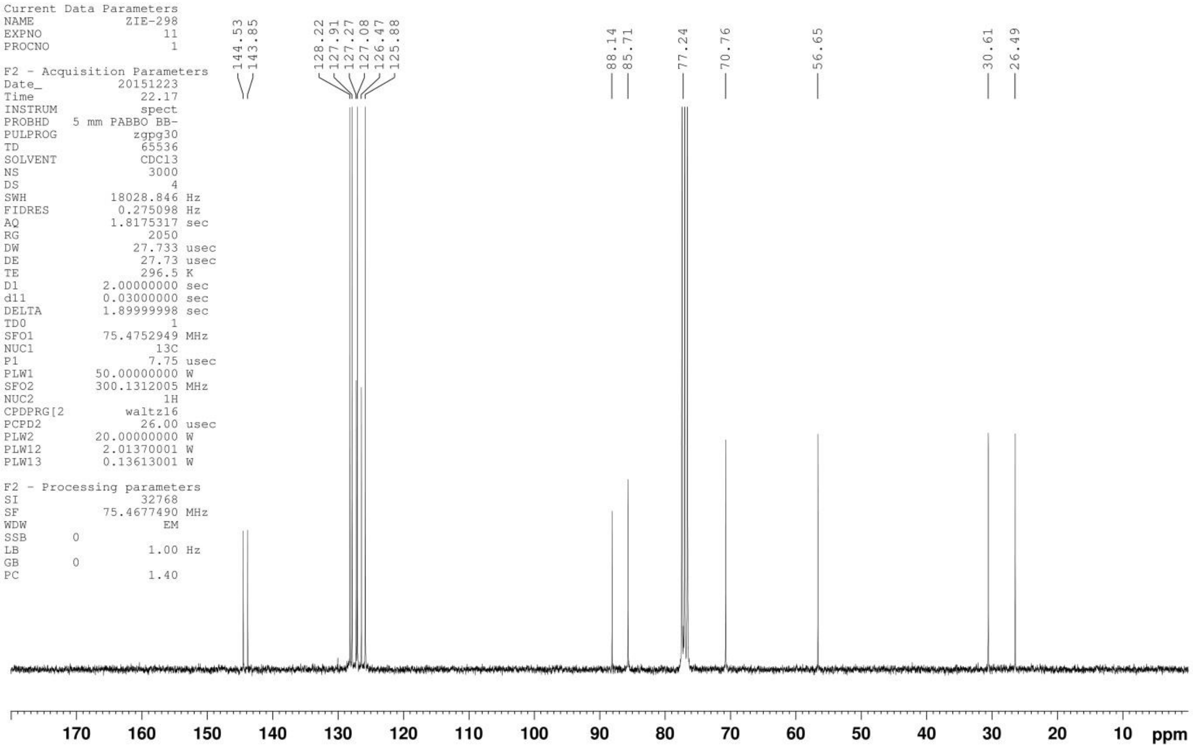
144.53 143.85 128.22 127.91 127.27 127.08 126.47 125.88 88.14 85.71 77.24 70.76 56.65 30.61 26.49
Current Data Parameters
NAME ZIE-298
EXPNO 11
PROCNO 1
F2 - Acquisition Parameters
Date_ 20151223
Time 22.17
INSTRUM spect
PROBHD 5 mm PABBO BB-
PULPROG zgpg30
TD 65536
SOLVENT CDCl3
NS 3000
DS 4
SWH 18028.846 Hz
FIDRES 0.275098 Hz
AQ 1.8175317 sec
RG 2050
DW 27.733 usec
DE 27.73 usec
TE 296.5 K
D1 2.00000000 sec
d11 0.03000000 sec
DELTA 1.89999998 sec
TD0 1
SFO1 75.4752949 MHz
NUC1 13C
P1 7.75 usec
PLW1 50.00000000 W
SFO2 300.1312005 MHz
NUC2 1H
CPDPRG[2 waltz16
PCPD2 26.00 usec
PLW2 20.00000000 W
PLW12 2.01370001 W
PLW13 0.13613001 W
F2 - Processing parameters
SI 32768
SF 75.4677490 MHz
WDW EM
SSB 0
LB 1.00 Hz
GB 0
PC 1.40
170 160 150 140 130 120 110 100 90 80 70 60 50 40 30 20 10 ppm

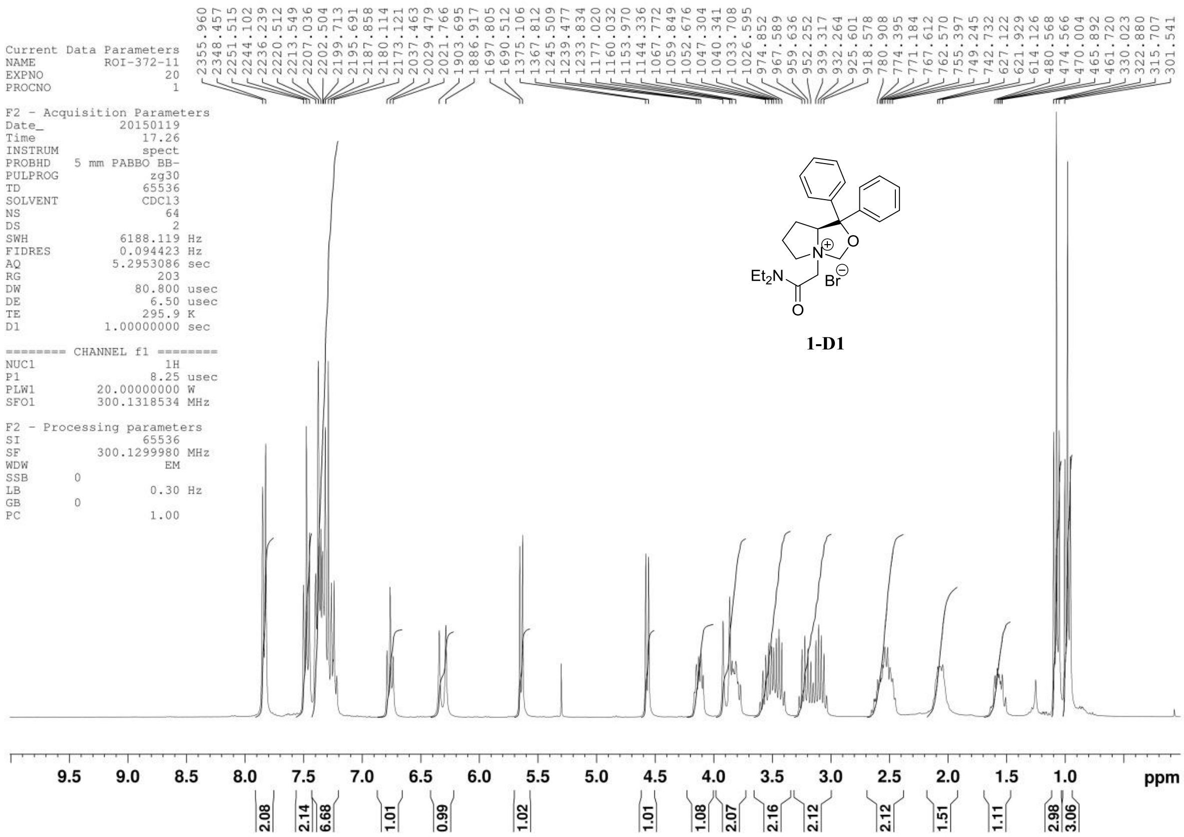
Current Data Parameters
NAME ROI-372-11
EXPNO 20
PROCNO 1
F2 - Acquisition Parameters
Date_ 20150119
Time 17.26
INSTRUM spect
PROBHD 5 mm PABBO BB-
PULPROG zg30
TD 65536
SOLVENT CDCl3
NS 64
DS 2
SWH 6188.119 Hz
FIDRES 0.094423 Hz
AQ 5.2953086 sec
RG 203
DW 80.800 usec
DE 6.50 usec
TE 295.9 K
D1 1.00000000 sec
======== CHANNEL f1 ========
NUC1 1H
P1 8.25 usec
PLW1 20.00000000 W
SFO1 300.1318534 MHz
F2 - Processing parameters
SI 65536
SF 300.1299980 MHz
WDW EM
SSB 0
LB 0.30 Hz
GB 0
PC 1.00
2355.960 2348.457 2251.515 2244.102 2236.239 2220.512 2213.549 2207.036 2202.504 2199.713 2195.691 2187.858 2180.114 2173.121 2037.463 2029.479 2021.766 1903.695 1886.917 1697.805 1690.512 1375.106 1367.812 1245.509 1239.477 1233.834 1177.020 1160.032 1153.970 1144.336 1067.772 1059.849 1052.676 1047.304 1040.341 1033.708 1026.595 974.852 967.589 959.636 952.252 939.317 932.264 925.601 918.578 780.908 774.395 771.184 767.612 762.570 755.397 749.245 742.732 627.122 621.929 614.126 480.568 474.566 470.004 465.892 461.720 330.023 322.880 315.707 301.541
Et2N
O
N
O
Br
1-D1
9.5 9.0 8.5 8.0 7.5 7.0 6.5 6.0 5.5 5.0 4.5 4.0 3.5 3.0 2.5 2.0 1.5 1.0 ppm
2.08 2.14 6.68 1.01 0.99 1.02 1.01 1.08 2.07 2.16 2.12 2.12 1.51 1.11 2.98 3.06

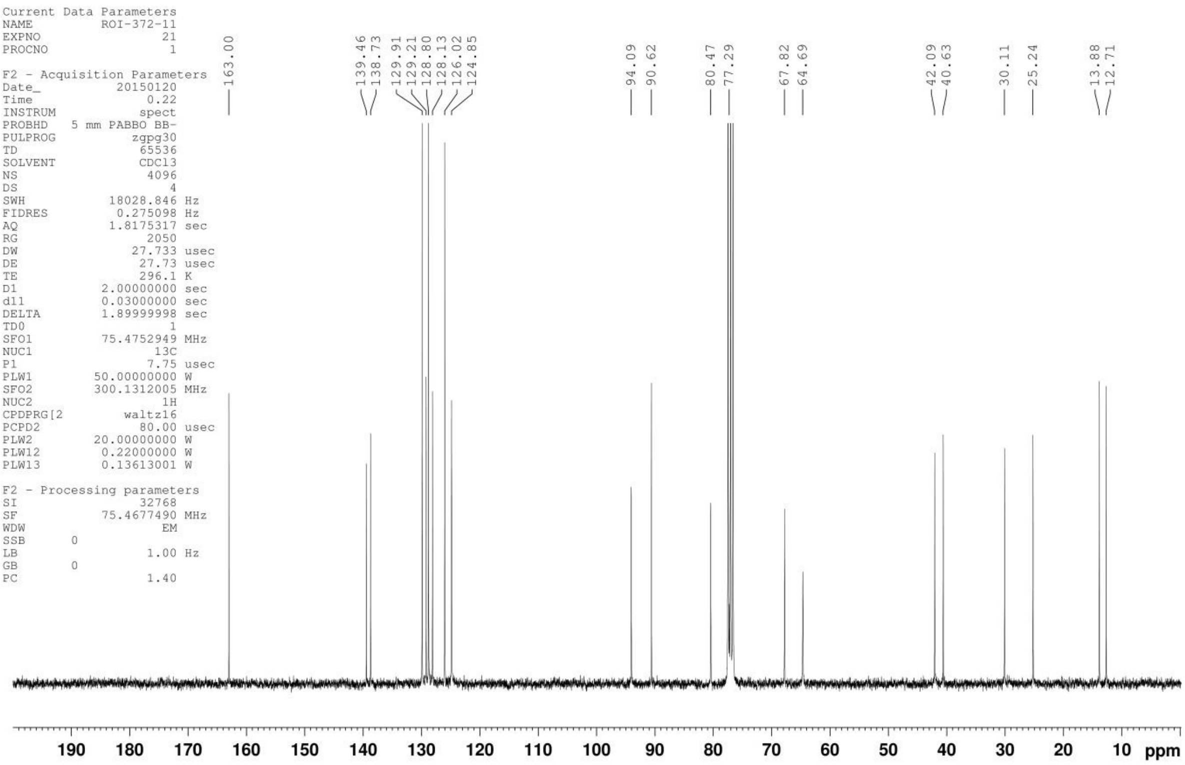
Current Data Parameters
NAME ROI-372-11
EXPNO 21
PROCNO 1
F2 - Acquisition Parameters
Date_ 20150120
Time 0.22
INSTRUM spect
PROBHD 5 mm PABBO BB-
PULPROG zgpg30
TD 65536
SOLVENT CDCl3
NS 4096
DS 4
SWH 18028.846 Hz
FIDRES 0.275098 Hz
AQ 1.8175317 sec
RG 2050
DW 27.733 usec
DE 27.73 usec
TE 296.1 K
D1 2.00000000 sec
d11 0.03000000 sec
DELTA 1.89999998 sec
TD0 1
SFO1 75.4752949 MHz
NUC1 13C
P1 7.75 usec
PLW1 50.00000000 W
SFO2 300.1312005 MHz
NUC2 1H
CPDPRG[2 waltz16
PCPD2 80.00 usec
PLW2 20.00000000 W
PLW12 0.22000000 W
PLW13 0.13613001 W
F2 - Processing parameters
SI 32768
SF 75.4677490 MHz
WDW EM
SSB 0
LB 1.00 Hz
GB 0
PC 1.40
163.00 139.46 138.73 129.91 129.21 128.80 128.13 126.02 124.85 94.09 90.62 80.47 77.29 67.82 64.69 42.09 40.63 30.11 25.24 13.88 12.71
190 180 170 160 150 140 130 120 110 100 90 80 70 60 50 40 30 20 10 ppm

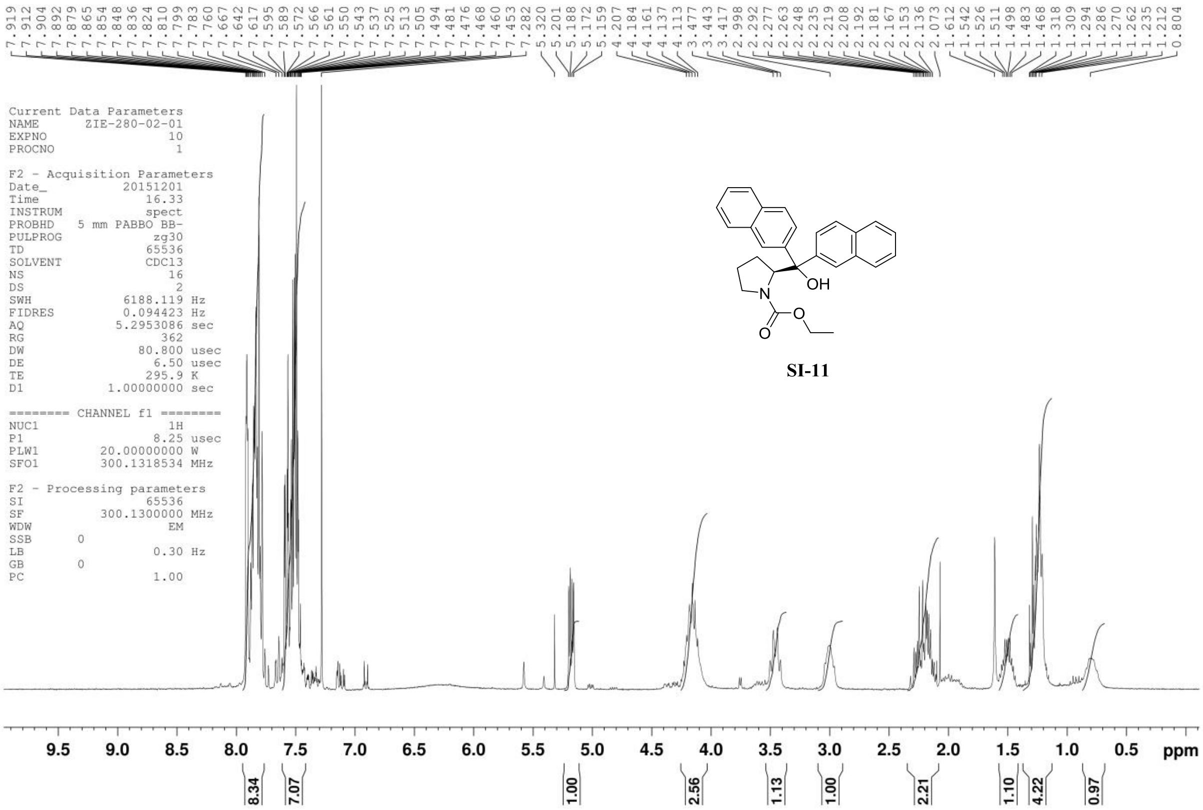
7.919 7.912 7.904 7.892 7.879 7.865 7.854 7.848 7.836 7.824 7.810 7.799 7.783 7.760 7.667 7.642 7.617 7.595 7.589 7.572 7.566 7.561 7.550 7.543 7.537 7.525 7.513 7.505 7.494 7.481 7.476 7.468 7.460 7.453 7.282 5.320 5.201 5.188 5.172 5.159 4.207 4.184 4.161 4.137 4.113 3.477 3.443 3.417 2.998 2.292 2.277 2.263 2.248 2.235 2.219 2.208 2.192 2.181 2.167 2.153 2.136 2.073 1.612 1.542 1.526 1.511 1.498 1.483 1.468 1.318 1.309 1.294 1.286 1.270 1.262 1.235 1.212 0.804
Current Data Parameters
NAME ZIE-280-02-01
EXPNO 10
PROCNO 1
F2 - Acquisition Parameters
Date_ 20151201
Time 16.33
INSTRUM spect
PROBHD 5 mm PABBO BB-
PULPROG zg30
TD 65536
SOLVENT CDCl3
NS 16
DS 2
SWH 6188.119 Hz
FIDRES 0.094423 Hz
AQ 5.2953086 sec
RG 362
DW 80.800 usec
DE 6.50 usec
TE 295.9 K
D1 1.00000000 sec
======== CHANNEL f1 ========
NUC1 1H
P1 8.25 usec
PLW1 20.00000000 W
SFO1 300.1318534 MHz
F2 - Processing parameters
SI 65536
SF 300.1300000 MHz
WDW EM
SSB 0
LB 0.30 Hz
GB 0
PC 1.00
OH
N
O
O
9.5 9.0 8.5 8.0 7.5 7.0 6.5 6.0 5.5 5.0 4.5 4.0 3.5 3.0 2.5 2.0 1.5 1.0 0.5 ppm
8.34 7.07 1.00 2.56 1.13 1.00 2.21 1.10 4.22 0.97

SI-11

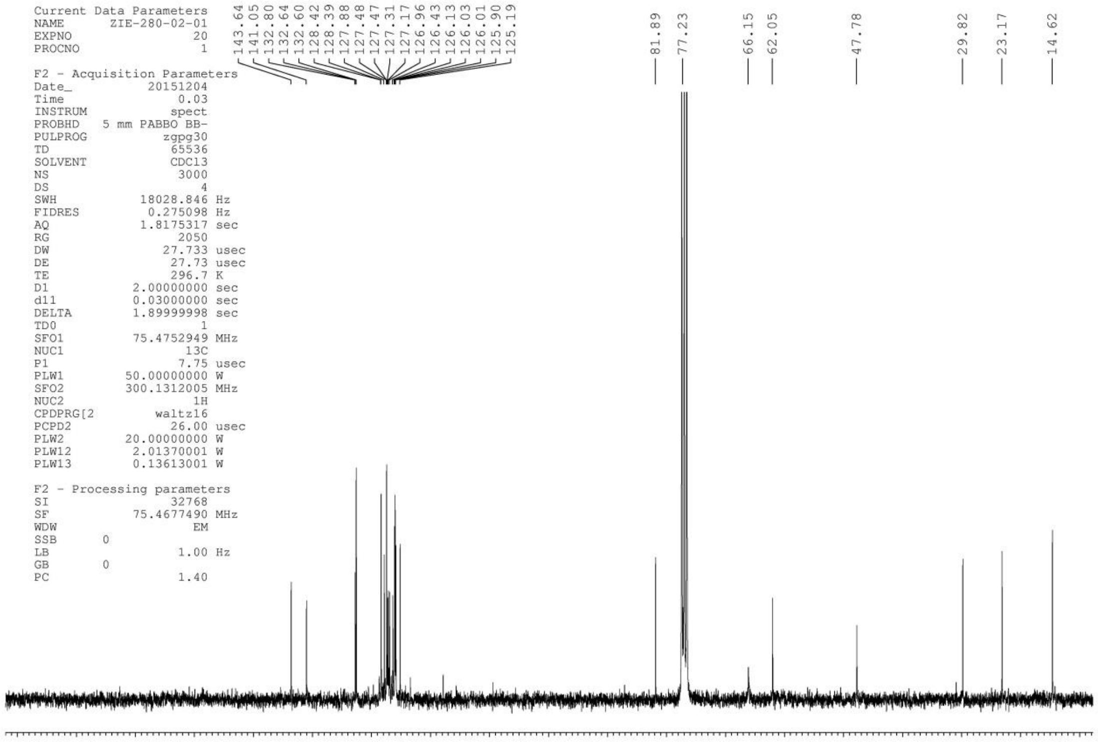
Current Data Parameters
NAME ZIE-280-02-01
EXPNO 20
PROCNO 1
F2 - Acquisition Parameters
Date_ 20151204
Time 0.03
INSTRUM spect
PROBHD 5 mm PABBO BB-
PULPROG zgpg30
TD 65536
SOLVENT CDCl3
NS 3000
DS 4
SWH 18028.846 Hz
FIDRES 0.275098 Hz
AQ 1.8175317 sec
RG 2050
DW 27.733 usec
DE 27.73 usec
TE 296.7 K
D1 2.00000000 sec
d11 0.03000000 sec
DELTA 1.89999998 sec
TD0 1
SFO1 75.4752949 MHz
NUC1 13C
P1 7.75 usec
PLW1 50.00000000 W
SFO2 300.1312005 MHz
NUC2 1H
CPDPRG[2 waltz16
PCPD2 26.00 usec
PLW2 20.00000000 W
PLW12 2.01370001 W
PLW13 0.13613001 W
F2 - Processing parameters
SI 32768
SF 75.4677490 MHz
WDW EM
SSB 0
LB 1.00 Hz
GB 0
PC 1.40
143.64 141.05 132.80 132.64 132.60 128.42 128.39 127.88 127.48 127.47 127.31 127.17 126.96 126.43 126.13 126.03 126.01 125.90 125.19
81.89 77.23 66.15 62.05 47.78 29.82 23.17 14.62

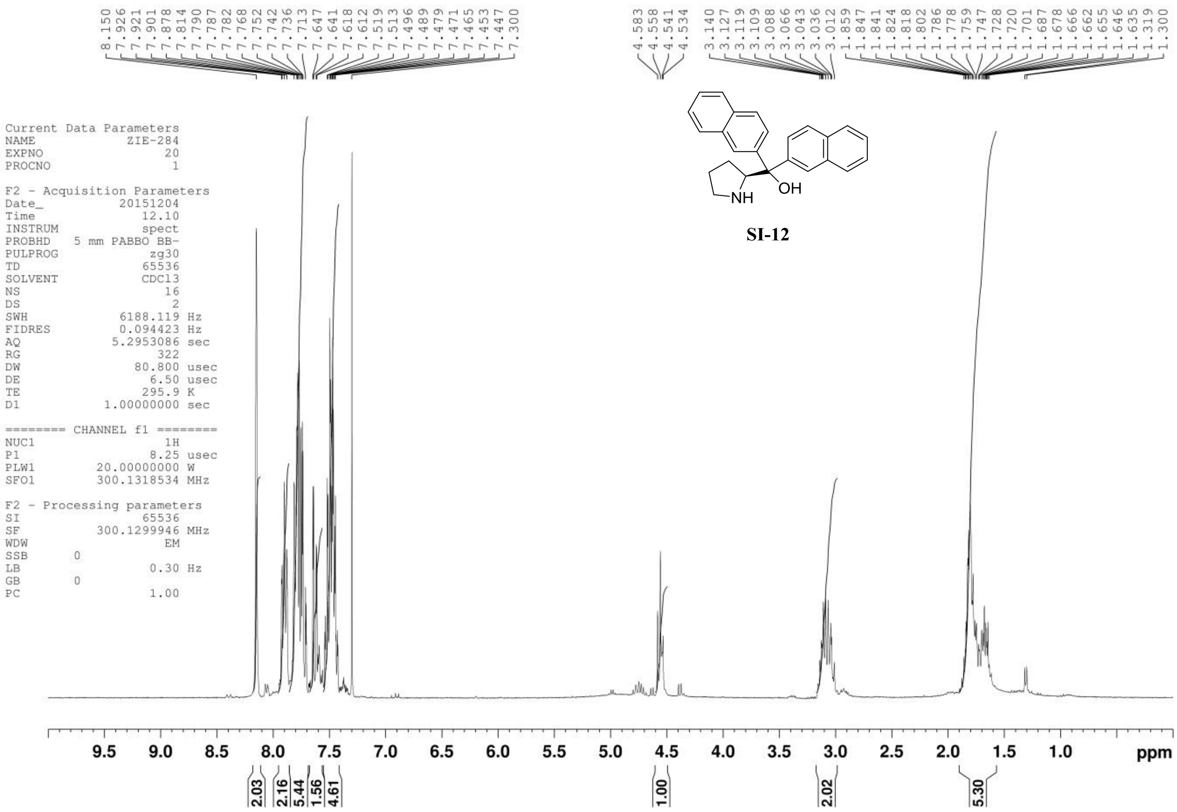
8.150 7.926 7.921 7.901 7.878 7.814 7.790 7.787 7.782 7.768 7.752 7.742 7.736 7.713 7.647 7.641 7.618 7.612 7.519 7.513 7.496 7.489 7.479 7.471 7.465 7.453 7.447 7.300
4.583 4.558 4.541 4.534
3.140 3.127 3.119 3.109 3.088 3.066 3.043 3.036 3.012
1.859 1.847 1.841 1.824 1.818 1.802 1.786 1.778 1.759 1.747 1.728 1.720 1.701 1.687 1.678 1.666 1.662 1.655 1.646 1.635 1.319 1.300
Current Data Parameters
NAME ZIE-284
EXPNO 20
PROCNO 1
F2 - Acquisition Parameters
Date_ 20151204
Time 12.10
INSTRUM spect
PROBHD 5 mm PABBO BB-
PULPROG zg30
TD 65536
SOLVENT CDCl3
NS 16
DS 2
SWH 6188.119 Hz
FIDRES 0.094423 Hz
AQ 5.2953086 sec
RG 322
DW 80.800 usec
DE 6.50 usec
TE 295.9 K
D1 1.00000000 sec
======== CHANNEL f1 ========
NUC1 1H
P1 8.25 usec
PLW1 20.00000000 W
SFO1 300.1318534 MHz
F2 - Processing parameters
SI 65536
SF 300.1299946 MHz
WDW EM
SSB 0
LB 0.30 Hz
GB 0
PC 1.00
9.5 9.0 8.5 8.0 7.5 7.0 6.5 6.0 5.5 5.0 4.5 4.0 3.5 3.0 2.5 2.0 1.5 1.0 ppm
2.03 2.16 5.44 1.56 4.61 1.00 2.02 5.30
NH
OH

SI-12

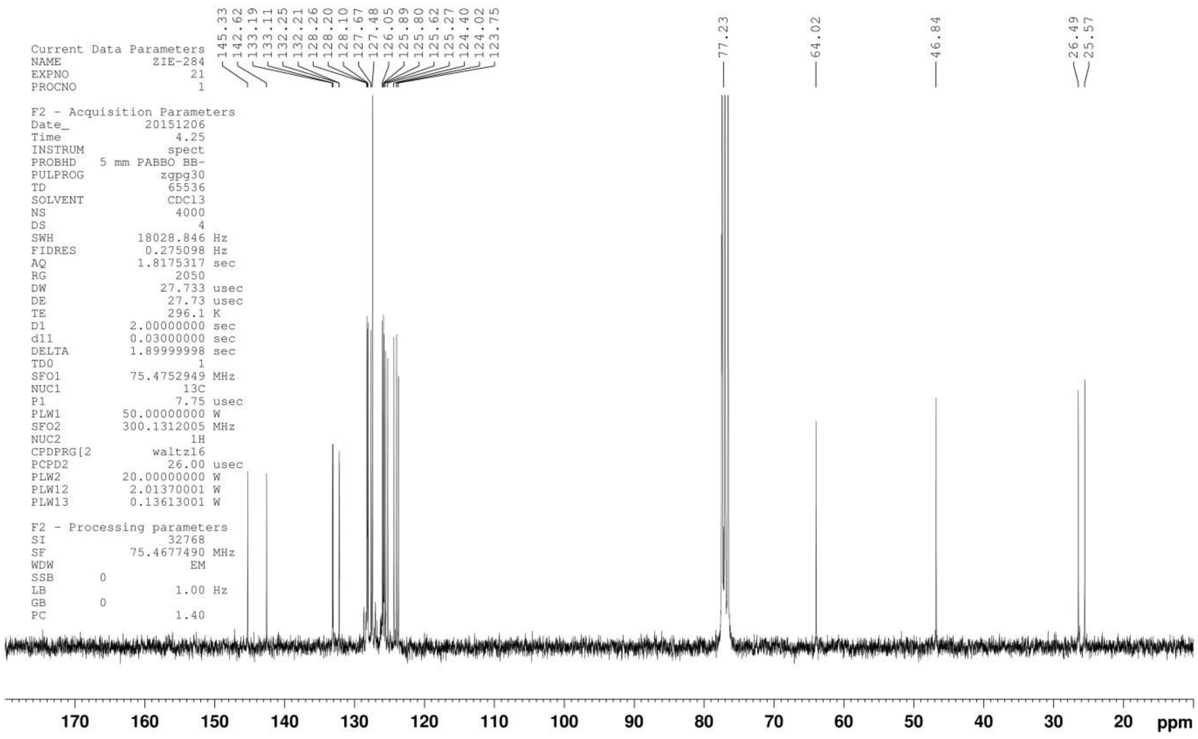
145.33 142.62 133.19 133.11 132.25 132.21 128.26 128.20 128.10 127.67 127.48 126.05 125.89 125.80 125.62 125.27 124.40 124.02 123.75
77.23
64.02
46.84
26.49 25.57
Current Data Parameters
NAME ZIE-284
EXPNO 21
PROCNO 1
F2 - Acquisition Parameters
Date_ 20151206
Time 4.25
INSTRUM spect
PROBHD 5 mm PABBO BB-
PULPROG zgpg30
TD 65536
SOLVENT CDCl3
NS 4000
DS 4
SWH 18028.846 Hz
FIDRES 0.275098 Hz
AQ 1.8175317 sec
RG 2050
DW 27.733 usec
DE 27.73 usec
TE 296.1 K
D1 2.00000000 sec
d11 0.03000000 sec
DELTA 1.89999998 sec
TD0 1
SFO1 75.4752949 MHz
NUC1 13C
P1 7.75 usec
PLW1 50.00000000 W
SFO2 300.1312005 MHz
NUC2 1H
CPDPRG[2 waltz16
PCPD2 26.00 usec
PLW2 20.00000000 W
PLW12 2.01370001 W
PLW13 0.13613001 W
F2 - Processing parameters
SI 32768
SF 75.4677490 MHz
WDW EM
SSB 0
LB 1.00 Hz
GB 0
PC 1.40
170 160 150 140 130 120 110 100 90 80 70 60 50 40 30 20 ppm

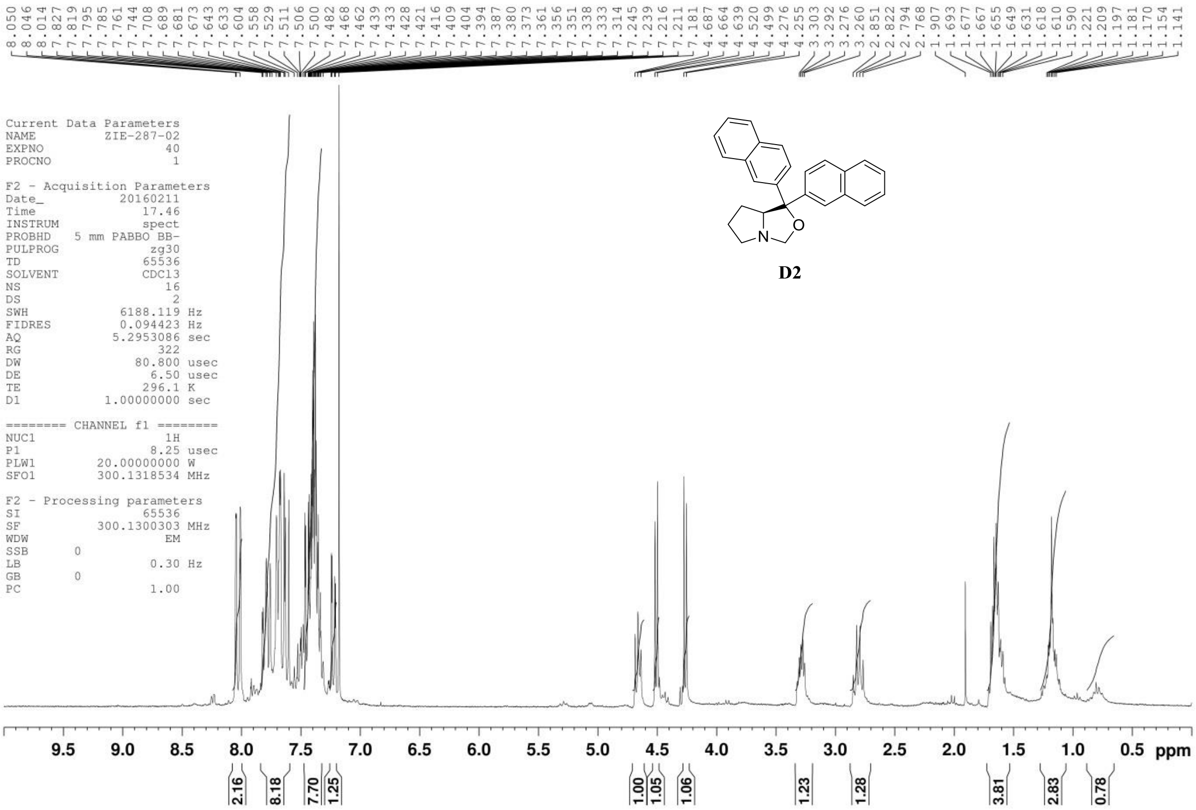
8.050 8.046 8.014 7.827 7.819 7.795 7.785 7.761 7.744 7.708 7.689 7.681 7.673 7.643 7.633 7.604 7.558 7.529 7.511 7.506 7.500 7.482 7.468 7.462 7.439 7.433 7.428 7.421 7.416 7.409 7.404 7.394 7.387 7.380 7.373 7.361 7.356 7.351 7.338 7.333 7.314 7.245 7.239 7.216 7.211 7.181 4.687 4.664 4.639 4.520 4.499 4.276 4.255 3.303 3.292 3.276 3.260 2.851 2.822 2.794 2.768 1.907 1.693 1.677 1.667 1.655 1.649 1.631 1.618 1.610 1.590 1.221 1.209 1.197 1.181 1.170 1.154 1.141
Current Data Parameters
NAME ZIE-287-02
EXPNO 40
PROCNO 1
F2 - Acquisition Parameters
Date_ 20160211
Time 17.46
INSTRUM spect
PROBHD 5 mm PABBO BB-
PULPROG zg30
TD 65536
SOLVENT CDCl3
NS 16
DS 2
SWH 6188.119 Hz
FIDRES 0.094423 Hz
AQ 5.2953086 sec
RG 322
DW 80.800 usec
DE 6.50 usec
TE 296.1 K
D1 1.00000000 sec
======== CHANNEL f1 ========
NUC1 1H
P1 8.25 usec
PLW1 20.00000000 W
SFO1 300.1318534 MHz
F2 - Processing parameters
SI 65536
SF 300.1300303 MHz
WDW EM
SSB 0
LB 0.30 Hz
GB 0
PC 1.00
N
O
D2
9.5 9.0 8.5 8.0 7.5 7.0 6.5 6.0 5.5 5.0 4.5 4.0 3.5 3.0 2.5 2.0 1.5 1.0 0.5 ppm
2.16 8.18 7.70 1.25 1.00 1.05 1.06 1.23 1.28 3.81 2.83 0.78

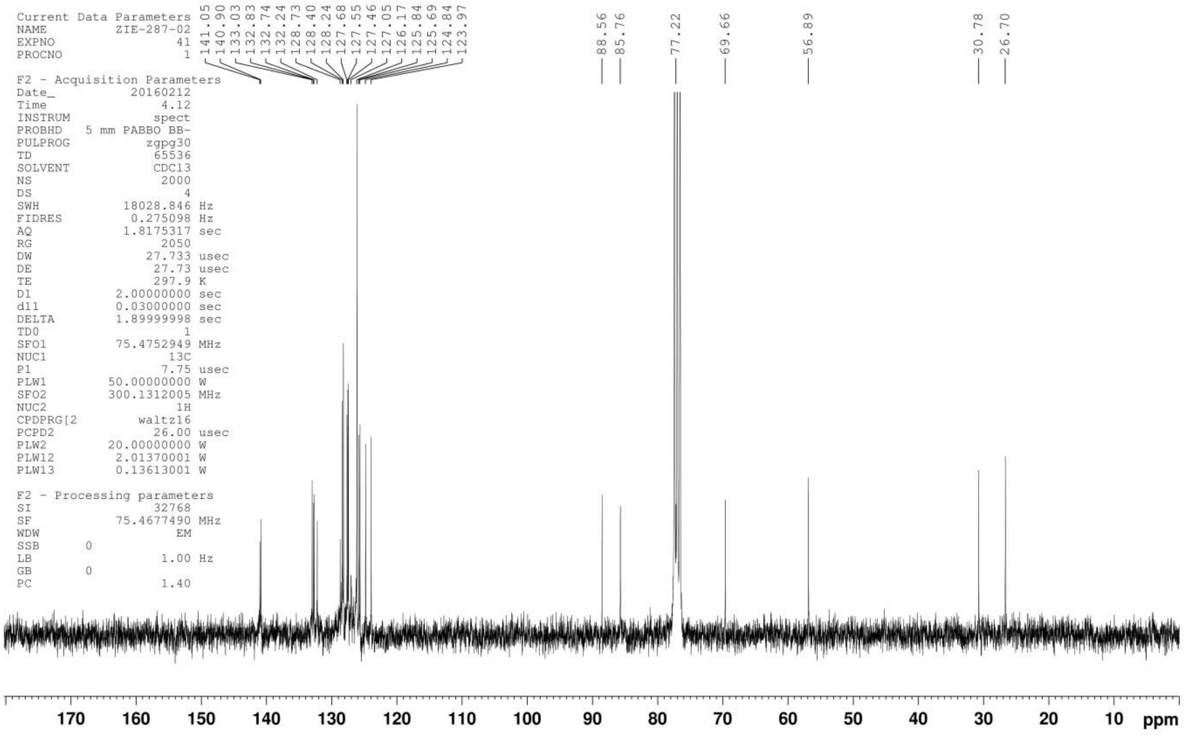
Current Data Parameters
NAME ZIE-287-02
EXPNO 41
PROCNO 1
141.05 140.90 133.03 132.83 132.74 132.24 128.73 128.40 128.24 127.68 127.55 127.46 127.05 126.17 125.84 125.69 124.84 123.97
88.56 85.76 77.22 69.66 56.89 30.78 26.70
F2 - Acquisition Parameters
Date_ 20160212
Time 4.12
INSTRUM spect
PROBHD 5 mm PABBO BB-
PULPROG zgpg30
TD 65536
SOLVENT CDCl3
NS 2000
DS 4
SWH 18028.846 Hz
FIDRES 0.275098 Hz
AQ 1.8175317 sec
RG 2050
DW 27.733 usec
DE 27.73 usec
TE 297.9 K
D1 2.00000000 sec
d11 0.03000000 sec
DELTA 1.89999998 sec
TD0 1
SFO1 75.4752949 MHz
NUC1 13C
P1 7.75 usec
PLW1 50.00000000 W
SFO2 300.1312005 MHz
NUC2 1H
CPDPRG[2 waltz16
PCPD2 26.00 usec
PLW2 20.00000000 W
PLW12 2.01370001 W
PLW13 0.13613001 W
F2 - Processing parameters
SI 32768
SF 75.4677490 MHz
WDW EM
SSB 0
LB 1.00 Hz
GB 0
PC 1.40
170 160 150 140 130 120 110 100 90 80 70 60 50 40 30 20 10 ppm

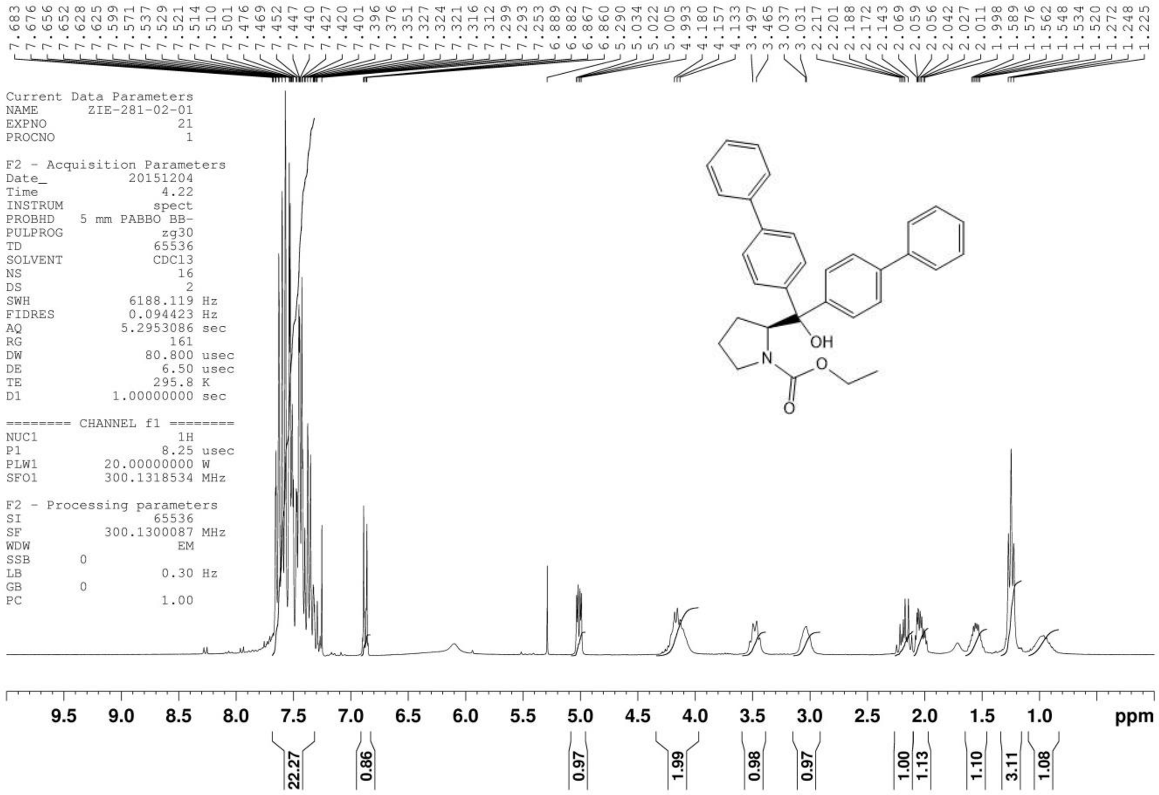
7.683 7.676 7.656 7.652 7.628 7.625 7.599 7.571 7.539 7.529 7.521 7.514 7.510 7.501 7.476 7.469 7.452 7.447 7.440 7.427 7.420 7.401 7.396 7.376 7.351 7.327 7.324 7.321 7.316 7.312 7.299 7.293 7.253 6.889 6.882 6.867 6.860 5.290 5.034 5.022 5.005 4.993 4.180 4.157 4.133 3.497 3.465 3.037 3.031 2.217 2.201 2.188 2.172 2.143 2.069 2.059 2.056 2.042 2.027 2.011 1.998 1.589 1.576 1.562 1.548 1.534 1.520 1.272 1.248 1.225
Current Data Parameters
NAME ZIE-281-02-01
EXPNO 21
PROCNO 1
F2 - Acquisition Parameters
Date_ 20151204
Time 4.22
INSTRUM spect
PROBHD 5 mm PABBO BB-
PULPROG zg30
TD 65536
SOLVENT CDCl3
NS 16
DS 2
SWH 6188.119 Hz
FIDRES 0.094423 Hz
AQ 5.2953086 sec
RG 161
DW 80.800 usec
DE 6.50 usec
TE 295.8 K
D1 1.00000000 sec
======== CHANNEL f1 ========
NUC1 1H
P1 8.25 usec
PLW1 20.00000000 W
SFO1 300.1318534 MHz
F2 - Processing parameters
SI 65536
SF 300.1300087 MHz
WDW EM
SSB 0
LB 0.30 Hz
GB 0
PC 1.00
OH
N
O
O
9.5 9.0 8.5 8.0 7.5 7.0 6.5 6.0 5.5 5.0 4.5 4.0 3.5 3.0 2.5 2.0 1.5 1.0 ppm
22.27 0.86 0.97 1.99 0.98 0.97 1.00 1.13 1.10 3.11 1.08

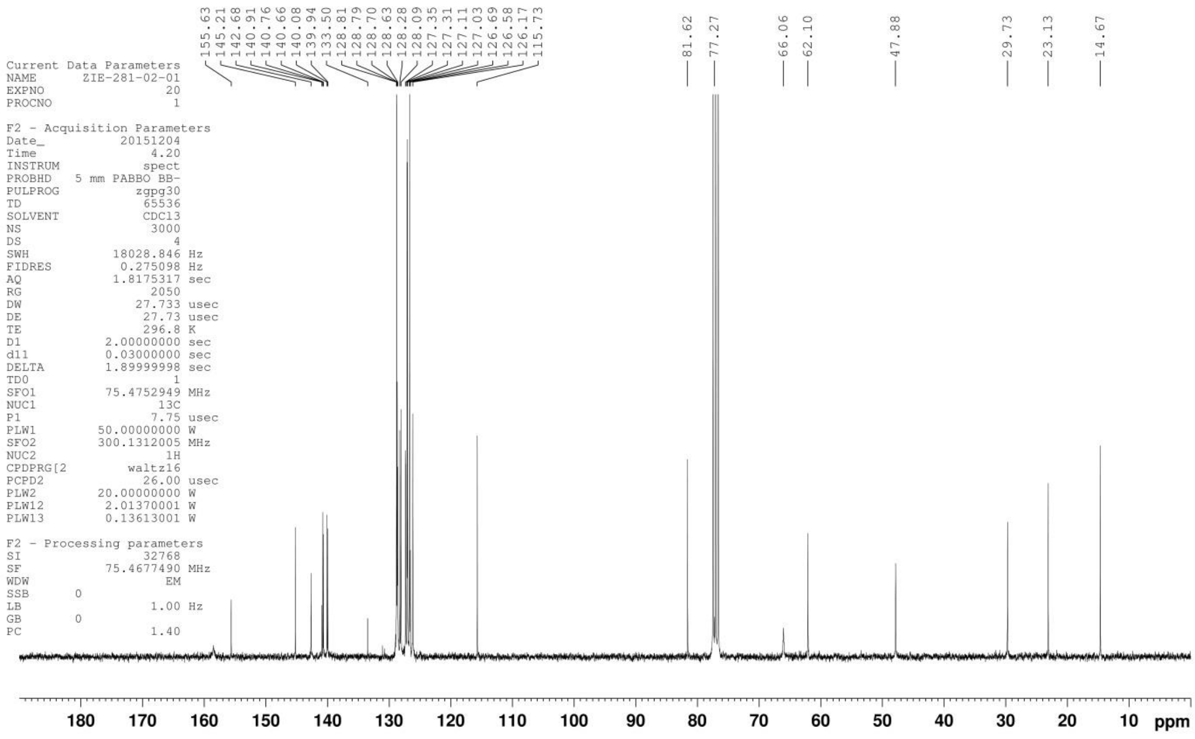
155.63 145.21 142.68 140.91 140.76 140.66 140.08 139.94 133.50 128.81 128.79 128.70 128.63 128.28 128.09 127.35 127.31 127.11 127.03 126.69 126.58 126.17 115.73 81.62 77.27 66.06 62.10 47.88 29.73 23.13 14.67
Current Data Parameters
NAME ZIE-281-02-01
EXPNO 20
PROCNO 1
F2 - Acquisition Parameters
Date_ 20151204
Time 4.20
INSTRUM spect
PROBHD 5 mm PABBO BB-
PULPROG zgpg30
TD 65536
SOLVENT CDCl3
NS 3000
DS 4
SWH 18028.846 Hz
FIDRES 0.275098 Hz
AQ 1.8175317 sec
RG 2050
DW 27.733 usec
DE 27.73 usec
TE 296.8 K
D1 2.00000000 sec
d11 0.03000000 sec
DELTA 1.89999998 sec
TD0 1
SFO1 75.4752949 MHz
NUC1 13C
P1 7.75 usec
PLW1 50.00000000 W
SFO2 300.1312005 MHz
NUC2 1H
CPDPRG[2 waltz16
PCPD2 26.00 usec
PLW2 20.00000000 W
PLW12 2.01370001 W
PLW13 0.13613001 W
F2 - Processing parameters
SI 32768
SF 75.4677490 MHz
WDW EM
SSB 0
LB 1.00 Hz
GB 0
PC 1.40
180 170 160 150 140 130 120 110 100 90 80 70 60 50 40 30 20 10 ppm

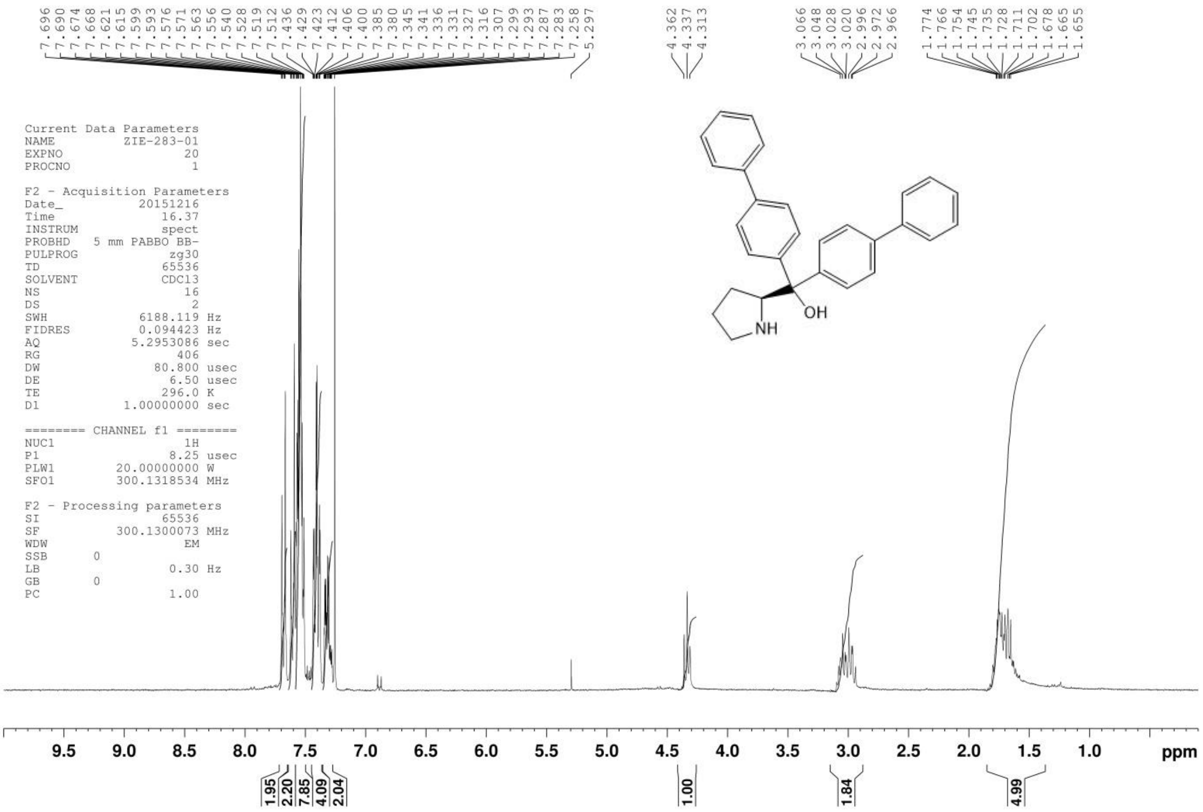
7.696 7.690 7.674 7.668 7.621 7.615 7.599 7.593 7.576 7.571 7.563 7.556 7.540 7.528 7.519 7.512 7.436 7.429 7.423 7.412 7.406 7.400 7.385 7.380 7.345 7.341 7.336 7.331 7.327 7.316 7.307 7.299 7.293 7.287 7.283 7.258 5.297
4.362 4.337 4.313
3.066 3.048 3.028 3.020 2.996 2.972 2.966
1.774 1.766 1.754 1.745 1.735 1.728 1.711 1.702 1.678 1.665 1.655
Current Data Parameters
NAME ZIE-283-01
EXPNO 20
PROCNO 1
F2 - Acquisition Parameters
Date_ 20151216
Time 16.37
INSTRUM spect
PROBHD 5 mm PABBO BB-
PULPROG zg30
TD 65536
SOLVENT CDCl3
NS 16
DS 2
SWH 6188.119 Hz
FIDRES 0.094423 Hz
AQ 5.2953086 sec
RG 406
DW 80.800 usec
DE 6.50 usec
TE 296.0 K
D1 1.00000000 sec
======== CHANNEL f1 ========
NUC1 1H
P1 8.25 usec
PLW1 20.00000000 W
SFO1 300.1318534 MHz
F2 - Processing parameters
SI 65536
SF 300.1300073 MHz
WDW EM
SSB 0
LB 0.30 Hz
GB 0
PC 1.00
OH
NH
9.5 9.0 8.5 8.0 7.5 7.0 6.5 6.0 5.5 5.0 4.5 4.0 3.5 3.0 2.5 2.0 1.5 1.0 ppm
1.95 2.20 7.85 4.09 2.04 1.00 1.84 4.99

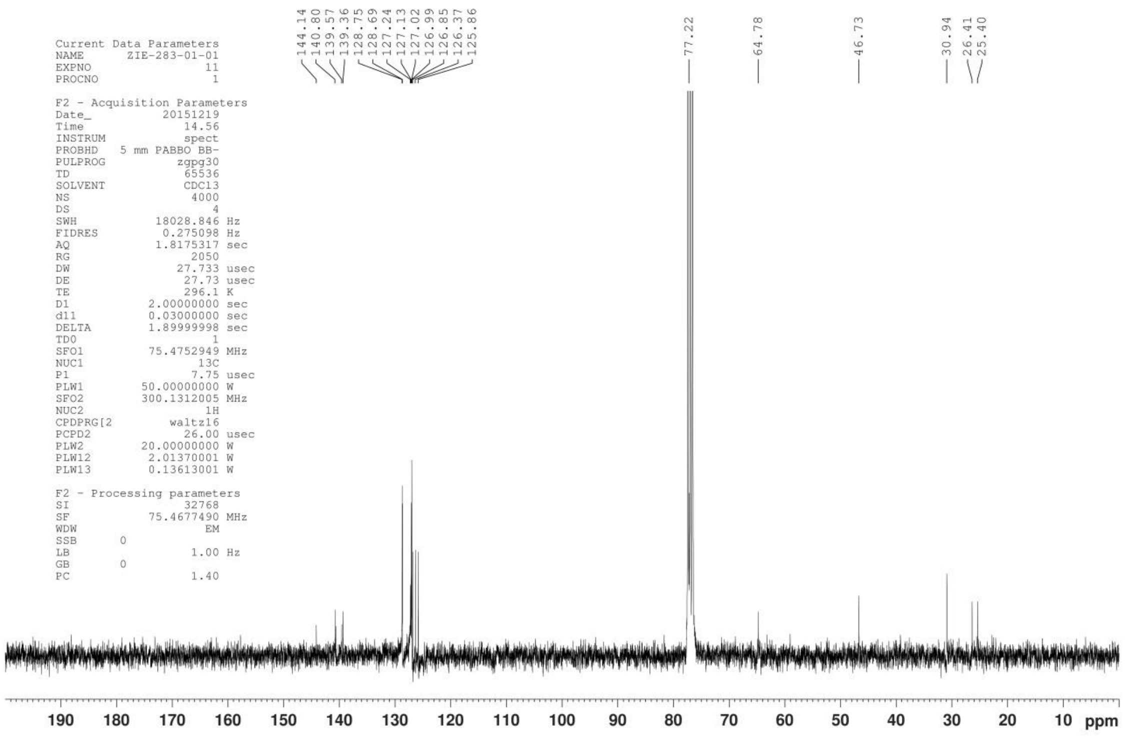
144.14 140.80 139.57 139.36 128.75 128.69 127.24 127.13 127.02 126.99 126.85 126.37 125.86
77.22
64.78
46.73
30.94 26.41 25.40
Current Data Parameters
NAME ZIE-283-01-01
EXPNO 11
PROCNO 1
F2 - Acquisition Parameters
Date_ 20151219
Time 14.56
INSTRUM spect
PROBHD 5 mm PABBO BB-
PULPROG zgpg30
TD 65536
SOLVENT CDCl3
NS 4000
DS 4
SWH 18028.846 Hz
FIDRES 0.275098 Hz
AQ 1.8175317 sec
RG 2050
DW 27.733 usec
DE 27.73 usec
TE 296.1 K
D1 2.00000000 sec
d11 0.03000000 sec
DELTA 1.89999998 sec
TD0 1
SFO1 75.4752949 MHz
NUC1 13C
P1 7.75 usec
PLW1 50.00000000 W
SFO2 300.1312005 MHz
NUC2 1H
CPDPRG[2 waltz16
PCPD2 26.00 usec
PLW2 20.00000000 W
PLW12 2.01370001 W
PLW13 0.13613001 W
F2 - Processing parameters
SI 32768
SF 75.4677490 MHz
WDW EM
SSB 0
LB 1.00 Hz
GB 0
PC 1.40
190 180 170 160 150 140 130 120 110 100 90 80 70 60 50 40 30 20 10 ppm

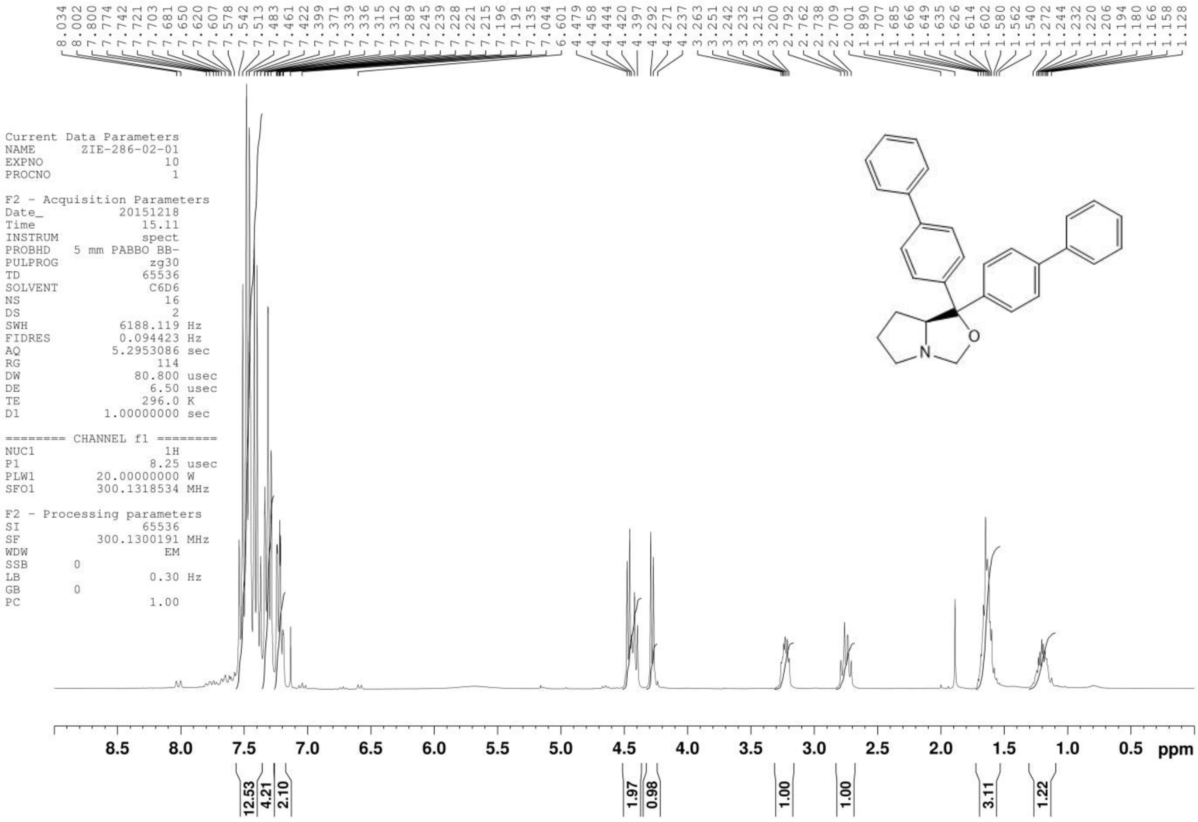
8.034 8.800 7.774 7.742 7.721 7.703 7.681 7.650 7.620 7.607 7.578 7.542 7.513 7.483 7.461 7.422 7.399 7.371 7.339 7.336 7.315 7.312 7.289 7.245 7.239 7.228 7.221 7.215 7.196 7.191 7.135 7.044 6.601 4.479 4.458 4.444 4.420 4.397 4.292 4.271 4.237 3.263 3.251 3.242 3.232 3.215 3.200 2.792 2.762 2.738 2.709 2.001 1.890 1.707 1.685 1.666 1.649 1.635 1.626 1.614 1.602 1.580 1.562 1.540 1.272 1.244 1.232 1.220 1.206 1.194 1.180 1.166 1.158 1.128
Current Data Parameters
NAME ZIE-286-02-01
EXPNO 10
PROCNO 1
F2 - Acquisition Parameters
Date_ 20151218
Time 15.11
INSTRUM spect
PROBHD 5 mm PABBO BB-
PULPROG zg30
TD 65536
SOLVENT C6D6
NS 16
DS 2
SWH 6188.119 Hz
FIDRES 0.094423 Hz
AQ 5.2953086 sec
RG 114
DW 80.800 usec
DE 6.50 usec
TE 296.0 K
D1 1.00000000 sec
======== CHANNEL f1 ========
NUC1 1H
P1 8.25 usec
PLW1 20.00000000 W
SFO1 300.1318534 MHz
F2 - Processing parameters
SI 65536
SF 300.1300191 MHz
WDW EM
SSB 0
LB 0.30 Hz
GB 0
PC 1.00
N
O
8.5 8.0 7.5 7.0 6.5 6.0 5.5 5.0 4.5 4.0 3.5 3.0 2.5 2.0 1.5 1.0 0.5 ppm
12.53 4.21 2.10 1.97 0.98 1.00 1.00 3.11 1.22

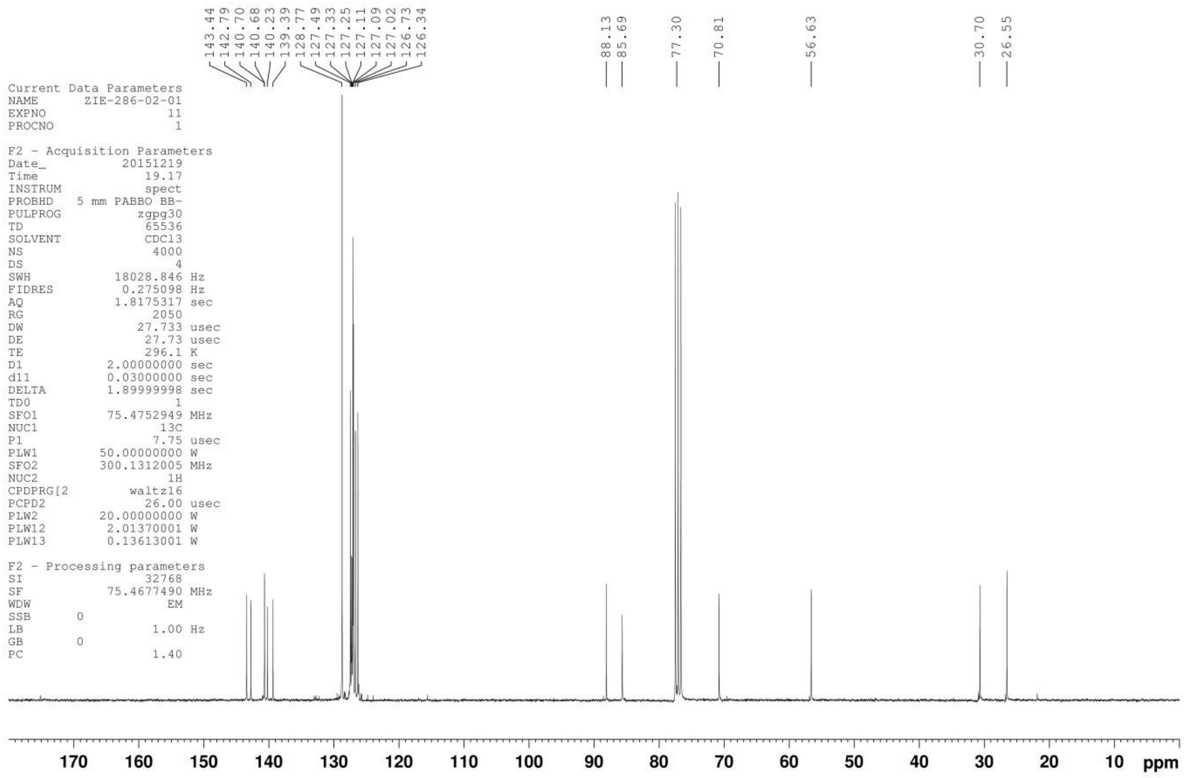
143.44 142.79 140.70 140.68 140.23 139.39 128.77 127.49 127.33 127.25 127.11 127.09 127.02 126.73 126.34 88.13 85.69 77.30 70.81 56.63 30.70 26.55
Current Data Parameters
NAME ZIE-286-02-01
EXPNO 11
PROCNO 1
F2 - Acquisition Parameters
Date_ 20151219
Time 19.17
INSTRUM spect
PROBHD 5 mm PABBO BB-
PULPROG zgpg30
TD 65536
SOLVENT CDCl3
NS 4000
DS 4
SWH 18028.846 Hz
FIDRES 0.275098 Hz
AQ 1.8175317 sec
RG 2050
DW 27.733 usec
DE 27.73 usec
TE 296.1 K
D1 2.00000000 sec
d11 0.03000000 sec
DELTA 1.89999998 sec
TD0 1
SFO1 75.4752949 MHz
NUC1 13C
P1 7.75 usec
PLW1 50.00000000 W
SFO2 300.1312005 MHz
NUC2 1H
CPDPRG[2 waltz16
PCPD2 26.00 usec
PLW2 20.00000000 W
PLW12 2.01370001 W
PLW13 0.13613001 W
F2 - Processing parameters
SI 32768
SF 75.4677490 MHz
WDW EM
SSB 0
LB 1.00 Hz
GB 0
PC 1.40
170 160 150 140 130 120 110 100 90 80 70 60 50 40 30 20 10 ppm

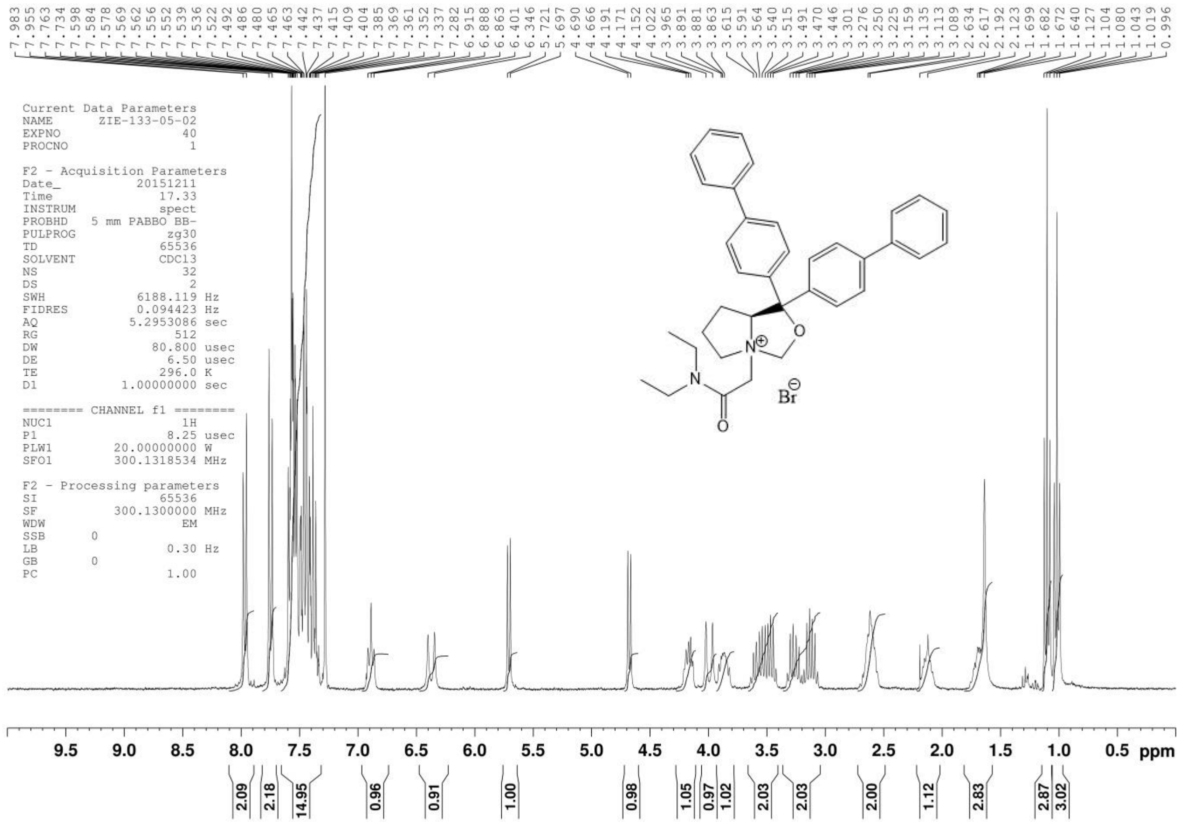
Current Data Parameters
NAME ZIE-133-05-02
EXPNO 40
PROCNO 1
F2 - Acquisition Parameters
Date_ 20151211
Time 17.33
INSTRUM spect
PROBHD 5 mm PABBO BB-
PULPROG zg30
TD 65536
SOLVENT CDCl3
NS 32
DS 2
SWH 6188.119 Hz
FIDRES 0.094423 Hz
AQ 5.2953086 sec
RG 512
DW 80.800 usec
DE 6.50 usec
TE 296.0 K
D1 1.00000000 sec
======== CHANNEL f1 ========
NUC1 1H
P1 8.25 usec
PLW1 20.00000000 W
SFO1 300.1318534 MHz
F2 - Processing parameters
SI 65536
SF 300.1300000 MHz
WDW EM
SSB 0
LB 0.30 Hz
GB 0
PC 1.00
Br⊖
9.5 9.0 8.5 8.0 7.5 7.0 6.5 6.0 5.5 5.0 4.5 4.0 3.5 3.0 2.5 2.0 1.5 1.0 0.5 ppm
2.09 2.18 14.95 0.96 0.91 1.00 0.98 1.05 0.97 1.02 2.03 2.03 2.00 1.12 2.83 2.87 3.02

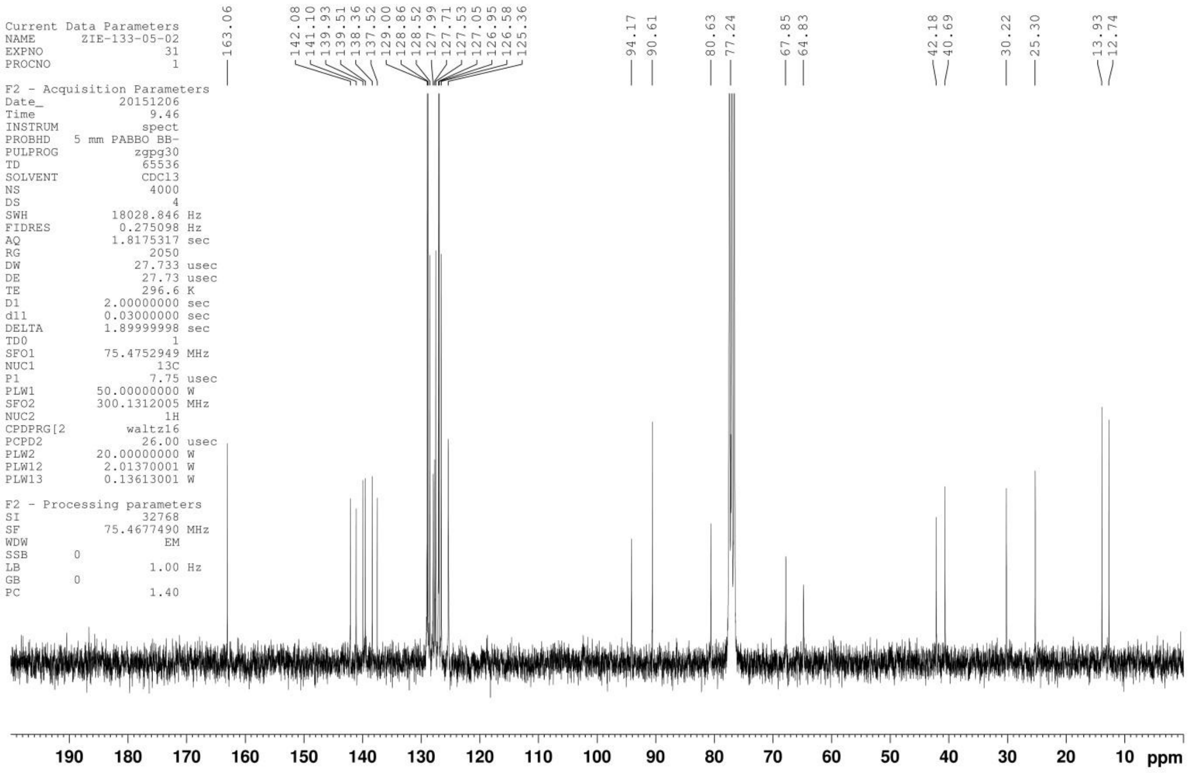
Current Data Parameters
NAME ZIE-133-05-02
EXPNO 31
PROCNO 1
F2 - Acquisition Parameters
Date_ 20151206
Time 9.46
INSTRUM spect
PROBHD 5 mm PABBO BB-
PULPROG zgpg30
TD 65536
SOLVENT CDCl3
NS 4000
DS 4
SWH 18028.846 Hz
FIDRES 0.275098 Hz
AQ 1.8175317 sec
RG 2050
DW 27.733 usec
DE 27.73 usec
TE 296.6 K
D1 2.00000000 sec
d11 0.03000000 sec
DELTA 1.89999998 sec
TD0 1
SFO1 75.4752949 MHz
NUC1 13C
P1 7.75 usec
PLW1 50.00000000 W
SFO2 300.1312005 MHz
NUC2 1H
CPDPRG[2 waltz16
PCPD2 26.00 usec
PLW2 20.00000000 W
PLW12 2.01370001 W
PLW13 0.13613001 W
F2 - Processing parameters
SI 32768
SF 75.4677490 MHz
WDW EM
SSB 0
LB 1.00 Hz
GB 0
PC 1.40
163.06
142.08 141.10 139.93 139.51 138.36 137.52 129.00 128.86 128.52 127.99 127.71 127.53 127.05 126.95 126.58 125.36
94.17 90.61
80.63 77.24
67.85 64.83
42.18 40.69
30.22 25.30
13.93 12.74
190 180 170 160 150 140 130 120 110 100 90 80 70 60 50 40 30 20 10 ppm

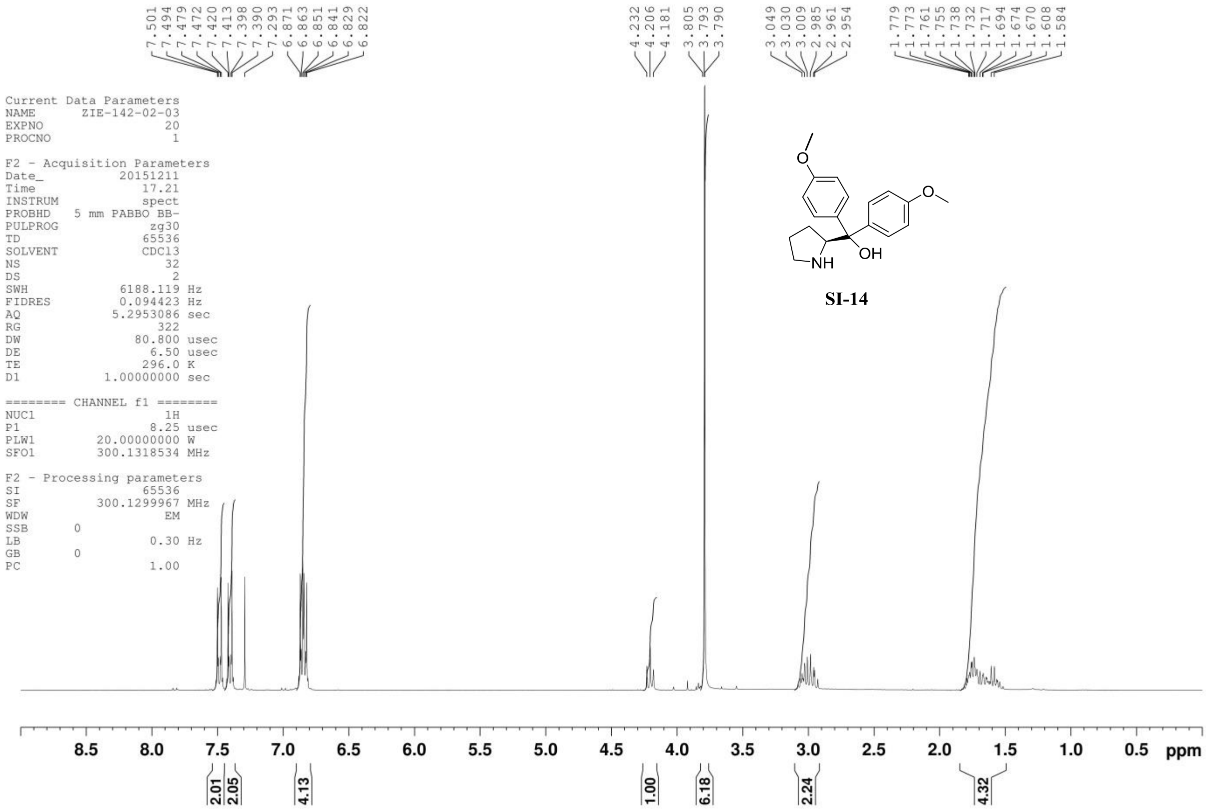
7.501
7.494
7.479
7.472
7.420
7.413
7.398
7.390
7.293
6.871
6.863
6.851
6.841
6.829
6.822
4.232
4.206
4.181
3.805
3.793
3.790
3.049
3.030
3.009
2.985
2.961
2.954
1.779
1.773
1.761
1.755
1.738
1.732
1.717
1.694
1.674
1.670
1.608
1.584
Current Data Parameters
NAME ZIE-142-02-03
EXPNO 20
PROCNO 1
F2 - Acquisition Parameters
Date_ 20151211
Time 17.21
INSTRUM spect
PROBHD 5 mm PABBO BB-
PULPROG zg30
TD 65536
SOLVENT CDCl3
NS 32
DS 2
SWH 6188.119 Hz
FIDRES 0.094423 Hz
AQ 5.2953086 sec
RG 322
DW 80.800 usec
DE 6.50 usec
TE 296.0 K
D1 1.00000000 sec
======== CHANNEL f1 ========
NUC1 1H
P1 8.25 usec
PLW1 20.00000000 W
SFO1 300.1318534 MHz
F2 - Processing parameters
SI 65536
SF 300.1299967 MHz
WDW EM
SSB 0
LB 0.30 Hz
GB 0
PC 1.00
8.5 8.0 7.5 7.0 6.5 6.0 5.5 5.0 4.5 4.0 3.5 3.0 2.5 2.0 1.5 1.0 0.5 ppm
2.01
2.05
4.13
1.00
6.18
2.24
4.32
O
O
NH
OH

SI-14

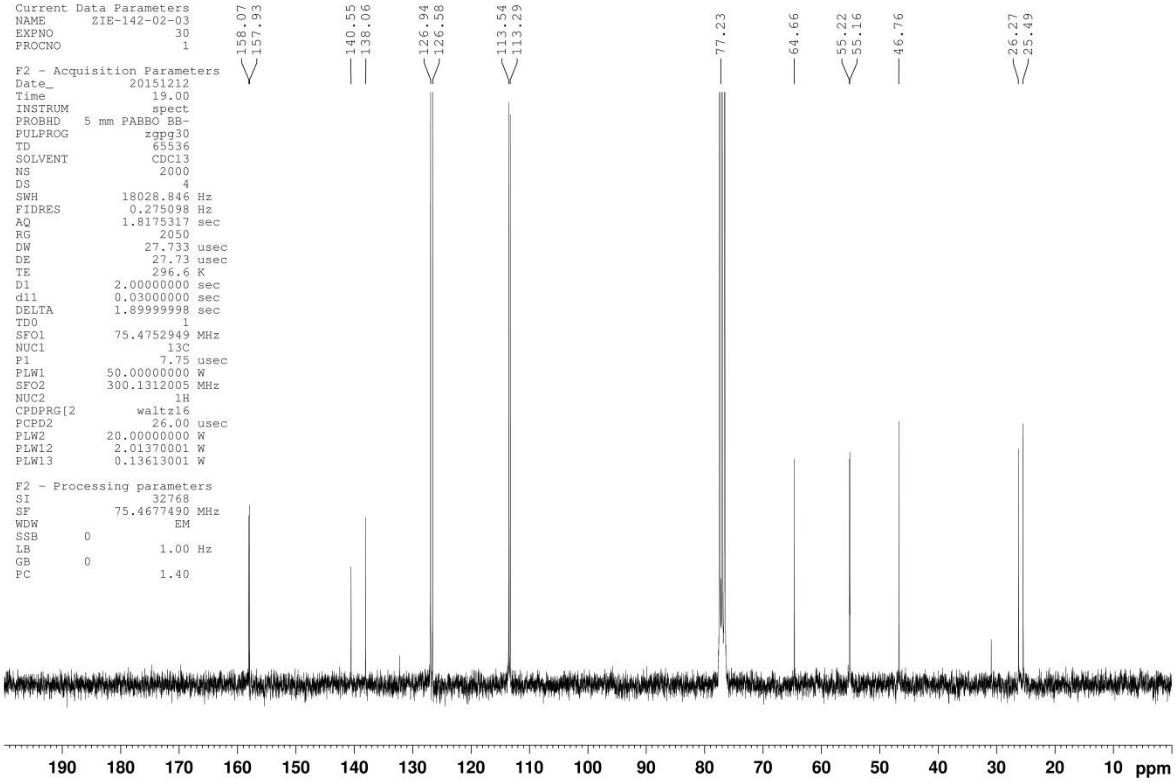
Current Data Parameters
NAME ZIE-142-02-03
EXPNO 30
PROCNO 1
F2 - Acquisition Parameters
Date_ 20151212
Time 19.00
INSTRUM spect
PROBHD 5 mm PABBO BB-
PULPROG zgpg30
TD 65536
SOLVENT CDCl3
NS 2000
DS 4
SWH 18028.846 Hz
FIDRES 0.275098 Hz
AQ 1.8175317 sec
RG 2050
DW 27.733 usec
DE 27.73 usec
TE 296.6 K
D1 2.00000000 sec
d11 0.03000000 sec
DELTA 1.89999998 sec
TD0 1
SFO1 75.4752949 MHz
NUC1 13C
P1 7.75 usec
PLW1 50.00000000 W
SFO2 300.1312005 MHz
NUC2 1H
CPDPRG[2 waltz16
PCPD2 26.00 usec
PLW2 20.00000000 W
PLW12 2.01370001 W
PLW13 0.13613001 W
F2 - Processing parameters
SI 32768
SF 75.4677490 MHz
WDW EM
SSB 0
LB 1.00 Hz
GB 0
PC 1.40
158.07
157.93
140.55
138.06
126.94
126.58
113.54
113.29
77.23
64.66
55.22
55.16
46.76
26.27
25.49
190 180 170 160 150 140 130 120 110 100 90 80 70 60 50 40 30 20 10 ppm

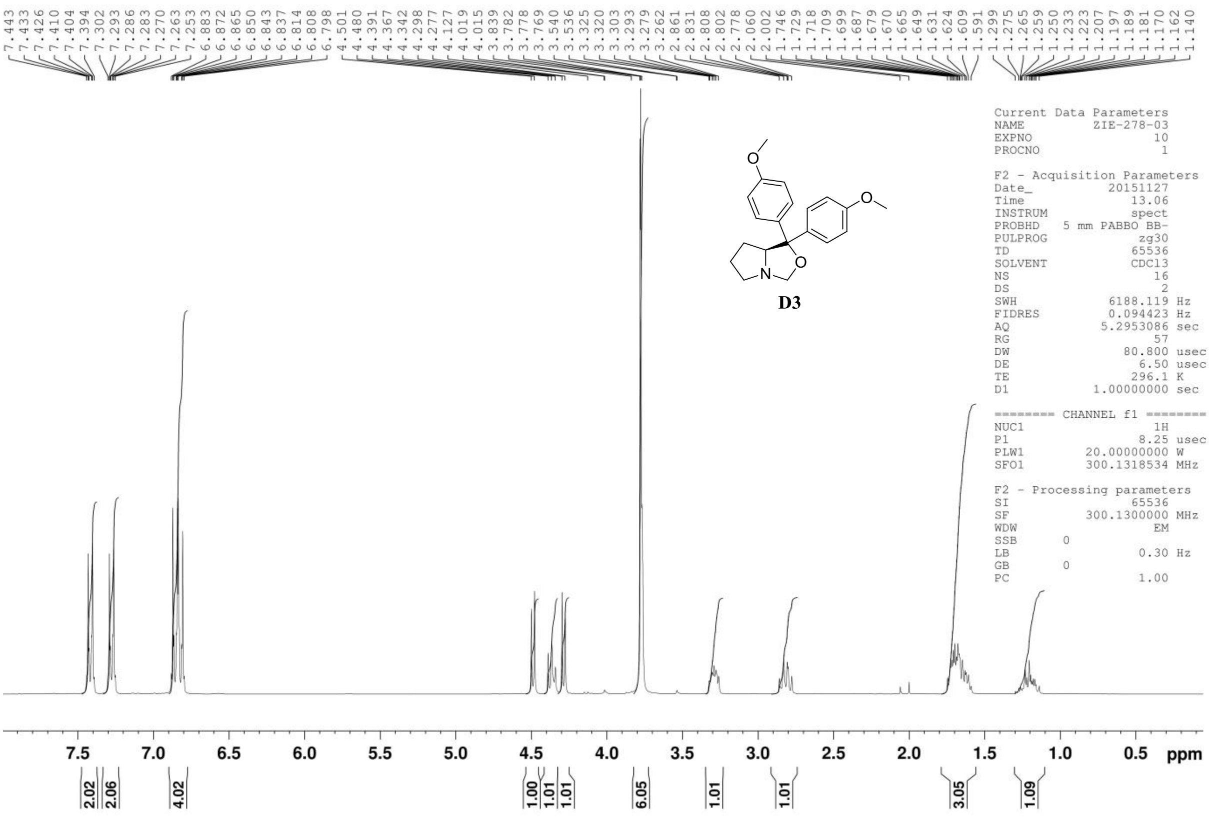
7.443 7.433 7.426 7.410 7.404 7.394 7.302 7.293 7.286 7.283 7.270 7.263 7.253 6.883 6.872 6.865 6.850 6.843 6.837 6.814 6.808 6.798 4.501 4.480 4.391 4.367 4.342 4.298 4.277 4.127 4.019 4.015 3.839 3.782 3.778 3.769 3.540 3.536 3.325 3.320 3.303 3.293 3.279 3.262 2.861 2.831 2.808 2.802 2.778 2.060 2.002 1.746 1.729 1.718 1.709 1.699 1.687 1.679 1.670 1.665 1.649 1.631 1.624 1.609 1.591 1.299 1.275 1.265 1.259 1.250 1.233 1.223 1.207 1.197 1.189 1.181 1.170 1.162 1.140
D3
Current Data Parameters
NAME ZIE-278-03
EXPNO 10
PROCNO 1
F2 - Acquisition Parameters
Date_ 20151127
Time 13.06
INSTRUM spect
PROBHD 5 mm PABBO BB-
PULPROG zg30
TD 65536
SOLVENT CDCl3
NS 16
DS 2
SWH 6188.119 Hz
FIDRES 0.094423 Hz
AQ 5.2953086 sec
RG 57
DW 80.800 usec
DE 6.50 usec
TE 296.1 K
D1 1.00000000 sec
======== CHANNEL f1 ========
NUC1 1H
P1 8.25 usec
PLW1 20.00000000 W
SFO1 300.1318534 MHz
F2 - Processing parameters
SI 65536
SF 300.1300000 MHz
WDW EM
SSB 0
LB 0.30 Hz
GB 0
PC 1.00
7.5 7.0 6.5 6.0 5.5 5.0 4.5 4.0 3.5 3.0 2.5 2.0 1.5 1.0 0.5 ppm
2.02 2.06 4.02 1.00 1.01 1.01 6.05 1.01 1.01 3.05 1.09

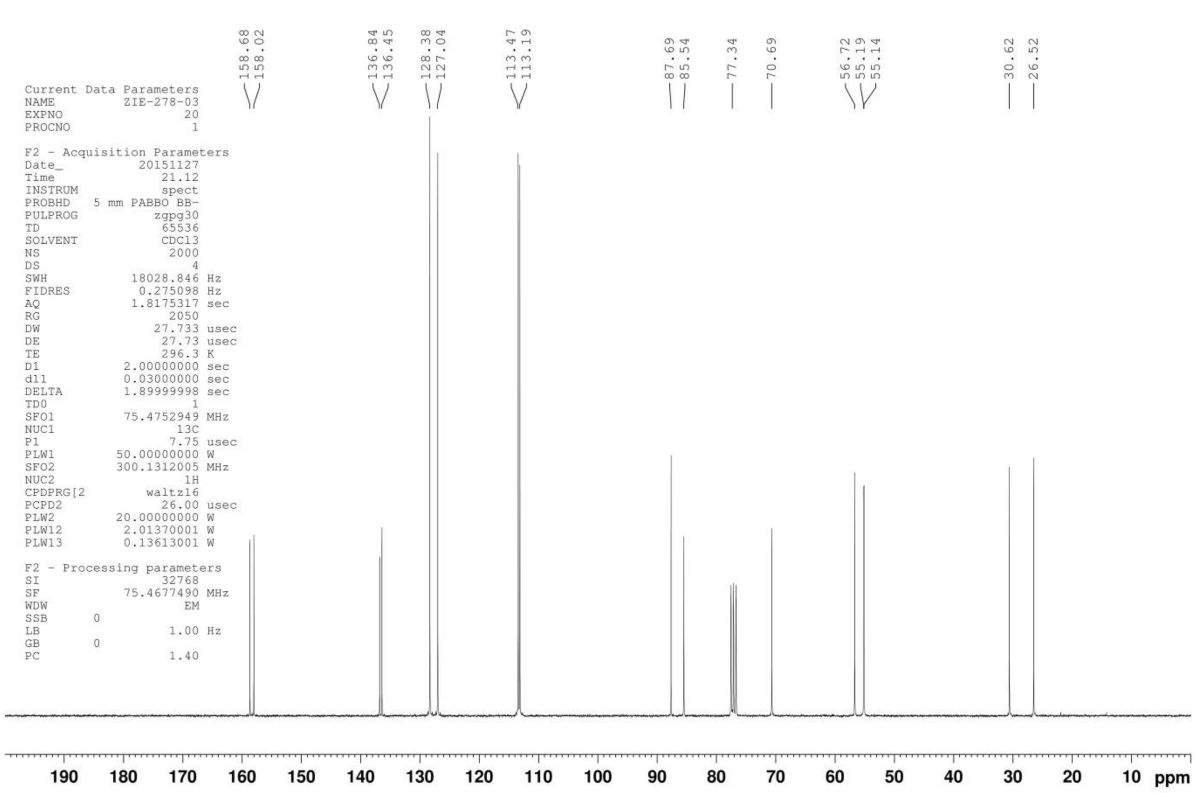
158.68 158.02 136.84 136.45 128.38 127.04 113.47 113.19 87.69 85.54 77.34 70.69 56.72 55.19 55.14 30.62 26.52
Current Data Parameters
NAME ZIE-278-03
EXPNO 20
PROCNO 1
F2 - Acquisition Parameters
Date_ 20151127
Time 21.12
INSTRUM spect
PROBHD 5 mm PABBO BB-
PULPROG zgpg30
TD 65536
SOLVENT CDCl3
NS 2000
DS 4
SWH 18028.846 Hz
FIDRES 0.275098 Hz
AQ 1.8175317 sec
RG 2050
DW 27.733 usec
DE 27.73 usec
TE 296.3 K
D1 2.00000000 sec
d11 0.03000000 sec
DELTA 1.89999998 sec
TD0 1
SFO1 75.4752949 MHz
NUC1 13C
P1 7.75 usec
PLW1 50.00000000 W
SFO2 300.1312005 MHz
NUC2 1H
CPDPRG[2 waltz16
PCPD2 26.00 usec
PLW2 20.00000000 W
PLW12 2.01370001 W
PLW13 0.13613001 W
F2 - Processing parameters
SI 32768
SF 75.4677490 MHz
WDW EM
SSB 0
LB 1.00 Hz
GB 0
PC 1.40
190 180 170 160 150 140 130 120 110 100 90 80 70 60 50 40 30 20 10 ppm

```
Current Data Parameters
NAME        ZIE-151-01-02
EXPNO                  20
PROCNO                  1

F2 - Acquisition Parameters
Date_           20151211
Time               17.39
INSTRUM            spect
PROBHD   5 mm PABBO BB-
PULPROG             zg30
TD                 65536
SOLVENT            CDCl3
NS                    32
DS                     2
SWH          6188.119 Hz
FIDRES       0.094423 Hz
AQ         5.2953086 sec
RG                   256
DW            80.800 usec
DE              6.50 usec
TE               295.9 K
D1        1.00000000 sec

======== CHANNEL f1 ========
NUC1                  1H
P1              8.25 usec
PLW1       20.00000000 W
SFO1      300.1318534 MHz

F2 - Processing parameters
SI                 65536
SF        300.1300000 MHz
WDW                   EM
SSB      0
LB               0.30 Hz
GB       0
PC               1.00
```

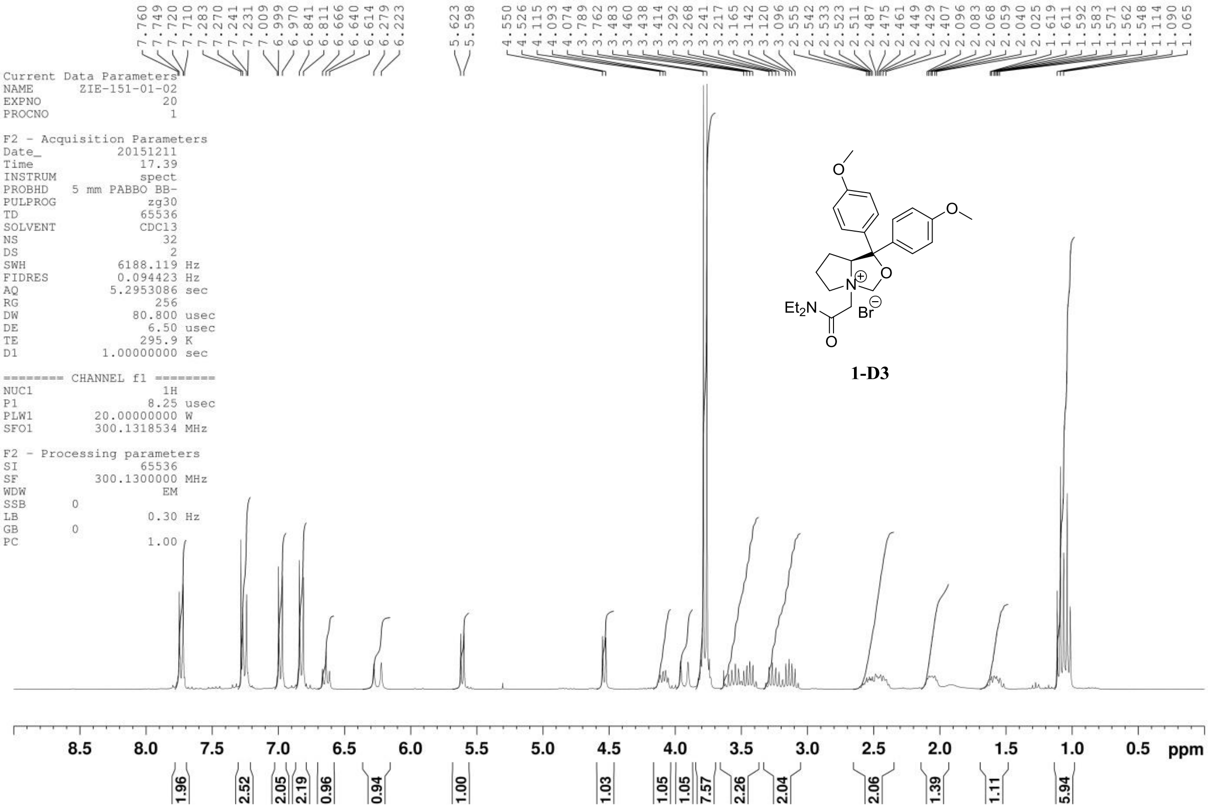

1-D3

```
Current Data Parameters
NAME        ZIE-151-01-02
EXPNO                  11
PROCNO                  1

F2 - Acquisition Parameters
Date_           20151120
Time                5.25
INSTRUM            spect
PROBHD   5 mm PABBO BB-
PULPROG           zgpg30
TD                 65536
SOLVENT            CDCl3
NS                  2000
DS                     4
SWH         18028.846 Hz
FIDRES       0.275098 Hz
AQ         1.8175317 sec
RG                  2050
DW            27.733 usec
DE             27.73 usec
TE               296.7 K
D1        2.00000000 sec
d11       0.03000000 sec
DELTA     1.89999998 sec
TD0                    1
SFO1      75.4752949 MHz
NUC1                 13C
P1              7.75 usec
PLW1       50.00000000 W
SFO2      300.1312005 MHz
NUC2                  1H
CPDPRG[2         waltz16
PCPD2          26.00 usec
PLW2       20.00000000 W
PLW12       2.01370001 W
PLW13       0.13613001 W

F2 - Processing parameters
SI                 32768
SF         75.4677490 MHz
WDW                   EM
SSB      0
LB               1.00 Hz
GB       0
PC               1.40
```

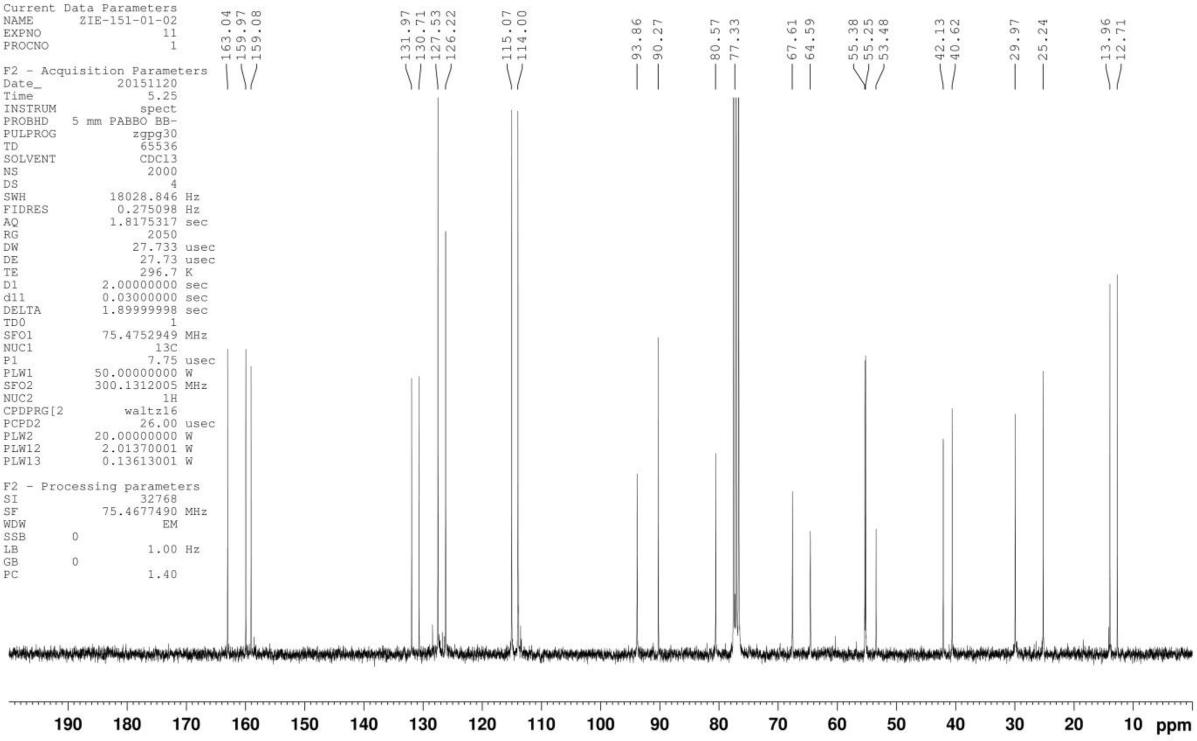

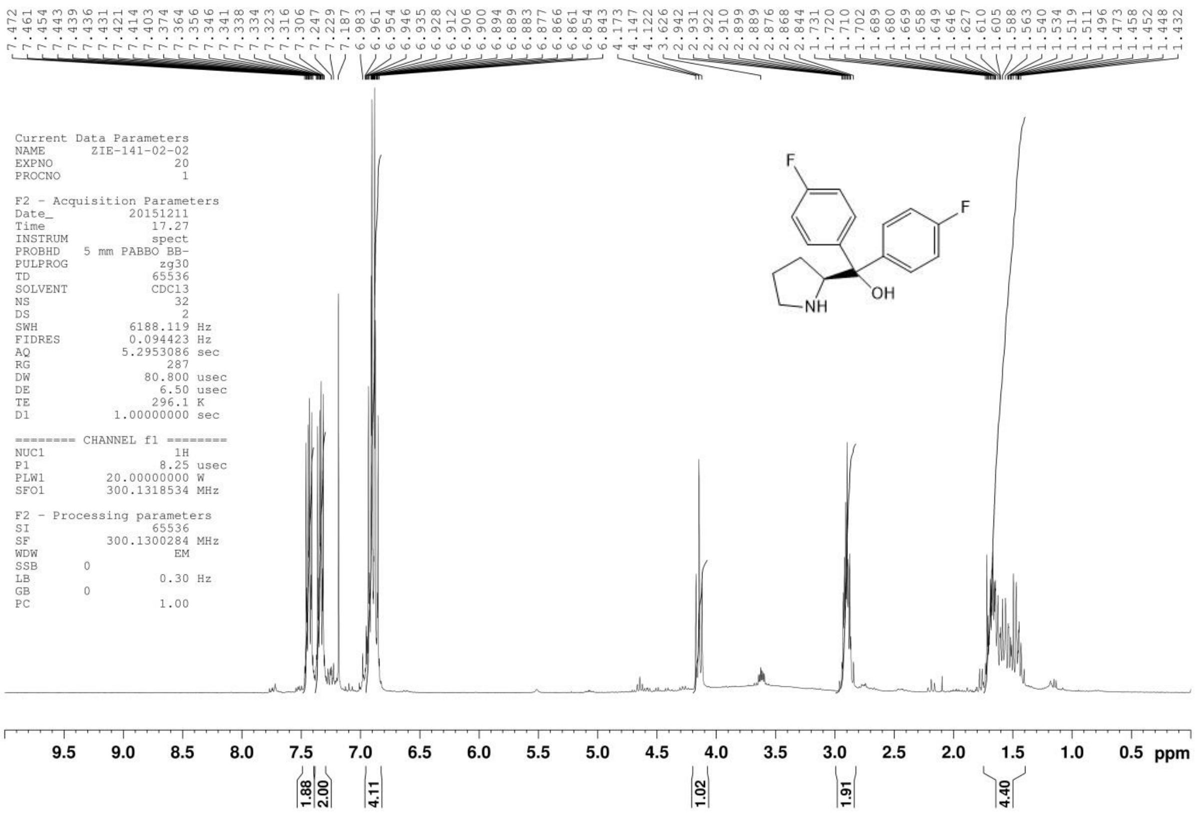
7.472 7.461 7.454 7.443 7.439 7.436 7.431 7.421 7.414 7.403 7.374 7.364 7.356 7.346 7.341 7.338 7.334 7.323 7.316 7.306 7.247 7.229 7.187 6.983 6.961 6.954 6.946 6.935 6.928 6.912 6.906 6.900 6.894 6.889 6.883 6.877 6.866 6.861 6.854 6.843 4.173 4.147 4.122 3.626 2.942 2.931 2.922 2.910 2.899 2.889 2.876 2.868 2.844 1.731 1.720 1.710 1.702 1.689 1.680 1.669 1.658 1.649 1.646 1.627 1.610 1.605 1.588 1.563 1.540 1.534 1.519 1.511 1.496 1.473 1.458 1.452 1.448 1.432
Current Data Parameters
NAME ZIE-141-02-02
EXPNO 20
PROCNO 1
F2 - Acquisition Parameters
Date_ 20151211
Time 17.27
INSTRUM spect
PROBHD 5 mm PABBO BB-
PULPROG zg30
TD 65536
SOLVENT CDCl3
NS 32
DS 2
SWH 6188.119 Hz
FIDRES 0.094423 Hz
AQ 5.2953086 sec
RG 287
DW 80.800 usec
DE 6.50 usec
TE 296.1 K
D1 1.00000000 sec
======== CHANNEL f1 ========
NUC1 1H
P1 8.25 usec
PLW1 20.00000000 W
SFO1 300.1318534 MHz
F2 - Processing parameters
SI 65536
SF 300.1300284 MHz
WDW EM
SSB 0
LB 0.30 Hz
GB 0
PC 1.00
F
F
OH
NH
9.5 9.0 8.5 8.0 7.5 7.0 6.5 6.0 5.5 5.0 4.5 4.0 3.5 3.0 2.5 2.0 1.5 1.0 0.5 ppm
1.88 2.00 4.11 1.02 1.91 4.40

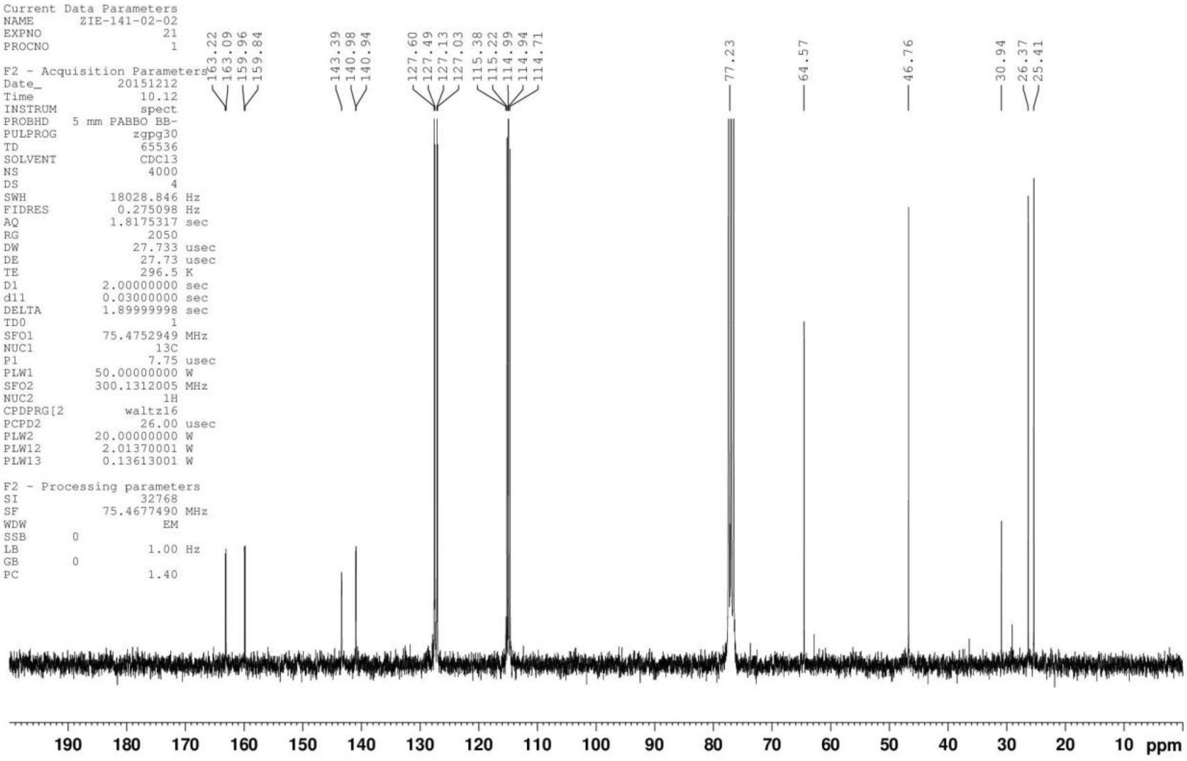
Current Data Parameters
NAME ZIE-141-02-02
EXPNO 21
PROCNO 1
F2 - Acquisition Parameters
Date_ 20151212
Time 10.12
INSTRUM spect
PROBHD 5 mm PABBO BB-
PULPROG zgpg30
TD 65536
SOLVENT CDCl3
NS 4000
DS 4
SWH 18028.846 Hz
FIDRES 0.275098 Hz
AQ 1.8175317 sec
RG 2050
DW 27.733 usec
DE 27.73 usec
TE 296.5 K
D1 2.00000000 sec
d11 0.03000000 sec
DELTA 1.89999998 sec
TD0 1
SFO1 75.4752949 MHz
NUC1 13C
P1 7.75 usec
PLW1 50.00000000 W
SFO2 300.1312005 MHz
NUC2 1H
CPDPRG[2 waltz16
PCPD2 26.00 usec
PLW2 20.00000000 W
PLW12 2.01370001 W
PLW13 0.13613001 W
F2 - Processing parameters
SI 32768
SF 75.4677490 MHz
WDW EM
SSB 0
LB 1.00 Hz
GB 0
PC 1.40
163.22 163.09 159.96 159.84 143.39 140.98 140.94 127.60 127.49 127.13 127.03 115.38 115.22 114.99 114.94 114.71 77.23 64.57 46.76 30.94 26.37 25.41
190 180 170 160 150 140 130 120 110 100 90 80 70 60 50 40 30 20 10 ppm

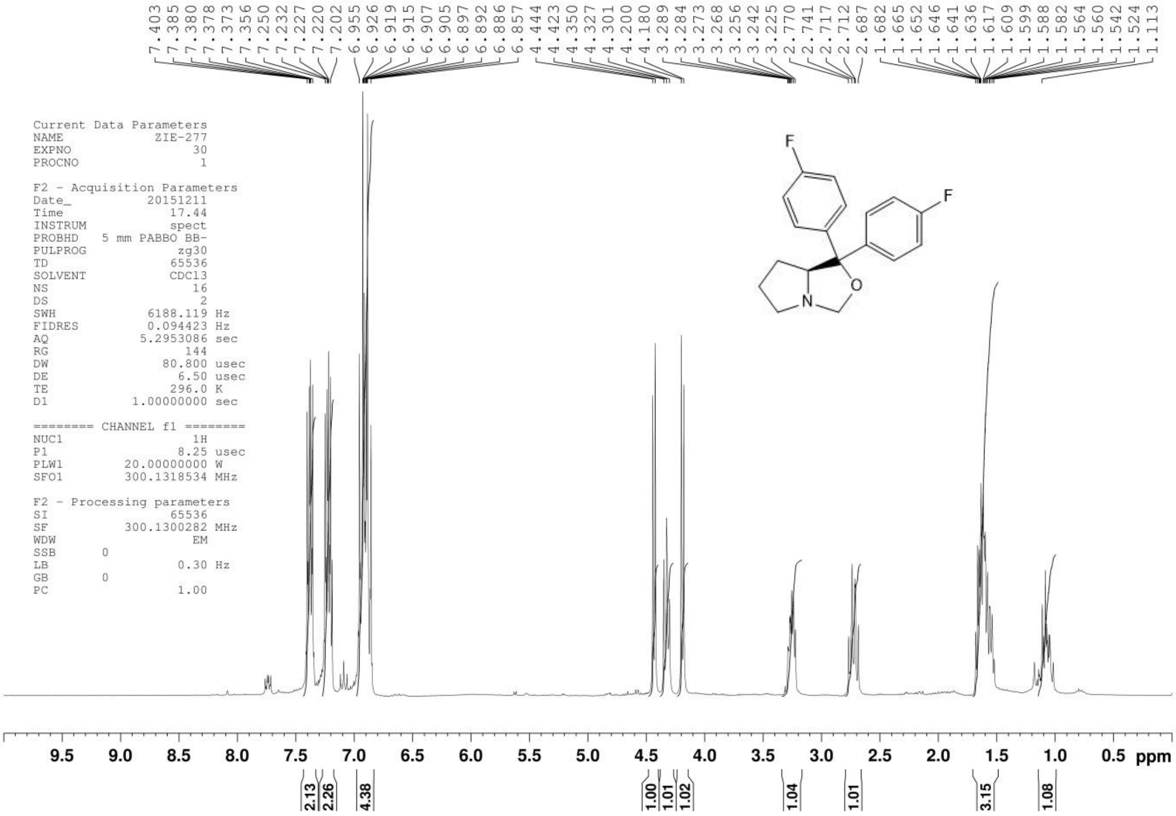
7.403 7.385 7.380 7.378 7.373 7.356 7.250 7.232 7.227 7.220 7.202 6.955 6.926 6.919 6.915 6.907 6.905 6.897 6.892 6.886 6.857 4.444 4.423 4.350 4.327 4.301 4.200 4.180 3.289 3.284 3.273 3.268 3.256 3.242 3.225 2.770 2.741 2.717 2.712 2.687 1.682 1.665 1.652 1.646 1.641 1.636 1.617 1.609 1.599 1.588 1.582 1.564 1.560 1.542 1.524 1.113
Current Data Parameters
NAME ZIE-277
EXPNO 30
PROCNO 1
F2 - Acquisition Parameters
Date_ 20151211
Time 17.44
INSTRUM spect
PROBHD 5 mm PABBO BB-
PULPROG zg30
TD 65536
SOLVENT CDCl3
NS 16
DS 2
SWH 6188.119 Hz
FIDRES 0.094423 Hz
AQ 5.2953086 sec
RG 144
DW 80.800 usec
DE 6.50 usec
TE 296.0 K
D1 1.00000000 sec
======== CHANNEL f1 ========
NUC1 1H
P1 8.25 usec
PLW1 20.00000000 W
SFO1 300.1318534 MHz
F2 - Processing parameters
SI 65536
SF 300.1300282 MHz
WDW EM
SSB 0
LB 0.30 Hz
GB 0
PC 1.00
F
F
N
O
9.5 9.0 8.5 8.0 7.5 7.0 6.5 6.0 5.5 5.0 4.5 4.0 3.5 3.0 2.5 2.0 1.5 1.0 0.5 ppm
2.13 2.26 4.38 1.00 1.01 1.02 1.04 1.01 3.15 1.08

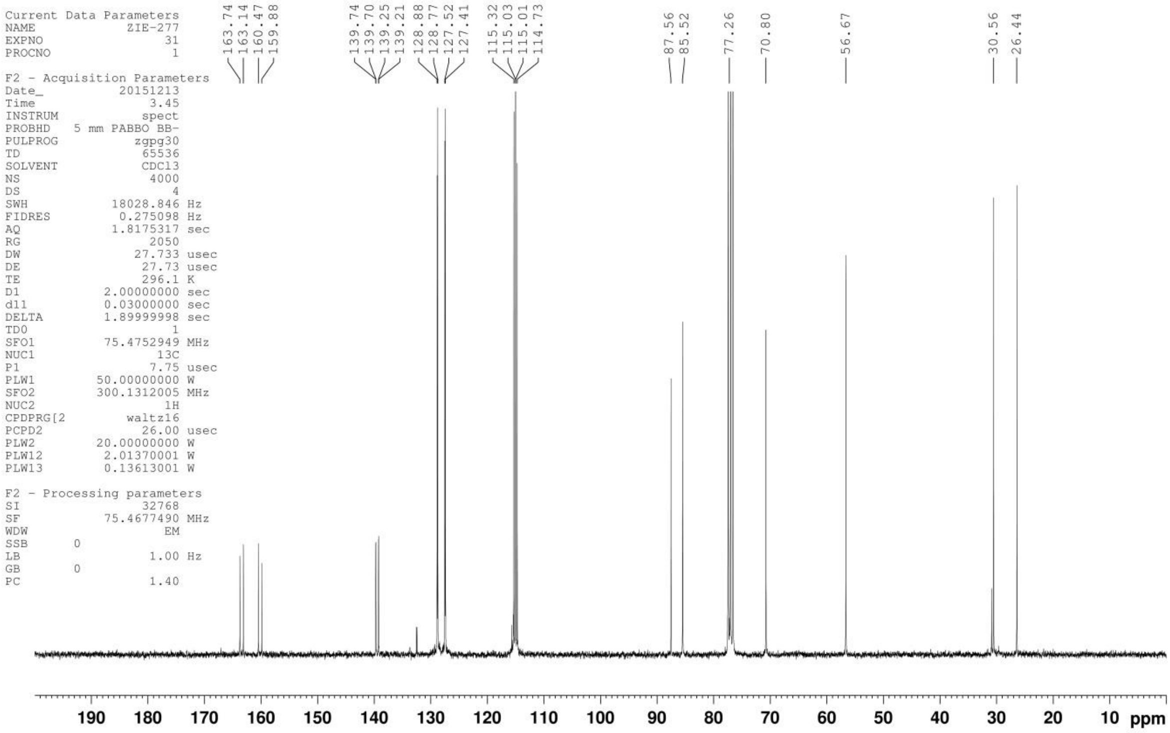
Current Data Parameters
NAME ZIE-277
EXPNO 31
PROCNO 1
F2 - Acquisition Parameters
Date_ 20151213
Time 3.45
INSTRUM spect
PROBHD 5 mm PABBO BB-
PULPROG zgpg30
TD 65536
SOLVENT CDCl3
NS 4000
DS 4
SWH 18028.846 Hz
FIDRES 0.275098 Hz
AQ 1.8175317 sec
RG 2050
DW 27.733 usec
DE 27.73 usec
TE 296.1 K
D1 2.00000000 sec
d11 0.03000000 sec
DELTA 1.89999998 sec
TD0 1
SFO1 75.4752949 MHz
NUC1 13C
P1 7.75 usec
PLW1 50.00000000 W
SFO2 300.1312005 MHz
NUC2 1H
CPDPRG[2 waltz16
PCPD2 26.00 usec
PLW2 20.00000000 W
PLW12 2.01370001 W
PLW13 0.13613001 W
F2 - Processing parameters
SI 32768
SF 75.4677490 MHz
WDW EM
SSB 0
LB 1.00 Hz
GB 0
PC 1.40
163.74 163.14 160.47 159.88 139.74 139.70 139.25 139.21 128.88 128.77 127.52 127.41 115.32 115.03 115.01 114.73 87.56 85.52 77.26 70.80 56.67 30.56 26.44
190 180 170 160 150 140 130 120 110 100 90 80 70 60 50 40 30 20 10 ppm

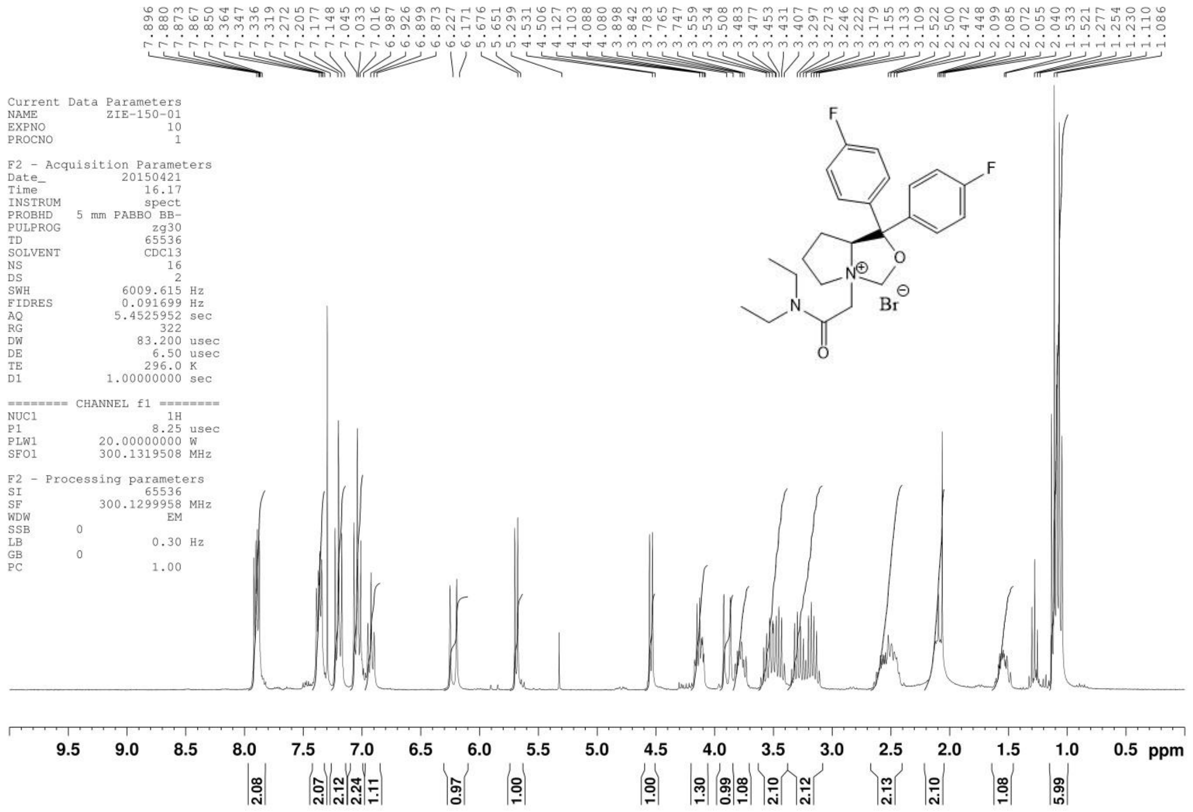

7.896 7.880 7.873 7.867 7.850 7.364 7.347 7.336 7.319 7.272 7.205 7.177 7.148 7.045 7.033 7.016 6.987 6.926 6.899 6.873 6.227 6.171 5.676 5.651 5.299 4.531 4.506 4.127 4.103 4.088 4.080 3.898 3.842 3.783 3.765 3.747 3.559 3.534 3.508 3.483 3.477 3.453 3.431 3.407 3.297 3.273 3.246 3.222 3.179 3.155 3.133 3.109 2.522 2.500 2.472 2.448 2.099 2.085 2.072 2.055 2.040 1.533 1.521 1.277 1.254 1.230 1.110 1.086
Current Data Parameters
NAME ZIE-150-01
EXPNO 10
PROCNO 1
F2 - Acquisition Parameters
Date_ 20150421
Time 16.17
INSTRUM spect
PROBHD 5 mm PABBO BB-
PULPROG zg30
TD 65536
SOLVENT CDCl3
NS 16
DS 2
SWH 6009.615 Hz
FIDRES 0.091699 Hz
AQ 5.4525952 sec
RG 322
DW 83.200 usec
DE 6.50 usec
TE 296.0 K
D1 1.00000000 sec
======== CHANNEL f1 ========
NUC1 1H
P1 8.25 usec
PLW1 20.00000000 W
SFO1 300.1319508 MHz
F2 - Processing parameters
SI 65536
SF 300.1299958 MHz
WDW EM
SSB 0
LB 0.30 Hz
GB 0
PC 1.00
F
F
O
N⊕
N
O
Br⊖
9.5 9.0 8.5 8.0 7.5 7.0 6.5 6.0 5.5 5.0 4.5 4.0 3.5 3.0 2.5 2.0 1.5 1.0 0.5 ppm
2.08 2.07 2.12 2.24 1.11 0.97 1.00 1.00 1.30 0.99 1.08 2.10 2.12 2.13 2.10 1.08 5.99

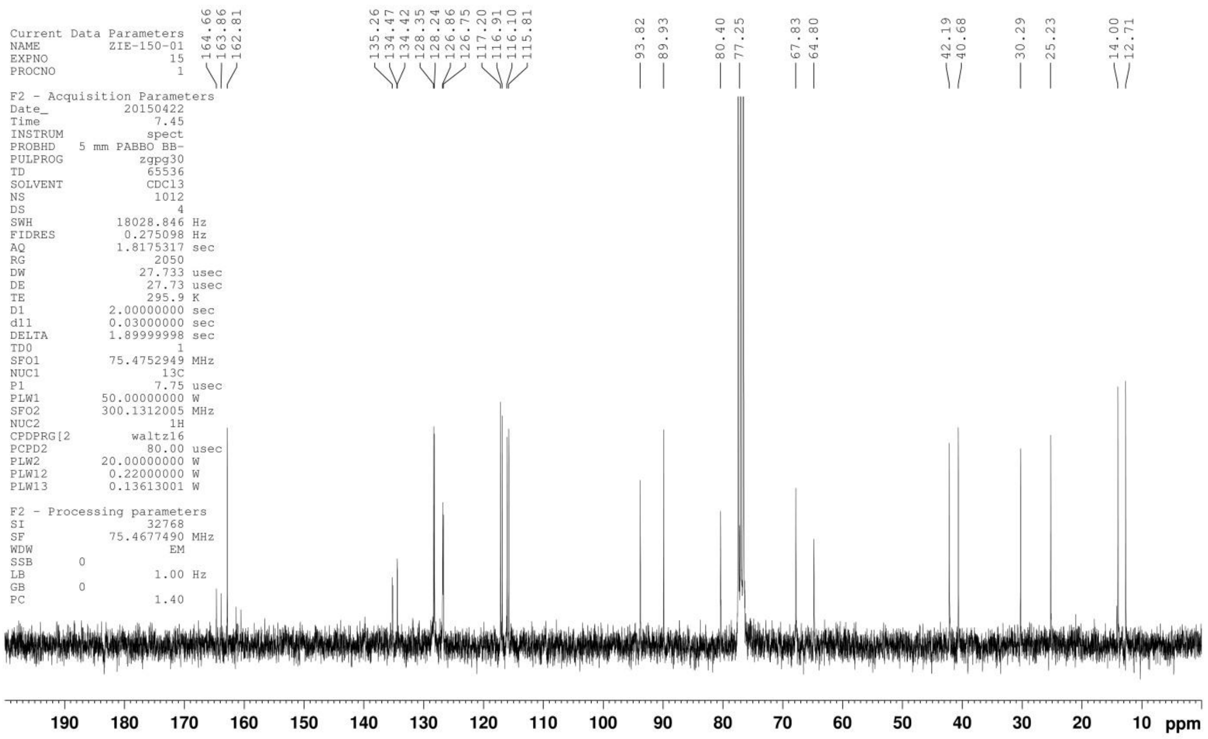

164.66 163.86 162.81 135.26 134.47 134.42 128.35 128.24 126.86 126.75 117.20 116.91 116.10 115.81 93.82 89.93 80.40 77.25 67.83 64.80 42.19 40.68 30.29 25.23 14.00 12.71
Current Data Parameters
NAME ZIE-150-01
EXPNO 15
PROCNO 1
F2 - Acquisition Parameters
Date_ 20150422
Time 7.45
INSTRUM spect
PROBHD 5 mm PABBO BB-
PULPROG zgpg30
TD 65536
SOLVENT CDCl3
NS 1012
DS 4
SWH 18028.846 Hz
FIDRES 0.275098 Hz
AQ 1.8175317 sec
RG 2050
DW 27.733 usec
DE 27.73 usec
TE 295.9 K
D1 2.00000000 sec
d11 0.03000000 sec
DELTA 1.89999998 sec
TD0 1
SFO1 75.4752949 MHz
NUC1 13C
P1 7.75 usec
PLW1 50.00000000 W
SFO2 300.1312005 MHz
NUC2 1H
CPDPRG[2 waltz16
PCPD2 80.00 usec
PLW2 20.00000000 W
PLW12 0.22000000 W
PLW13 0.13613001 W
F2 - Processing parameters
SI 32768
SF 75.4677490 MHz
WDW EM
SSB 0
LB 1.00 Hz
GB 0
PC 1.40
190 180 170 160 150 140 130 120 110 100 90 80 70 60 50 40 30 20 10 ppm

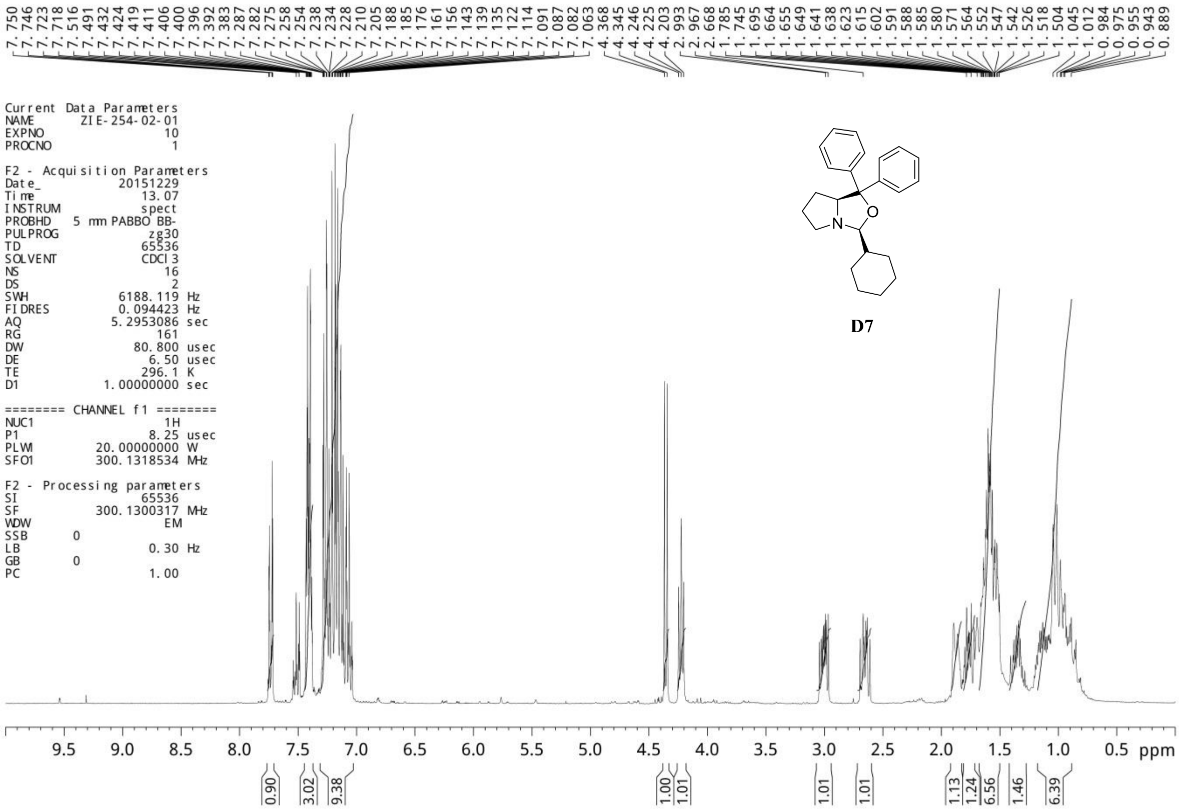
7.750 7.746 7.723 7.718 7.516 7.491 7.432 7.424 7.419 7.411 7.406 7.400 7.396 7.392 7.383 7.287 7.282 7.275 7.258 7.254 7.238 7.234 7.228 7.210 7.205 7.188 7.185 7.176 7.161 7.156 7.143 7.139 7.135 7.122 7.114 7.091 7.087 7.082 7.063 4.368 4.345 4.246 4.225 4.203 2.993 2.967 2.668 1.785 1.745 1.695 1.664 1.655 1.649 1.641 1.638 1.623 1.615 1.602 1.591 1.588 1.580 1.571 1.564 1.552 1.547 1.542 1.526 1.518 1.504 1.045 1.012 0.984 0.975 0.955 0.943 0.889
Current Data Parameters
NAME ZIE-254-02-01
EXPNO 10
PROCNO 1
F2 - Acquisition Parameters
Date_ 20151229
Time 13.07
INSTRUM spect
PROBHD 5 mm PABBO BB-
PULPROG zg30
TD 65536
SOLVENT CDCl3
NS 16
DS 2
SWH 6188.119 Hz
FIDRES 0.094423 Hz
AQ 5.2953086 sec
RG 161
DW 80.800 usec
DE 6.50 usec
TE 296.1 K
D1 1.00000000 sec
======== CHANNEL f1 ========
NUC1 1H
P1 8.25 usec
PLW1 20.00000000 W
SFO1 300.1318534 MHz
F2 - Processing parameters
SI 65536
SF 300.1300317 MHz
WDW EM
SSB 0
LB 0.30 Hz
GB 0
PC 1.00
N
O
D7
9.5 9.0 8.5 8.0 7.5 7.0 6.5 6.0 5.5 5.0 4.5 4.0 3.5 3.0 2.5 2.0 1.5 1.0 0.5 ppm
0.90 3.02 9.38 1.00 1.01 1.01 1.01 1.13 1.24 6.56 1.46 6.39

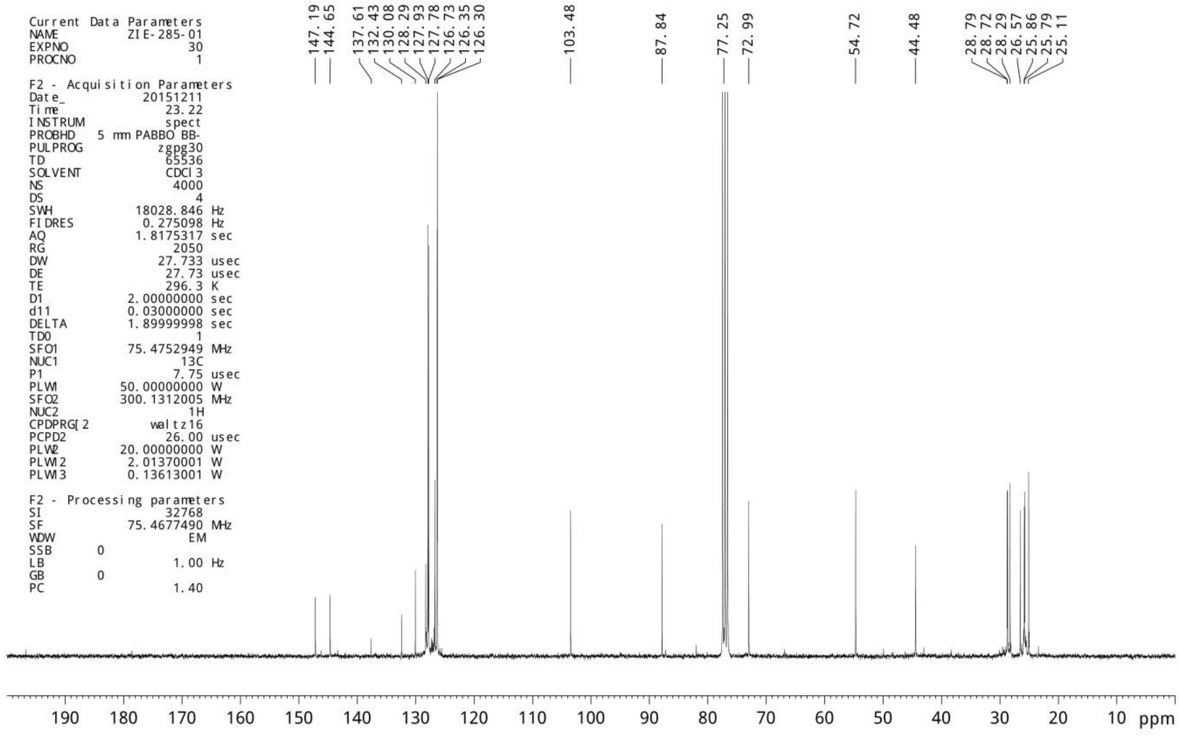
147.19 144.65 137.61 132.43 130.08 128.29 127.93 127.78 126.73 126.35 126.30 103.48 87.84 77.25 72.99 54.72 44.48 28.79 28.72 28.29 26.57 25.86 25.79 25.11
Current Data Parameters
NAME ZIE-285-01
EXPNO 30
PROCNO 1
F2 - Acquisition Parameters
Date_ 20151211
Time 23.22
INSTRUM spect
PROBHD 5 mm PABBO BB-
PULPROG zgpg30
TD 65536
SOLVENT CDCl3
NS 4000
DS 4
SWH 18028.846 Hz
FIDRES 0.275098 Hz
AQ 1.8175317 sec
RG 2050
DW 27.733 usec
DE 27.73 usec
TE 296.3 K
D1 2.00000000 sec
d11 0.03000000 sec
DELTA 1.89999998 sec
TD0 1
SFO1 75.4752949 MHz
NUC1 13C
P1 7.75 usec
PLW1 50.00000000 W
SFO2 300.1312005 MHz
NUC2 1H
CPDPRG[2 waltz16
PCPD2 26.00 usec
PLW2 20.00000000 W
PLW12 2.01370001 W
PLW13 0.13613001 W
F2 - Processing parameters
SI 32768
SF 75.4677490 MHz
WDW EM
SSB 0
LB 1.00 Hz
GB 0
PC 1.40
190 180 170 160 150 140 130 120 110 100 90 80 70 60 50 40 30 20 10 ppm

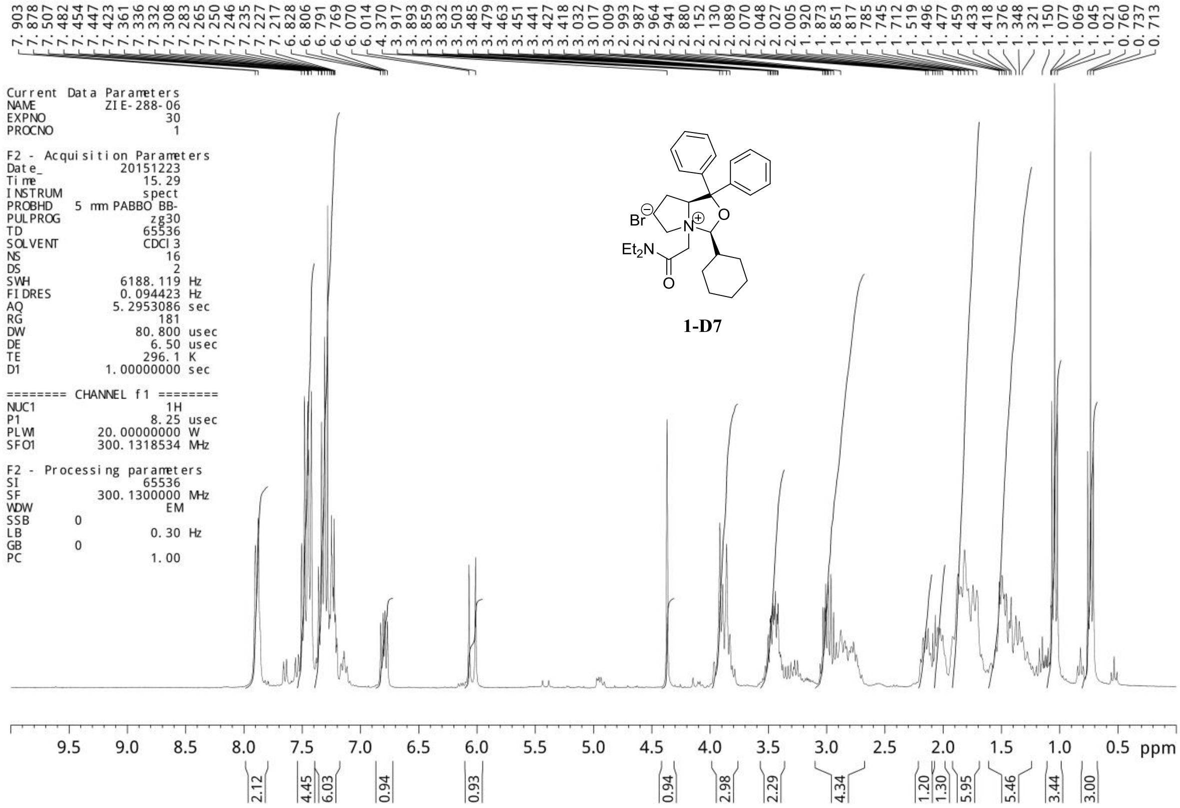
7.903 7.878 7.507 7.482 7.454 7.447 7.423 7.361 7.336 7.332 7.308 7.283 7.265 7.250 7.246 7.235 7.227 7.217 6.828 6.806 6.791 6.769 6.070 6.014 4.370 3.917 3.893 3.859 3.832 3.503 3.485 3.479 3.463 3.451 3.441 3.427 3.418 3.032 3.017 3.009 2.993 2.987 2.964 2.941 2.880 2.152 2.130 2.089 2.070 2.048 2.027 2.005 1.920 1.873 1.851 1.817 1.785 1.745 1.712 1.519 1.496 1.477 1.459 1.433 1.418 1.376 1.348 1.321 1.150 1.077 1.069 1.045 1.021 0.760 0.737 0.713
Current Data Parameters
NAME ZIE-288-06
EXPNO 30
PROCNO 1
F2 - Acquisition Parameters
Date_ 20151223
Time 15.29
INSTRUM spect
PROBHD 5 mm PABBO BB-
PULPROG zg30
TD 65536
SOLVENT CDCl3
NS 16
DS 2
SWH 6188.119 Hz
FIDRES 0.094423 Hz
AQ 5.2953086 sec
RG 181
DW 80.800 usec
DE 6.50 usec
TE 296.1 K
D1 1.00000000 sec
======== CHANNEL f1 ========
NUC1 1H
P1 8.25 usec
PLW1 20.00000000 W
SFO1 300.1318534 MHz
F2 - Processing parameters
SI 65536
SF 300.1300000 MHz
WDW EM
SSB 0
LB 0.30 Hz
GB 0
PC 1.00
9.5 9.0 8.5 8.0 7.5 7.0 6.5 6.0 5.5 5.0 4.5 4.0 3.5 3.0 2.5 2.0 1.5 1.0 0.5 ppm
2.12 4.45 6.03 0.94 0.93 0.94 2.98 2.29 4.34 1.20 1.30 5.95 5.46 3.44 3.00

1-D7

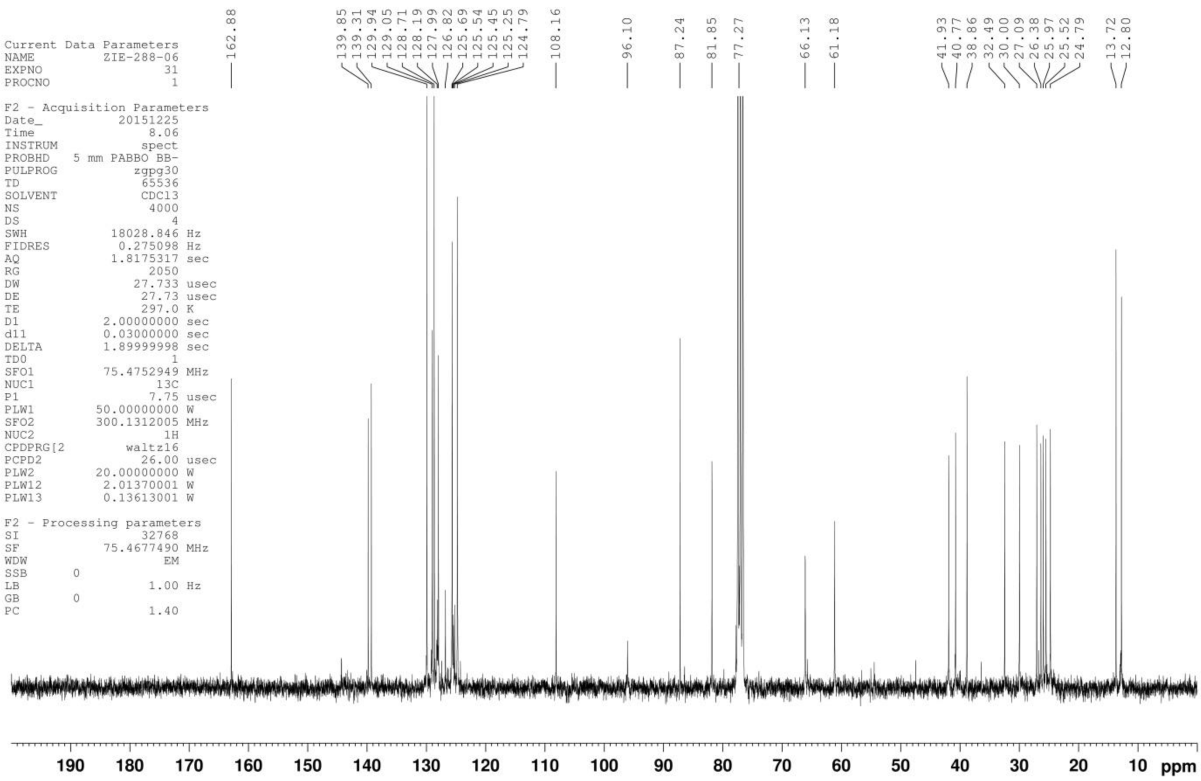
162.88 139.85 139.31 129.94 129.05 128.71 128.19 127.99 126.82 125.69 125.54 125.45 125.25 124.79 108.16 96.10 87.24 81.85 77.27 66.13 61.18 41.93 40.77 38.86 32.49 30.00 27.09 26.38 25.97 25.52 24.79 13.72 12.80
Current Data Parameters
NAME ZIE-288-06
EXPNO 31
PROCNO 1
F2 - Acquisition Parameters
Date_ 20151225
Time 8.06
INSTRUM spect
PROBHD 5 mm PABBO BB-
PULPROG zgpg30
TD 65536
SOLVENT CDC13
NS 4000
DS 4
SWH 18028.846 Hz
FIDRES 0.275098 Hz
AQ 1.8175317 sec
RG 2050
DW 27.733 usec
DE 27.73 usec
TE 297.0 K
D1 2.00000000 sec
d11 0.03000000 sec
DELTA 1.89999998 sec
TD0 1
SFO1 75.4752949 MHz
NUC1 13C
P1 7.75 usec
PLW1 50.00000000 W
SFO2 300.1312005 MHz
NUC2 1H
CPDPRG[2 waltz16
PCPD2 26.00 usec
PLW2 20.00000000 W
PLW12 2.01370001 W
PLW13 0.13613001 W
F2 - Processing parameters
SI 32768
SF 75.4677490 MHz
WDW EM
SSB 0
LB 1.00 Hz
GB 0
PC 1.40
190 180 170 160 150 140 130 120 110 100 90 80 70 60 50 40 30 20 10 ppm

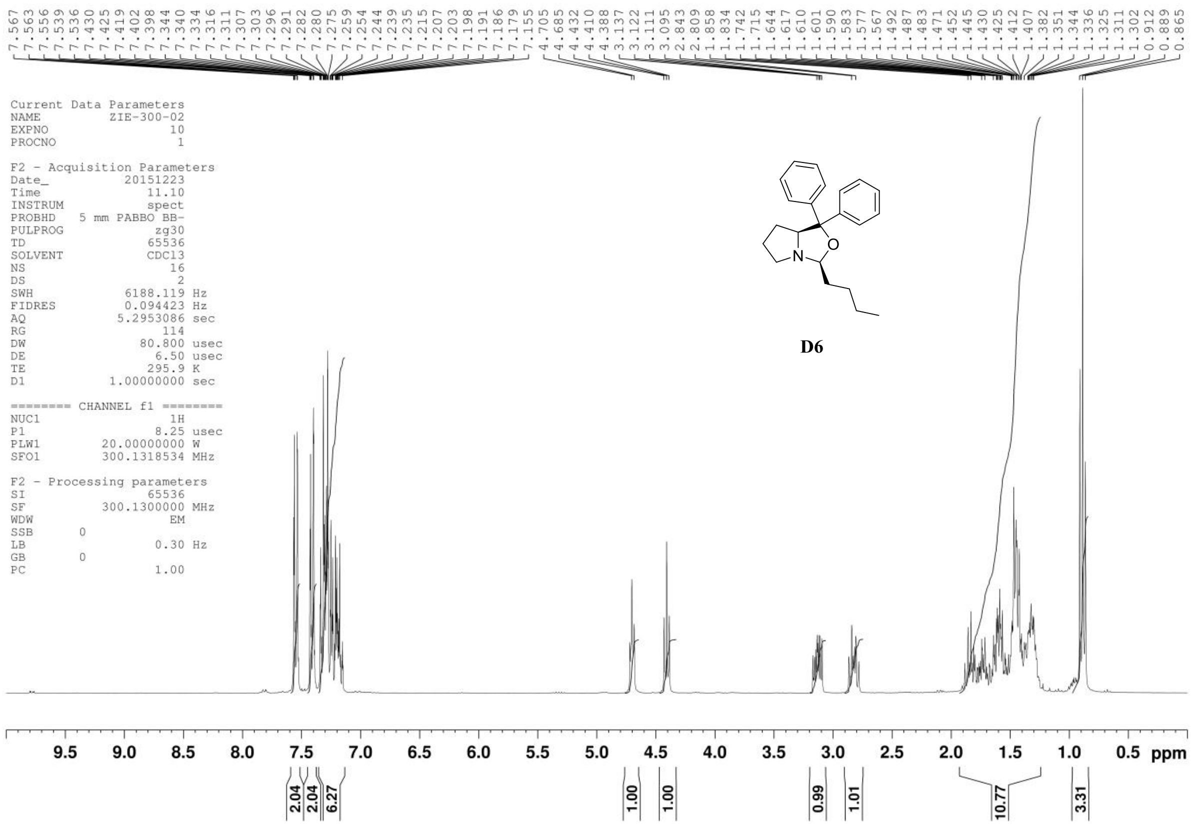
7.567 7.563 7.556 7.539 7.536 7.430 7.425 7.419 7.402 7.398 7.344 7.340 7.334 7.316 7.311 7.307 7.303 7.296 7.291 7.282 7.280 7.275 7.259 7.254 7.244 7.239 7.235 7.215 7.207 7.203 7.198 7.191 7.186 7.179 7.155 4.705 4.685 4.432 4.410 4.388 3.137 3.122 3.111 3.095 2.843 2.809 1.858 1.834 1.742 1.715 1.644 1.617 1.610 1.601 1.590 1.583 1.577 1.567 1.492 1.487 1.483 1.471 1.452 1.445 1.430 1.425 1.412 1.407 1.382 1.351 1.344 1.336 1.325 1.311 1.302 0.912 0.889 0.865
Current Data Parameters
NAME ZIE-300-02
EXPNO 10
PROCNO 1
F2 - Acquisition Parameters
Date_ 20151223
Time 11.10
INSTRUM spect
PROBHD 5 mm PABBO BB-
PULPROG zg30
TD 65536
SOLVENT CDCl3
NS 16
DS 2
SWH 6188.119 Hz
FIDRES 0.094423 Hz
AQ 5.2953086 sec
RG 114
DW 80.800 usec
DE 6.50 usec
TE 295.9 K
D1 1.00000000 sec
======== CHANNEL f1 ========
NUC1 1H
P1 8.25 usec
PLW1 20.00000000 W
SFO1 300.1318534 MHz
F2 - Processing parameters
SI 65536
SF 300.1300000 MHz
WDW EM
SSB 0
LB 0.30 Hz
GB 0
PC 1.00
N
O
D6
9.5 9.0 8.5 8.0 7.5 7.0 6.5 6.0 5.5 5.0 4.5 4.0 3.5 3.0 2.5 2.0 1.5 1.0 0.5 ppm
2.04 2.04 6.27 1.00 1.00 0.99 1.01 10.77 3.31

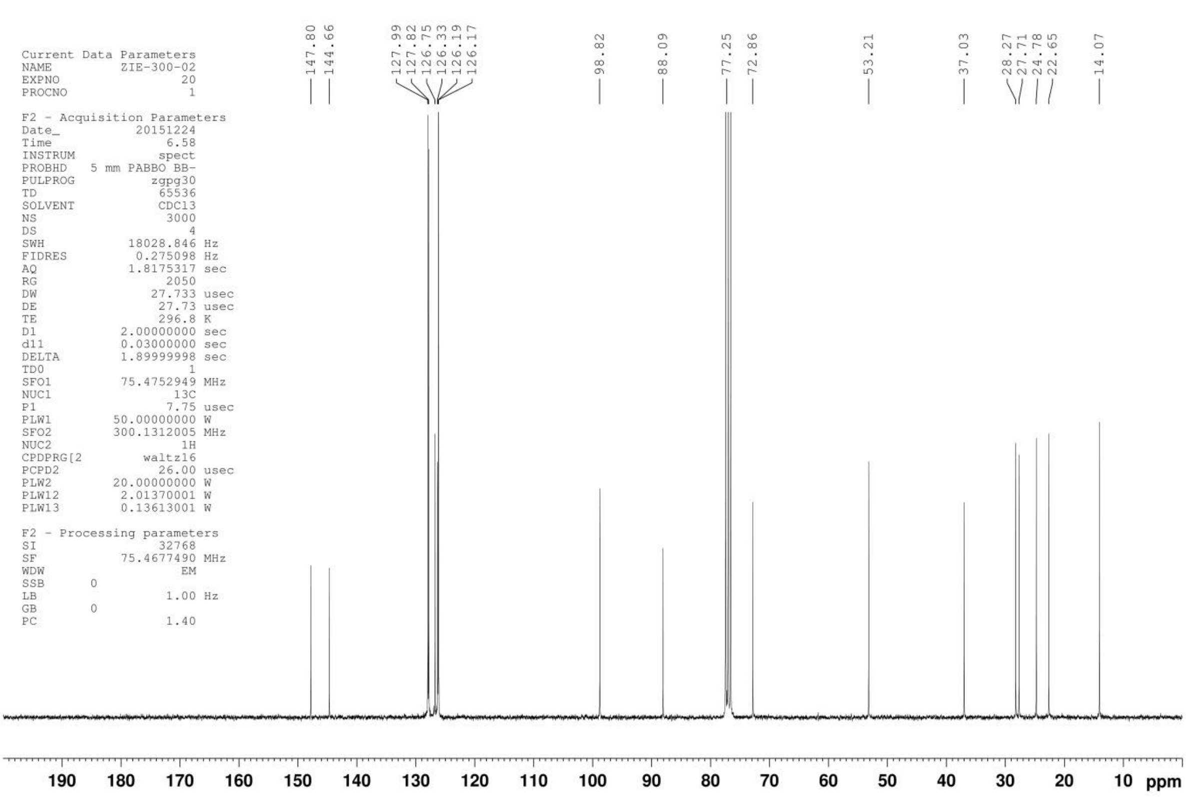
147.80 144.66 127.99 127.82 126.75 126.33 126.19 126.17 98.82 88.09 77.25 72.86 53.21 37.03 28.27 27.71 24.78 22.65 14.07
Current Data Parameters
NAME ZIE-300-02
EXPNO 20
PROCNO 1
F2 - Acquisition Parameters
Date_ 20151224
Time 6.58
INSTRUM spect
PROBHD 5 mm PABBO BB-
PULPROG zgpg30
TD 65536
SOLVENT CDCl3
NS 3000
DS 4
SWH 18028.846 Hz
FIDRES 0.275098 Hz
AQ 1.8175317 sec
RG 2050
DW 27.733 usec
DE 27.73 usec
TE 296.8 K
D1 2.00000000 sec
d11 0.03000000 sec
DELTA 1.89999998 sec
TD0 1
SFO1 75.4752949 MHz
NUC1 13C
P1 7.75 usec
PLW1 50.00000000 W
SFO2 300.1312005 MHz
NUC2 1H
CPDPRG[2 waltz16
PCPD2 26.00 usec
PLW2 20.00000000 W
PLW12 2.01370001 W
PLW13 0.13613001 W
F2 - Processing parameters
SI 32768
SF 75.4677490 MHz
WDW EM
SSB 0
LB 1.00 Hz
GB 0
PC 1.40
190 180 170 160 150 140 130 120 110 100 90 80 70 60 50 40 30 20 10 ppm

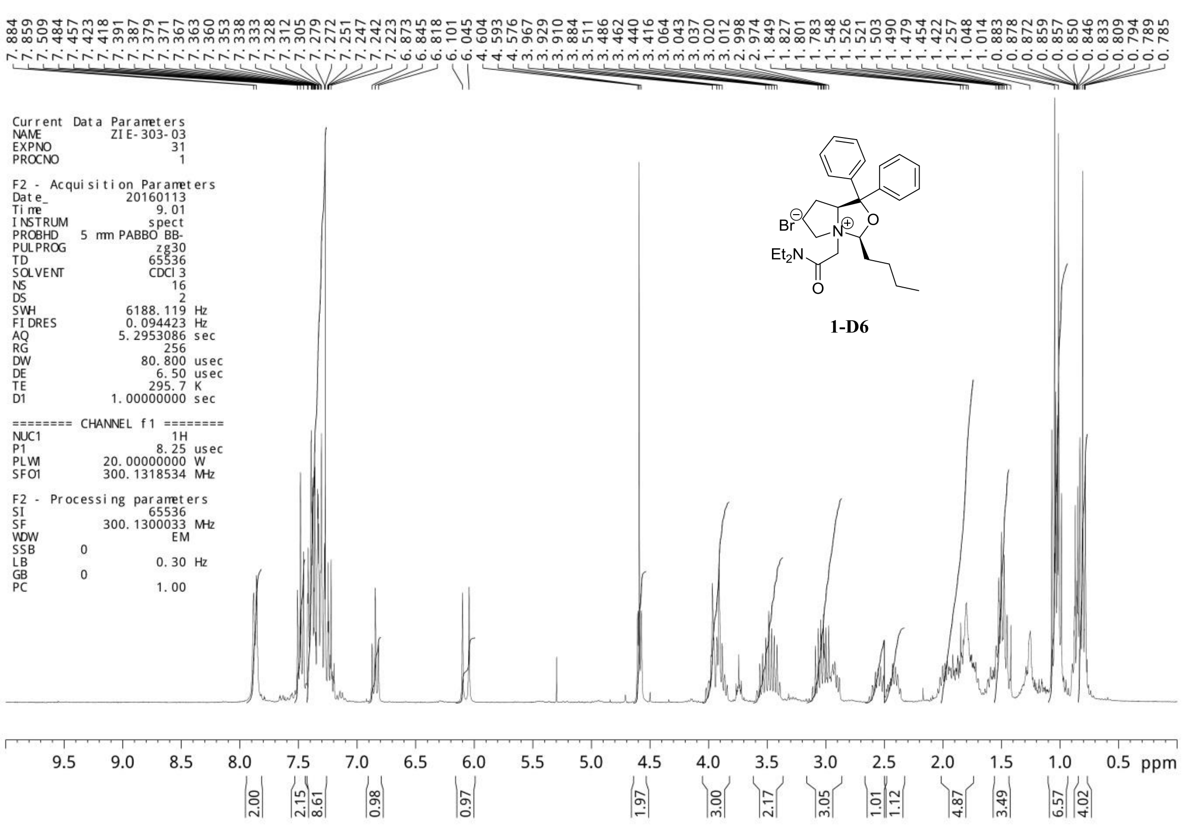
7.884 7.859 7.484 7.457 7.423 7.418 7.391 7.387 7.379 7.371 7.367 7.363 7.360 7.353 7.338 7.333 7.328 7.312 7.305 7.279 7.272 7.251 7.247 7.242 7.223 6.873 6.845 6.818 6.101 6.045 4.604 4.593 4.576 3.967 3.929 3.910 3.884 3.511 3.486 3.462 3.440 3.416 3.064 3.043 3.037 3.020 3.012 2.998 2.974 1.849 1.827 1.801 1.783 1.548 1.526 1.521 1.503 1.490 1.479 1.454 1.422 1.257 1.048 1.014 0.883 0.878 0.872 0.859 0.857 0.850 0.846 0.833 0.809 0.794 0.789 0.785
Current Data Parameters
NAME ZIE-303-03
EXPNO 31
PROCNO 1
F2 - Acquisition Parameters
Date_ 20160113
Time 9.01
INSTRUM spect
PROBHD 5 mm PABBO BB-
PULPROG zg30
TD 65536
SOLVENT CDCl3
NS 16
DS 2
SWH 6188.119 Hz
FIDRES 0.094423 Hz
AQ 5.2953086 sec
RG 256
DW 80.800 usec
DE 6.50 usec
TE 295.7 K
D1 1.00000000 sec
======== CHANNEL f1 ========
NUC1 1H
P1 8.25 usec
PLW1 20.00000000 W
SFO1 300.1318534 MHz
F2 - Processing parameters
SI 65536
SF 300.1300033 MHz
WDW EM
SSB 0
LB 0.30 Hz
GB 0
PC 1.00
Br
Et2N
O
N
O
1-D6
9.5 9.0 8.5 8.0 7.5 7.0 6.5 6.0 5.5 5.0 4.5 4.0 3.5 3.0 2.5 2.0 1.5 1.0 0.5 ppm
2.00 2.15 8.61 0.98 0.97 1.97 3.00 2.17 3.05 1.01 1.12 4.87 3.49 6.57 4.02

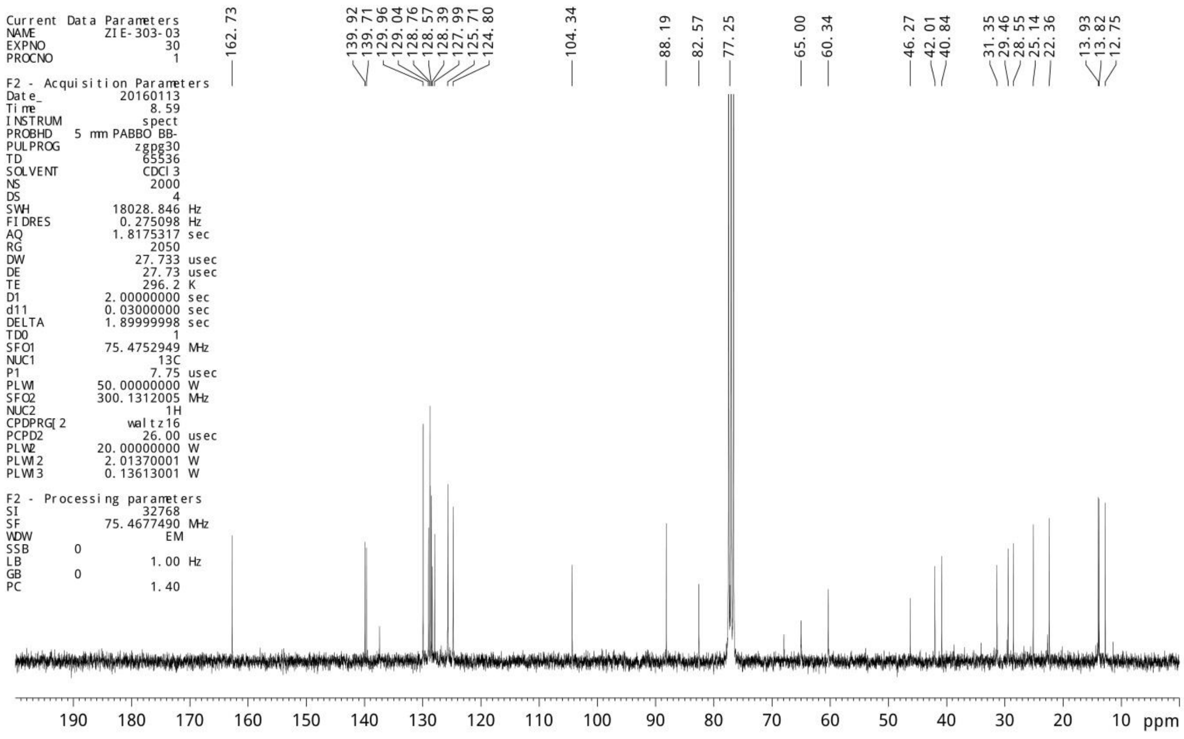
162.73 139.92 139.71 129.96 129.04 128.76 128.57 128.39 127.99 125.71 124.80 104.34 88.19 82.57 77.25 65.00 60.34 46.27 42.01 40.84 31.35 29.46 28.55 25.14 22.36 13.93 13.82 12.75
Current Data Parameters
NAME ZIE-303-03
EXPNO 30
PROCNO 1
F2 - Acquisition Parameters
Date_ 20160113
Time 8.59
INSTRUM spect
PROBHD 5 mm PABBO BB-
PULPROG zgpg30
TD 65536
SOLVENT CDCl3
NS 2000
DS 4
SWH 18028.846 Hz
FIDRES 0.275098 Hz
AQ 1.8175317 sec
RG 2050
DW 27.733 usec
DE 27.73 usec
TE 296.2 K
D1 2.00000000 sec
d11 0.03000000 sec
DELTA 1.89999998 sec
TD0 1
SFO1 75.4752949 MHz
NUC1 13C
P1 7.75 usec
PLW1 50.00000000 W
SFO2 300.1312005 MHz
NUC2 1H
CPDPRG[2 waltz16
PCPD2 26.00 usec
PLW2 20.00000000 W
PLW12 2.01370001 W
PLW13 0.13613001 W
F2 - Processing parameters
SI 32768
SF 75.4677490 MHz
WDW EM
SSB 0
LB 1.00 Hz
GB 0
PC 1.40
190 180 170 160 150 140 130 120 110 100 90 80 70 60 50 40 30 20 10 ppm
